# Synthesis of Novel Anion Recognition Molecules as Quinazoline Precursors

**DOI:** 10.3390/ijms262411975

**Published:** 2025-12-12

**Authors:** Gábor Krajsovszky, László Piros, Dóra Bogdán, Eszter Kalydi, Tamás Gáti, Pál Szabó, Péter Horváth, István M. Mándity

**Affiliations:** 1 Department of Organic Chemistry, Semmelweis University, Hőgyes Endre u. 7, H-1092 Budapest, Hungary; krajsovszky.gabor@semmelweis.hu (G.K.); pirilaci95@gmail.com (L.P.); bogdan.dora@semmelweis.hu (D.B.); kalydie@gmail.com (E.K.); 2 Artificial Transporters Research Group, Institute of Materials and Environmental Chemistry, Research Centre for Natural Sciences, Magyar tudósok körútja 2, H-1117 Budapest, Hungary; 3 Servier Research Institute of Medicinal Chemistry (SRIMC), Záhony u. 7, H-1031 Budapest, Hungary; tamas.gati@servier.com; 4 Research Center for Natural Sciences, Structure Research Center, Magyar tudósok körútja 2, H-1117 Budapest, Hungary; szabo.pal@ttk.hu; 5 Department of Pharmaceutical Chemistry, Semmelweis University, Hőgyes Endre u. 9, H-1092 Budapest, Hungary; horvath.peter@semmelweis.hu

**Keywords:** thiourea derivatives, anion recognition, supramolecular chemistry, NMR titration, structure–activity relationship

## Abstract

Thiourea and structurally related urea derivatives are widely recognised for their ability to transport anions through hydrogen bonding interactions. The strength of these interactions correlates with the electronegativity of the ligand and the acidity of the NH hydrogens involved. Thiourea, being more acidic than urea, exhibits partial deprotonation in the presence of certain anions such as organic carboxylates, fluoride, and bromide, while remaining resistant to deprotonation by chloride. This behaviour suggests a degree of selectivity toward chloride ions. Additionally, while carbamide-containing molecules tend to aggregate—potentially reducing their ion-binding efficiency—thiourea derivatives show reduced aggregation, preserving their binding capabilities. In this study, we report the synthesis and characterisation of 21 novel thiourea derivatives obtained by reacting 2-aminobenzoylamino acid esters with various substituted phenyl isothiocyanates. Seven similar thiourea-containing molecules were made as a comparison—without the amino acids—by reacting aniline with the different phenyl isothiocyanates. The reaction kinetics were found to be influenced primarily by the electronic nature of the substituents on the phenyl ring. Electron-withdrawing groups (EWGs), such as *para*-nitro, 3,5-bis(trifluoromethyl), and fluorine, accelerated the reaction, while electron-donating groups (EDGs), such as *para*-methoxy, slowed it down. Interestingly, the nature of the amino acid precursors had no significant impact on reaction time; however, reactions with aniline proceeded the fastest. Solvent choice also played a role: reactions in *N*,*N*-dimethylformamide (DMF) proceeded faster than in acetone, although with reduced yields. Consequently, reaction conditions were optimised to balance time efficiency and product yield. To evaluate the chloride ion-binding properties of the synthesised compounds, ^1^H NMR titration experiments were conducted in deuterated dimethyl sulfoxide (DMSO-*d*_6_). The association constants (K_a_) derived from these studies revealed a clear correlation with the electronic nature of the substituents. Compounds bearing EWGs exhibited enhanced chloride binding, while those with EDGs showed diminished binding affinity. Surprisingly, the presence of amino acid moieties led to a decrease in K_a_ values, despite the electron-withdrawing nature of the amide groups. This suggests that steric or conformational factors may play a role in modulating binding strength. Overall, the synthesised thiourea derivatives demonstrate mild, reversible chloride ion-binding behaviour, making them promising candidates for further development as selective anion receptors. The insights gained from this study contribute to a deeper understanding of structure–activity relationships in anion-binding systems and may inform the design of future supramolecular architectures with tailored ion recognition properties.

## 1. Introduction

Thiourea-based chloride ion transporters have emerged as a vibrant research area due to their promising applications across biological, biomedical, and therapeutic domains. These synthetic small molecules facilitate transmembrane chloride transport—an essential process for maintaining osmotic balance, regulating membrane potential, and enabling physiological functions such as neurotransmission and muscle contraction. Recent advances in the molecular design of thiourea-containing anionophores have underscored their selective chloride-binding properties, efficient transport across lipid bilayers, and potential utility as therapeutic agents.

Thiourea motifs serve as powerful hydrogen-bond donors, significantly enhancing chloride binding and enabling efficient membrane translocation [1,2]. Structural diversification strategies, including tripodal and tren-based architectures, have further improved transport efficacy. Tren-derived tripodal thioureas, incorporating multiple thiourea groups, are especially well-studied and consistently exhibit high selectivity for chloride over competing anions such as nitrate [3,4,5,6].

Steric and lipophilic tuning are now recognised as critical design parameters. Increasing steric bulk around the thiourea moiety has been shown to enhance chloride recognition and transport efficiency [2,5]. Likewise, optimising lipophilicity—often through incorporation of alkyl substituents—improves membrane compatibility and enables more effective insertion into lipid bilayers [7]. These insights highlight the importance of balancing hydrophobic character with strong anion-binding capability to achieve high transport performance.

Functionalization strategies have also expanded the utility of thiourea-based carriers. Electron-withdrawing substituents, such as fluorinated groups, have been shown to boost chloride affinity and overall transport rates [8]. Hybrid designs combining thiourea elements with heterocycles, macrocyclic motifs, or cavity-forming scaffolds have yielded next-generation anionophores with enhanced selectivity and transmembrane transport characteristics [9].

Beyond simple uniport, many thiourea-based systems mediate more sophisticated ion-exchange processes. Notably, several derivatives facilitate chloride/bicarbonate antiport—an activity highly relevant to epithelial ion homeostasis and pathologies such as cystic fibrosis, where defective chloride transport underlies disease progression [3,7,10,11].

More recently, biomedical investigations have revealed that thiourea-based transporters can disrupt intracellular ion gradients, triggering apoptosis in cancer cells and positioning these molecules as potential anticancer agents [12,13,14]. Such findings underscore their dual roles as both mechanistic tools for probing ion dysregulation and as therapeutic leads with translational potential.

Furthermore, novel applications of thiourea-based transporters have expanded into the fields of sensor development and the synthesis of complex molecular constructs for targeted drug delivery systems [15,16]. Their ability to form stable complexes with anions and modulate ionic concentrations makes them excellent candidates for designing smart drug delivery systems responsive to environmental cues, such as pH and ion concentration.

The aim of this study is to investigate the design, synthesis, and chloride-transport properties of thiourea-based anionophores with improved selectivity and membrane activity. We seek to elucidate how structural features—such as steric tuning and functional group modification—govern chloride binding.

## 2. Results and Discussion

In the first step of the synthesis route, isatoic anhydride (**1**) was reacted with three different amino acids (l-valine, l-leucine, and l-isoleucine) in water in the presence of triethylamine (TEA) to yield *N*-substituted *ortho*-aminobenzoic acid derivatives (**2**–**4**) shown in Table 1. These three amino acids were chosen due to their relatively high membrane permeability [17] and because the neutral side chains enabled the use of simple synthesis conditions. However, later, TEA was changed to NaOH_(aq)_ (2M), because its presence as a buffer made it difficult to extract the product. Reactions were carried out according to Bakavoli et al. [18].

**Scheme 1:**
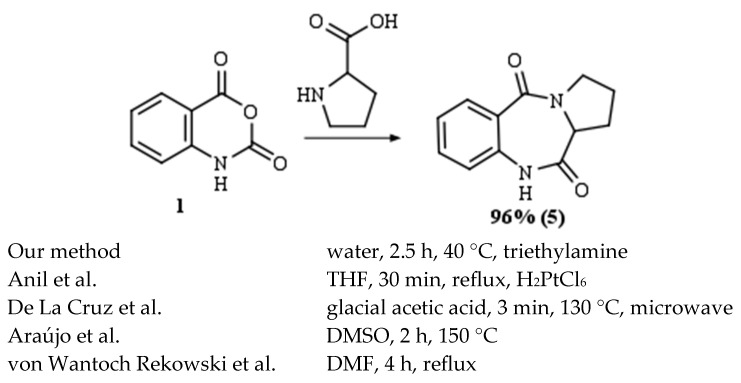
Synthesis of a benzodiazepine derivative from l-proline compared to Anil et al. [24], De La Cruz et al. [25], Araújo et al. [26] and von Wantoch Rekowski et al. [27].

This reaction was also investigated with two other amino acids. l-lysine did not react selectively under these conditions without amino-group protection. The reaction, had it proceeded, could have yielded another free amino group for further useful synthesis [19]. According to TLC, in the reaction carried out under conditions discussed above, various products were formed. Then, a controlled mildly acidic environment (pH 5) was tested with a buffer in the *N*-acylation of lysine [20]. Unfortunately, this approach was unsuccessful. The theory behind our idea indicates that the deprotonated α-amino group attacks the carbon atom of the carboxyl group. That is, our goal was to keep the α-amino group neutral (amino form), while the ε-amino group could still be protonated (ammonium form). However, the respective proximate pK_a_ values of 8.95 [21] and 10.53 [21] suggest that solely by pH adjustment, little to no selectivity could be achieved. Testing triethylamine (a pK_a_ of 10.8), [22] both amino groups were deprotonated, resulting in an unselective reaction. Similarly, the reaction with l-proline, a secondary amine (Scheme 1), yielded an interesting [23] ring molecule (**5**). Fortunately enough, our synthesis proved to be environmentally more benign than other approaches, since the synthesis took place in 2.5 h in water at 40 °C. In contrast, in a rather similar synthesis, tetrahydrofuran (THF) was refluxed for 30 min [24]. Note that the stereochemistry of the reaction was not investigated. In other methods, considerably higher temperatures (over 100 °C) and more toxic solvents such as acetic acid [25], DMSO [26], and DMF [27] were applied. Bakavoli et al. [18] introduced a two-step synthesis route for the synthesis of similar 1,4-benzodiazepines from isatoic anhydride and amino acids. In the first step, *N*-substituted *ortho*-aminobenzoic acid derivatives (**2**, **3,** and others) are formed, and the following step includes the synthesis of the heterocycle with POCl_3_ under reflux. In this article, results of primary amines are discussed. Obviously, when secondary amines are reacted, the reaction steps could be reduced to one (Scheme 1).

The main goal of our study was the synthesis of molecules containing both thiourea and amino acid moieties in open-chain form, because thiourea, as part of a ring system, loses the ability to bind anions. Note that 2-mercaptopyrimidine, a similar compound, shows pH-dependent binding to some heavy metals through its thiol form [28], where mainly sulphur acts as a binding site instead of the hydrogens of thiourea. The substituted amino acids (**2**–**4**) produced above were reacted with phenyl and 4-nitrophenyl isothiocyanates in acetonitrile at 40 °C. Instead of the expected open-chain thiourea, ring products, namely substituted 2-thioxoquinazolin-4-one derivatives (**6**–**8**), were formed by simultaneous cleavage of the amino acid moiety (Table 2).

**Scheme 2:**
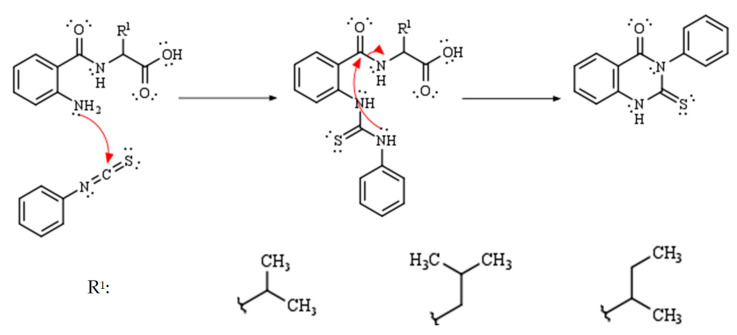
A possible reaction mechanism for the intramolecular ring closure reaction to quinazoline.

The possible reason is that higher temperatures (above 40 °C) favour ring closure and cleavage of the amino acid moiety, in contrast to lower temperatures (below 20 °C), which lead to an open-chain compound containing both the amino acid and the thiourea moieties. It can be noted here that 6-bromo-2,3-dihydro-3-phenyl-2-thioxo-4(1*H*)-quinazolinone, with a structure analogous to these compounds, was also synthesised previously, and it gave remarkable derivatives via a Suzuki cross-coupling reaction pathway [29]. The suggested mechanism for the ring closure reactions mentioned above is shown in Scheme 2. Similarly, methyl esters (**9**–**11**) undergo the same reaction shown below when reacted at 40 °C or at a higher temperature. The use of the esters was mainly for three reasons. First, they are less likely to be closed into a ring. Second, carboxyl groups could have reacted with the isothiocyanate group as well [30,31]. Third, since potential drug synthesis was also a criterion, esters have a better possible absorption [32] from the gut in comparison to acids. Quinazolinones shown in Table 2 have all been synthesised in the literature [33,34], except compound **8**, which has no literature reference.

Our next attempt was to synthesise methyl esters from the appropriate acids, carried out with thionyl chloride in excess methanol [35], as shown in Table 3.

The reason for selecting isatoic anhydride as a starting material is that it provides a common synthetic route for quinazolines described below, applying reaction circumstances under varied conditions [36,37,38,39]. Another similar reaction route includes reflux treatment of amino esters in pyridine according to Süsse et al. [40].

Initially, reactions of the methyl esters (**9**–**11**) with substituted phenyl isothiocyanates were carried out at room temperature in acetonitrile, resulting in significant formation of quinazolines (**6**–**8**). Then, when the temperature was lowered to 0 °C with an icy water bath, the open-chain form of the molecules was favoured, but the reaction time was 4–5 times slower than at room temperature. Finally, reactions were performed at room temperature, where the closed compounds were still not detected in acetone. Some of the reactions were performed in DMF as shown in Table 4, since the reaction time was found to be four-fold shorter in the case of **9a** during try-outs.

Although it is clear from Table 4 that **9e** and **11e** need a quarter of the reaction time compared to **10e,** which was carried out in acetone. The reason for using acetone as a solvent throughout was the easier and less time-consuming extraction of the product molecules in comparison to DMF. In addition, reactions in DMF produced lower yields. Overall, 21 molecules were synthesised, previously unknown in the literature. Note, however, that higher temperature —as was already mentioned above—favoured the cyclisation reaction (see a possible mechanism in Scheme 2). The reaction time was different for each substituted phenyl isothiocyanate. 4-Nitrophenyl, 3,5-bis(trifluoromethylphenyl), and 3,5-difluorophenyl isothiocyanates reacted in a time range of 2–9 h. This is considerably shorter than the transformation of phenyl isothiocyanate and 4-fluorophenyl isothiocyanate in DMF, ranging from 9 to 24 h. The reaction of **10e** was carried out in acetone and required 100 h. Reactions with 2,6-difluorophenyl isothiocyanates took place in 20–24 h. The transformation of 4-methoxyphenyl isothiocyanate was the slowest, with 76–120 h, even in DMF. The tendency in reaction times is visible, and it correlates with the data described above when aniline was used (**12a**–**12g**). There is a significant difference between the reactivity of 3,5-difluorophenyl and 2,6-difluorophenyl isothiocyanates. The latter required a two– to three-fold longer reaction time. This indicates that reactivity decreases when electron-withdrawing groups (EWG) are in the *ortho* position in comparison to that with the *meta* position. Generally, it can be stated that EWG afforded faster reactions. Transformations with the unsubstituted phenyl isothiocyanate required a four- or five-fold increase in reaction time, in accordance with previous observations [41]. The presence of an electron-donating group (EDG) significantly increased the reaction time. It is also a notable observation that in the cases of phenyl and 4-methoxyphenyl isothiocyanate, the reaction was not fully complete, as observed by TLC. As a consequence, reactions were terminated after no change was detected in the concentration of the starting materials. It is surmised that an equilibrium is reached, and the substituted amino acid remains in a stable and steady concentration. After isolation of the product by column chromatography, the reaction could be continued with the appropriate isothiocyanates. As a result, the overall yield could be improved by over 80%. This phenomenon was investigated with **10a**, giving a yield of 81% with two chromatographic purifications, which is a significant increase compared to 52–73% observed when the reaction was terminated.

**Figure 1:**
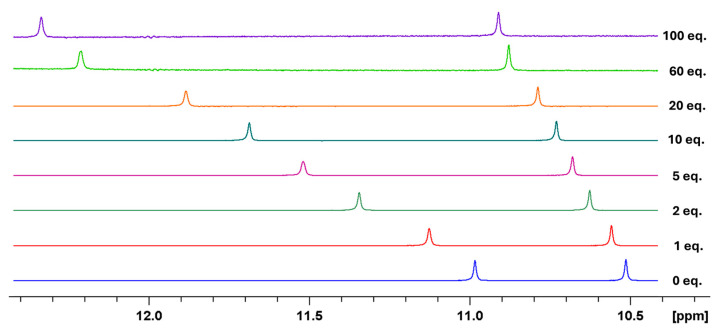
^1^H NMR titration stack plot of compound **9b** with TBA-Cl in DMSO-*d*_6_.

The association constants (K_a_) determined for chloride binding (Table 4) show that the identity of the attached amino acid has only a marginal effect on affinity. By contrast, the electronic effect of the aromatic ring is decisive. Compounds with substituents such as NO_2_, CF_3,_ and F, that exert a strong inductive electron-withdrawing (−I) effect, provide higher K_a_ values. From these substituents, NO_2_ can be highlighted as **12b** and **9b** produced the highest association constants; the titration stack plot of the latter is shown in Figure 1. The unsubstituted ring and the methoxy derivative bind chloride more weakly. The latter exerts both a –I effect and a more influential positive mesomeric effect (+M) through the unpaired electrons of the oxygen atom. An exception is the bis-*ortho*-fluoro analogue, whose affinity is unexpectedly low despite the pronounced −I character of fluorine. We ascribe this to the close proximity of two electron-rich F atoms, which likely create local electrostatic repulsion and/or steric congestion that hinders chloride coordination. Substances **9b** and **11a** were investigated for further ion interaction with ^1^H NMR titration (NO_3_^−^, SO_4_^2−^); however, no significant shift change was observed (spectra included in the Supplementary Material), indicating a lack of binding.

To put binding into context, synthetic reversible chloride ion receptors have K_a_ values between 10^2^–10^5^ M^−1^ in water [42]. However, our measurements were conducted in dimethyl sulfoxide (DMSO), and this environmental change might differ compared to the binding in water. Reversible interval for K_a_ can also be achieved in DMSO [43]. For thiourea-containing receptors, the association constant can be increased to 10^2^–10^4^ M^−1^ in acetonitrile [44]. As water provides the strongest environment for H-bonds and the bases for chloride ion binding to thiourea derivatives are basically H-bonds, this interaction should be further investigated in water to be able to determine its strength and implement its uses in biological systems. In general, it can be stated that moderate, reversible chloride-ion binding K_a_ values are between 10^1^–10^2^ M^−1^. The newly synthesised molecules had values between 10^0^–10^1^ M^−1^, which refer to weak, reversible binding.

## 3. Materials and Methods

### Supplementary Data

All commercially available chemicals were of analytical quality, and solvents were either of analytical quality or used after further purification. The reactions were monitored by thin-layer chromatography using a Merck (Darmstadt, Germany) type silica gel 60 GF 254 thin-layer plate, with the developed chromatograms visualised under 254 nm UV light. The silica gel used in the column chromatography procedures was Merck type Kieselgel (0.040–0.063 mm particle size).

Melting point determinations were performed using a Büchi (Flawil, Switzerland) M-560 melting point apparatus, and data are given in °C.

HRMS measurements were performed on a Sciex (Toronto, ON, Canada) 5600+ Triple TOF mass spectrometer. The instrument was equipped with a DuoSpray ion source, and measurements were performed under electrospray conditions with positive ion detection. The resolution of the instrument is >30,000 over the full mass range. Samples were dissolved in methanol and were injected into the ion source of the mass spectrometer at a flow rate of 0.2 mL/min of methanol. The source temperature was 350 °C. The mass range was 100–1000, with an accumulation time of 1 s. The instrument was controlled using Analyst 1.7 software, and data were evaluated using PeakView 2.2 software.

IR instrument: Jasco (Tokyo, Japan) FT/IR-4600 and Jasco (Tokyo, Japan) ATR PRO ONE with PKS-Z1 ZnSe prism kit. Spectrum registration parameters: scan: 32, resolution 4 cm^−1^, range: 4000–500 cm^−1^. During the spectrum registration, the following parameters were pre-set: automatic H_2_O, CO_2_ reduction; auto baseline correction, auto smoothing–moving means (with = 5); automatic ATR correction.

Nuclear magnetic resonance (^1^H NMR) measurements were carried out using a Varian MercuryPlus spectrometer (Palo Alto, CA, USA) (^1^H: 400 MHz, ^13^C: 100 MHz) and a Bruker Avance III (^1^H: 500 MHz, ^13^C: 125 MHz) spectrometer equipped with a standard and a cryogenic head, respectively. For structural assignment, samples were prepared by dissolving 10 mg of the solid compounds in 600 μL of DMSO-*d*_6_. The NMR spectra were recorded at room temperature using the ^2^H signal of the solvent as lock and tetramethylsilane as internal standard (TMS = 0 ppm), or spectra were referenced to the solvent signal (^1^H: 2.50 ppm and ^13^C: 39.50 ppm). The solvent is given in the characterisation of the compound. Chemical shift values (*δ*) are in ppm and coupling constants (*J*) in Hz. The multiplicity is given using designations commonly used in spectroscopy. Structural characterisation was performed using ^1^H, ^13^C, DEPTQ, ^1^H–^1^H COSY, gradient-selected HSQC (gHSQC), and HMBC (gHMBC) experiments. All NMR pulse sequences were taken from the VnmrJ and TopSpin experiment library.

^1^H NMR titrations were conducted for compounds 1–32 as follows: 1 mL of 10 mM solution in DMSO-*d*_6_ was prepared. A 2M solution of tetrabutylammonium chloride (TBA-Cl) was prepared by dissolving the calculated amount of TBA-Cl in 0.5 mL of the corresponding 10 mM solution of the compound under study, thereby compensating for dilution effects. A series of ^1^H NMR spectra was recorded for each compound in the presence of increasing concentrations of TBA-Cl (0–1 M). The exact concentration of TBA-Cl in each titration point was determined using signal integrals. Chemical shifts (*δ*, ppm) were referenced to the residual DMSO-*d*_6_ signal at 2.500 ppm. Binding isotherms were constructed by plotting the chemical shift changes as a function of TBA-Cl concentration, and the resulting data were fitted to a 1:1 binding model to calculate the stability (association) constants.

## 4. Conclusions

In summary, 21 novel isothiocyanates were synthesised. Reaction time was determined by the substituent on the phenyl ring of the isothiocyanate. Generally speaking, electron-withdrawing groups attached to phenylisothiocyanate (*para*-nitro, 3,5-bis(trifluoromethyl), fluorine substituents) enhanced the reaction, while electron-donating groups (*para*-methoxy) had a slowing effect. The amino acids used for the precursor had no significant or visible effect at all. Reaction time also shortened in DMF compared to acetone, albeit resulting in lower yields. Consequently, conditions were optimised to have a reasonable time–yield ratio.

The chloride ion-binding property of the above-mentioned molecules was investigated via ^1^H NMR titration. Respective K_a_ values show correlation with the EWG and EDG substituents of the isothiocyanates. Generally, EWG increases the binding property, whereas EDG decreases it. Amino acids, on the other hand, had a decreasing effect on K_a_ values despite the fact that amides are electron-withdrawing substituents. Chloride ion-binding receptors with a mild reversible character were synthesised.

## Figures and Tables

**Table 1:** Synthesis of *ortho*-aminobenzoic acid derivatives from isatoic anhydride at 40 °C oil bath temperature in water with equimolar NaOH solution for 3 h.

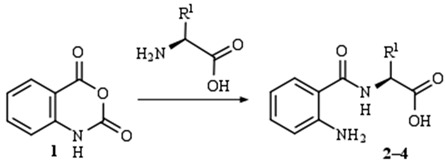			
R^1^:	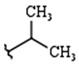	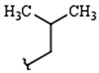	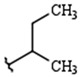
	79% (**2**)	83% (**3**)	81% (**4**)

**Table 2:** Side products of reactions intended to synthesise open-chain thiourea derivatives at 40 °C.

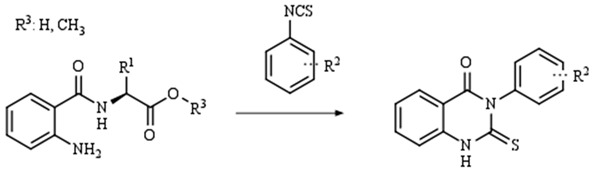			
R^1^:	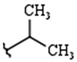	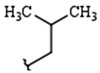	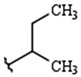
R^2^:	H (**6**)	4-NO_2_ (**7**)	3,5-diCF_3_ (**8**)

**Table 3:** Esterification of the used acids at room temperature.

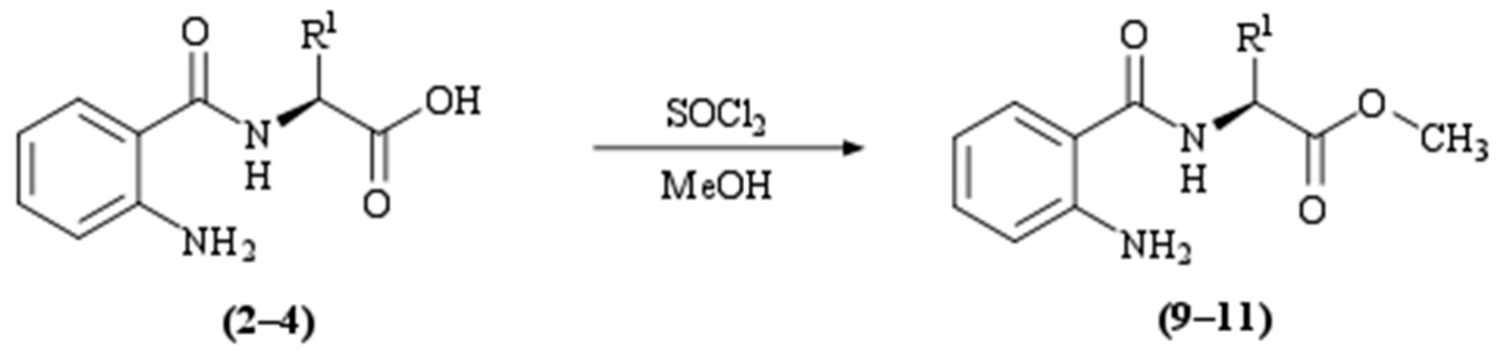			
R^1^:	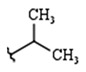	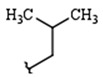	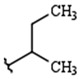
	68% (**9**)	85% (**10**)	76% (**11**)

**Table 4:** Synthesis of open-chain thiourea derivatives at room temperature in acetone or DMF with association constants (K_a_ [M^−1^]) determined for chloride binding using ^1^H NMR titration. Reactions − where solvent is not indicated − were carried out in acetone.

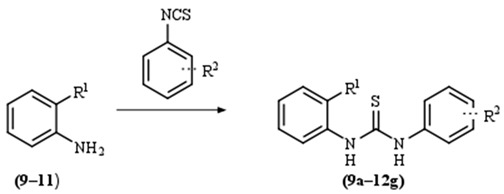					
	R^2^:	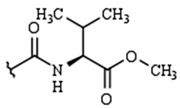	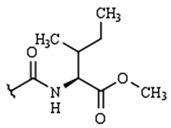	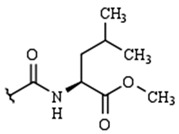	**H**
R^1^:					
H		**9a**	**10a**	**11a**	**12a**
		24 h (DMF) 52%	22 h (DMF) 73%	9 h (DMF) 54%	7 h 84%
		4.0 ± 0.1	2.4 ± 0.1	2.4 ± 0.1	22.9 ± 0.4
4-NO_2_		**9b**	**10b**	**11b**	**12b**
		7 h 93%	5 h 80%	6 h 82%	2 h 98%
		8.1 ± 0.5	6.6 ± 0.3	4.4 ± 0.2	146.6 ± 8.9
3,5-di-CF_3_		**9c**	**10c**	**11c**	**12c**
		6h 90%	2 h 63%	4 h 90%	1 h 88%
		7.1 ± 0.3	7.4 ± 0.3	7.7 ± 0.4	38.4 ± 0.4
4-CH_3_O		**9d**	**10d**	**11d**	**12d**
		120 h (DMF) 42%	82 h (DMF) 74%	76 h (DMF) 74%	6 h 68%
		2.7 ± 0.1	1.9 ± 0.1	2.6 ± 0.1	15.9 ± 0.5
4-F		**9e**	**10e**	**11e**	**12e**
		22 h (DMF) 81%	100 h 54%	24 h (DMF) 36%	2 h 88%
		4.9 ± 0.2	2.4 ± 0.1	3.7 ± 0.1	28.6 ± 0.7
3,5-di-F		**9f**	**10f**	**11f**	**12f**
		8 h 97%	9 h 77%	3 h 84%	2 h 91%
		6.9 ± 0.2	5.5 ± 0.2	6.5 ± 0.5	42.4 ± 0.7
2,6-di-F		**9g**	**10g**	**11g**	**12g**
		24 h 91%	20 h 65%	22 h 75%	2 h 83%
		2.5 ± 0.1	1.5 ± 0.1	2.8 ± 0.2	19.8 ± 0.4

Bold numbers, identifiers of the compounds.

## Data Availability

The datasets supporting the findings of this study are available as Supplementary Information.

## Supplementary Materials

The following supporting information can be downloaded at: https://www.mdpi.com/article/10.3390/ijms262411975/s1. Reference [45] is cited in the supplementary materials.
