# Supplementary material for: Synthesis of Novel Anion Recognition Molecules as Quinazoline Precursors
Source: Int J Mol Sci. 2025 Dec 12;26(24):11975. doi: 10.3390/ijms262411975 (PMC12733046; doi:10.3390/ijms262411975)
Supplement: Supplementary file 1 [file ijms-26-11975-s001.zip › ijms-3959841-supplementary.pdf]

Synthesis of novel anion recognition molecules as quinazoline precursors

Gábor Krajsovszky[a,‡], László Piros[a,‡], Dóra Bogdán[a,b], Eszter Kalydi[a], Tamás Gáti[c], Pál Szabó[d], Péter Horváth[e], István M. Mándity[a,b*]

[a] Semmelweis University, Department of Organic Chemistry
H-1092 Budapest, Hőgyes Endre u. 7.
[b] Artificial Transporters Research Group, Institute of Materials and Environmental Chemistry
Research Centre for Natural Sciences H-1117, Budapest Magyar Tudósok körútja 2.
[c] Servier Research Institute of Medicinal Chemistry (SRIMC)
H-1031 Budapest, Záhony u. 7.
[d] Research Center for Natural Sciences, Structure Research Center
H-1519 Budapest, Pf. 286.
[e] Semmelweis University, Department of Pharmaceutical Chemistry
H-1092 Budapest, Hőgyes Endre u. 9.
*E-mail: mandity.istvan@semmelweis.hu
‡ These authors contributed equaly

Supplementary data

All commercially available chemicals were of analytical quality and solvents were either of analytical quality or used after further purification. The reactions were monitored by thin-layer chromatography using a Merck type silica gel 60 GF 254 thin-layer plate, with the developed chromatograms visualised under 254 nm UV light. The silica gel used in the column chromatography procedures was Merck type Kieselgel (0.040-0.063 mm particle size).

Melting point determinations were performed using a Büchi M-560 melting point apparatus and data are given in °C.

HRMS measurements were performed on a Sciex 5600+ Triple TOF mass spectrometer. The instrument was equipped with a DuoSpray ion source and measurements were performed under electrospray conditions with positive ion detection. The resolution of the instrument is >30 000

over the full mass range. Samples were dissolved in methanol and were injected into the ion source of the mass spectrometer at a flow rate of 0.2 mL/min methanol. The source temperature was 350 °C. The mass range was 100–1000, with an accumulation time of 1 second. The instrument was controlled using Analyst 1.7 software and data were evaluated using PeakView 2.2 software.

IR instrument: Jasco FT/IR-4600. Jasco ATR PRO ONE with PKS-Z1 ZnSe prism kit. Spectrum registration parameters: Scan: 32 Resolution 4 cm^{-1}, Range: 4000-500 $cm^{-1.}$ During the spectrum registration the following parameters were pre-set: Automatic H_2O, CO_2 reduction; auto baseline correction, auto smoothing-moving means (with=5); automatic ATR correction.

Nuclear magnetic resonance (NMR) measurements were carried out using a Varian MercuryPlus spectrometer (1H: 400 MHz, 13C: 100 MHz) and a Bruker Avance III (1H: 500 MHz, 13C: 125 MHz) spectrometer equipped with a standard and a cryogenic head, respectively. For structural assignment, samples were prepared by dissolving 10 mg of the solid compounds in 600 μL of DMSO-d_6. The NMR spectra were recorded at room temperature using the 2H signal of the solvent as lock and tetrame-thylsilane as internal standard (TMS = 0 ppm) or spectra were referenced to the solvent signal (1H: 2.50 ppm and 13C: 39.50 ppm). The solvent is given in the characterization of the compound. Chemical shift values (δ) are in ppm and coupling constants (J) in Hz. The multiplicity is given using designations commonly used in spectroscopy. Struc-tural characterization was performed using 1H, 13C, DEPTQ, 1H–1H COSY, gradi-ent-selected HSQC (gHSQC) and HMBC (gHMBC) experiments. All NMR pulse se-quences were taken from the VnmrJ and TopSpin experiment library.^{1}H NMR titrations were conducted for compounds 1–32 as follows: 1 mL of 10 mM solution in DMSO-d_6 was prepared. A 2M solution of tetrabutylammonium chloride (TBA-Cl) was prepared by dissolving the calculated amount of TBA-Cl in 0.5 mL of the corresponding 10 mM solution of the compound

under study, thereby compensating dilution effects. A series of ^{1}H NMR spectra were recorded for each compound in the presence of increasing concentrations of TBA-Cl (0–1 M). The exact concentration of TBA-Cl in each titration point was determined using signal integrals. Chemical shifts (δ, ppm) were referenced to the residual DMSO-d_6 signal at 2.500 ppm. Binding isotherms were constructed by plotting the chemical shift changes as a function of TBA-Cl concentration, and the resulting data were fitted to a 1:1 binding model to calculate the stability (association) constants [45].

Synthesis of amino acid derivatives

(2*S*)-2-[(2-Aminobenzoyl)amino]-3-methylbutanoic acid (2)

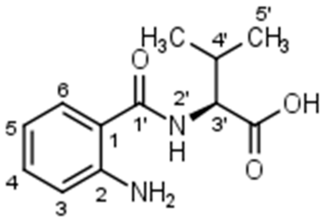

L-Valine (51 mmol, 5.98 g) was suspended in water (50.0 ml) and then $NaOH_{(aq)}$ (2M, 25.0 ml) and isatoic anhydride (50 mmol, 8.16 g) were added. Then the suspension reaction mixture was stirred at an external bath temperature of 40 °C for 3 hours, in the meantime the suspension dissolved. After the reaction time, the solution was treated with 25.0 ml 2M hydrochloric acid and extracted with 3x100.0 ml ethyl acetate (EtOAc). The combined organic phases were dried over anhydrous sodium sulfate and evaporated to give a yellowish-brown oil which was treated with 50.0 ml hexane giving yellow solid (9.36 g, 79.2%).

Mp.: 119.0–123.9 °C, R_f (Hexane:EtOAc 3:2 + 5 drops of acetic acid): long line from the start midline at around 0.15

δH (400 MHz, DMSO-d_6): 0.90 (d, *J*=6.8 Hz, 3H, H-5′), 0.92 (d, *J*=6.7 Hz, 3H, H-5'), 2.17 (m, 1H, H-4'), 4.13 (dd, *J*=7.8, 5.5 Hz, 1H, H-3'), 6.30 (brs, 2H, NH_2), 6.52 (t, *J*=7.9 Hz, 1H, H-5), 6.68 (d, *J*=7.9 Hz, 1H, H-3), 7.13 (t, *J*=7.9 Hz, 1H, H-4), 7.49 (d, *J*=7.9 Hz, 1H, H-6), 7.71 (d, *J*=7.8 Hz, 1H, H-2');

δC (100 MHz, DMSO-d_6): 18.6 (C-5'), 19.5 (C-5'), 30.2 (C-4'), 58.4 (C-3'), 114.7 (C-5), 115.3 (C-1), 116.2 (C-3), 128.2 (C-6), 131.6 (C-4), 149.3 (C-2), 168.6 (C-1'), 173.9 (*C*OOH).

(2*S*)-2-[(2-Aminobenzoyl)amino]-4-methylpentanoic acid (3)

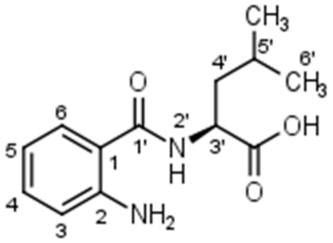

L-Leucine (51 mmol, 6.69 g) was suspended in water (50.0 ml) to which $NaOH_{(aq)}$ (2M, 25.0 ml) and isatoic anhydride (50 mmol, 8.16 g) were added, and the suspension reaction mixture was then stirred at an external bath temperature of 40 °C for 3 hours, in the meantime the suspension dissolved. After the reaction time, the solution was treated with 25.0 ml 2M hydrochloric acid and extracted with 3x100.0 ml ethyl acetate. The combined organic phases were dried over anhydrous sodium sulfate and evaporated to give a yellowish-brown oil which was treated with 50.0 ml hexane giving yellow solid (10.36 g, 82.7%).

Mp.: 60.9–75.2 °C, R_f (Hexane:EtOAc 3:2 + 5 drops of acetic acid): long line from the start midline at around 0.15

δH (400 MHz, DMSO-d_6): 0.93-0.85 (m, 6H, H-6'), 1.74-1.51 (m, 3H, H-4', H-5'), 4.30 (m, 1H, H-3'), 6.35 (brs, 2H, NH_2) 6.50 (t, *J*=7.7 Hz, 1H, H-5), 6.67 (d, *J*=7.7 Hz, 1H, H-3), 7.12 (t, *J*=7.7Hz, 1H, H-4), 7.50 (d, *J*=7.7 Hz, 1H, H-6), 7.98 (d, *J*=8.0 Hz, 1H, H-2');

δC (100 MHz, DMSO-d_6): 21.6 (C-6'), 23.2 (C-6'), 24.7 (C-5'), 40.4 (C-4'), 51.4 (C-3'), 114.5 (C-5), 115.0 (C-1), 116.2 (C-3), 128.2 (C-6), 131.6 (C-4), 149.5 (C-2), 168.5 (C-1'), 175.2 (*C*OOH).

(2*S*)-2-[(2-Aminobenzoyl)amino]-3-methylpentanoic acid (4)

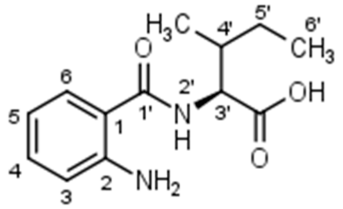

L-Isoleucine (51 mmol, 6.69 g) was suspended in water (50.0 ml) to which $NaOH_{(aq)}$ (2M, 25.0 ml) and isatoic anhydride (50 mmol, 8.16 g) were added, and the suspension reaction mixture was then stirred at an external bath temperature of 40 °C for 3 hours. In the meantime, the suspension dissolved. After the reaction time, the solution was treated with 25.0 ml 2M hydrochloric acid and extracted with 3x100.0 ml ethyl acetate. The combined organic phases were dried over anhydrous sodium sulfate and evaporated to give a yellowish-brown oil which was treated with 50.0 ml hexane giving yellow solid (10.19 g, 81.4%).

Mp.: 83.8–83.9 °C, R_f (Hexane:EtOAc 3:2 + 5 drops of acetic acid): long line from the start midline at around 0.15

L-Isoleucine (22 mmol, 2.90 g) was suspended in water (28.0 mL) to which triethylamine (22 mmol, 2.22 g, 3.0 mL) and isatoic anhydride (20 mmol, 3.26 g) were added, and the suspension reaction mixture was then stirred at an external bath temperature of 40 °C for 2 hours. After the reaction time, the solution was adjusted to pH 7 with 2M hydrochloric acid and extracted with 3x30 ml dichloromethane followed by 3x30 ml ethyl acetate. The combined organic phases were dried over anhydrous sodium sulfate and evaporated to give a yellowish-brown oil as the crude product (4.95 g, 99%).

R_f (chloroform/methanol 93:7): 0.64

δH (400 MHz, DMSO-d_6): 0.92-0.82 (m, 6H, H-6', CHCH_3), 1.23 (m, 1H, H-5'), 1.50 (m, 1H, H-5'), 1.90 (m, 1H, H-4'), 4.17 (dd, *J*=7.6, 6.0 Hz, 1H, H-3'), 6.30 (brs, 2H, NH_2), 6.52 (t, *J*=7.8 Hz, 1H, H-5), 6.68 (d, *J*=7.8 Hz, 1H, H-3), 7.13 (t, *J*=7.8 Hz, 1H, H-4), 7.48 (d, *J*=7.8 Hz, 1H, H-6), 7.81 (d, *J*=7.6 Hz, 1H, H-2');

δC (100 MHz, DMSO-d_6): 11.5 (C-6'), 15.8 (CH*C*H_3), 25.2 (C-5'), 36.5 (C-4'), 57.4 (C-3'), 114.7 (C-5), 115.1 (C-1), 116.2 (C-3), 128.2 (C-6), 131.7 (C-4), 149.4 (C-2), 168.5 (C-1'), 173.7 (*C*OOH).

2,3-Dihydro-1*H*-pyrrolo[2,1-*c*][1,4]benzodiazepine-5,11(10*H*,11a*H*)-dione (5)

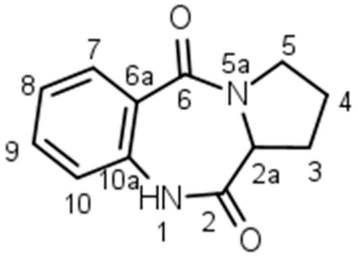

L-Proline (7.8 mmol, 0.91 g) was suspended in water (15.0 ml) to which triethylamine (15.6 mmol, 1.57 g, 2.2 ml) and isatoic anhydride (7.8 mmol, 1.28 g) were added, and the suspension reaction mixture was then stirred at 40 °C external bath temperature for 2.5 h. After the reaction time, the mixture was extracted with 3x25 ml ethyl acetate, then the combined organic phases were dried over anhydrous sodium sulfate and evaporated to give a yellowish crystalline crude product (1.92 g). This was rubbed twice with 4 ml of diethyl ether and washed with hexane, during which the multicomponent product was removed of the (upper) spots running with higher R_f values. The two main components of the crude product were separated on 100 g silica gel by column chromatography using a 10:1 mixture of dichloromethane and methanol as eluent, then the evaporated fractions, 2-5 were recrystallized from a 5:1 mixture of isopropyl

alcohol and ethanol, the product (1.14 g, 67 %) was obtained with the structure given above. Subsequently, a further amount of product (0.50 g, 29%) was separated from the filtrate (mother liquor) as a second fraction.

Mp.: 74.5–82.3°C. R_f (chloroform/methanol 40:1): 0.53

δH (500 MHz, DMSO-d_6): 1.70-2.03 (m, 3H, H-4, H-3), 2.48 (m, 1H, H-3), 3.46 (m, 1H, H-5), 3.59 (m, 1H, H-5), 4.11 (m, 1H, H-2a), 7.13 (d, *J*=7.8 Hz, 1H, H-10), 7.22 (t, *J*=7.8 Hz, 1H, H-8), 7.51 (t, *J*=7.8 Hz, 1H, H-9), 7.78 (d, *J*=7.8 Hz, 1H, H-7), 10.50 (s, 1H, NH);

δC (125 MHz, DMSO-d_6): 23.1 (C-4), 25.8 (C-3), 46.9 (C-5), 56.2 (C-2a), 121.3 (C-10), 123.9 (C-8), 126.6 (C-6a), 130.3 (C-7), 132.1 (C-9), 136.4 (C-10a), 164.5 (C-6), 170.8 (C-2).

2,3-Dihydro-3-phenyl-2-thioxo-4(1*H*)-quinazolinone (6)

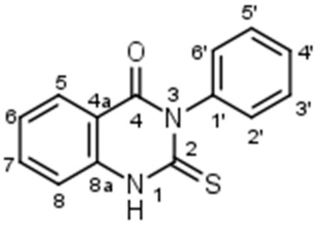

Side product during the synthesis of **10a** when the reaction proceeds at 40°C.

Substance **10** (1.0 mmol, 265 mg) was dissolved in acetonitrile (5.0 ml), then phenyl isothiocyanate (1.1 mmol, 153 mg, 135µl) was added and stirred at 40 °C for 57 hours. The solvent was evaporated, giving yellow oil. The oil was purified on a chromatographic column in hexane:ethyl-acetate 3:2 yielding yellow crystals (68 mg 27%).

δH (400 MHz, DMSO-d_6): 7.28 (d, *J*=7.3 Hz, 2H, H-2’), 7.35 (t, *J*=7.6 Hz, 1H, H-6), 7.37-7.52 (m, 4H, H-8, H-3’, H-4’, H-5′), 7.78 (t, *J*=7.6 Hz, H-7), 7.95 (m, 1H, H-5), 13.05 (s, 1H, NH);

δC (100 MHz, DMSO-d_6): 115.7 (C-8), 116.2 (C-4a), 124.4 (C-6), 127.4, (C-5) 128.1 (C-4'), 128.9 (C-3'), 129.0 (C-2'), 135.6 (C-7), 139.3 (C-1'), 139.6 (C-8a), 159.8 (C-4), 176.1 (C-2).

2,3-Dihydro-3(4-nitrophenyl)-2-thioxo-4(1*H*)-quinazolinone (7)

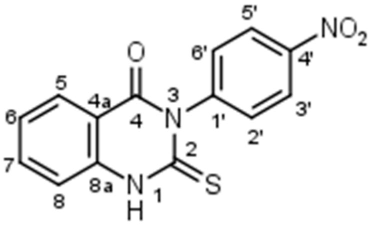

Side product during the synthesis of **9b** when the reaction proceeds at 40°C.

Substance **9** (1.0 mmol, 250 mg) was dissolved in acetonenitrile (5.0 ml), then nitrophenyl isothiocyanate (1.1 mmol, 200 mg) was added and stirred at 40 °C for 6 hours. The solvent was evaporated, giving yellow oil. The oil was purified on a chromatographic column in hexane:ethyl-acetate 3:2 yielding yellow crystals (100 mg 33%).

δH (400 MHz, DMSO-d_6): 7.35 (d, *J*=7.4 Hz, 2H, H-2', H-6`), 7.40 (d, *J*=8.9 Hz, 1H, H-8), 7.44 (m, 1H, H-4'), 7.51 (t, *J*=7.4 Hz, 2H, H-3', H-5′), 8.52 (dd, *J*=8.9, 2.1 Hz, 1H, H-7), 8.63 (d, *J*=7.1 Hz, 1H, H-5), 12.21 (s, 1H, NH);

δC (100 MHz, DMSO-d_6): 114.6 (C-4a), 116.6 (C-8), 123.7 (C-5), 128.4 (C-4'), 128.9 (C-2'), 128.9 (C-3'), 129.8 (C-7), 135.2 (C-1'), 141.9 (C-6), 144.6 (C-8a), 149.9 (C-2), 161.1 (C-4).

2,3-Dihydro-3-[3,5-bis(trifluoromethyl)phenyl]-2-thioxo-4(1*H*)-quinazolinone (8)

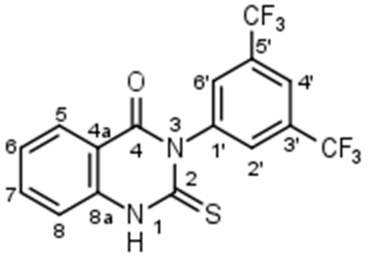

Side product during the synthesis of **10c** when the reaction proceeds at 40°C.

Substance **10** (1.0 mmol, 265 mg) was dissolved in acetonitrile (5.0 ml), then 3,5-bis(trifluoromethyl)phenyl isothiocyanate (1.1 mmol, 297 mg, 380 µl) was added and stirred at 40 °C for 7 hours. The solvent was evaporated, giving yellow oil. The oil was purified on a chromatographic column in hexane:ethyl-acetate 3:2 yielding yellow crystals (132 mg 34%).

δH (500 MHz, DMSO-d_6): 7.38 (m, 1H, H-6), 7.48 (dm, *J*=8.2 Hz, H-8), 7.82 (m, 1H, H-7), 7.97 (dm, *J*=7.9 Hz, H-5), 8.21 (m, 1H, H-4'), 8.23 (m, 2H, H-2', H-6′), 13.24 (s, 1H, NH);

δC (125 MHz, DMSO-d_6): 115.9 (C-8), 116.4 (C-4a), 122.3 (m, C-4'), 123.0 (q, *J*=272.8 Hz, CF_3), 124.6 (C-6), 127.4 (C-5), 130.8 (C-3'), 131.1 (m, C-2'), 135.9 (C-7), 139.7 (C-8a), 141.4 (C-1'), 159.9 (C-4), 175.7 (C-2).

Methyl (2*S*)-2-[(2-aminobenzoyl)amino]-3-methylbutanoate (9)

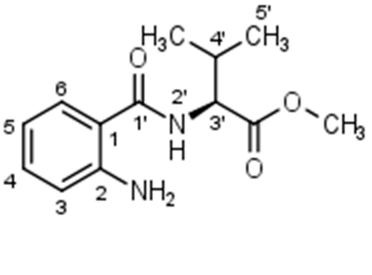

2 (12 mmol, 2.83 g) was dissolved in methanol (15.0 ml) and thionyl chloride (25 mmol, 2.95 g, 1.8 ml) was added dropwise through a dropping funnel, taking care not to boil the solution. The reaction mixture was then stirred at room temperature for 4 hours, then the mixture was set

to pH 10 with 35.0 ml cc. Na_2CO_3 solution. Extraction was carried out with 3x50.0 ml EtOAc, the solvent was evaporated, dissolved in 50.0 ml EtOAc and was extracted with 50.0 ml of pH 10 water, the organic phase was dried on anhydrous sodium sulfate and evaporated. The yellow liquid was treated with 2.0 ml EtOAc and 40.0 ml hexane, yielding white solid (2.05 g, 68.2%).

Mp.: 84.4–86.8°C, R_f (Hexane:EtOAc 3:2): 0.50

δH (400 MHz, $CDCl_3$): 0.99 (d, *J*=7.2 Hz, 3H, H-5'), 1.01 (d, *J*=7.2 Hz, 3H, H-5'), 2.26 (m, 1H, H-4'), 3.78 (s, 3H, OCH_3), 4.73 (dd, *J*=8.3, 5.0 Hz, 1H, H-3'), 6.55 (d, *J*=8.3 Hz, 1H, H-2'), 6.74-6.64 (m, 2H, H-3, H-5), 7.23 (t, *J*=7.6 Hz, 1H, H-4), 7.42 (d, *J*=7.6 Hz ,1H, H-6);

δC (100 MHz, $CDCl_3$): 18.0 (C-5'), 19.0 (C-5'), 31.5 (C-4'), 52.3 (OCH_3), 57.0 (C-3'), 115.6 (C-1), 116.7 (C-3), 117.3 (C-5), 127.4 (C-6), 132.6 (C-4), 148.7 (C-2), 169.0 (C-1'), 172.7 (*C*=O(OCH_3)).

Methyl (2*S*)-2-[(2-aminobenzoyl)amino]-4-methylpentanoate (10)

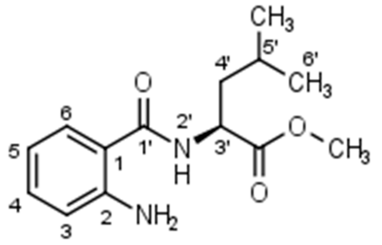

3 (12 mmol, 3.00 g) was dissolved in methanol (15.0 ml) and thionyl chloride (25 mmol, 2.95 g, 1.8 ml) was added dropwise through a dropping funnel, taking care not to boil the solution. The reaction mixture was then stirred at room temperature for 4 hours, then the mixture was set to pH 10 with 35.0 ml cc. Na_2CO_3 solution. Extraction was carried out with 3x50.0 ml EtOAc, the solvent was evaporated, dissolved in 50.0 ml EtOAc and was extracted with 50.0 ml of pH 10 water, the organic phase was dried on anhydrous sodium sulfate and evaporated. The yellow liquid was treated with 2.0 ml EtOAc and 40.0 ml hexane, yielding white solid (2.70 g, 85.2%).

Mp.: 88.5–91.4°C, R_f (Hexane:EtOAc 3:2): 0.57

δH (400 MHz, $CDCl_3$): 0.96 (d, *J*=6.5 Hz, 3H, H-6'), 0.98 (d, *J*=6.5 Hz, 3H, H-6'), 1.68 (m, 1H, H-4'), 1.72 (m, 2H, H-4', H-5'), 3.75 (s, 3H, OC*H*3), 4.83 (m, 1H, H-3'), 7.20 (t, *J*=7.5 Hz, 1H, H-5), 7.25 (d, *J*=7.5 Hz, 1H, H-3), 7.44 (tm, *J*=7.5 Hz, 1H, H-4), 8.15 (dm, *J*=7.5 Hz, 1H, H-6), 10.45 (d, *J*=6.9 Hz, 1H, H-2');

δC (100 MHz, $CDCl_3$): 22.1 (C-6'), 22.8 (C-6'), 25.1 (C-5'), 41.8 (C-4'), 50.9 (C-3'), 52.1 (O*C*H3), 120.2 (C-3), 124.5 (C-5), 127.1 (C-1), 131.5 (C-6), 132.1 (C-4), 152.7 (C-2), 166.0 (C-1'), 173.8 (*C*=O(OCH_3)).

Methyl (2*S*)-2-[(2-aminobenzoyl)amino]-3-methylpentanoate (11)

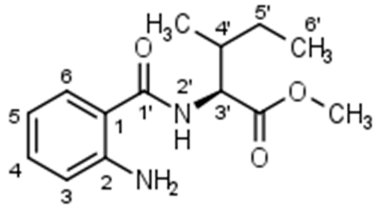

4 (12 mmol, 3.00 g) was dissolved in methanol (15.0 ml) and thionyl chloride (25 mmol, 2.95 g, 1.8 ml) was added dropwise through a dropping funnel, taking care not to boil the solution. The reaction mixture was then stirred at room temperature for 4 hours, then the mixture was set to pH 10 with 35.0 ml cc. Na_2CO_3 solution. Extraction was carried out with 3x50.0 ml EtOAc, the solvent was evaporated, dissolved in 50.0 ml EtOAc and was extracted with 50.0 ml of pH 10 water, the organic phase was dried on anhydrous sodium sulfate and evaporated. The yellow liquid was treated with 2.0 ml EtOAc and 40.0 ml hexane, yielding white solid (2.40 g, 75.8%).

Mp.: 64.6–66.1°C, R_f (Hexane:EtOAc 3:2): 0.49

δH (400 MHz, $CDCl_3$): 1.00-0.92 (m, 6H, H-6', CHC*H*3), 1.25 (m, 1H, H-5'), 1.52 (m, 1H, H-5'), 1.99 (m, 1H, H-4'), 3.77 (s, 3H, OC*H*3), 4.76 (dd, *J*=8.4, 5.0 Hz, 1H, H-3'), 6.56 (d, *J*=8.4

Hz, 1H, H-2'), 6.72-6.63 (m, 2H, H-3, H-5), 7.22 (t, *J*=8.0 Hz, 1H, H-4), 7.40 (d, *J*=8.0 Hz 1H, H-6);

δC (100 MHz, $CDCl_3$): 11.6 (C-6'), 15.5 (CH_3), 25.3 (C-5'), 38.2 (C-4'), 52.2 (OCH_3), 56.4 (C-3'), 115.6 (C-1), 116.7 (C-3), 117.3 (C-5), 127.4 (C-6), 132.6 (C-4), 148.7 (C-2), 168.9 (C-1'), 172.7 (*C*=O(OCH_3)).

Synthesis of thiourea derivatives

Methyl (2*S*)-3-methyl-2-({2-[(phenylcarbamothioyl)amino]benzoyl}amino)butanoate (9a)

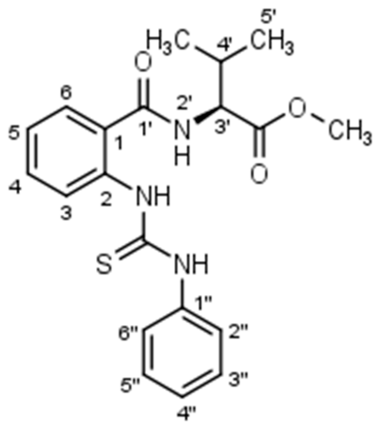

Substance **9** (1.0 mmol, 251 mg) was dissolved in dimethylformamide (DMF) (5.0 ml), then phenyl isothiocyanate (1.1 mmol, 153 mg, 135μl) was added and stirred at room temperature for 24 hours. The solvent was evaporated with toluene and the evaporation residue was treated with diethyl-ether (2.0 ml) and hexane (15.0 ml). Yellow precipitate (201 mg, 52.1%) was formed which was filtered and recrystallized from 1.2 ml MeOH, filtered, washed with hexane, dried at room temperature yielding 100 mg white crystals.

Mp.: 119.0–121.4°C. R_f (hexane:EtOAc 3:2): 0.43

IR (ZnSe) ν_{max}: 3333, 2960, 1745, 1632, 1523, 1197, 743 cm^{-1};

δH (400 MHz, DMSO-d_6): 0.92 (d, *J*=6.8 Hz, 3H, H-5'), 0.96 (d, *J*=6.8 Hz, 3H, C-5'), 2.16 (m, 1H, H-4'), 3.64 (s, 3H, OCH_3), 4.26 (t, *J*=7.3 Hz, 3H, H-3'), 7.17 (t, *J*=7.4 Hz, 1H, H-4'') 7.23 (t, *J*=7.8 Hz, 1H, H-5), 7.35 (t, *J*=7.4 Hz, 2H, H-3'', H-5''), 7.47 (d, *J*=7.4 Hz, 2H, H-2'', H-6''), 7.48 (m, 1H, H-4), 7.63 (d, *J*=7.8 Hz, 1H, H-6), 8.05 (d, *J*=7.8 Hz, 1H, H-3), 8.80 (brs, 1H, H-2'), 10.25 (s, 1H, N*H*C-2), 10.45 (s, 1H, N*H*C-1'');

δC (100 MHz, DMSO-d_6): 18.8 (C-5'), 19.1 (C-5'), 29.6 (C-4'), 51.8 (O*C*H_3), 58.4 (C-3'), 123.8 (C-2'', C-6''), 124.1 (C-5), 125.0 (C-4''), 126.4 (C-3), 126.9 (C-1), 128.5 (C-6), 128.7 (C-3'', C-5''), 130.4 (C-4), 138.3 (C-2), 138.7 (C-1''), 168.2 (C-1'), 171.8 (*C*=O(CH_3)), 179.4 (*C*S).

HRMS (ESI) $C_{20}H_{23}N_3NaO_3S$ $[M+H]^+$: calculated: 408.1352, measured: 408.1371, deviation: 4.6 ppm, 1.88 mDa, DBE: 11.0.

Methyl (2*S*)-3-methyl 2-[(2-{[(4-nitrophenyl)carbamothioyl]amino}benzoyl)amino]butanoate (9b)

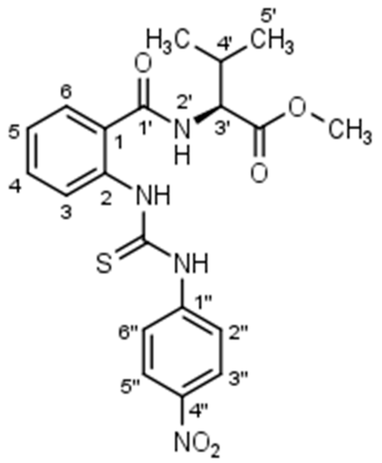

Substance **9** (1.0 mmol, 250 mg) was dissolved in acetone (5.0 ml), then nitrophenyl isothiocyanate (1.1 mmol, 200 mg) was added and stirred at room temperature for 7 hours. The solvent was evaporated; the evaporation residue was treated with diethyl-ether (2.0 ml) and hexane (15.0 ml). Yellow precipitate (402 mg, 93.3%) was formed which was filtered and 202

mg was recrystallized from 1.4 ml MeOH, filtered, washed with hexane, dried at room temperature yielding 119 mg yellow crystals.

Mp.: 144.0–147.4°C. R_f (hexane:EtOAc 3:2): 0.28

IR (ZnSe) ν_{max}: 3019, 1737, 1649, 1339, 1113, 1045, 853 cm^{-1};

δH (400 MHz, $CDCl_3$): 0.93 (d, *J*=6.7 Hz, 3H, H-5'), 0.95 (d, *J*=6.7 Hz, 3H, H-5'), 2.22 (m, 3H, H-4'), 3.74 (s, 3H, OCH_3), 4.56 (dd, *J*=8.5, 5.0 Hz, 1H, H-3'), 6.85 (d, *J*=8.5 Hz, 1H, H-2'), 7.26 (t, *J*=7.5 Hz 1H, H-5), 7.58-7.48 (m, 2H, H-4, H-6), 7.62 (d, *J*=9.0 Hz, 2H, H-2'', H-6''), 8.23 (d, *J*=9.0 Hz, 2H, H-3'', H-5''), 8.32 (d, *J*=8.3 Hz, 1H, H-3), 8.69 (s, 1H, N*H*C-1''), 10.69 (s, 1H, N*H*C-2);

δC (100 MHz, $CDCl_3$): 17.9 (C-5'), 19.0 (C-5'), 31.3 (C-4'), 52.4 (OCH_3), 57.7 (C-3'), 122.6 (C-2'', C-6''), 125.1 (C-3'', C-5''), 125.5 (C-5), 125.8 (C-1), 125.8 (C-3), 127.1 (C-6), 131.8 (C-4), 138.1 (C-2), 143.4 (C-1''), 144.4 (C-4''), 168.6 (C-1'), 172.0 (*C*=O(CH_3)), 179.3 (*C*S).

HRMS (ESI) $C_{20}H_{22}N_4NaO_5S$ $[M+H]^+$: calculated: 453.1203, measured: 453.1229, deviation: 5.7 ppm, 2.57 mDa, DBE: 12.0.

Methyl (2*S*)-3-methyl 2-[(2-{[(3,5-bistrifluoromethylphenyl)carbamothioyl]amino} benzoyl)amino]butanoate (9c)

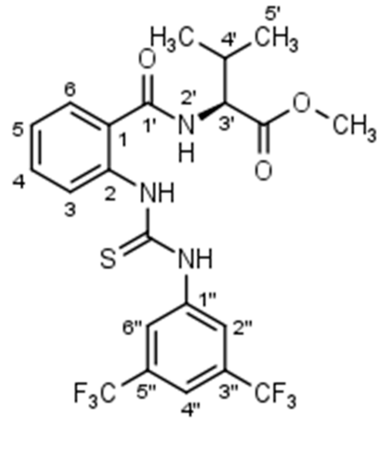

Substance **9** (1.0 mmol, 251 mg) was dissolved in acetone (5.0 ml), then 3,5-bis(trifluoromethyl)phenyl isothiocyanate (1.1 mmol, 301 mg, 385 μl) was added and stirred at room temperature for 6 hours. The solvent was evaporated; the evaporation residue was treated with diethyl-ether (2.0 ml) and hexane (15.0 ml). White precipitate (467 mg, 89.5%) was formed which was recrystallized from 3.5 ml MeOH, filtered, washed with hexane, dried at room temperature yielding 209 mg white crystals.

Mp.: 128.2–131.2°C. R_f (hexane:EtOAc 3:2): 0.54

IR (ZnSe) ν_{max}: 2962, 1709, 1621, 1524, 1469, 1275, 1121, 760 cm^{-1};

δH (400 MHz, $CDCl_3$): 0.91 (d, *J*=6.7 Hz, 3H, H-5'), 0.93 (d, *J*=6.7 Hz, 3H, H-5'), 2.22 (m, 1H, H-4'), 3.73 (s, 3H, OCH_3), 4.55 (dd, *J*=8.4, 4.9 Hz, 1H, H-3'), 6.98 (d, *J*=8.4 Hz, 1H, H-2'), 7.26 (m, 1H, H-5), 7.57-7.46 (m, 2H, H-4, H-6), 7.69 (m, 1H, H-4''), 7.96 (m, 2H, H-2'', H-6''), 8.89 (s, 1H, N*H*C-1''), 10.31 (s, 1H, N*H*C-2);

δC (100 MHz, $CDCl_3$): 17.8 (C-5'), 18.9 (C-5'), 31.1 (C-4'), 52.4 (OCH_3), 57.8 (C-3'), 119.2 (m, C-4''), 123.0 (q, *J*=272.6 Hz, CF_3), 124.4 (m, C-2'', C-6''), 125.9 (C-5), 126.5 (C-3), 127.3 (C-6), 127.4 (C-1), 131.8 (C-4), 132.1 (q, *J*=33.6 Hz,C-3'', C-5''), 137.5 (C-2), 139.3 (C-1''), 168.9 (C-1'), 172.0 (*C*=O(OCH_3)), 180.4 (*C*S).

HRMS (ESI) $C_{22}H_{21}F_6N_3NaO_3S$ $[M+H]^+$: calculated: 544.1100, measured: 544.1125, deviation: 4.6 ppm, 2.51 mDa, DBE: 11.0.

Methyl (2*S*)-3-methyl 2-[(2-{[(4-methoxyphenyl)carbamothioyl]amino}benzoyl)amino]butanoate (9d)

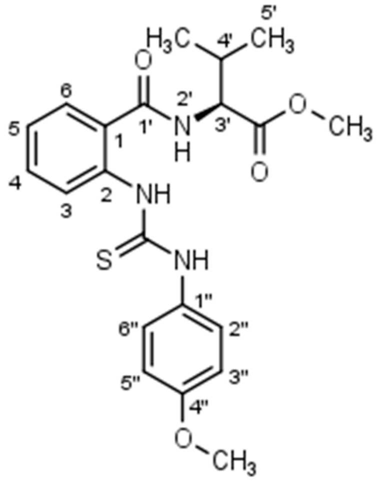

Substance **9** (2.0 mmol, 500 mg) was dissolved in DMF (5.0 ml), then 4-methoxyphenyl isothiocyanate (3.3 mmol, 544 mg, 455 µl) was added and stirred at room temperature for 120 hours. The solvent was evaporated with toluene; the evaporation residue was treated with diethyl-ether (2.0 ml) and hexane (15.0 ml). Yellow precipitate (301 mg, 36.3%) was formed which was purified on a chromatographic column with hexane:EtOAc 3:2 resulting in 115 mg white crystal after evaporation.

Mp.: 119.0–120.9°C. R_f (hexane:EtOAc 3:2): 0.29

IR (ZnSe) ν_{max}: 3217, 2916, 1737, 1658, 1507, 1240, 1031, 758 cm^{-1};

δH (500 MHz, DMSO-d_6): 0.94 (d, *J*=6.7 Hz, 1H, H-5'), 0.97 (d, *J*=6.7 Hz, 3H, H-5'), 2.17 (m, 1H, H-4'), 3.66 (s, 3H, COOCH_3), 3.75 (s, 3H, 4''-COCH_3), 4.24 (t, *J*=7.3 Hz, 1H, H-3'), 6.93 (m, 2H, H-3'', H-5''), 7.21 (m, 1H, H-5), 7.33 (m, 2H, H-2'', H-6''), 7.47 (m, 1H, H-4,), 7.63 (dd, *J*=8.0, 1.2 Hz, 1H, H-6), 8.13 (d, *J*=8.0 Hz, 1H, H-3), 8.79 (d, *J*=7.3 Hz, 1H, H-2'), 10.18 (s, 1H, N*H*C-2), 10.26 (s, 1H, N*H*C-1'');

δC (125 MHz, DMSO-d_6): 18.8 (C-5'), 19.1 (C-5'), 29.6 (C-4'), 51.7 (O*C*H_3), 55.2 (4''-O*C*H_3), 58.4 (C-3'), 114.0 (C-3'', C-5''), 123.8 (C-5), 126.1 (C-3), 126.1 (C-2'', C-6''), 126.5 (C-1), 128.4 (C-6), 130.3 (C-4), 131.2 (C-1''), 138.5 (C-2), 157.0 (C-4''), 168.3 (C-1'), 171.8 (*C*=O(OCH_3)179.5 (*C*S).

HRMS (ESI) $C_{21}H_{26}N_3O_4S$ $[M+H]^+$: calculated: 416.1639, measured: 416.1647, deviation: 2.0 ppm, 1.83 mDa, DBE: 11.0.

Methyl (2*S*)-3-methyl 2-[(2-{[(4-fluorophenyl)carbamothioyl]amino}benzoyl)amino]butanoate (9e)

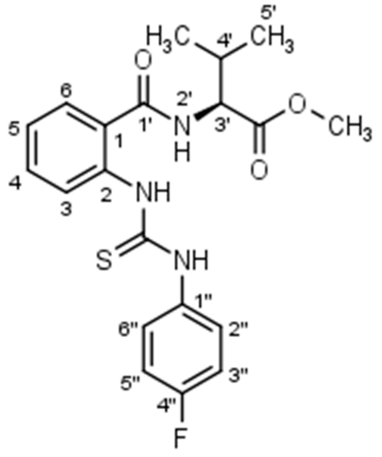

Substance **9** (1.0 mmol, 250 mg) was dissolved in DMF (5.0 ml), then 4-fluorophenyl isothiocyanate (1.1 mmol, 169 mg) was added and stirred at room temperature for 22 hours. The solvent was evaporated with toluene; the evaporation residue was treated with diethyl-ether (2.0 ml) and hexane (15.0 ml). Yellow precipitate (326 mg, 80.8%) was formed, 205 mg was recrystallized from 1.0 ml MeOH, filtered, washed with hexane, dried at room temperature yielding 109 mg white crystals.

Mp.: 124.9–128.9°C. R_f (hexane:EtOAc 3:2): 0.41

IR (ZnSe) ν_{max}: 3332, 2968, 1738, 1523, 1504, 1350, 1207, 744 cm^{-1};

δH NMR (400 MHz, DMSO-d_6): 0.93 (d, J = 6.7 Hz, 3H, H-5'), 0.96 (d, J = 6.8 Hz, 3H, CH_3), 2.05 – 2.26 (m, 1H, H-4'), 3.65 (s, 3H, OCH_3), 4.28 (t, J = 7.2 Hz, 1H, H-3'), 7.11 – 7.33 (m, 3H, H-5, H-3'', H-5''), 7.40 – 7.55 (m, 3H, H-4, H-2'', H-6''), 7.64 (d, J = 7.8 Hz, 1H, H-6), 8.06 (d, J = 8.2 Hz, 1H, H-3), 8.76 (d, J = 7.7 Hz, 1H, H-2'), 10.25 (s, 1H, NH, NHC-1''), 10.39 (s, 1H, NH, NHC-2).

δC NMR (101 MHz, DMSO-d_6): 18.7 (CH3), 19.0 (C-5'), 29.6 (C-4'), 51.7 (C-3'), 58.4 (OCH3), 115.3 (d, J=22.5 Hz, 2C, C-3'', C-5''), 124.1 (C-5), 126.2 (C-3), 126.3 (2C, C-2'', C-6''), 126.9 (C-1), 128.5 (C-6), 130.4 (C-4), 135.0 (C-1''), 138.2 (C-2), 159.4 (d, J=242.2 Hz, C-4''), 168.2 (C-1'), 171.7 (CO), 179.7 (CS).

HRMS (ESI) $C_{20}H_{23}N_3O_3FS$ $[M+H]^+$: calculated: 404.1438, measured: 404.1425, deviation: 4.7 ppm, 1.92 mDa, DBE: 10.5.

Methyl (2*S*)-3-methyl 2-[(2-{[(3,5-difluorophenyl)carbamothioyl]amino}benzoyl)amino]butanoate (9f)

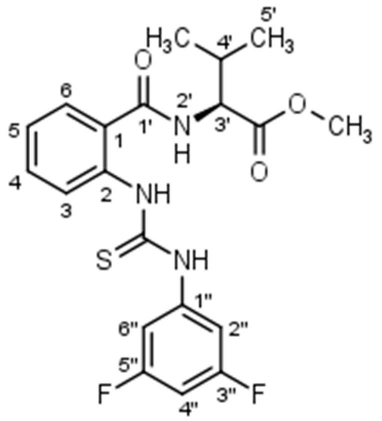

Substance **9** (1.0 mmol, 251 mg) was dissolved in acetone (5.0 ml), then 3,5-difluorophenyl isothiocyanate (1.1 mmol, 183 mg, 145 µl) was added and stirred at room temperature for 8 hours. The solvent was evaporated, meanwhile white precipitate (407 mg, 96.6%) appeared and 201 mg was recrystallized from 7.5 ml MeOH, filtered, washed with hexane, and dried at room temperature yielding 125 mg low density white crystals.

Mp.: 152.2–158.4°C. R_f (hexane:EtOAc 3:2): 0.46

IR (ZnSe) ν_{max}: 3406, 3177. 2967, 1739, 1617, 1475, 729 cm^{-1};

δH (400 MHz, DMSO-d_6): 0.92 (d, J = 6.7 Hz, 3H, H-5'), 0.95 (d, J = 6.8 Hz, 3H, C*H*3), 2.05 – 2.24 (m, 1H, H-4'), 3.64 (s, 3H, OC*H*3), 4.29 (t, J = 7.2 Hz, 1H, H-3'), 6.99 (t, J = 9.4 Hz, 1H, H-4''), 7.28 (t, J = 7.6 Hz, 1H, H-5), 7.37 (d, J = 8.9 Hz, 2H, H-2'',H-6''), 7.50 (t, J = 7.9 Hz, 1H, H-4), 7.65 (d, J = 7.7 Hz, 1H, H-6), 7.94 (d, J = 8.2 Hz, 1H, H-3), 8.76 (d, J = 7.7 Hz, 1H, H-2'), 10.39 (s, 1H, NH, N*H*C-2), 10.68 (s, 1H, NH, N*H*C-1'').

δC (101 MHz, DMSO-d_6): 18.7 (C-5'), 19.0 (*C*H3), 29.6 (C-4'), 51.7 (O*C*H3), 58.4 (C-3'), 99.6 (t, J = 24.5 Hz, C-4''), 105.8 (m, 2C, C-2'',C-6''), 124.9 (C-5), 126.8 (C-3), 128.0 (C-1), 128.8 (C-6), 130.7 (C-4), 137.9 (C-2), 142.0 (t, J=13.4 Hz, C-1''), 162.4 (dd, J=244.2, 15.4 Hz, 2C, C-3'', C-5''), 168.3 (C-1'), 172.0 (*C*O), 179.7 (*C*S).

HRMS (ESI) $C_{20}H_{22}N_3O_3F_2S$ $[M+H]^+$: calculated: 422.1344, measured: 422.1334, deviation: 3.8 ppm, 1.60 mDa, DBE: 10.5.

Methyl (2*S*)-3-methyl 2-[(2-{[(2,6-difluorophenyl)carbamothioyl]amino}benzoyl)amino]butanoate (9g)

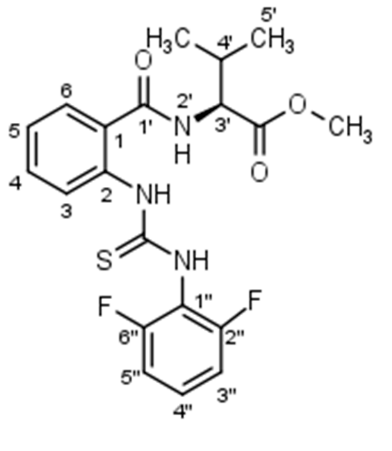

Substance **9** (2.0 mmol, 500 mg) was dissolved in acetone (5.0 ml), then 2,6-difluorophenyl isothiocyanate (2.2 mmol, 378 mg, 285 µl) was added and stirred at room temperature for 24 hours. The solvent was evaporated; the evaporation residue was treated with diethyl-ether (2.0

ml) and hexane (15.0 ml). Yellow precipitate (773 mg, 91.7%) was formed and 200 mg was purified on a chromatographic column with hexane:EtOAc 2:1 resulting in 33 mg white crystal after evaporation.

Mp.: 138.6–139.9°C. R_f (hexane:EtOAc 3:2): 0.44

IR (ZnSe) ν_{max}: 3156, 2962, 1739, 1656, 1530, 1200, 1008, 780 cm^{-1};

δH NMR (400 MHz, DMSO-d_6): 0.94 (d, *J* = 6.8 Hz, 3H, H-5'), 0.98 (d, *J* = 6.9 Hz, 3H, CH_3), 2.11 – 2.23 (m, 1H, H-4'), 3.67 (s, 3H, OCH_3), 4.27 (t, *J* = 7.4 Hz, 1H, H-3'), 7.17 (t, *J* = 8.3 Hz, 2H, H-3'', H-5''), 7.24 (t, *J* = 7.6 Hz, 1H, C-5), 7.42 (p, *J* = 6.6 Hz, 1H, H-4''), 7.50 (t, *J* = 7.7 Hz, 1H, H-4), 7.67 (d, *J* = 7.7 Hz, 1H, H-6), 8.25 (d, *J* = 8.2 Hz, 1H, H-3), 8.85 (d, *J* = 7.7 Hz, 1H, H-2'), 10.02 (s, 1H, NH, N*H*C-2), 10.61 (s, 1H, NH-N*H*C-1'').

δC NMR (101 MHz, DMSO-d_6): 18.8 (CH_3), 19.0 (C-5'), 29.5 (C-4'), 51.7 (OCH_3), 58.5 (C-3'), 112.2 (d, *J* = 22.2 Hz, 2C, C-3'', C-5''), 124.2 (C-5), 125.3 (C-3), 125.9 (C-1), 129.0 (d, *J* = 52.7 Hz, C-6), 129.4 (t, *J* = 11.3 Hz, C-4''), 130.9 (C-4), 138.6 (C-2), 158.8 (dd, *J* = 249.6, 4.3 Hz, 2C, C-2'', C-6''), 168.6 (C-1'), 171.9 (*C*O), 181.6 (*C*S).

C-1" could not be identified due to the low signal-to-noise ratio in the ^{13}C NMR spectrum.

HRMS (ESI) $C_{20}H_{22}N_3O_3F_2S$ $[M+H]^+$: calculated: 422.1344, measured: 422.1333, deviation: 4.0 ppm, 1.70 mDa, DBE: 10.5.

Methyl (2*S*)-4-methyl 2-({2-[(phenylcarbamothioyl)amino]benzoyl}amino)pentanoate (10a)

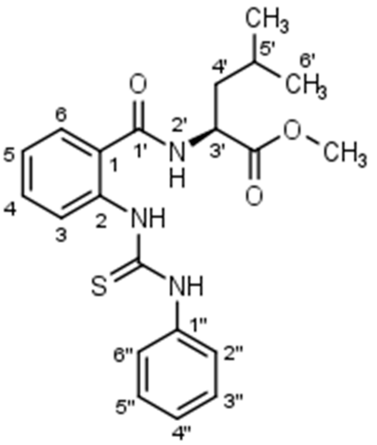

Substance **10** (1.0 mmol, 265 mg) was dissolved in dimethylformamide (DMF) (5.0 ml), then phenyl isothiocyanate (1.1 mmol, 153 mg, 135μl) was added and stirred at room temperature for 22 hours. The solvent was evaporated with toluene and the evaporation residue was treated with diethyl-ether (2.0 ml) and hexane (15.0 ml). A yellow precipitate (290 mg, 72.6%) was formed, 200 was filtered and recrystallized from 1.2 ml MeOH, filtered, washed with hexane, dried at room temperature yielding 99 mg white crystals.

Mp.: 133.5–136.9°C. R_f (hexane:EtOAc 3:2): 0.43

IR (ZnSe) ν_{max}: 3363, 3192, 2960, 1740, 1520, 1356, 693 cm^{-1};

δH (400 MHz, DMSO-d_6): 0.86 (d, *J*=6.4 Hz, 3H, H-6’), 0.90 (d, *J*=6.4 Hz, 3H, H-6’), 1.55 (m, 1H, H-4’), 1.69 (m, 1H, H-5’), 1.74 (m, 1H, H-4’), 3.64 (s, 3H, OCH_3), 4.44 (m, 1H, H-3’), 7.17 (t, *J*=7.1 Hz, 1H, H-4’’) 7.22 (t, *J*=7.7 Hz, 1H, H-5), 7.35 (t, *J*=7.1 Hz, 2H, H-3’’, H-5’’), 7.54-7.42 (m, 3H, H-4, H-2’’, H-6’’), 7.64 (dm, *J*=7.7 Hz, 1H, H-6), 8.15 (d, *J*=7.7 Hz, 1H, H-3), 8.95 (d, *J*=7.5 Hz 1H, H-2’), 10.43 (s, 1H, N*H*C-1’’), 10.48 (s, 1H, N*H*C-2);

δC (100 MHz, DMSO-d_6): 21.2 (C-6’), 22.9 (C-6’), 24.4 (C-5’), 39.5 (C-4’), 50.8 (C-3’), 52.0 (OCH_3), 123.8 (C-5, C-2’’, C-6’’), 125.0 (C-4’’), 125.8 (C-1), 125.9 (C-3), 128.1 (C-6), 128.7

(C-3'', C-5''), 130.4 (C-4), 138.6 (C-2), 138.7 (C-1''), 168.1 (C-1'), 172.7 (*C*=O(OCH3)), 179.2 (*C*S).

HRMS (ESI) $C_{21}H_{25}N_3NaO_3S$ $[M+H]^+$: calculated: 422.1509, measured: 422.1525, deviation: 3.9 ppm, 1.66 mDa, DBE: 11.0.

Methyl (2*S*)-4-methyl 2-[(2-{[(4-nitrophenyl)carbamothioyl]amino}benzoyl)amino]pentanoate (10b)

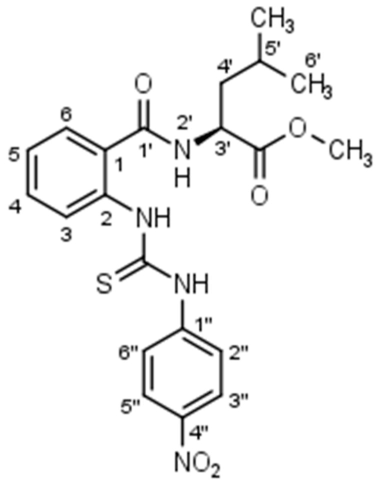

Substance **10** (1.0 mmol, 264 mg) was dissolved in acetone (5.0 ml), then nitrophenyl isothiocyanate (1.1 mmol, 200 mg) was added and stirred at room temperature for 5 hours. The solvent was evaporated; the evaporation residue was treated with diethyl-ether (2.0 ml) and hexane (15.0 ml). Yellow precipitate (354 mg, 79.7%) was formed which was filtered and 200 mg was recrystallized from 2.2 ml MeOH, filtered, washed with hexane, dried at room temperature yielding 132 mg yellow crystals.

Mp.: 144.9–146.7°C. R_f (hexane:EtOAc 3:2): 0.48

IR (ZnSe) ν_{max}: 3325, 2920, 1742, 1624, 1596, 1529, 1502, 1196, 746 cm^{-1};

δH (400 MHz, $CDCl_3$): 0.90-0.80 (m, 6H, H-6'), 1.69-1.54 (m, 3H, H-4', H-5'), 3.67 (s, 3H, OC*H*3), 4.58 (m, 1H, H-3'), 7.14 (d, *J*=7.9 Hz, 1H, H-2'), 7.19 (t, *J*=7.7 Hz 1H, H-5), 7.44 (t, *J*=7.7 Hz, 1H, H-4), 7.52 (d, *J*=7.7 Hz, 1H, H-6), 7.65 (d, *J*=8.9 Hz, 2H, H-2'', H-6''), 8.06 (d,

J=7.7 Hz, 1H, H-3), 8.15 (d, *J*=8.9 Hz, 1H, H-3'', H-5''), 9.30 (s, 1H, N*H*C-1''), 10.37 (s, 1H, N*H*C-2);

δC (100 MHz, $CDCl_3$): 21.6 (C-6'), 22.6 (C-6'), 24.8 (C-5'), 40.8 (C-4'), 51.3 (C-3'), 52.5 (O*C*H_3), 122.3 (C-2'', C-6''), 124.7 (C-3'', C-5''), 125.7 (C-5), 126.4 (C-3), 127.0 (C-1), 127.3 (C-6), 131.5 (C-4), 137.4 (C-2), 143.9 (C-1'')143.9 (C-4''), 168.7 (C-1'), 173.0 (*C*=O(OCH_3)), 179.5 (*C*S).

HRMS (ESI) $C_{21}H_{2}4N_4NaO_5S$ $[M+H]^+$: calculated: 467.1360, measured: 467.1385, deviation: 5.4 ppm, 2.53 mDa, DBE: 12.0.

Methyl (2*S*)-4-methyl 2-[(2-{[(3,5-bistrifluoromethylphenyl)carbamothioyl]amino}-benzoyl)amino]pentanoate (10c)

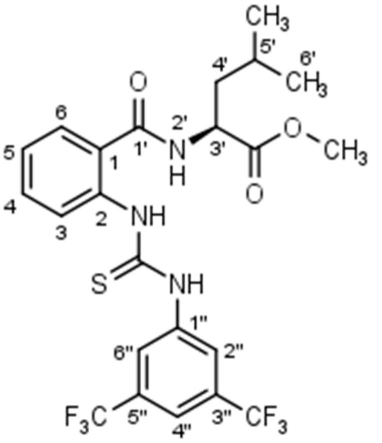

Substance **10** (1.0 mmol, 265 mg) was dissolved in acetone (5.0 ml), then 3,5-bis(trifluoromethyl)phenyl isothiocyanate (1.1 mmol, 297 mg, 380 µl) was added and stirred at room temperature for 2 hours. The solvent was evaporated, white precipitate (338 mg, 63.1%) was formed and 205 mg was recrystallized from 1.5 ml MeOH, filtered, washed with hexane, dried at room temperature yielding 99 mg white crystals.

Mp.: 137.2–140.4°C. R_f (hexane:EtOAc 3:2): 0.57

IR (ZnSe) ν_{max}: 3622, 3262, 2964, 1713, 1622, 1525, 1123, 880 cm^{-1};

δH (400 MHz, $CDCl_3$): 0.92-0.84 (m, 6H, H-6'), 1.71-1.55 (m, 3H, C-4', C-5'), 3.70 (s, 3H, O*C*H_3), 4.62 (m, 1H, H-3'), 7.03 (d, *J*=7.8 Hz, 1H, H-2'), 7.27 (m, 1H, H-5), 7.50 (m, 1H, H-4), 7.53 (m, 1H, H-6), 7.68 (m, 1H, H-4''), 7.99 (m, 2H, H-2'', H-6''), 8.10 (d, *J*=8.1 Hz, 1H, H-3), 8.98 (s, 1H, N*H*C-1''), 10.21 (s, 1H, N*H*C-2);

δC (100 MHz, $CDCl_3$): 21.6 (C-6'), 22.6 (C-6'), 24.9 (C-5'), 41.0 (C-4'), 51.4 (C-3'), 52.5 (O*C*H_3), 119.1 (m, C-4''), 123.0 (q, *J*=272.6 Hz, *C*F_3), 124.2 (m, C-2'', C-6''), 126.1 (C-5), 126.7 (C-3), 127.3 (C-6), 127.8 (C-1), 131.8 (C-4), 132.1 (q, *J*=33.6 Hz, C-3'', C-5''), 137.4 (C-2), 139.5 (C-1''), 169.0 (C-1'), 173.1 (*C*=O(OCH_3)), 180.6 (*C*S).

HRMS (ESI) $C_{23}H_{23}F_6N_3NaO_3S$ $[M+H]^+$: calculated: 558.1257, measured: 558.1286, deviation: 5.3 ppm, 2.99 mDa, DBE: 11.0.

Methyl (2*S*)-4-methyl 2-[(2-{[(4-methoxyphenyl)carbamothioyl]amino}benzoyl)amino]pentanoate (10d)

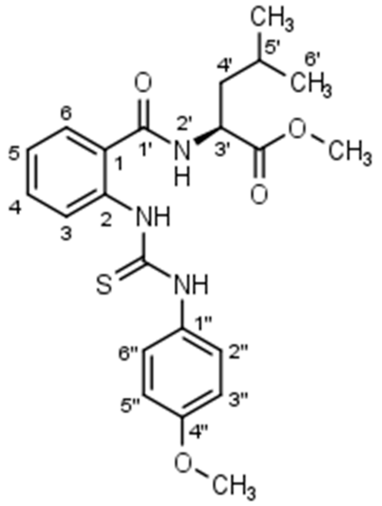

Substance **10** (1.0 mmol, 264 mg) was dissolved in DMF (5.0 ml), then 4-methoxyphenyl isothiocyanate (1.1 mmol, 173 mg, 145 μl) was added and stirred at room temperature for 82 hours. The solvent was evaporated with toluene; the evaporation residue was treated with diethyl-ether (2.0 ml) and hexane (15.0 ml). Yellow precipitate (316 mg, 73.6%) was formed, 206 mg was recrystallized from ethanol EtOH and 20 drops of hexane were added, solid was

filtered, washed with hexane and dried at room temperature resulting in 85 mg yellowish crystals.

Mp.: 101.3–104.9°C. R_f (hexane:EtOAc 3:2): 0.42

IR (ZnSe) ν_{max}: 3362, 2956, 1750, 1586, 1508, 1249, 1159, 821 cm^{-1};

δH (500 MHz, DMSO-d_6): 0.87 (d, 3H, H-6'), 0.91 (d, 3H, H-6'), 1.55 (m, 1H, H-4'), 1.67 (m, 1H, H-5'), 1.74 (m, 1H, H-4'), 3.65 (s, 3H, COOCH_3), 3.75 (s, 3H, 4''-COCH_3), 4.42 (m, 1H, H-3'), 6.92 (m, 2H, H-3'', H-5''), 7.20 (m, 1H, H-5), 7.30 (m, 2H, H-2'', H-6''), 7.47 (m, 1H, H-4,), 7.62 (dm, *J*=8.0 Hz, 1H, H-6), 8.22 (d, *J*=8.0 Hz, 1H, H-3), 8.93 (d, *J*=7.6 Hz, 1H, H-2'), 10.26 (s, 1H, N*H*C-1''), 10.34 (s, 1H, N*H*C-2);

δC (125 MHz, DMSO-d_6): 21.2 (C-6'), 22.9 (CH_3), 24.4 (C-5'), 39.2 (C-4'), 50.8 (C-3'), 52.0 (COOCH_3), 55.2 (4''-OCH_3), 114.0 (C-3'', C-5''), 123.5 (C-5), 125.3 (C-1), 125.5 (C-3), 126.1 (C-2'', C-6''), 128.0 (C-6), 130.4 (C-4), 131.2 (C-1''), 138.8 (C-2), 156.9 (C-4''), 168.1 (C-1'), 172.7 (*C*=O(OCH_3)), 179.3 (*C*S).

HRMS (ESI) $C_{22}H_{28}N_3O_4S$ $[M+H]^+$: calculated: 430.1795, measured: 430.1810, deviation: 3.4 ppm, 1.47 mDa, DBE: 11.0.

Methyl (2*S*)-4-methyl 2-[(2-{[(4-fluorophenyl)carbamothioyl]amino}benzoyl)amino]pentanoate (10e)

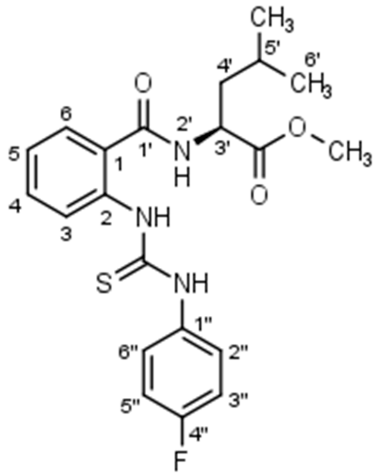

Substance **10** (1.0 mmol, 265 mg) was dissolved in acetone (5.0 ml), then 4-fluorophenyl isothiocyanate (1.1 mmol, 169 mg) was added and stirred at room temperature for 100 hours. The solvent was evaporated; the evaporation residue was treated with diethyl-ether (2.0 ml) and hexane (15.0 ml). Yellow precipitate (224 mg, 53.7%) was formed, 201 mg was recrystallized from 1.0 ml MeOH, filtered, washed with hexane, dried at room temperature yielding 68 mg white crystals.

Mp.: 128.5–131.1°C. R_f (hexane:EtOAc 3:2): 0.48

IR (ZnSe) ν_{max}: 3673, 3339, 1786, 1742, 1592, 1504, 1077, 745 cm^{-1};

δH NMR (400 MHz, DMSO-d_6): 0.87 (d, J = 6.2 Hz, 3H, H-6’), 0.91 (d, J = 6.2 Hz, 3H, CH_3), 1.46 – 1.62 (m, 1H, H-5’), 1.62 – 1.84 (m, 2H, H-4’), 3.65 (s, 3H, OCH_3), 4.37 – 4.53 (m, 1H, H-3’), 7.13 – 7.27 (m, 3H, H-5, H-3’’, H-5’’), 7.48 (t, J = 7.0 Hz, 3H, H-4, H-2’’, H-6’’), 7.65 (d, J = 7.8 Hz, 1H, H-6), 8.18 (d, 1H, H-3), 8.93 (d, J = 7.6 Hz, 1H, H-2’), 10.43 (s, 1H, NH, N*H*C-2), 10.44 (s, 1H, NH, N*H*C-1’’).

δC NMR (101 MHz, DMSO-d_6): 21.2 (C-6’), 22.8 (CH$_3$), 24.4 (C-5’), 39.3 (C-4’), 50.8 (C-3’), 52.0 (OCH$_3$), 115.3 (d, J = 22.4 Hz, 2C, C-3’’, C-5’’), 123.8 (C-5), 125.6 (C-3), 125.7 (C-1),

126.2 (d, J = 8.2 Hz, 2C, C-2'', C-6''), 130.5 (C-4), 135.0 (C-1''), 138.6 (C-2), 159.4 (d, J = 242.1 Hz, C-4''), 168.1 (C-1'), 172.6 (*C*O), 179.5 (*C*S).

HRMS (ESI) $C_{21}H_{25}N_3O_3FS$ $[M+H]^+$: calculated: 418.1595, measured: 418.1585, deviation: 3.7 ppm, 1.57 mDa, DBE: 10.5.

Methyl (2*S*)-4-methyl 2-[(2-{[(3,5-difluorophenyl)carbamothioyl]amino}benzoyl)amino]pentanoate (10f)

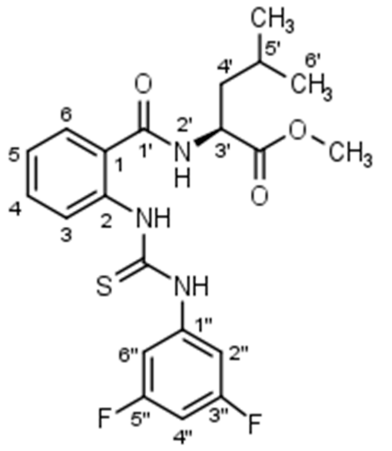

Substance **10** (1.0 mmol, 265 mg) was dissolved in acetone (5.0 ml), then 3,5-difluorophenyl isothiocyanate (1.0 mmol, 176 mg, 140 µl) was added and stirred at room temperature for 9 hours. The solvent was evaporated; the evaporation residue was treated with diethyl-ether (2.0 ml) and hexane (15.0 ml). White precipitate (333 mg, 76.5%) was formed, 200 mg was recrystallized from 1.6 ml MeOH, filtered, washed with hexane, dried at room temperature yielding 130 mg low density white crystals.

Mp.: 149.2–150.9°C. R_f (hexane:EtOAc 3:2): 0.60

IR (ZnSe) ν_{max}: 3407, 3322, 2958, 1740, 1524, 1297, 727 cm^{-1};

δH NMR (400 MHz, DMSO-d_6): 0.89 (d, J = 6.3 Hz, 3H, H-6'), 0.92 (d, J = 6.3 Hz, 3H, CH_3), 1.51 – 1.63 (m, 1H, H-5'), 1.63 – 1.84 (m, 2H, H-4'), 3.66 (s, 3H, OCH_3), 4.32 – 4.55 (m, 1H, H-3'), 7.17 (t, J = 8.3 Hz, 2H, H-3'', H-5''), 7.23 (t, J = 7.5 Hz, 1H, H-5), 7.41 (q, J = 7.0 Hz,

1H, H-4''), 7.50 (t, $J = 7.9$ Hz, 1H, H-4), 7.68 (d, $J = 7.8$ Hz, 1H, H-6), 8.37 (d, $J = 8.3$ Hz, 1H, H-3), 9.00 (d, $J = 7.5$ Hz, 1H, H-2'), 10.06 (s, 1H, NH, N*H*C-2), 10.83 (s, 1H, NH, N*H*C-1'').

δC NMR (101 MHz, DMSO-d_6): 21.2 (C-6') 22.9 (*C*H_3). 24.5 (C-5'), 39.2 (C-4'), 50.9 (C-3'), 52.0 (O*C*H_3), 99.4 (t, J=26.2 Hz, C-4''), 105.6 (m, C-2'',C-6''), 123.8 (C-5), 124.6 (C-3'), 128.2 (C-6), 130.9 (C-4), 138.8 (C-2), 141.8 (t, J=13.3 Hz, C-1''), 158.4, 162.1 (dd, J=243.5, 15.2 Hz, 2C, C-3'', C-5''), 168.2 (C-1'), 172.6 (*C*O), 181.2 (*C*S).

HRMS (ESI) $C_{21}H_{24}N_3O_3F_2S$ $[M+H]^+$: calculated: 436.15, measured: 436.1489, deviation: -4.0 ppm, -1.74 mDa, DBE: 10.5.

Methyl (2*S*)-4-methyl 2-[(2-{[(2,6-difluorophenyl)carbamothioyl]amino}benzoyl)amino]pentanoate (10g)

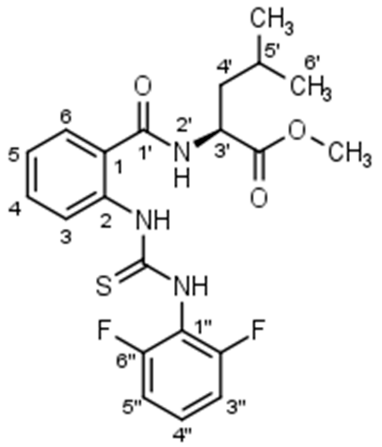

Substance **10** (1.0 mmol, 265 mg) was dissolved in acetone (5.0 ml), then 3,5-difluorophenyl isothiocyanate (1.0 mmol, 179 mg, 135 µl) was added and stirred at room temperature for 20 hours. The solvent was evaporated; the evaporation residue was treated with diethyl-ether (2.0 ml) and hexane (15.0 ml). A yellow precipitate (285 mg, 65.4%) was formed, 200 mg was recrystallized from 1.5 ml MeOH, filtered, washed with hexane, dried at room temperature yielding 102 mg white crystals.

Mp.: 147.9–150.0°C. R_f (hexane:EtOAc 3:2): 0.44

IR (ZnSe) ν_{max}: 331, 2958, 1744, 1593, 1434, 1183, 1007, 748 cm^{-1};

δH NMR (400 MHz, DMSO-d_6): 0.89 (d, J = 6.3 Hz, 3H, H-6'), 0.92 (d, J = 6.3 Hz, 3H, CH_3), 1.51 – 1.63 (m, 1H, H-5'), 1.63 – 1.84 (m, 2H, H-4'), 3.66 (s, 3H, OCH_3), 4.32 – 4.55 (m, 1H, H-3'), 7.17 (t, J = 8.3 Hz, 2H, H-3''=H-5''), 7.23 (t, J = 7.5 Hz, 1H, H-5), 7.41 (q, J = 7.0 Hz, 1H, H-4''), 7.50 (t, J = 7.9 Hz, 1H, H-4), 7.68 (d, J = 7.8 Hz, 1H, H-6), 8.37 (d, J = 8.3 Hz, 1H, H-3), 9.00 (d, J = 7.5 Hz, 1H, H-2'), 10.06 (s, 1H, NH, N*H*C-2), 10.83 (s, 1H, NH, N*H*C-1'').

δC NMR (101 MHz, DMSO-d_6): 21.2 (C-6') 22.9 (CH$_3$). 24.5 (C-5'), 39.2 (C-4'), 50.9 (C-3'), 52.0 (OCH$_3$), 112.0 (d, J=21.5 Hz,C-3'', C-5'') 115.7 (m, C-1''), 123.8 (C-5), 124.6 (C-3'), 128.2 (C-6), 129.2 (t, J = 9.1 Hz, C-4''), 130.9 (C-4), 138.8 (C-2), 158.4 (dd, J = 249.6, 4.8 Hz, 2C, C-2'', C-6''), 168.2 (C-1'), 172.6 (*C*O), 181.2 (*C*S)

HRMS (ESI) $C_{21}H_{24}N_3O_3F_2S$ $[M+H]^+$: calculated: 436.15, measured: 436.1488, deviation: 4.2 ppm, 1.85 mDa, DBE: 10.5.

Methyl (2*S*)-3-methyl 2-({2-[(phenylcarbamothioyl)amino]benzoyl}amino)pentanoate (11a)

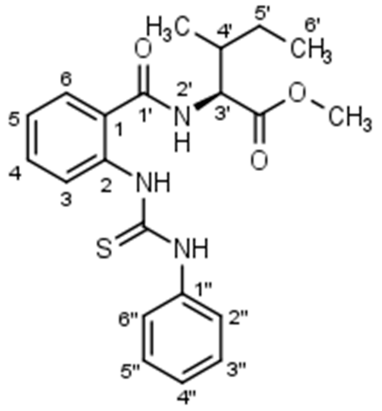

Substance **11** (1.0 mmol, 265 mg) was dissolved in dimethylformamide (DMF) (5.0 ml), then phenyl isothiocyanate (1.0 mmol, 141 mg, 125µl) was added and stirred at room temperature for 9 hours. The solvent was evaporated with toluene; the evaporation residue was treated with diethyl-ether (2.0 ml) and hexane (15.0 ml). Yellow precipitate (214 mg, 53.5%) was formed, 213 was filtered and recrystallized from 1.6 ml MeOH, filtered, washed with hexane, dried at room temperature yielding 120 mg white crystals.

Mp.: 134.5–138.0°C. R_f (hexane:EtOAc 3:2): 0.43

IR (ZnSe) ν_{max}: 3347, 2954, 1738, 1430, 1351, 1179, 753 cm^{-1};

δH (400 MHz, DMSO-d_6): 0.84 (d, J=7.4 Hz, 3H, H-6'), 0.89 (d, J=6.9 Hz, 3H, CH_3), 1.26 (m, 1H, H-5'), 1.47 (m, 1H, H-5') 1.92, (m, 1H, H-4'), 3.64 (s, 3H, OCH_3), 4.32 (t, J=7.3 Hz, 1H, H-3'), 7.17 (t, J=7.6 Hz, 1H, H-4'') 7.23 (t, J=7.4 Hz, 1H, H-5), 7.35 (t, J=7.6 Hz, 2H, H-3'', H-5''), 7.52-7.44 (m, 3H, H-4, H-2'', H-6''), 7.62 (dm, J=7.4Hz, 1H, H-6), 8.04 (d, J=8.2 Hz, H-3), 8.78 (d, J=7.3 Hz 1H, H-2'), 10.24 (s, 1H, N*H*C-2), 10.43 (s, 1H, N*H*C-1'');

δC (100 MHz, DMSO-d_6): 11.0 (C-6'), 15.5 (CH_3), 25.1 (C-5'), 35.8 (C-4'), 51.7 (OCH_3), 57.2 (C-3'), 123.8 (C-2'', C-6''), 124.1 (C-5), 125.0 (C-4''), 126.4 (C-3), 126.9 (C-1), 128.5 (C-6), 128.7 (C-3'', C-5''), 130.4 (C-4), 138.3 (C-2), 138.7 (C-1''), 168.1 (C-1'), 171.8 (*C*=O(OCH_3)), 179.4 (*C*S).

HRMS (ESI) $C_{21}H_{25}N_3NaO_3S$ $[M+H]^+$: calculated: 422.1509, measured: 422.1525, deviation: 3.9 ppm, 1.65 mDa, DBE: 11.0.

Methyl (2*S*)-3-methyl 2-[(2-{[(4-nitrophenyl)carbamothioyl]amino}benzoyl)amino]pentanoate (11b)

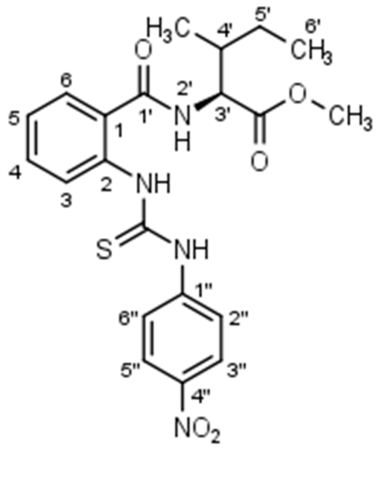

Substance **11** (1.0 mmol, 265 mg) was dissolved in acetone (5.0 ml), then 4-nitrophenyl isothiocyanate (1.1 mmol, 200 mg) was added and stirred at room temperature for 6 hours. The

solvent was evaporated; the evaporation residue was treated with diethyl-ether (2.0 ml) and hexane (15.0 ml). Yellow precipitate (363 mg, 81.6%) was formed which was filtered and 202 mg was recrystallized from 1.4 ml MeOH, filtered, washed with hexane, and dried at room temperature yielding 75 mg yellow crystals.

Mp.: 137.4–141.5°C. R_f (hexane:EtOAc 3:2): 0.41

IR (ZnSe) ν_{max}: 2962, 2875, 1642, 1504, 1327, 1110, 750 cm^{-1};

δH (400 MHz, $CDCl_3$): 0.89 (t, *J*=7.4 Hz, 3H, H-6'), 0.91 (d, *J*=6.8 Hz, 3H, C*H*3), 1.22 (m, 1H, H-5'), 1.44 (m, 1H, H-5'), 1.94 (m, 1H, H-4'), 3.74 (s, 3H, OC*H*3), 4.60 (dd, *J*=8.4, 4.9 Hz, 1H, H-3'), 6.88 (d, *J*=8.4, 1H, H-2'), 7.26 (m, 1H, H-5), 7.52 (m, 1H, H-4), 7.55 (m, 1H, H-6), 7.63 (d, *J*=9.1 Hz 2H, H-2'', H-6''), 8.23 (d, *J*=9.1 Hz, 1H, H-3'', H-5''), 8.32 (d, *J*=8.1 Hz, 1H, H-3), 8.70 (s, 1H, N*H*C-1''), 10.70 (s, 1H, N*H*C-2);

δC (100 MHz, $CDCl_3$): 11.5 (C-6'), 15.5 (*C*H3), 25.2 (C-5'), 37.8 (C-4'), 52.4 (O*C*H3), 57.0 (C-3'), 122.5 (C-2'', C-6''), 125.0 (C-3'', C-5''), 125.5 (C-5), 125.8 (C-3), 125.8 (C-1), 127.1 (C-6), 131.8 (C-4), 138.0 (C-2), 143.5 (C-1''), 144.3 (C-4''), 168.5 (C-1'), 172.0 (*C*=O(OCH_3)), 179.2 (*C*S).

HRMS (ESI) $C_{21}H_{24}N_4NaO_5S$ $[M+H]^+$: calculated: 467.1360, measured: 467.1379, deviation: 4.1 ppm, 1.94 mDa, DBE: 12.0.

Methyl (2*S*)-3-methyl 2-[(2-{[(3,5-bistetrifluoromethylphenyl)carbamothioyl]amino} benzoyl)amino]pentanoate (11c)

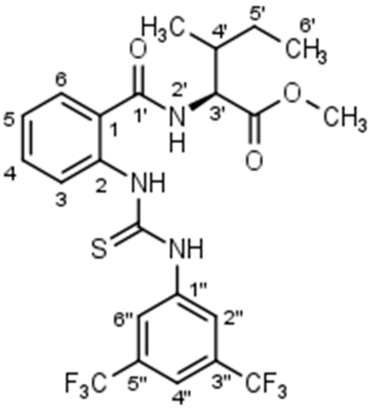

Substance **11** (1.0 mmol, 268 mg) was dissolved in acetone (5.0 ml), then 3,5-bis(trifluoromethyl)phenyl isothiocyanate (1.1 mmol, 301 mg, 385 µl) was added and stirred at room temperature for 4 hours. The solvent was evaporated; the evaporation residue was treated with diethyl-ether (2.0 ml) and hexane (15.0 ml). White precipitate (483 mg, 90.2%) was formed which was filtered and 200 mg was recrystallized from 2.0 ml MeOH, filtered, washed with hexane, dried at room temperature yielding 130 mg white crystals.

Mp.: 142.5–144.8°C. R_f (hexane:EtOAc 3:2): 0.56

IR (ZnSe) ν_{max}: 3294, 1726, 1622, 1524, 1271, 1123, 880 cm^{-1};

δH (400 MHz, $CDCl_3$): 0.85 (t, *J*=7.5 Hz, 3H, H-6’), 0.89 (d, *J*=6.9 Hz, 3H, C*H*3), 1.20 (m, 1H, C-5’), 1.40 (m, 1H, C-5’), 1.93 (m, 1H, C-4’), 3.71 (s, 3H, OC*H*3), 4.59 (dd, *J*=8.1, 4.8 Hz, 1H, H-3’), 7.03 (d, *J*=8.1 Hz, 1H, H-2’), 7.26 (t, *J*=7.9 Hz, 1H, H-5), 7.49 (m, 1H, H-4), 7.53 (m, 1H, H-6), 7.68 (m, 1H, H-4’’), 7.98 (m, 2H, H-2’’, H-6’’), 8.10 (d, *J*=7.9 Hz 1H, H-3), 8.95 (brs, 1H, N*H*C-1’’), 10.26 (brs, 1H, N*H*C-2);

δC (100 MHz, $CDCl_3$): 11.4 (C-6’), 15.5 (*C*H3), 25.2 (C-5’), 37.7 (C-4’), 52.3 (O*C*H3), 57.2 (C-3’), 119.1 (m, C-4’’), 123.1 (*J*=271.8 Hz, *C*F3), 124.3 (m, C-2’’, C-6’’), 126.0 (C-5), 126.6

(C-3), 127.4 (C-6), 127.7 (C-1), 131.7 (C-4), 132.1 (*J*=33.9 Hz, C-3'', C-5''), 137.4 (C-2), 139.4 (C-1''), 168.9 (C-1'), 172.0 (*C*=O(OCH$_3$)), 180.5 (*C*S).

HRMS (ESI) $C_{23}H_{23}F_6N_3NaO_3S$ $[M+H]^+$: calculated: 558.1257, measured: 558.1276, deviation: 3.4 ppm, 1.91 mDa, DBE: 11.0.

Methyl (2*S*)-3-methyl 2-[(2-{[(4-methoxyphenyl)carbamothioyl]amino}benzoyl)amino]pentanoate (11d)

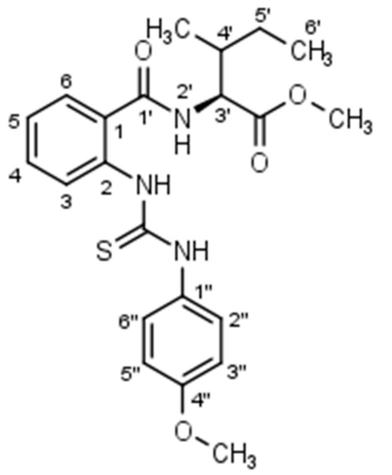

Substance **11** (1.0 mmol, 265 mg) was dissolved in DMF (5.0 ml), then 4-methoxyphenyl isothiocyanate (1.1 mmol, 185 mg, 155 μl) was added and stirred at room temperature for 76 hours. The solvent was evaporated with toluene; the evaporation residue was treated with diethyl-ether (2.0 ml) and hexane (15.0 ml). Yellow precipitate (370 mg, 73.6%) was formed, 201 mg was recrystallized from 2.2 ml MeOH, solid was filtered, washed with hexane and dried at room temperature resulting in 119 mg yellowish crystals.

Mp.: 137.5–141.2°C. R_f (hexane:EtOAc 3:2): 0.32

IR (ZnSe) ν_{max}: 3154, 2965, 1736, 1589, 1503, 1351, 1238, 746 cm^{-1};

δH (500 MHz, DMSO-*d*$_6$): 0.85 (t, *J*=7.5 Hz, 3H, H-6'), 0.89 (d, *J*=6.8 Hz, 3H, C*H*$_3$), 1.26 (m, 1H, H-5'), 1.47 (m, 1H, H-5'), 1.92, (m, 1H, H-4'), 3.65 (s, 3H, COOC*H*$_3$), 3.75 (s, 3H, 4''-COC*H*$_3$), 4.30 (t, *J*=7.1 Hz, 1H, H-3'), 6.92 (m, 2H, H-3'', H-5''), 7.21 (m, 1H, H-5), 7.31 (m,

2H, H-2’’, H-6’’), 7.46 (m, 1H, H-4,), 7.60 (dm, *J*=7.7 Hz, 1H, H-6), 8.10 (dm, *J*=7.7 Hz, 1H, H-3), 8.79 (d, *J*=7.1 Hz, 1H, H-2’);

δC (125 MHz, DMSO-*d*6): 11.0 (C-6’), 15.5 (*C*H3), 25.1 (C-5’), 35.8 (C-4’), 51.7 (O*C*H3), 55.2 (4’’-O*C*H3), 57.2 (C-3’), 114.0 (C-3’’, C-5’’), 123.9 (C-5), 126.1 (C-3, C-2’’, C-6’’), 126.5 (C-1), 128.4 (C-6), 130.3 (C-4), 131.2 (C-1’’), 138.5 (C-2), 156.9 (C-4’’), 168.1 (C-1’), 171.8 (*C*=O(CH3)), 179.5 (*C*S).

HRMS (ESI) $C_{22}H_{28}N_3O_4S$ [M+H]$^+$: calculated: 430.1795, measured: 430.1806, deviation: 2.5 ppm, 1.09 mDa, DBE: 11.0.

Methyl (2*S*)-3-methyl 2-[(2-{[(4-fluorophenyl)carbamothioyl]amino}benzoyl)amino]pentanoate (11e)

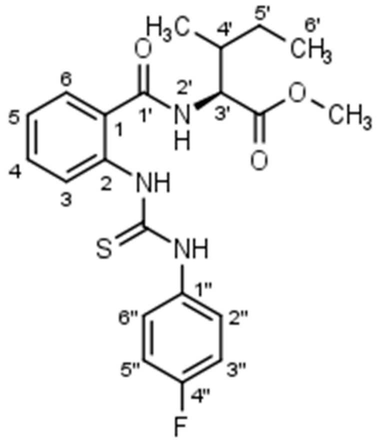

Substance **11** (2.0 mmol, 529 mg) was dissolved in DMF (5.0 ml), then 4-fluorophenyl isothiocyanate (2.2 mmol, 336 mg) was added and stirred at room temperature for 24 hours. The solvent was evaporated with toluene; the evaporation residue was treated with diethyl-ether (2.0 ml) and hexane (15.0 ml). Yellow precipitate (300 mg, 35.9%) was formed, 250 mg was purified on a chromatographic column with hexane:EtOAc 2:1. After solvent evaporation 103 mg white crystals were yielded.

Mp.: 101.6–106.3°C. R_f (hexane:EtOAc 3:2): 0.44

IR (ZnSe) ν_{max}: 3168, 2968, 1739, 1591, 1514, 1211, 741 cm^{-1};

δH NMR (400 MHz, DMSO-d_6): 0.85 (t, J = 7.4 Hz, 3H, H-6'), 0.89 (d, J = 6.8 Hz, 3H, CH_3), 1.16 – 1.54 (m, 2H, H-5'), 1.83 – 1.99 (m, 1H, H-4'), 3.65 (s, 3H, OCH_3), 4.33 (t, J = 7.3 Hz, 1H, H-3'), 7.19 (t, J = 8.9 Hz, 2H, H-3'', H-5''), 7.23 (t, J = 7.3 Hz, 1H, H-5), 7.48 (td, J = 7.2, 3.5 Hz, 3H, H-4, H-2'', H-6''), 7.62 (dd, J = 7.8, 1.6 Hz, 1H, H-6), 8.05 (d, J = 8.2 Hz, 1H, H-3), 8.76 (d, J = 7.6 Hz, 1H, H-2'), 10.25 (s, 1H, NHC-2), 10.38 (s, 1H, NHC-1'').

δC NMR (101 MHz, DMSO-d_6): 11.0 (C-6'), 15.5 (CH3), 25.1 (C-5'), 35.9 (C-4'), 51.8 (OCH3), 57.2 (C-3'), 115.5 (d, J = 22.4 Hz, 2C, C-3''=C-5''), 124.7 (C-5), 126.4 (2C, d, J=8.5 Hz, C-2'', C-6''), 126.7 (C-3), 127.6 (C-1), 128.9 (C-6), 130.9 (C-4), 135.3 (d, J = 2.3 Hz, C-1''), 138.4 (C-2), 159.7 (d, J = 242.5 Hz, C-4''), 168.4 (C-1'), 172.1 (CO), 180.1 (CS).

HRMS (ESI) $C_{21}H_{25}N_3O_3FS$ $[M+H]^+$: calculated: 418.1595, measured: 418.1587, deviation: 3.3 ppm, 1.37 mDa, DBE: 10.5.

Methyl (2*S*)-3-methyl 2-[(2-{[(3,5-difluorophenyl)carbamothioyl]amino}benzoyl)amino]pentanoate (11f)

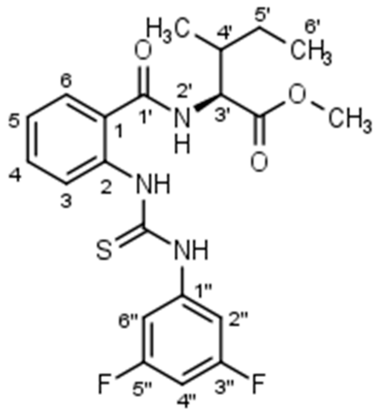

Substance **11** (1.0 mmol, 264 mg) was dissolved in acetone (5.0 ml), then 3,5-difluorophenyl isothiocyanate (1.1 mmol, 183 mg, 145 μl) was added and stirred at room temperature for 3 hours. The solvent was evaporated, meanwhile white precipitate (365 mg, 83.8%) appeared,

and 203 mg was recrystallized from 10.0 ml MeOH, filtered, washed with hexane, dried at room temperature yielding 200 mg low density white crystals.

Mp.: 160.0–166.6°C. R_f (hexane:EtOAc 3:2): 0.59

IR (ZnSe) ν_{max}: 3402, 3324, 3179, 2965, 1734, 1542, 732 cm^{-1};

δH NMR (400 MHz, DMSO-d_6): 0.83 (t, *J* = 7.4 Hz, 3H, H-6'), 0.89 (d, *J* = 6.8 Hz, 3H, C*H*3), 1.17 – 1.31 (m, 2H, H-5'), 1.34 – 1.59 (m, 1H, H-4'), 3.64 (s, 3H, OC*H*3), 4.35 (t, *J* = 7.2 Hz, 1H, H-3'), 6.98 (t, *J* = 9.5 Hz, 1H, H-4''), 7.28 (t, *J* = 7.6 Hz, 1H, H-5), 7.39 (d, *J* = 7.0 Hz, 2H, H-2'', H-6''), 7.50 (t, *J* = 7.8 Hz, 1H, H-4), 7.92 (d, *J* = 8.2 Hz, 1H, H-3), 8.75 (d, *J* = 7.6 Hz, 1H, H-2'), 10.39 (s, 1H, NH, N*H*C-2), 10.69 (s, 1H, NH, N*H*C-1'').

δC NMR (101 MHz, DMSO-d_6): 10.9 (C-6'), 15.4 (*C*H3), 25.1 (C-5'), 35.9 (C-4'), 51.7 (O*C*H3), 57.2 (C-3'), 99.6 (t, *J* = 26.3 Hz, C-4''), 105.7 (d, *J* = 28.6 Hz, 2C, C-2'', C-6''), 125.0 (C-5), 126.9 (C-3), 128.2 (C-1), 128.8 (C-6), 130.7 (C-4), 137.8 (C-2), 142.0 (t, *J* = 13.4 Hz, C-1''), 162.4 (dd, *J* = 244.0, 15.4 Hz, 2C, C-3'', C-5''), 168.2 (C-1'), 172.0 (*C*O), 179.7 (*C*S).

HRMS (ESI) $C_{21}H_{25}N_3O_3F_2S$ $[M+H]^+$: calculated: 436.15, measured: 436.1490, deviation: 3.7 ppm, 1.65 mDa, DBE: 10.5.

Methyl (2*S*)-3-methyl 2-[(2-{[(2,6-difluorophenyl)carbamothioyl]amino}benzoyl)amino]pentanoate (11g)

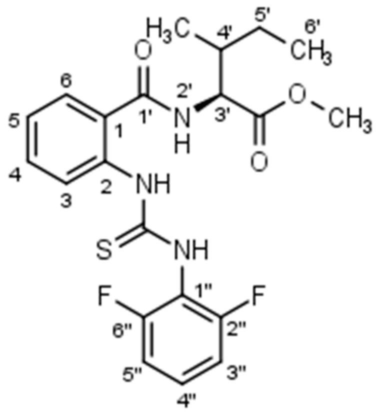

Substance **11** (1.0 mmol, 264 mg) was dissolved in acetone (5.0 ml), then 2,6-difluorophenyl isothiocyanate (1.1 mmol, 192 mg, 145 µl) was added and stirred at room temperature for 22 hours. The solvent was evaporated; the evaporation residue was treated with diethyl-ether (2.0 ml) and hexane (15.0 ml). Yellow precipitate (326 mg, 74.8%) was formed and 205 mg was recrystallized from 1.0 ml MeOH. The solid was filtered, washed with hexane and dried at room temperature resulting in 90 mg white crystals.

Mp.: 138.6–139.9°C. R_f (hexane:EtOAc 3:2): 0.56

IR (ZnSe) ν_{max}: 3725, 3310, 2970, 1739, 1518, 1434, 1007, 760 cm^{-1};

δH NMR (400 MHz, DMSO-d_6): 0.85 (t, J = 7.5 Hz, 3H, H-6'), 0.89 (d, J = 6.8 Hz, 3H, CH_3), 1.08 – 1.68 (m, 2H, H-5'), 1.82 – 2.07 (m, 1H, H-4'), 3.65 (s, 3H, OCH_3), 4.31 (t, J = 7.3 Hz, 1H, H-3'), 7.15 (t, J = 8.3 Hz, 2H, H-3'', H-5''), 7.22 (t, J = 7.5 Hz, 1H, H-5), 7.40 (d, J = 7.3 Hz, 1H, H-4''), 7.48 (t, J = 7.9 Hz, 1H, H-4), 7.64 (d, J = 7.7 Hz, 1H, H-6), 8.24 (d, J = 8.3 Hz, 1H, H-3), 8.84 (d, J = 7.6 Hz, 1H, H-2'), 10.01 (s, 1H, NH, NHC-1''), 10.62 (s, 1H, NH, NHC-2).

δC NMR (101 MHz, DMSO-d_6): 11.1 (C-6'), 15.6 (CH$_3$), 25.4 (C-5'), 35.9 (C-4'), 51.9 (OCH$_3$), 57.5 (C-3'), 112.4 (d, J = 22.2 Hz, 2C, C-3''=C-5''), 124.4 (C-5), 125.4 (C-1), 126.0

(C-3), 128.9 (C-6), 129.5 (t, *J* = 8.2 Hz, C-4’’), 131.1 (C-1), 159.0 (dd, *J* = 249.1, 4.2 Hz, 2C, C-2’’, C-6’’), 168.7 (C-1’), 172.2 (*C*O), 181.8 (*C*S).

C-1” could not be identified due to the low signal-to-noise ratio in the ^{13}C NMR spectrum.

HRMS (ESI) $C_{21}H_{25}N_3O_3F_2S$ $[M+H]^+$: calculated: 436.15, measured: 436.1488, deviation: 4.2 ppm, 1.84 mDa, DBE: 10.5.

***N,N'*-diphenylthiourea (12a)**

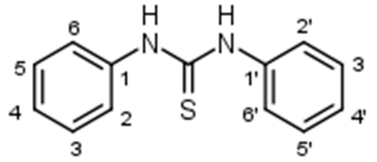

Aniline (1.0 mmol, 94 mg, 92 µl) was added to acetone (5.0 ml), then phenyl isothiocyanate (1.1 mmol, 147 mg, 130 µl) was added and stirred at room temperature for 7 hours. The solvent was evaporated, and the evaporation residue was treated with diethyl-ether (2.0 ml) and hexane (15.0 ml). Yellow precipitate (191 mg, 83.7%) was formed, filtered and recrystallized from 1.5 ml MeOH, filtered, washed with hexane, dried at room temperature yielding 136 mg white crystals.

Mp.: 157.5–159.4°C. R_f (hexane:EtOAc 3:2): 0.63

δH NMR (400 MHz, DMSO-d_6): 7.13 (t, *J* = 7.4 Hz, 2H, H-4=H-4’), 7.33 (t, *J* = 7.9 Hz, 4H, H-3, H-5, H-3’, H-5’), 7.49 (d, *J* = 7.5 Hz, 4H, H-2, H-6, H-2’, H-6’), 9.78 (s, 2H, N*H*).

δC NMR (101 MHz, DMSO-d_6): 124.0 (4C, C-2, C-6, C-2’, C-6’), 124.8 (2C, C-4, C-4’), 128.8 (4C, C-3, C-5, C-3’, C-5’), 139.8 (2C, C-1, C-1’), 179.9 (*C*S).

***N*-(4-nitrophenyl)-*N'*-phenylthiourea (12b)**

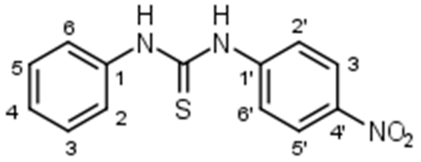

Aniline (1.0 mmol, 94 mg, 92 µl) was added to acetone (5.0 ml), then 4-nitrophenyl isothiocyanate (1.1 mmol, 201 mg) was added and stirred at room temperature for 2 hours. The solvent was evaporated, and the evaporation residue was treated with diethyl-ether (2.0 ml) and hexane (15.0 ml). Yellow precipitate (268 mg, 98.0%) was formed, filtered and 201 mg was recrystallized from 2.4 ml MeOH, filtered, washed with hexane, dried at room temperature yielding 123 mg yellow crystals.

Mp.: 158.9–160.5°C. R_f (hexane:EtOAc 3:2): 0.31

δH NMR (400 MHz, DMSO-d_6): 7.17 (t, J = 7.4 Hz, 1H, H-4), 7.37 (t, J = 7.7 Hz, 2H, H-3, H-5), 7.50 (d, J = 7.9 Hz, 2H, H-2, H-6), 7.84 (d, J = 8.7 Hz, 2H, H-3', H-5'), 8.20 (d, J = 8.8 Hz, 2H, H-2', H-6'), 10.26 (s, 1H, N*H*C-1), 10.36 (s, 1H, N*H*C-1').

δC NMR (101 MHz, DMSO-d_6): 121.7 (2C, C-3', C-5'), 123.9 (2C, C-2, C-6), 124.5 (2C, C-2', C-6'), 125.2 (C-4), 128.8 (2C, C-3, C-5), 139.2 (C-1), 142.5 (C-1'), 146.5 (C-4'), 179.6 (*C*S).

***N*-[3,5-bis(trifluoromethyl)phenyl]-*N'*-phenylthiourea (12c)**

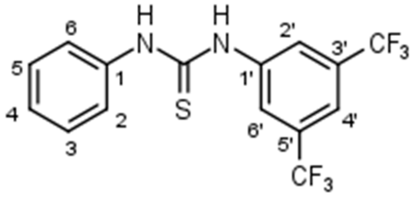

Aniline (1.0 mmol, 94 mg, 92 µl) was added to acetone (5.0 ml), then 3,5-bis(trifluoromethyl)phenyl isothiocyanate (1.1 mmol, 297 mg, 380 µl) was added and stirred at

room temperature for 1 hour. The solvent was evaporated, and the evaporation residue was treated with diethyl-ether (2.0 ml) and hexane (15.0 ml). White crystals (319 mg, 87.6%) were formed.

Mp.: 134.6–138.2°C. R_f (hexane:EtOAc 3:2): 0.77

δH NMR (400 MHz, DMSO-d_6): 7.19 (t, J = 7.3 Hz, 1H, H-4), 7.38 (t, J = 7.7 Hz, 2H, H-3, H-5), 7.46 (d, J = 8.0 Hz, 2H, H-2, H-6), 7.78 (s, 1H, H-4'), 8.26 (s, 2H, H-2', H-6'), 10.20 (s, 1H, N*H*C-1'), 10.29 (s, 1H, N*H*C-1).

δC NMR (101 MHz, DMSO-d_6): 116.8 (m, C-4'), 123.6 (m, 2C, 2*C*F_3, C-2', C-6'), 124.2 (2C, C-2=C-6), 125.4 (C-4), 129.0 (2C, C-3, C-5), 130.2 (q, J = 32.7 Hz, 2C, C-3', C-5'), 138.9 (C-1), 142.1 (C-1'), 180.1 (*C*S).

***N*-(4-methoxyphenyl)-*N*'-phenylthiourea (12d)**

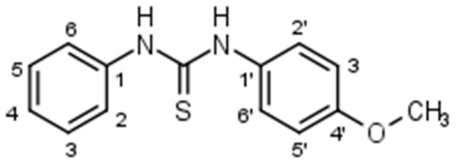

Aniline (1.0 mmol, 94 mg, 92 µl) was added to acetone (5.0 ml), then 4-methoxyphenyl isothiocyanate (1.1 mmol, 185 mg, 155 µl) was added and stirred at room temperature for 6 hours. The solvent was evaporated, and the evaporation residue was treated with diethyl-ether (2.0 ml) and hexane (15.0 ml). Yellow precipitate (175.8 mg, 68.0%) was formed, filtered and recrystallized from 5.6 ml MeOH, filtered, washed with hexane, dried at room temperature yielding 122 mg white crystals.

Mp.: 150.8–151.8°C. R_f (hexane:EtOAc 3:2): 0.55

δH NMR (400 MHz, DMSO-d_6): 3.75 (s, 3H, OC*H*$_3$), 6.91 (d, *J* = 8.9 Hz, 2H, H-3', H-5'), 7.11 (t, *J* = 7.4 Hz, 1H, H-4), 7.28 – 7.38 (m, 4H, H-3, H-5, H-2', H-6'), 7.48 (d, *J* = 7.7 Hz, 2H, H-2, H-6), 9.59 (s, 1H, N*H*C-1'), 9.61 (s, 1H, N*H*C-1).

δC NMR (101 MHz, DMSO-d_6): 55.2 (O*C*H$_3$), 113.5 (2C, C-3'=C-5'), 123.5 (2C, C-2=C-6), 124.1 (C-4), 125.8 (2C, C-2', C-6'), 128.2 (2C, C-3, C-5), 131.9 (C-1'), 139.3 (C-1), 156.3 (C-4'), 179.6 (*C*S).

***N*-(4-fluorophenyl)-*N*'-phenylthiourea (12e)**

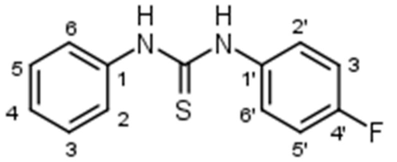

Aniline (1.0 mmol, 94 mg, 92 μl) was added to acetone (5.0 ml), then 4-fluorophenyl isothiocyanate (1.1 mmol, 169 mg) was added and stirred at room temperature for 1 hour. The solvent was evaporated, and the evaporation residue was treated with diethyl-ether (2.0 ml) and hexane (15.0 ml). White precipitate (217 mg, 88.0%) was formed, filtered and 200 mg was recrystallized from 4.4 ml MeOH, filtered, washed with hexane, dried at room temperature yielding 121 mg white crystals.

Mp.: 172.0–174.2°C. R_f (hexane:EtOAc 3:2): 0.53

δH NMR (400 MHz, DMSO-d_6): 7.13 (d, *J* = 7.6 Hz, 1H, H-4), 7.17 (t, *J* = 7.4 Hz, 2H, H-3', H-5'), 7.33 (t, *J* = 7.7 Hz, 2H, H-3, H-5), 7.41 – 7.57 (m, 4H, H-2, H-6, H-2', H-6'), 9.73 (s, 1H, N*H*C-1), 9.78 (s, 1H, N*H*C-1'),

δC NMR (101 MHz, DMSO-d_6): 115.2 (d, *J* = 22.4 Hz, 2C, C-3', C-5'), 124.7 (C-4), 126.4 (d, *J* = 8.1 Hz, 2C, C-2', C-6'), 128.6 (2C, C-3, C-5), 135.9 (d, *J* = 2.8 Hz, C-1'), 139.6 (C-1), 159.4 (d, *J* = 241.8 Hz, C-4'), 180.3 (*C*S).

***N*-(3,5-difluorophenyl)-*N*'-phenylthiourea (12f)**

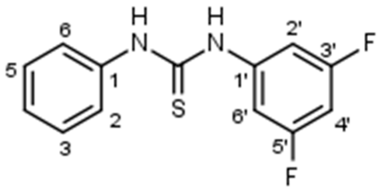

Aniline (1.0 mmol, 94 mg, 92 µl) was added to acetone (5.0 ml), then 3,5-difluorophenyl isothiocyanate (1.1 mmol, 189 mg, 150 µl) was added and stirred at room temperature for 2 hours. The solvent was evaporated and the evaporation residue was treated with diethyl-ether (2.0 ml) and hexane (15.0 ml). White crystals (241 mg, 91.2%) were formed.

Mp.: 145.2–146.4°C. R_f (hexane:EtOAc 3:2): 0.69

δH NMR (400 MHz, DMSO-d_6): 6.94 (t, *J* = 9.3 Hz, 1H, H-4'), 7.16 (t, *J* = 7.4 Hz, 1H, H-4), 7.24 – 7.41 (m, 4H, H-3, H-5, H2', H6'), 7.46 (d, *J* = 7.9 Hz, 2H, H-2, H-6), 10.05 (s, 1H, N*H*C-1'), 10.06 (s, 1H, N*H*C-1).

δC NMR (101 MHz, DMSO-d_6): 99.2 (t, *J* = 26.3 Hz, C-4'), 105.7 (d, *J* = 28.5 Hz, 2C, C-2', C-6'), 124.0 (2C, C-2, C-6), 125.1 (C-4), 128.8 (2C, C-3, C-5), 139.2 (C-1), 142.6 (t, *J* = 13.5 Hz, C-1'), 162.3 (dd, *J* = 243.7, 15.2 Hz, 2C, C-3', C-5'), 179.8 (*C*S).

***N*-(2,6-difluorophenyl)-*N*'-phenylthiourea (12g)**

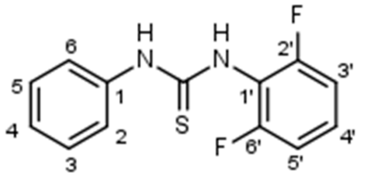

Aniline (1.0 mmol, 94 mg, 92 µl) was added to acetone (5.0 ml), then 4-methoxyphenyl isothiocyanate (1.1 mmol, 192 mg, 145 µl) was added and stirred at room temperature for 2 hours. The solvent was evaporated and the evaporation residue was treated with diethyl-ether (2.0 ml) and hexane (15.0 ml). White crystals (219 mg, 82.8%) were formed.

Mp.: 154.8–160.4°C. R_f (hexane:EtOAc 3:2): 0.58

δH NMR (400 MHz, DMSO-d_6): 7.07 – 7.21 (m, 3H, H-3', H-4', H-5'), 7.29 – 7.44 (m, 3H, H-3, H-4, H-5), 7.48 (d, *J* = 7.9 Hz, 2H, H-2, H-6), 9.18 (s, 1H, N*H*C-1'), 10.10 (s, 1H, N*H*C-1),

δC NMR (101 MHz, DMSO-d_6): 111.9 (d, *J* = 23.3 Hz, 2C, C-3', C-5'), 116.8 (t, *J* = 16.4 Hz, C-1'), 123.9 (C-2, C-6), 125.0 (C-4), 128.8 (C-3, C-5), 128.8 (t, *J* = 10.1 Hz, C-4'), 139.3 (C-1), 159.0 (dd, *J* = 248.9, 4.8 Hz, C-2', C-6'), 181.8 (*C*S).

IR spectra

Wavenumber [cm-1]	Methyl (2*S*)-3-methyl-2-({2-[(phenylcarbamothioyl)amino]benzoyl}amino)butanoate (9a)
3333.36	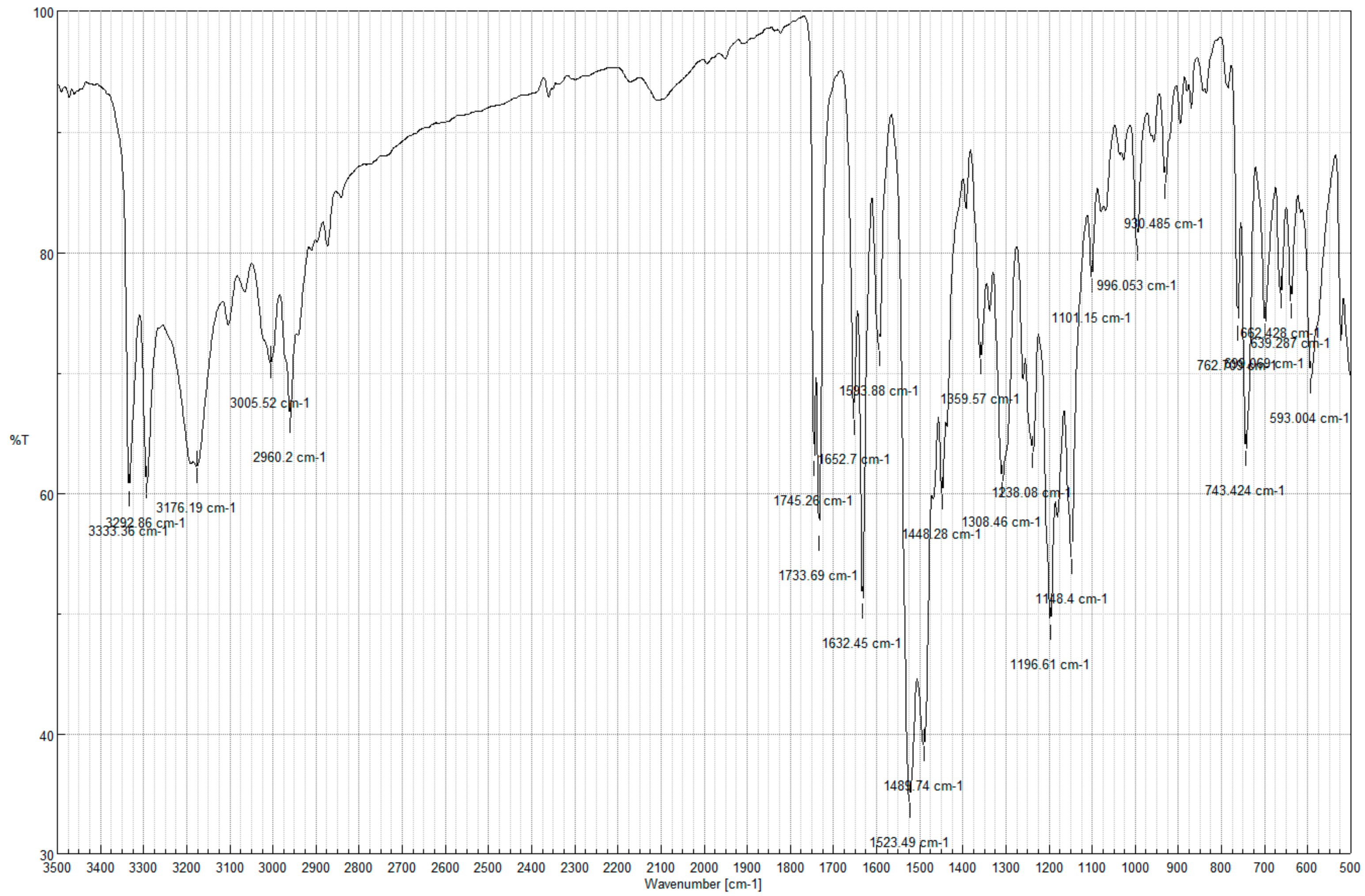
3292.86	
3176.19	
3005.52	
2960.20	
1745.26	
1733.69	
1652.7	
1632.45	
1593.88	
1523.49	
1489.74	
1448.28	
1359.57	
1308.46	
1238.08	
1196.61	
1148.4	
1101.15	
996.053	
930.485	
762.709	
743.424	
699.069	
662.428	
639.287	

Wavenumber [cm-1]

3726.76
3624.55
3019.010
2399.98
2359.48
1736.58
1648.84
1598.7
1514.81
1449.24
1339.32
1213.97
1112.73
1045.23
929.521
853.347
743.424
666.285
627.716

Methyl (2*S*)-3-methyl 2-[(2-{[(4-nitrophenyl)carbamothioyl]amino}benzoyl)amino]butanoate (9b)

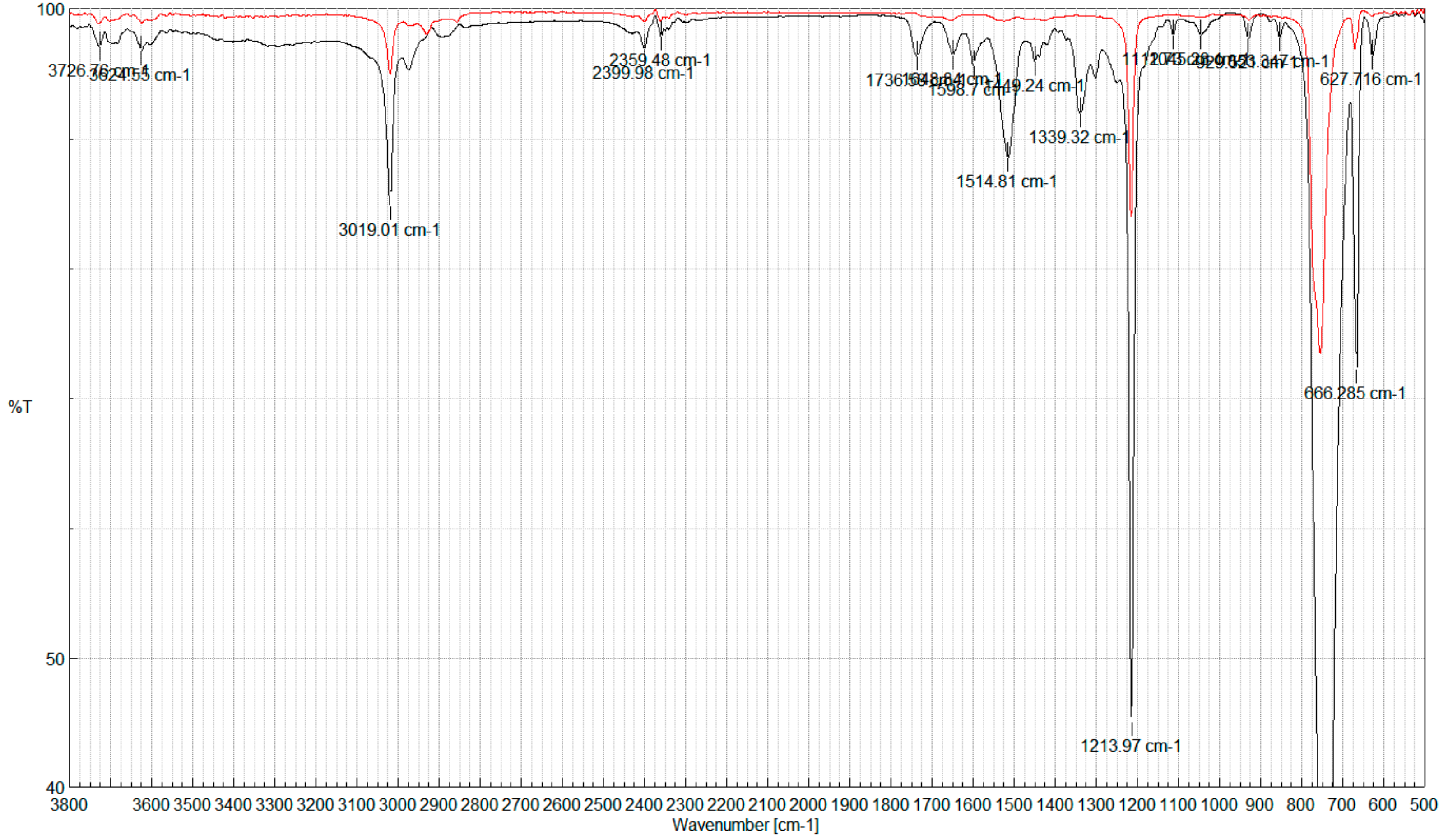

Wavenumber [cm-1]	Methyl (2*S*)-3-methyl 2-[(2-{[(3,5-bistrifluoromethylphenyl)carbamothioyl]amino} benzoyl)amino]butanoate (9c)
3100.97	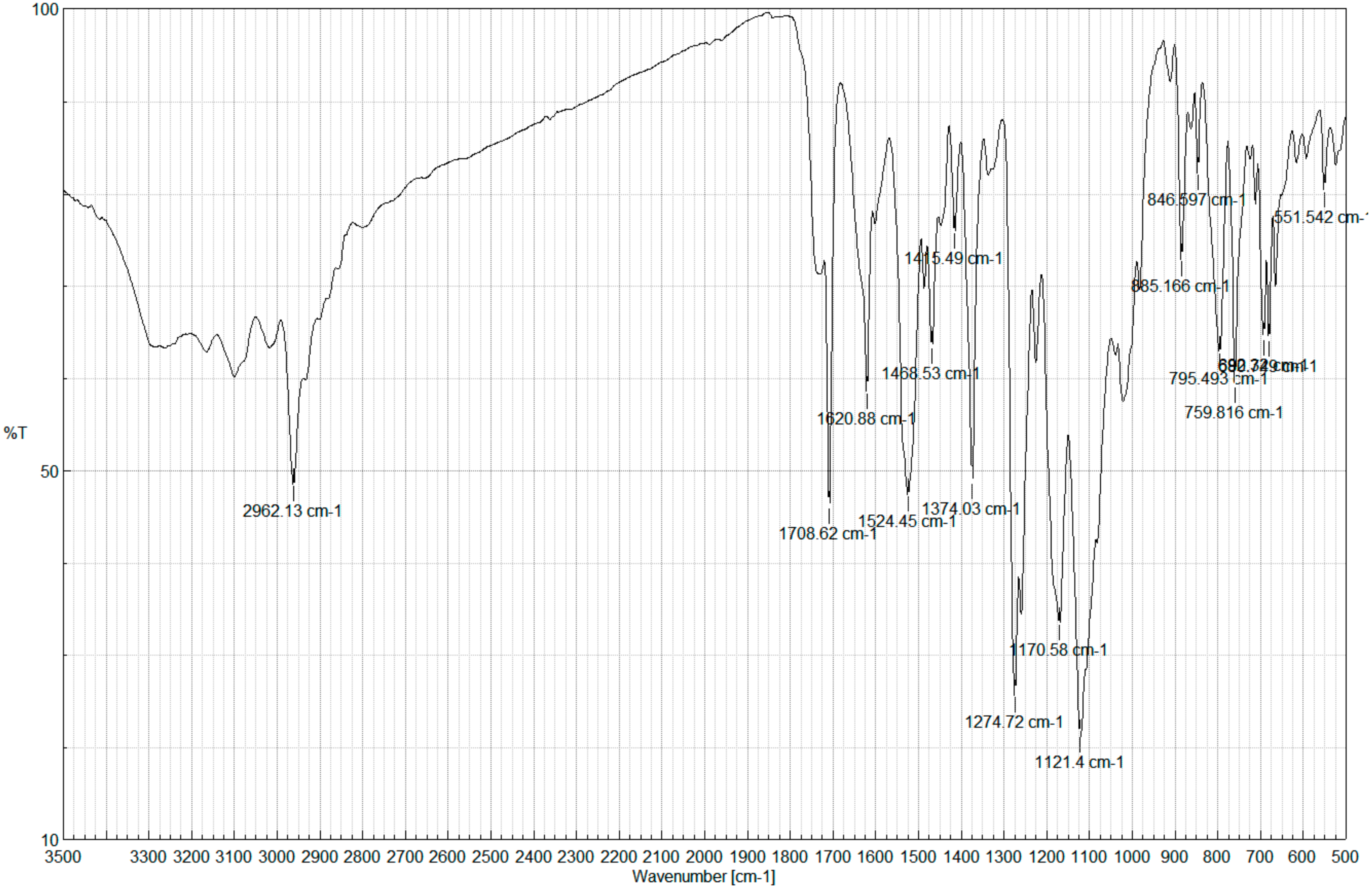
3018.05	
2962.13	
1733.69	
1708.62	
1620.88	
1524.45	
1486.85	
1468.53	
1415.49	
1374.03	
1337.39	
1274.72	
1260.25	
1225.54	
1170.58	
1121.4	
1021.12	
982.554	
911.201	
885.166	
846.597	
795.493	
759.816	
711.604	
692.32	
680.749	
665.321	

Wavenumber [cm-1]
3216.68
3003.59
2957.3
2915.84
2848.35
2360.44
1736.58
1658.48
1619.91
1529.27
1507.1
1450.21
1406.82
1336.43
1240
1197.58
1164.79
1104.05
1030.77
820.563
757.888
722.211
691.355
674.963

Methyl (2*S*)-3-methyl 2-[(2-{[(4-methoxyphenyl)carbamothioyl]amino}benzoyl)amino]butanoate (9d)

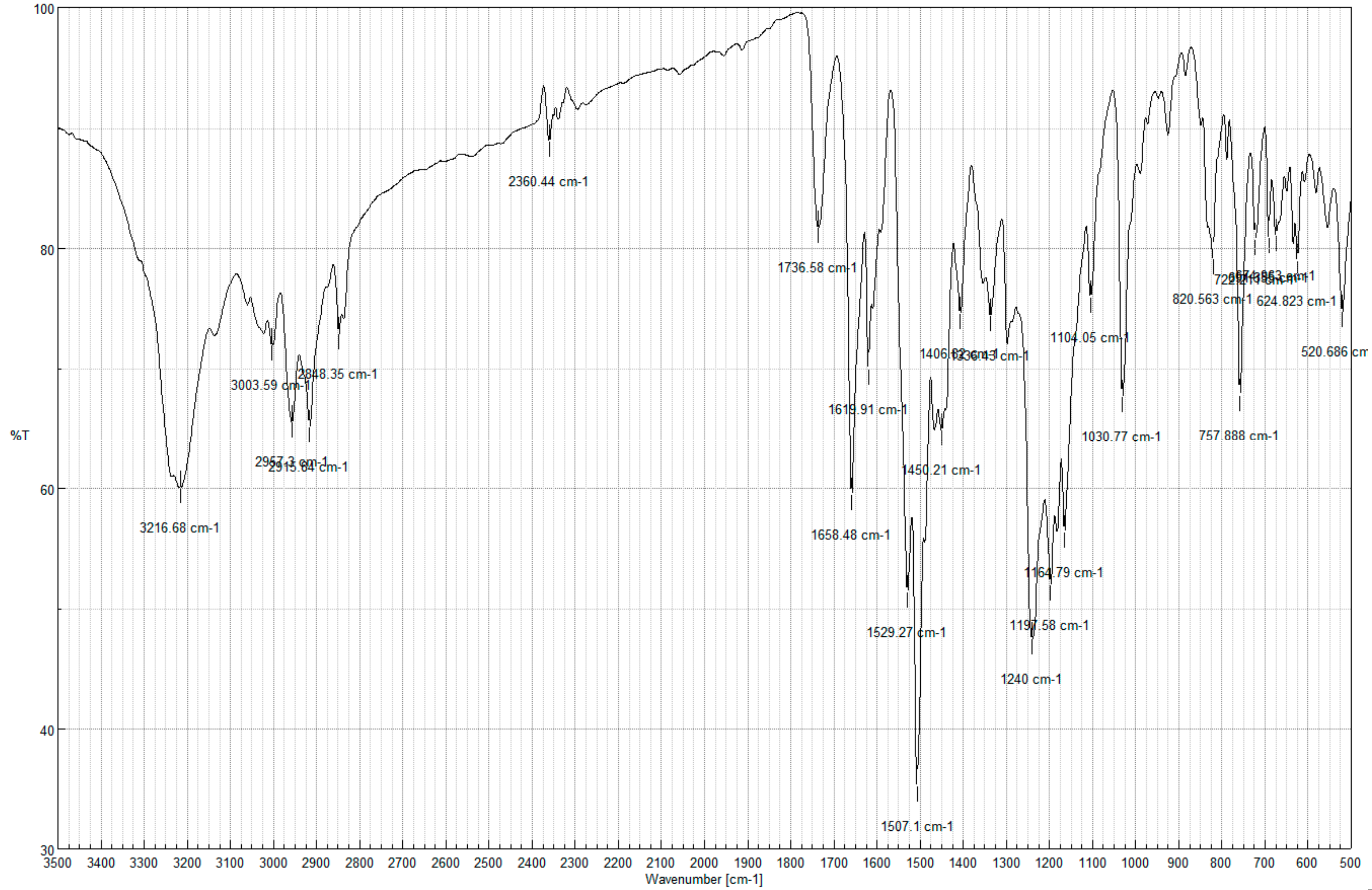

624.823<br>520.686	

Wavenumber [cm-1]
555,398
648,929
675,928
701,962
744,388
831,169
926,628
950,734
997,982
1030,77
1089,58
1148,4
1207,22
1269,9
1350,89
1438,64
1504,2
1523,49
1588,09
1620,88
1659,45
1737,55
2967,91
3241,75
3332,39

Methyl (2*S*)-3-methyl 2-[(2-{[(4-fluorophenyl)carbamothioyl]amino}benzoyl)amino]butanoate (9e)

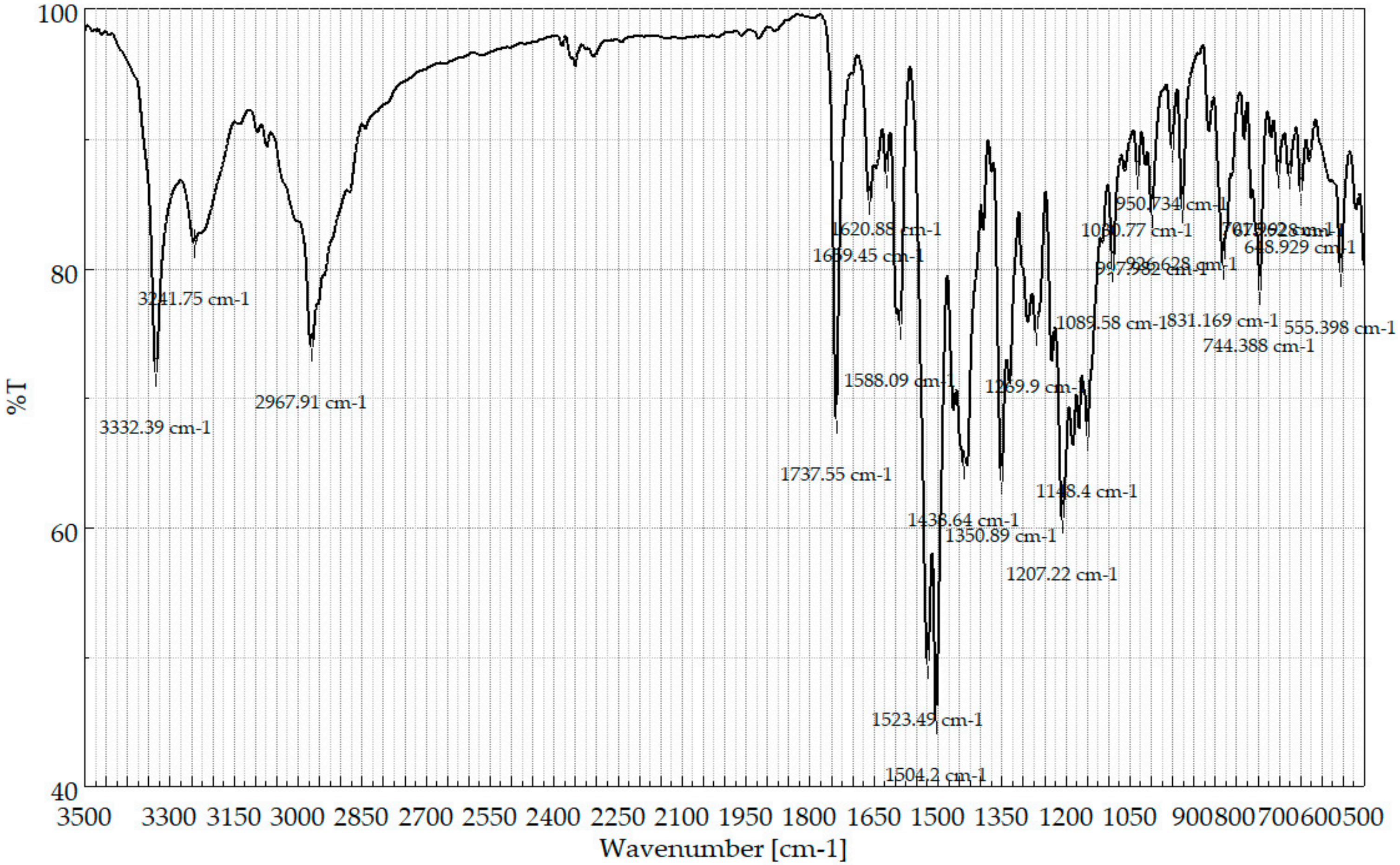

Wavenumber [cm-1]
510,08
584,325
645,072
664,357
728,961
781,993
835,99
990,268
1020,16
1111,76
1201,43
1237,11
1299,79
1358,6
1475,28
1542,77
1578,45
1599,66
1617,02
1738,51
2348,87
2967,91
3177,15
3317,93
3405,67
3725,8

Methyl (2*S*)-3-methyl 2-[(2-{[(3,5-difluorophenyl)carbamothioyl]amino}benzoyl)amino]butanoate (9f)

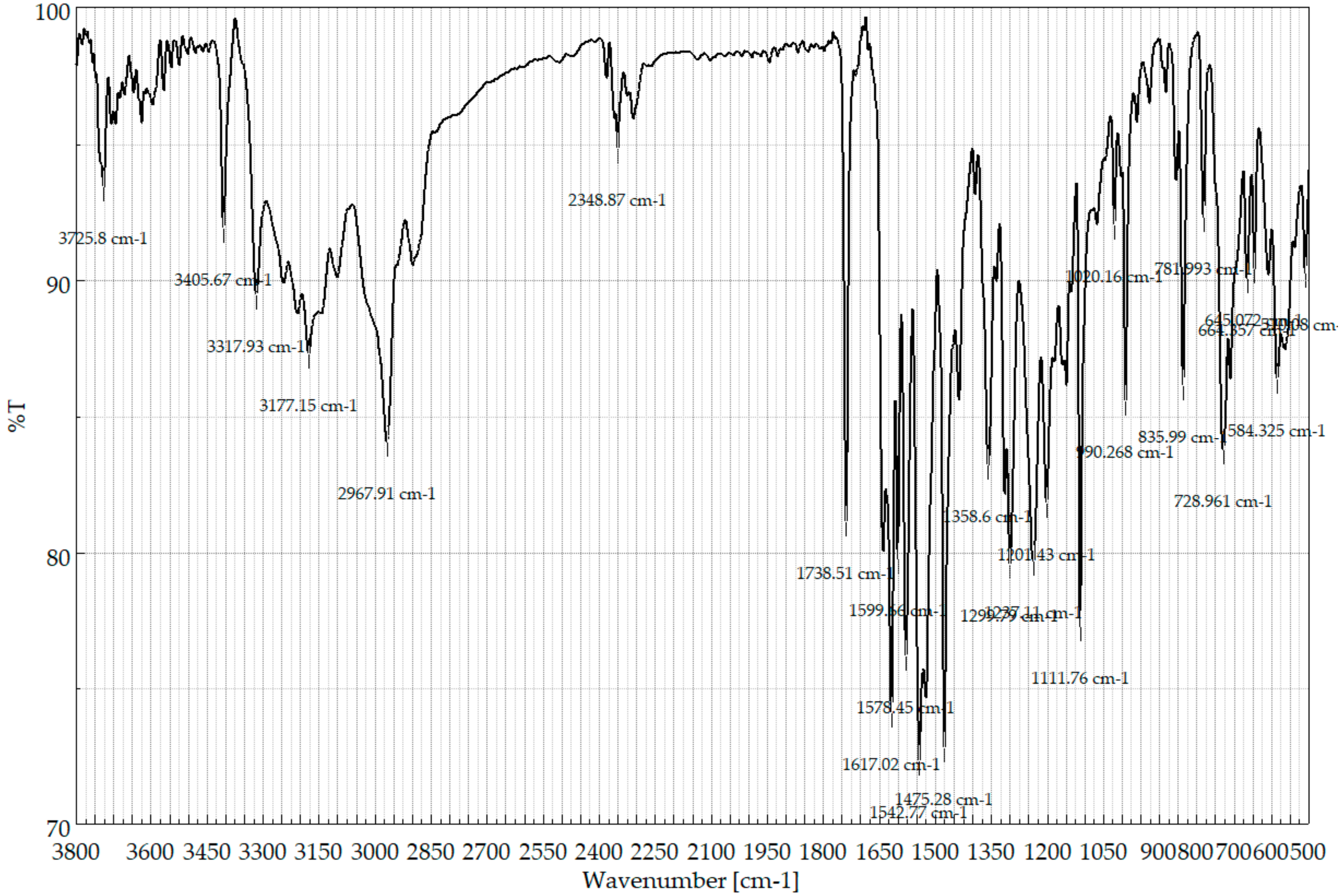

Wavenumber [cm-1]	Methyl (2*S*)-3-methyl 2-[(2-{[(2,6-difluorophenyl)carbamothioyl]amino}benzoyl)amino]butanoate (9g)
522,615<br>547,685<br>568,898<br>639,287<br>759,816<br>779,101<br>854,311<br>871,667<br>926,628<br>1007,62<br>1057,76<br>1101,15<br>1136,83<br>1154,19<br>1199,51<br>1242,9<br>1299,79<br>1350,89<br>1371,14<br>1469,49<br>1485,88<br>1530,24<br>1597,73<br>1620,88<br>1655,59<br>1738,51<br>2962,13<br>3155,94<br>1617,98<br>1734,66<br>2877,27<br>2933,2<br>2965,02<br>3031,55<br>3100,01<br>3179,08<br>3323,71<br>3401,82	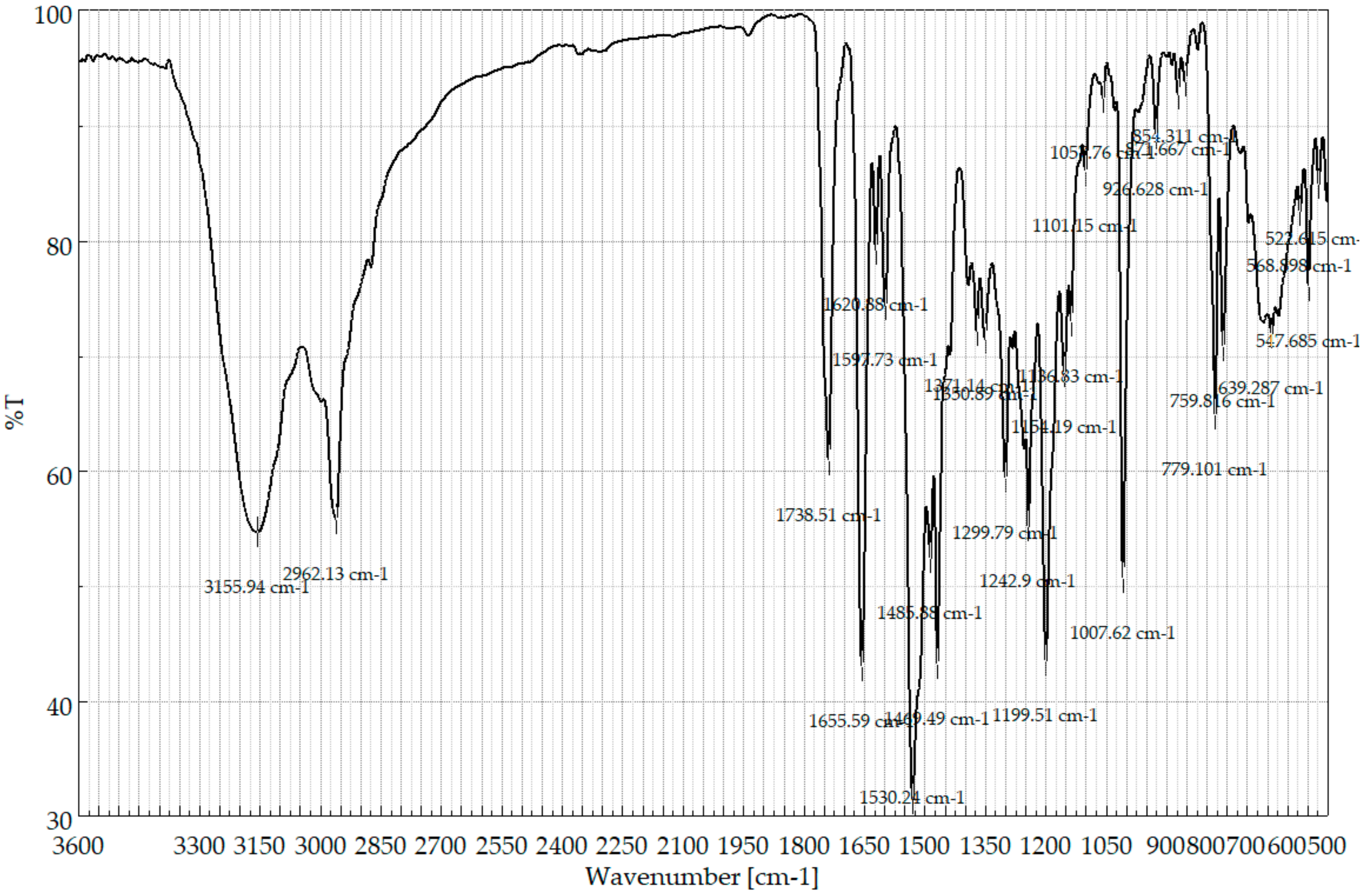

Wavenumber [cm-1]	Methyl (2*S*)-4-methyl 2-({2-[(phenylcarbamothioyl)amino]benzoyl}amino)pentanoate (10a)
3363.25<br>3192.58<br>3018.05<br>2960.2<br>1739.48<br>1648.84<br>1596.77<br>1519.63<br>1446.35<br>1355.71<br>1213.97<br>745.352<br>693.284<br>664.357	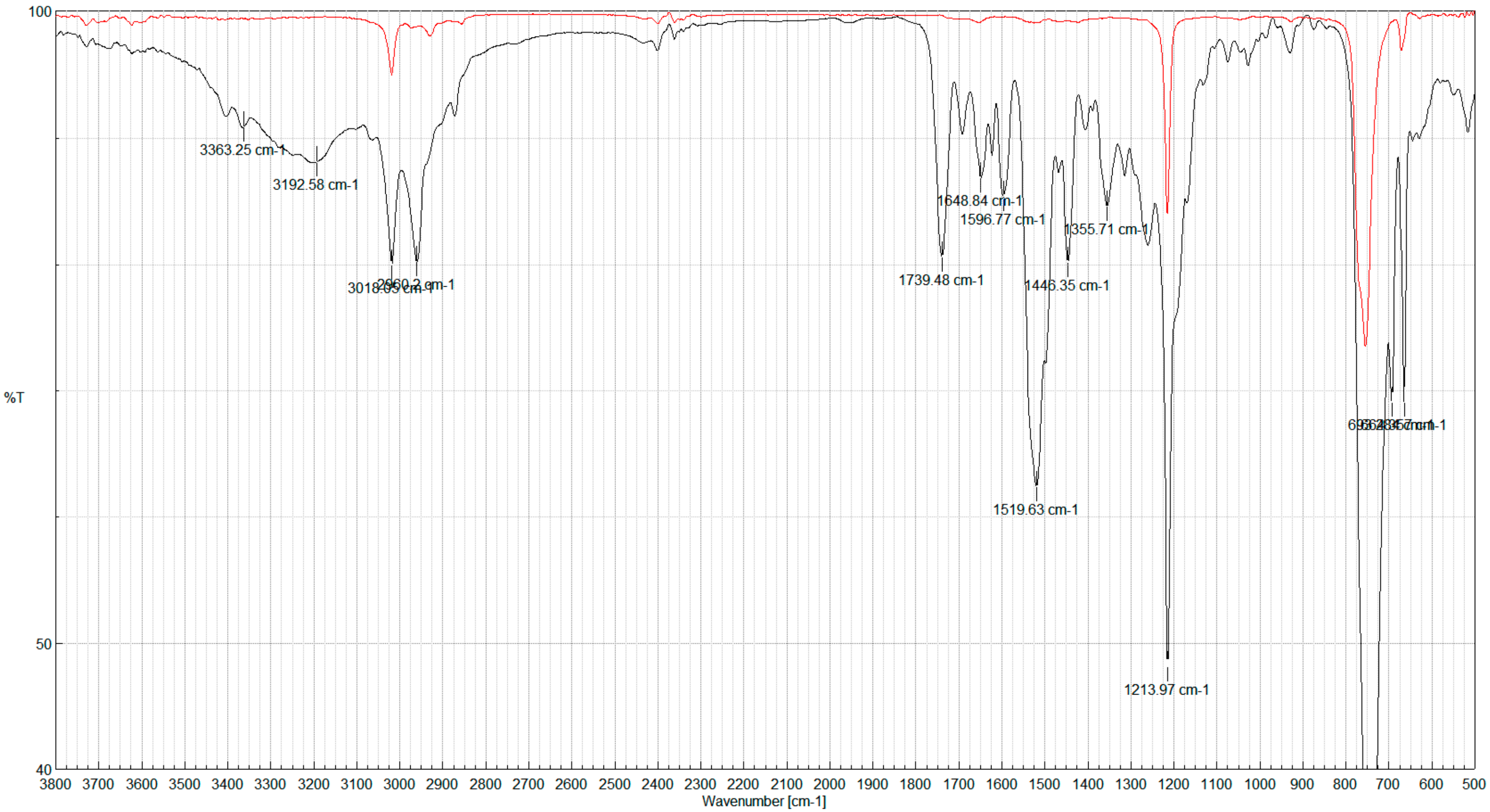

Wavenumber [cm-1]
3325.64
3228.25
3065.30
2953.45
2919.70
2851.24
1742.37
1668.12
1623.77
1595.81
1529.27
1502.28
1443.46
1409.71
1339.32
1264.11
1195.65
1110.8
1017.27
982.554
923.736
905.415
850.454
825.384
794.528

Methyl (2*S*)-4-methyl 2-[(2-{[(4-nitrophenyl)carbamothioyl]amino}benzoyl)amino]pentanoate (10b)

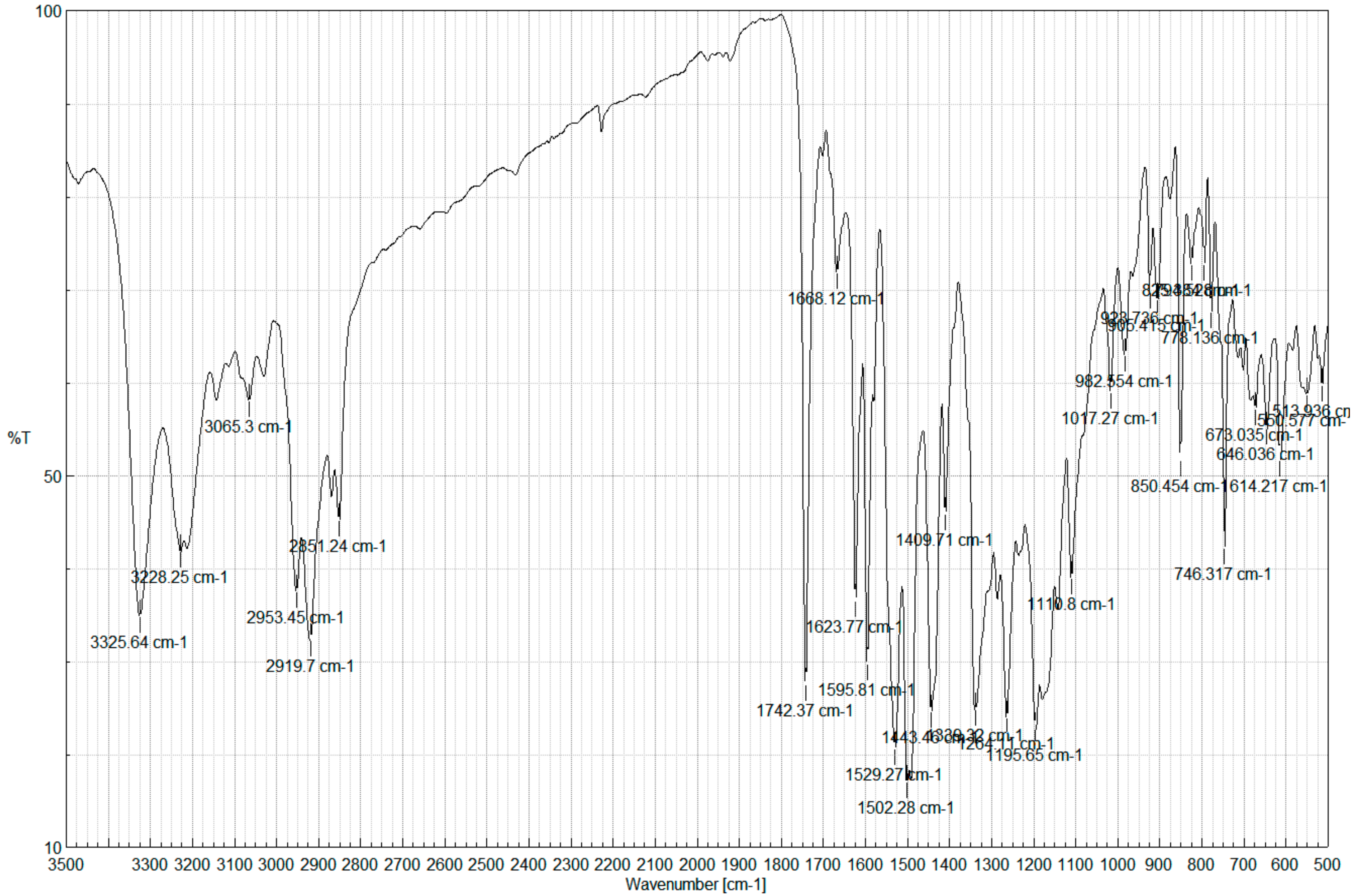

Methyl (2*S*)-4-methyl 2-[(2-{[(3,5-bistrifluoromethylphenyl)carbamothioyl]amino}-benzoyl)amino]pentanoate (10c)

Wavenumber [cm-1]
3726.76
3622.63
3528.13
3262.00
3012.27
2964.051
2360.44
2294.88
1713.44
1622.8
1600.63
1554.34
1525.42
1469.49
1439.6
1383.68
1350.89
1321.96
1269.9
1192.76
1168.65
1123.33
1020.16
980.625
926.628

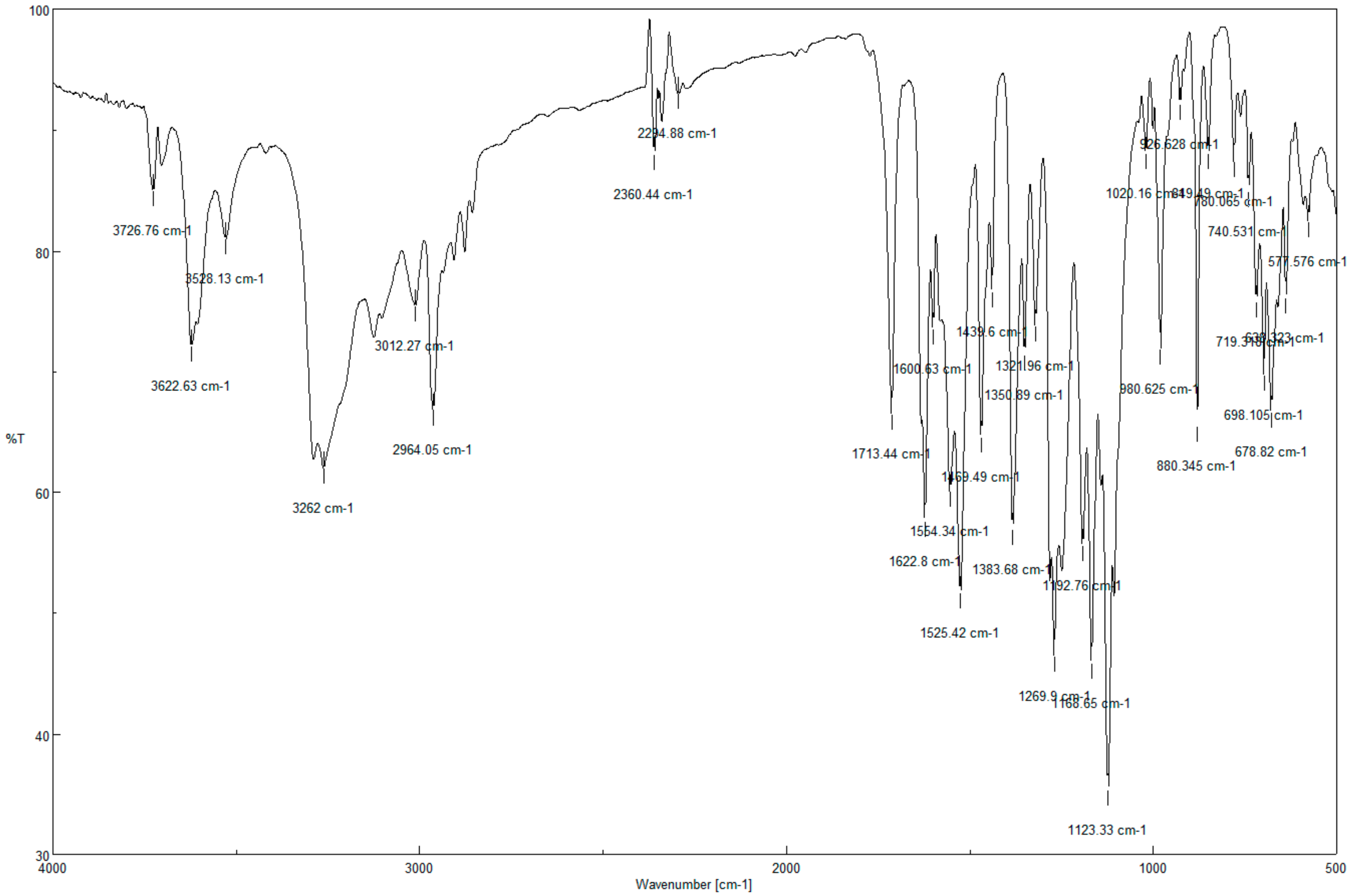

Wavenumber [cm-1]
3362.28
3006.48
2956.34
2870.52
1750.08
1644.02
1586.16
1508.06
1435.74
1348.00
1299.79
1274.72
1249.65
1223.61
1180.22
1159.01
1103.08
1029.8
985.447
945.913
920.843
870.703
821.527
787.779
755.959

Methyl (2*S*)-4-methyl 2-[(2-{[(4-methoxyphenyl)carbamothioyl]amino}benzoyl)amino]pentanoate (10d)

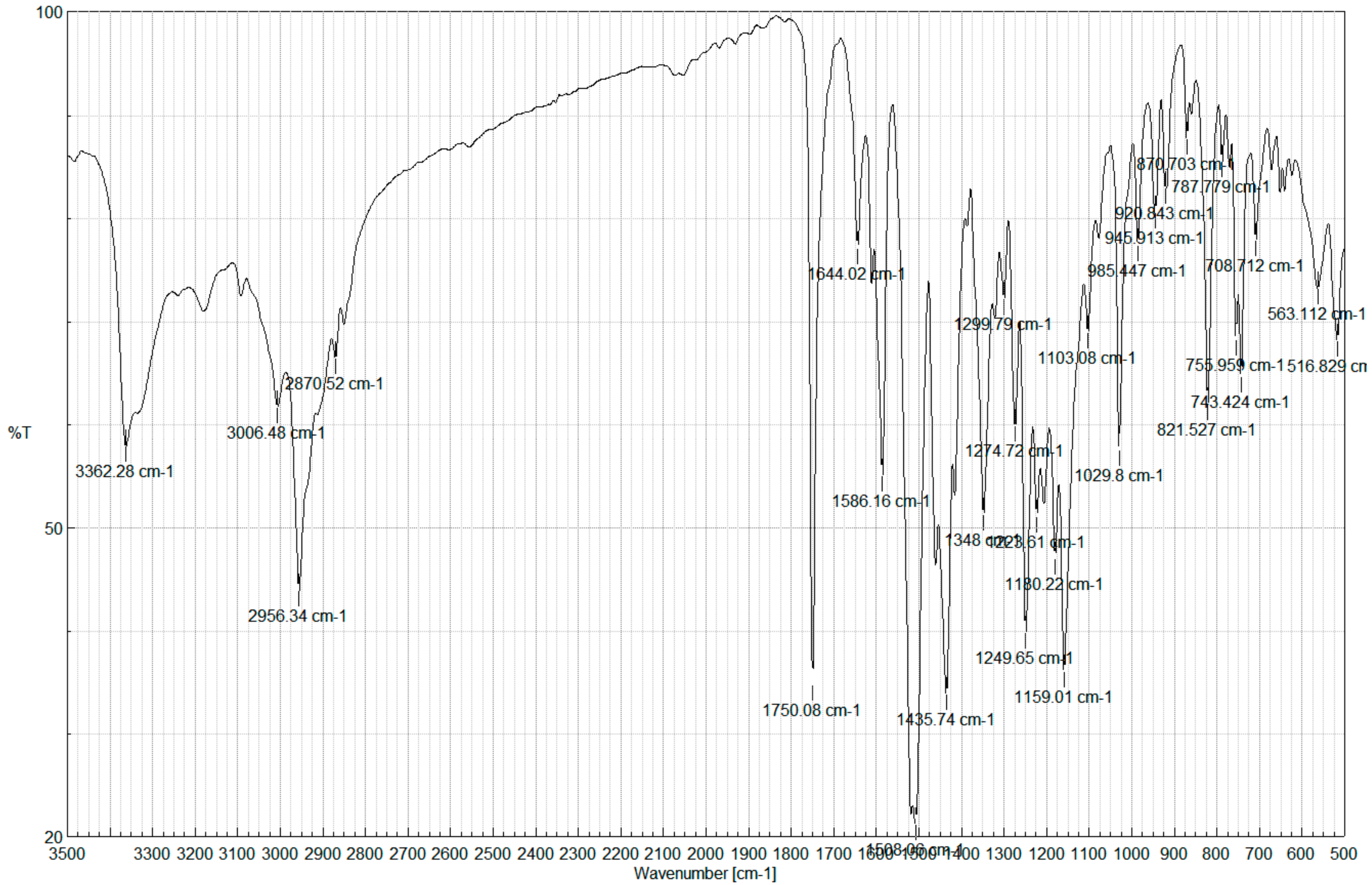

Wavenumber [cm-1]	**Methyl (2*S*)-4-methyl 2-[(2-{[(4-fluorophenyl)carbamothioyl]amino}benzoyl)amino]pentanoate (10e)**
511,044	
553,47	
589,147	
650,858	
700,034	
745,352	
782,958	
831,169	
864,917	
926,628	
958,448	
1077,05	
1149,37	
1168,65	
1210,11	
1272,79	
1350,89	
1436,71	
1504,2	
1522,52	
1591,95	
1643,05	
1742,37	
2349,84	
2901,38	
2986,23	
3339,14	
3672,77	

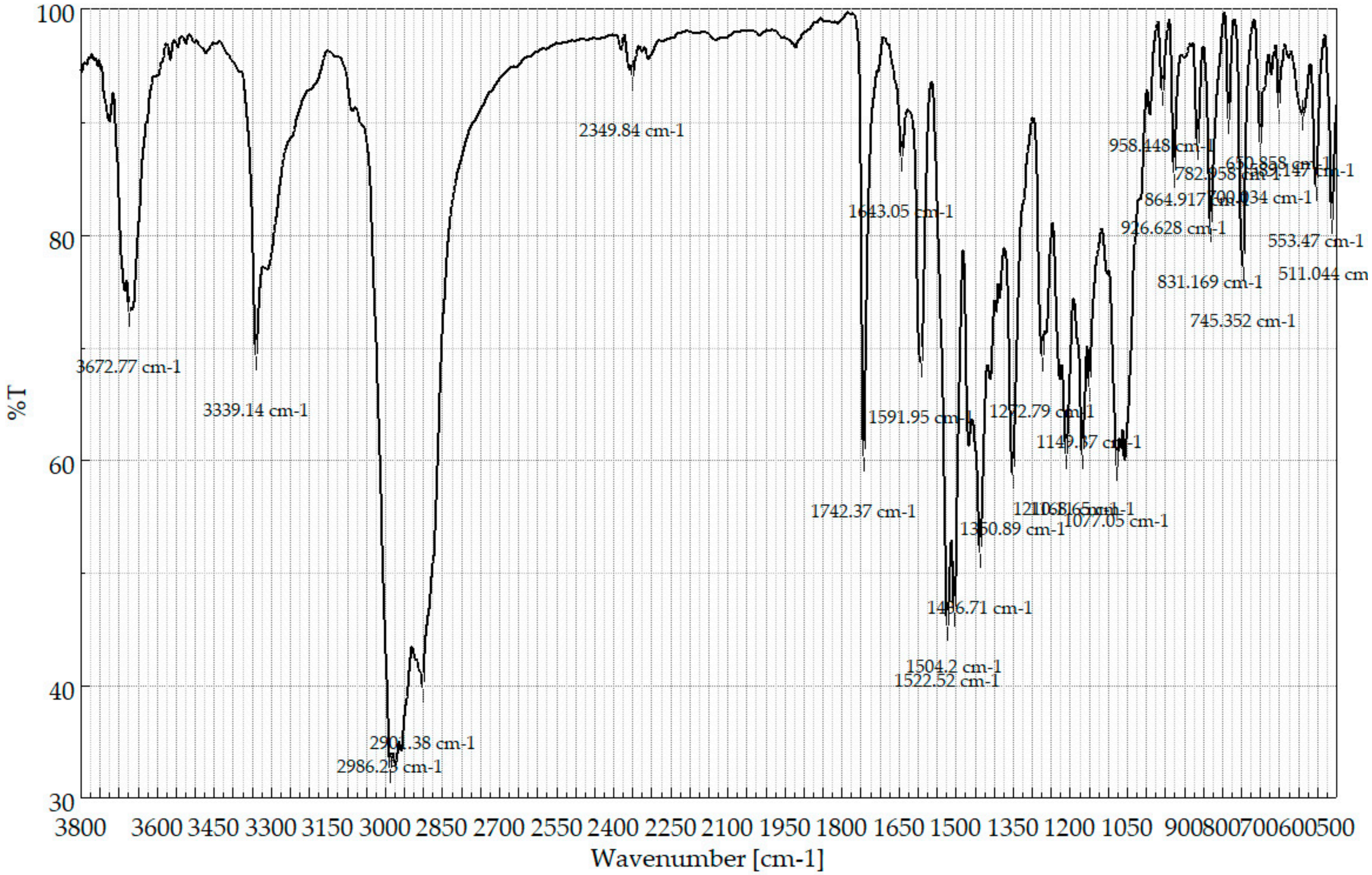

Wavenumber [cm-1]	**Methyl (2*S*)-4-methyl 2-[(2-{[(3,5-difluorophenyl)carbamothioyl]amino}benzoyl)amino]pentanoate (10f)**
508,151<br>553,47<br>644,108<br>706,783<br>727,032<br>835,99<br>883,238<br>925,664<br>989,304<br>1018,23<br>1111,76<br>1159,01<br>1200,47<br>1250,61<br>1296,89<br>1356,68<br>1440,56<br>1475,28<br>1524,45<br>1573,63<br>1599,66<br>1628,59<br>1740,44<br>2349,84<br>2958,27<br>3173,29<br>3321,78<br>3406,64	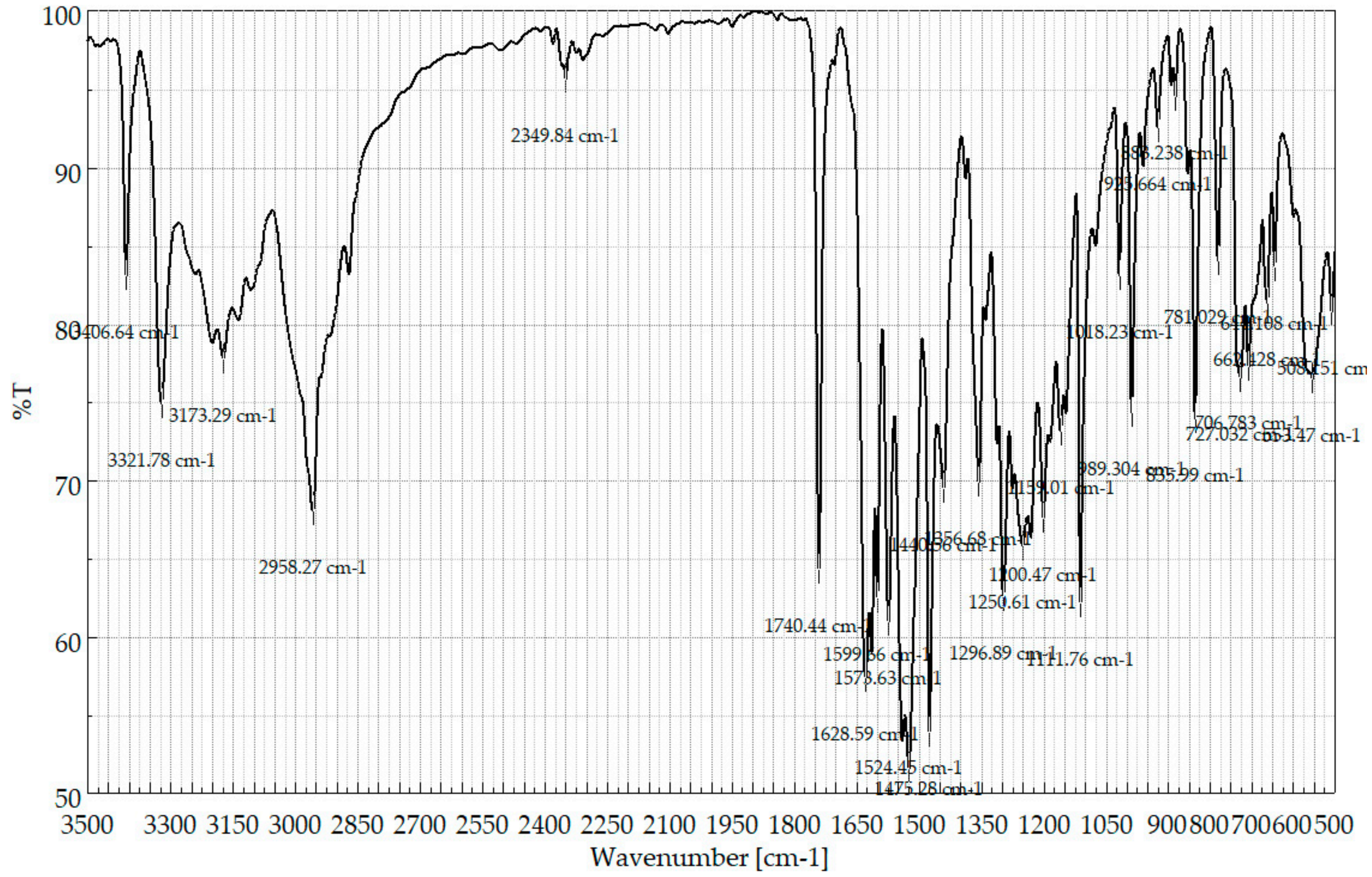

Wavenumber [cm-1]	**Methyl (2*S*)-4-methyl 2-[(2-{[(2,6-difluorophenyl)carbamothioyl]amino}benzoyl)amino]pentanoate (10g)**
512,972<br>554,434<br>648,929<br>671,106<br>695,212<br>719,318<br>748,245<br>782,958<br>858,168<br>936,271<br>984,482<br>1007,62<br>1074,16<br>1159,97<br>1183,11<br>1227,47<br>1244,83<br>1277,61<br>1293,04<br>1348,96<br>1434,78<br>1459,85<br>1474,31<br>1493,6<br>1518,67<br>1592,91<br>1620,88<br>1642,09<br>1744,3<br>2871,49<br>2958,27<br>3331,43	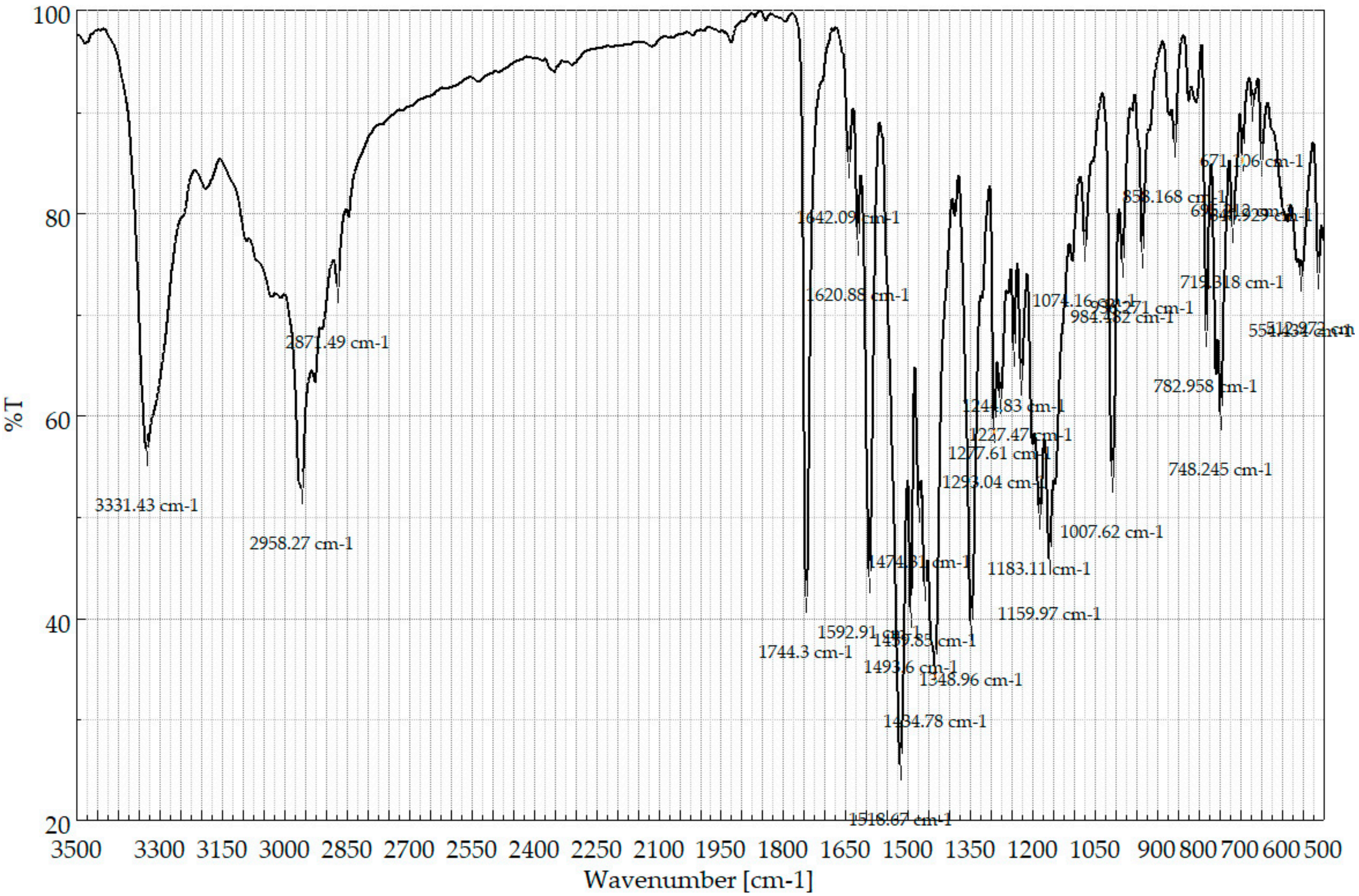

Wavenumber [cm-1]	
3346.85	
3286.110	
2954.41	
2936.02	
2876.34	
1737.55	
1643.05	
1587.13	
1519.63	
1491.67	
1453.1	
1430.92	
1383.68	
1350.89	
1279.54	
1233.25	
1179.26	
1101.15	
1066.44	
988.339	
945.913	
911.201	
868.774	
841.776	
814.777	
770.423	

Methyl (2*S*)-3-methyl 2-({2-[(phenylcarbamothioyl)amino]benzoyl}amino)pentanoate (11a)

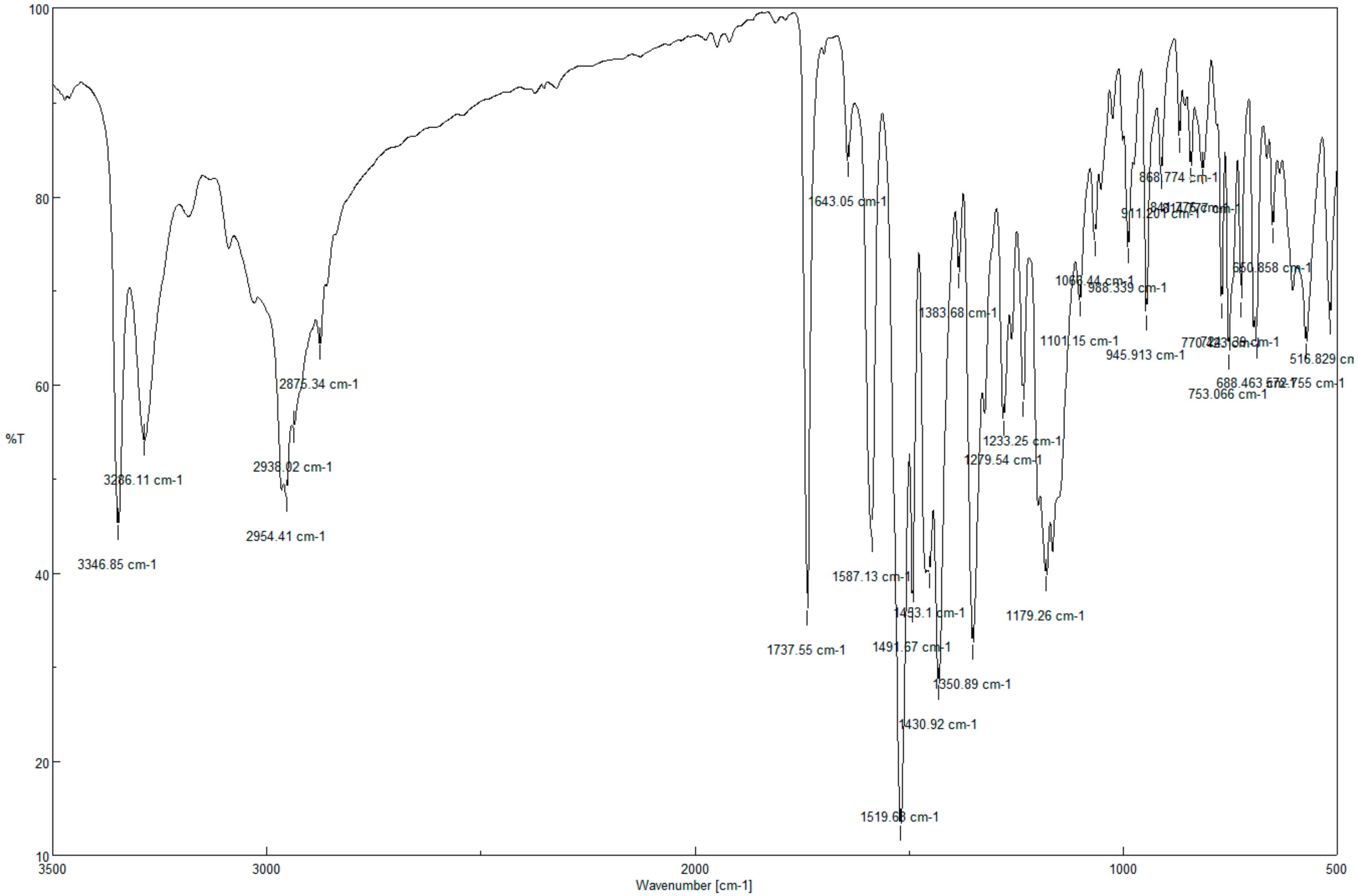

Wavenumber [cm-1]
3284.18
2962.13
2925.48
2875.34
1737.55
1642.09
1595.81
1504.2
1448.28
1326.79
1296.89
1247.72
1176.36
1109.83
1011.48
930.485
851.418
750.174
725.104
690.391
582.397
528.4

Methyl (2*S*)-3-methyl 2-[(2-{[(4-nitrophenyl)carbamothioyl]amino}benzoyl)amino]pentanoate (11b)

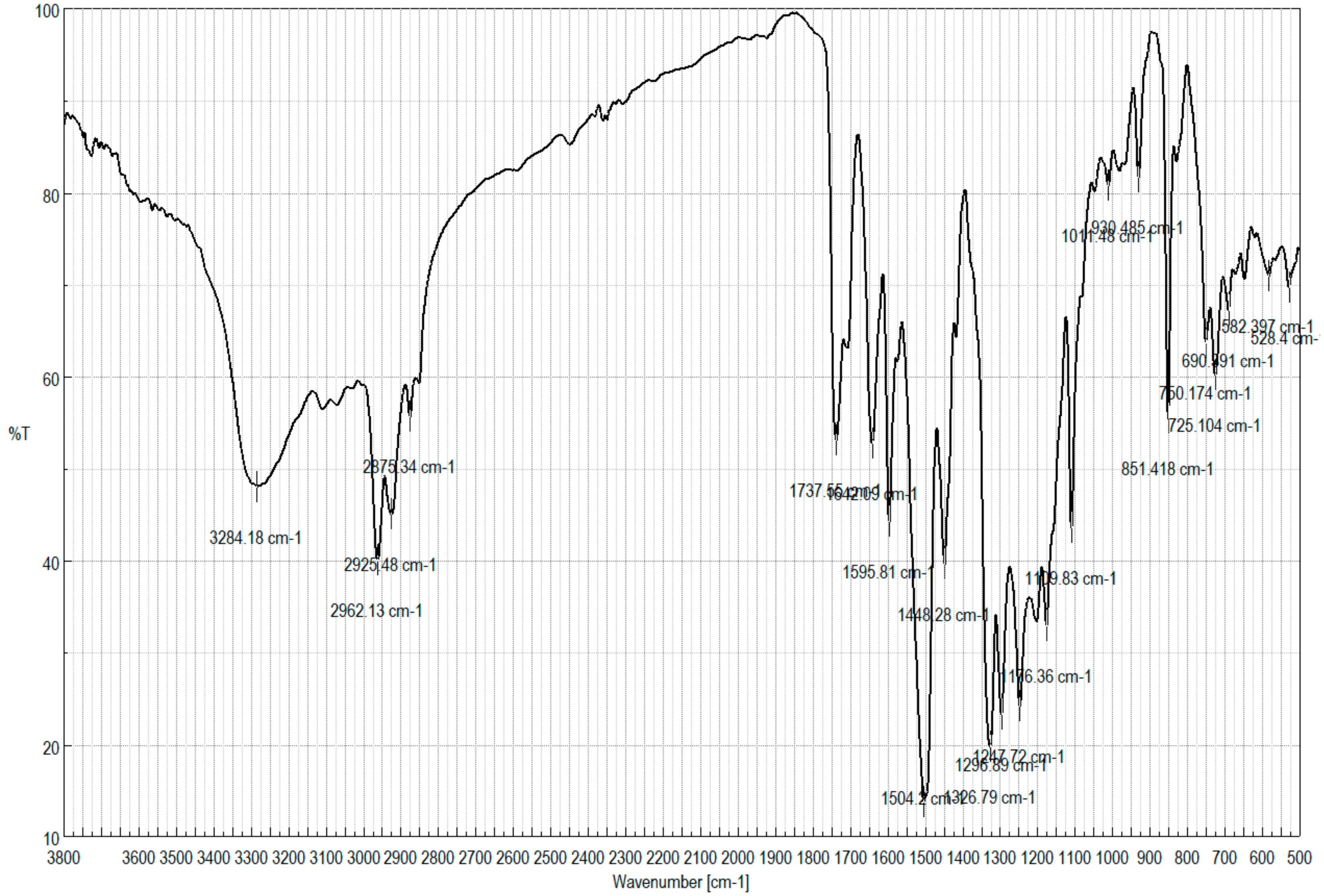

Wavenumber [cm-1]
3622.63
3531.020
3293.82
2971.77
1725.98
1621.84
1599.66
1524.45
1471.42
1381.75
1323.89
1270.86
1191.79
1168.65
1123.33
980.625
879.381
848.525
678.82
639.287
593.004

Methyl (2*S*)-3-methyl 2-[(2-{[(3,5-bistetrifluoromethylphenyl)carbamothioyl]amino} benzoyl)amino]pentanoate (11c)

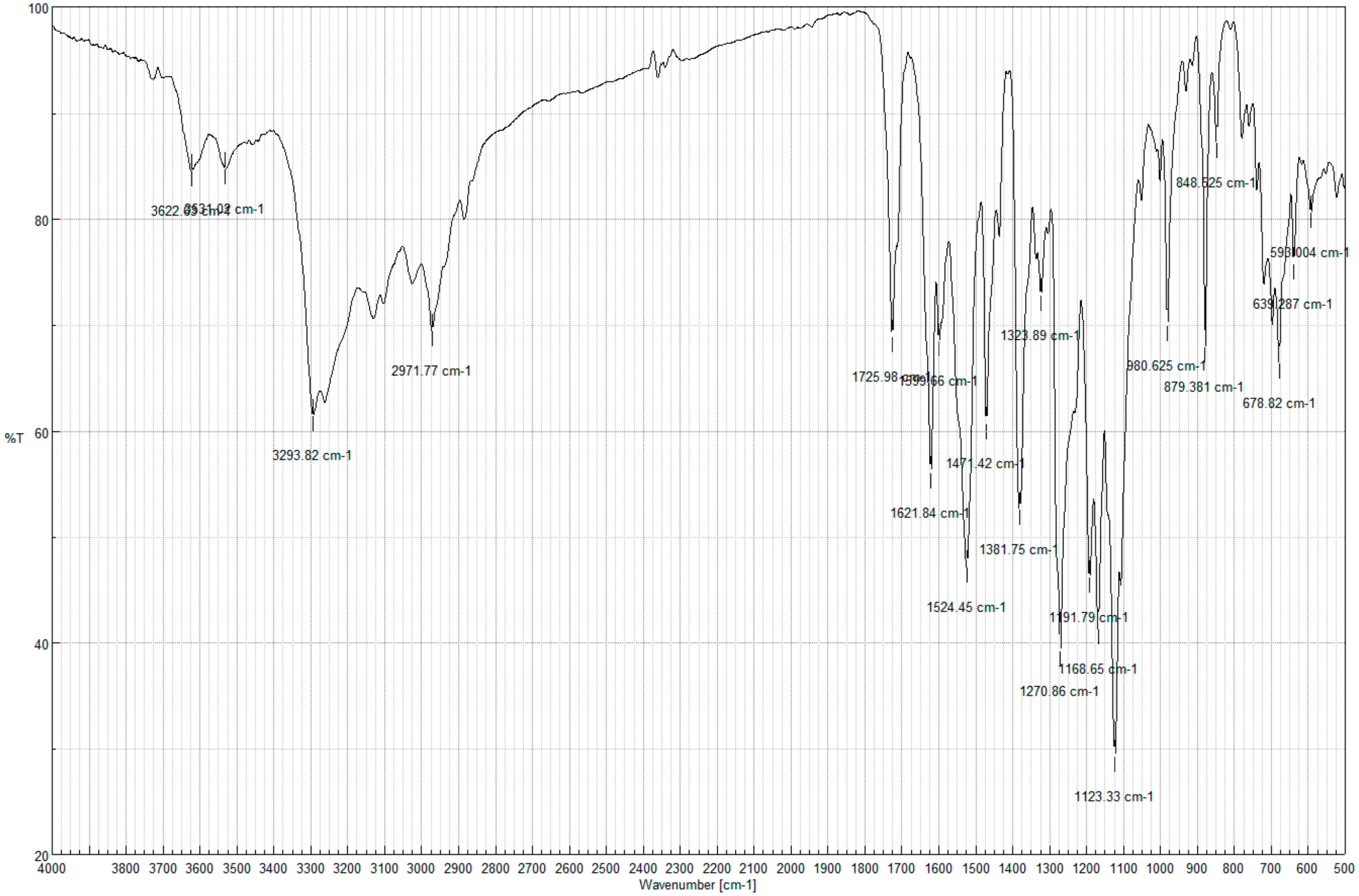

Wavenumber [cm-1]	**Methyl (2*S*)-3-methyl 2-[(2-{[(4-methoxyphenyl)carbamothioyl]amino}benzoyl)amino]pentanoate (11d)**
3395.070<br>3154.01<br>2965.020<br>2110.71<br>1735.62<br>1698.02<br>1644.02<br>1589.06<br>1503.24<br>1448.28<br>1350.89<br>1298.82<br>1238.08<br>1168.65<br>1101.15<br>1029.8<br>944.949<br>923.736<br>825.384<br>746.317<br>703.89<br>613.252<br>559.255<br>519.722	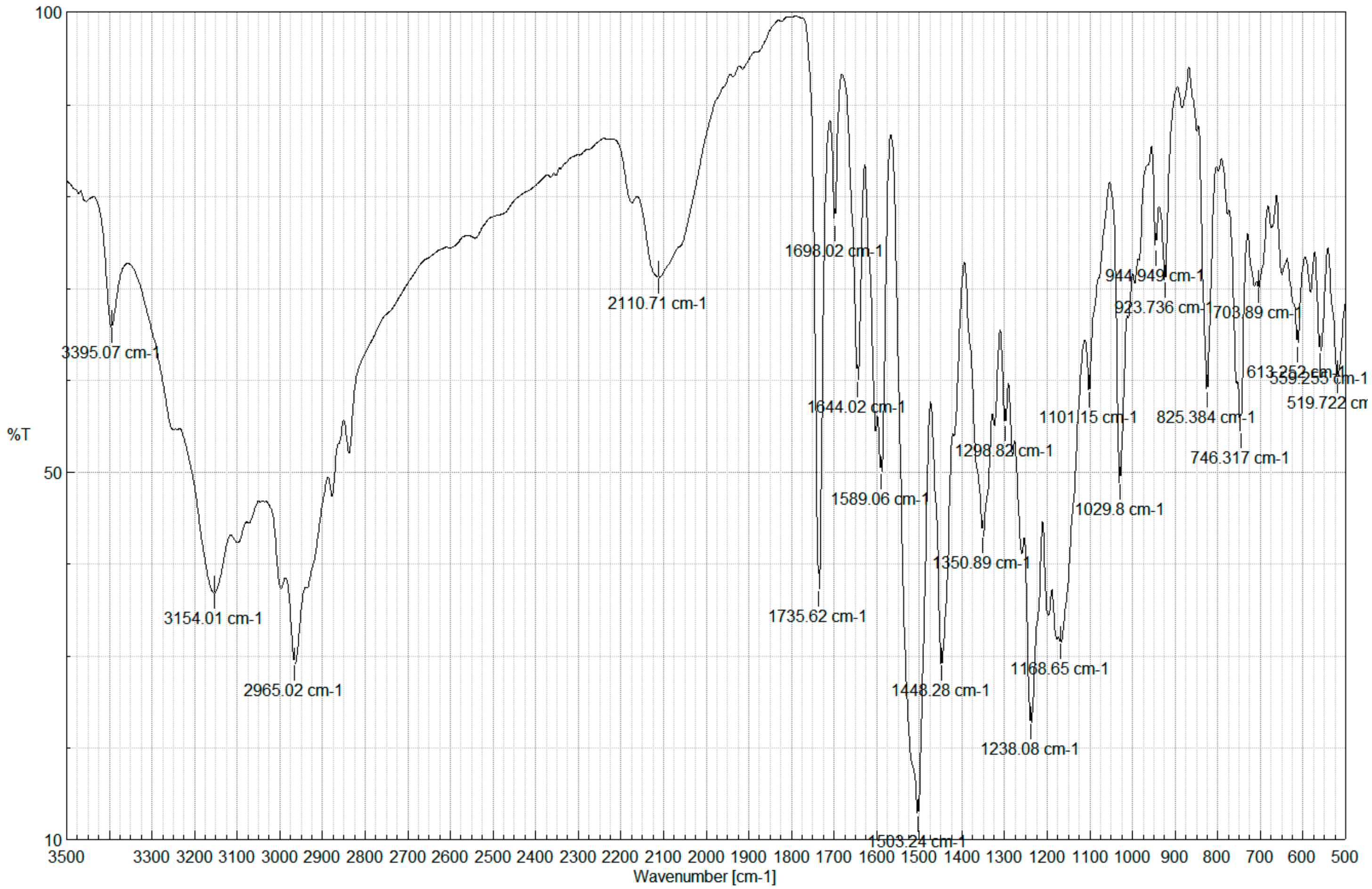

Wavenumber [cm-1]	**Methyl (2*S*)-3-methyl 2-[(2-{[(4-fluorophenyl)carbamothioyl]amino}benzoyl)amino]pentanoate (11e)**
513,936<br>555,398<br>590,111<br>620,966<br>650,858<br>701,962<br>741,496<br>781,029<br>833,098<br>866,846<br>933,378<br>946,877<br>989,304<br>1013,41<br>1056,8<br>1090,55<br>1152,26<br>1187,94<br>1211,08<br>1229,4<br>1241,93<br>1269,9<br>1285,32<br>1368,25<br>1451,17<br>1513,85<br>1590,99<br>1656,55<br>1738,51<br>2348,87<br>2967,91<br>3168,47<br>3348,78	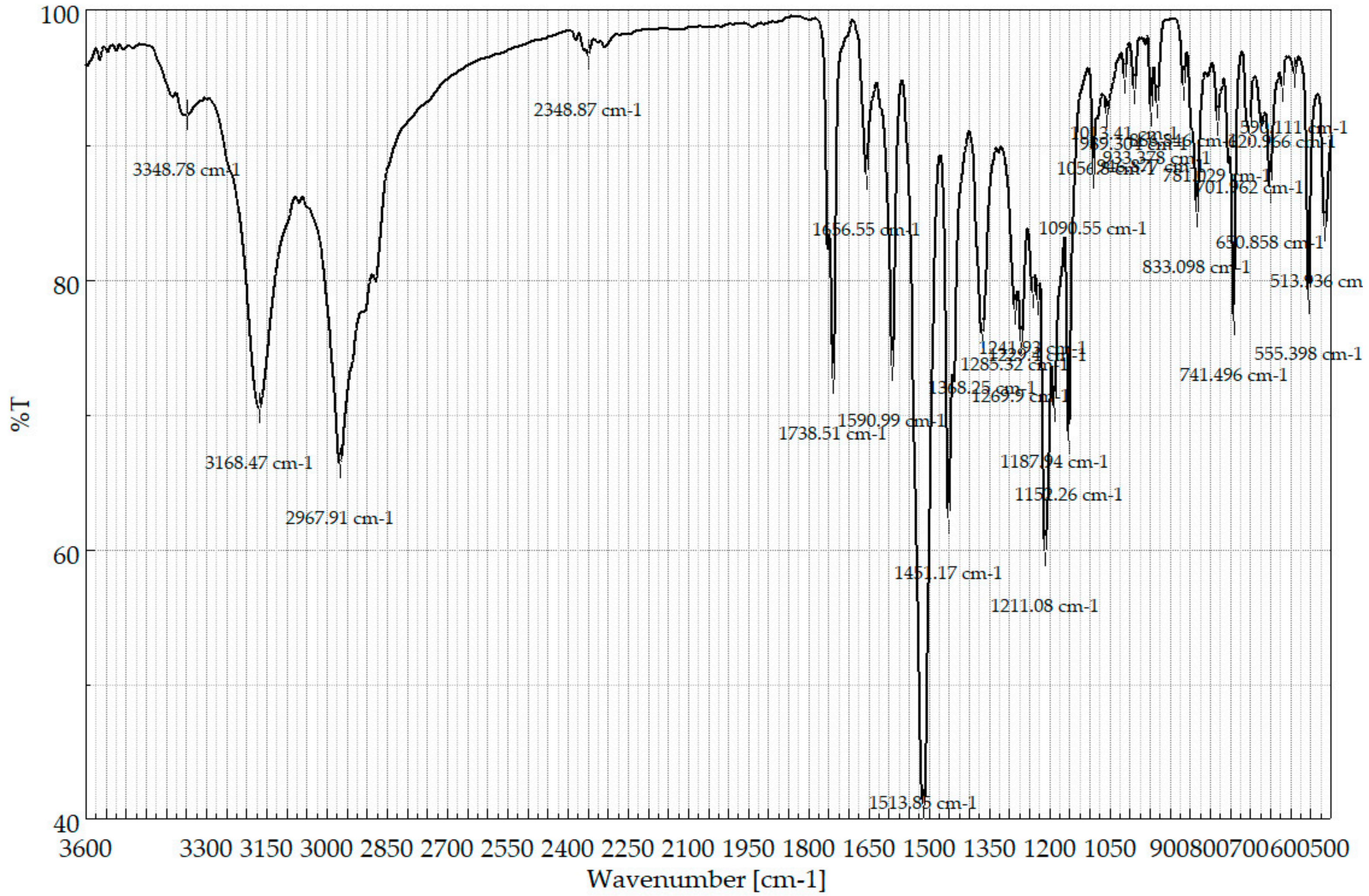

Methyl (2*S*)-3-methyl 2-[(2-{[(3,5-difluorophenyl)carbamothioyl]amino}benzoyl)amino]pentanoate (11f)

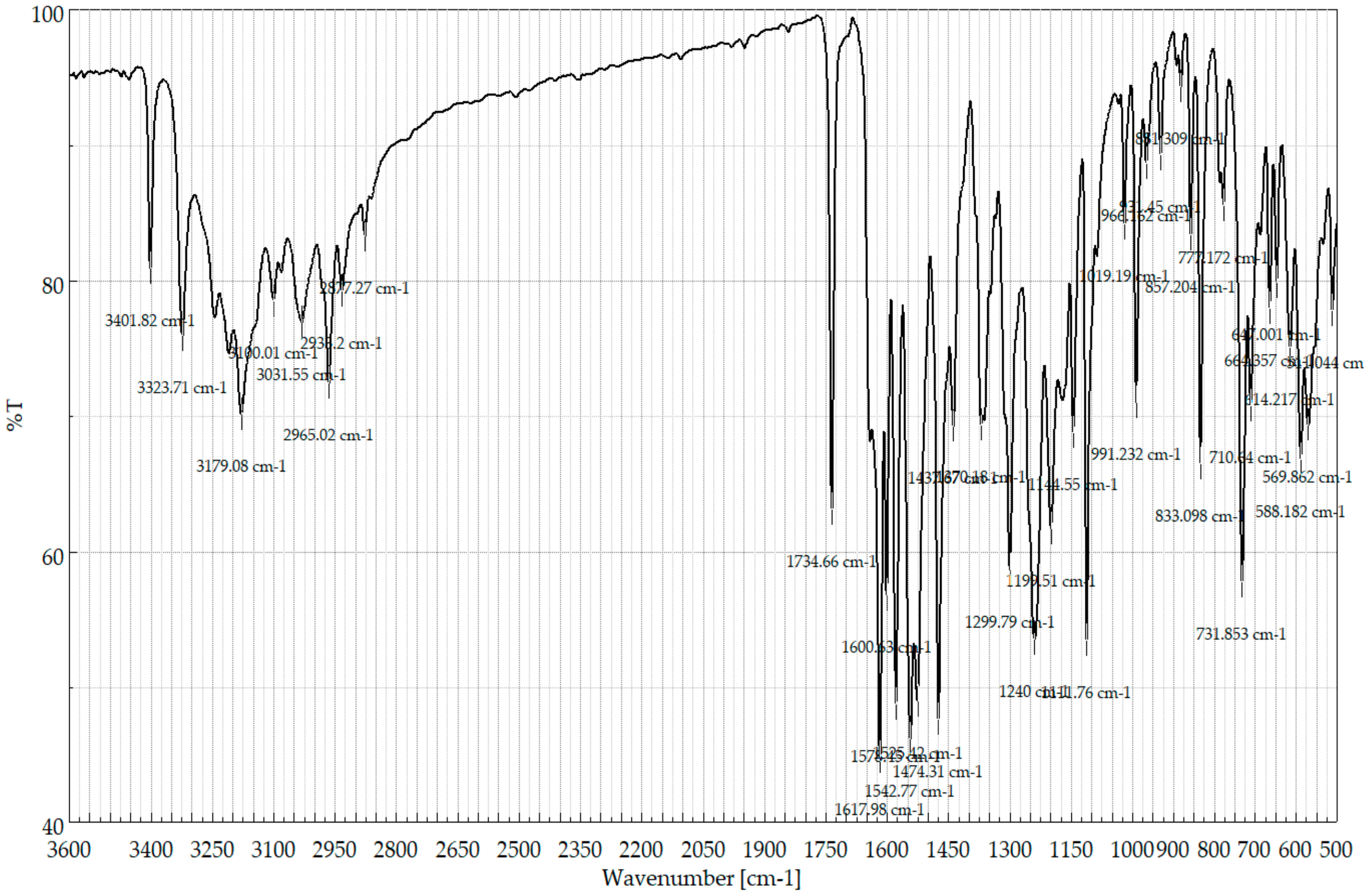

Wavenumber [cm-1]
569,862
614,217
647,001
710,64
731,853
777,172
833,098
857,204
881,309
931,45
966,162
991,232
1111,76
1144,55
1199,51
1299,79
1370,18
1437,67
1474,31
1525,42
1542,77
1578,45
1600,63
1617,98
1734,66
2877,27
2933,2
2965,02
3031,55
3100,01
3179,08
3323,71
3401,82

Wavenumber [cm-1]
759,816
1006,66
1187,94
1292,07
1348
1433,82
1475,28
1517,7
1591,95
1738,51
2348,87
2969,84
3310,21
3623,59
3725,8

Methyl (2*S*)-3-methyl 2-[(2-{[(2,6-difluorophenyl)carbamothioyl]amino}benzoyl)amino]pentanoate (11g)

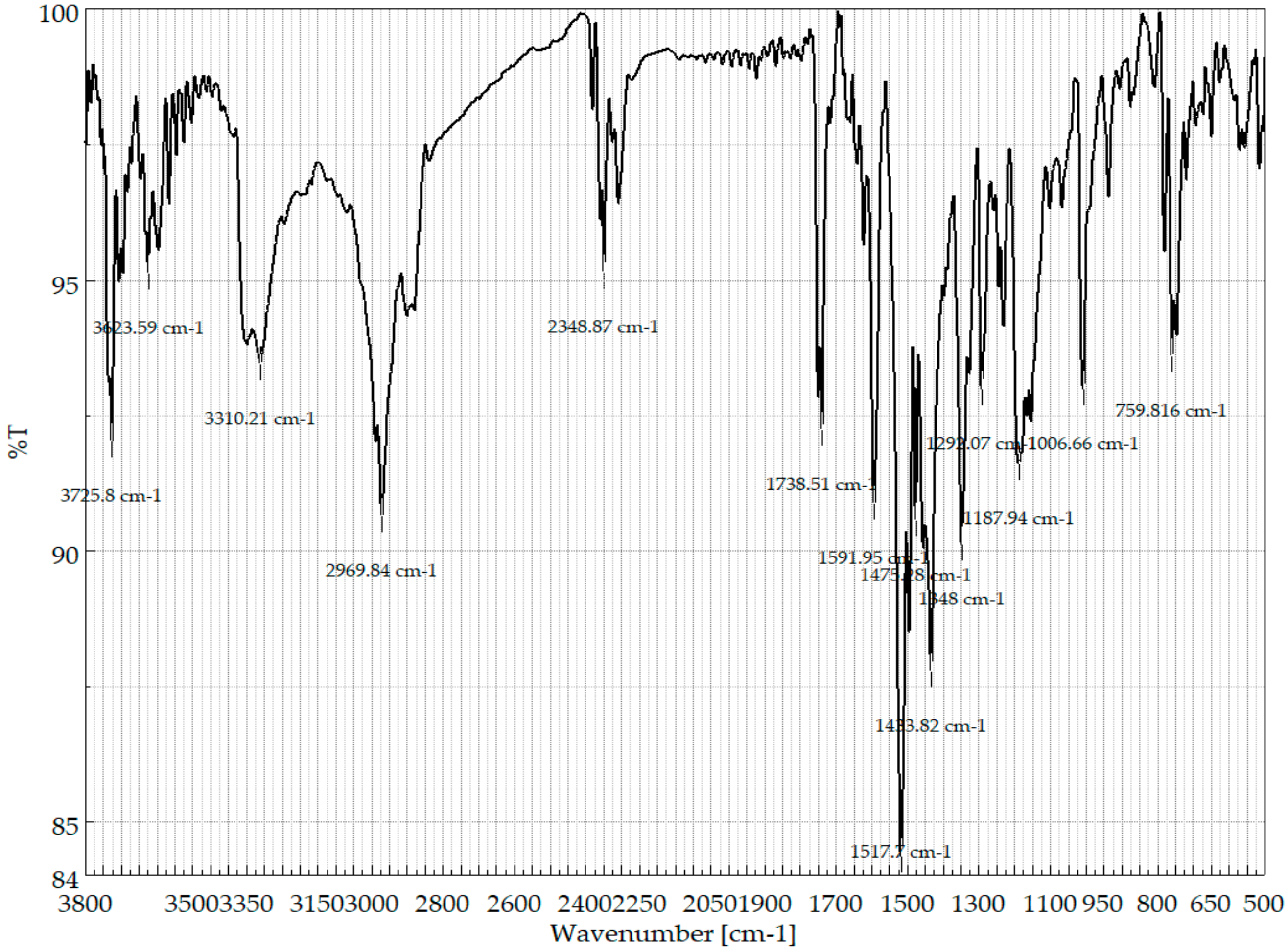

NMR spectra

^{1}H-NMR spectrum of **(2*S*)-2-[(2-aminobenzoyl)amino]-3-methylbutanoic acid (2)**

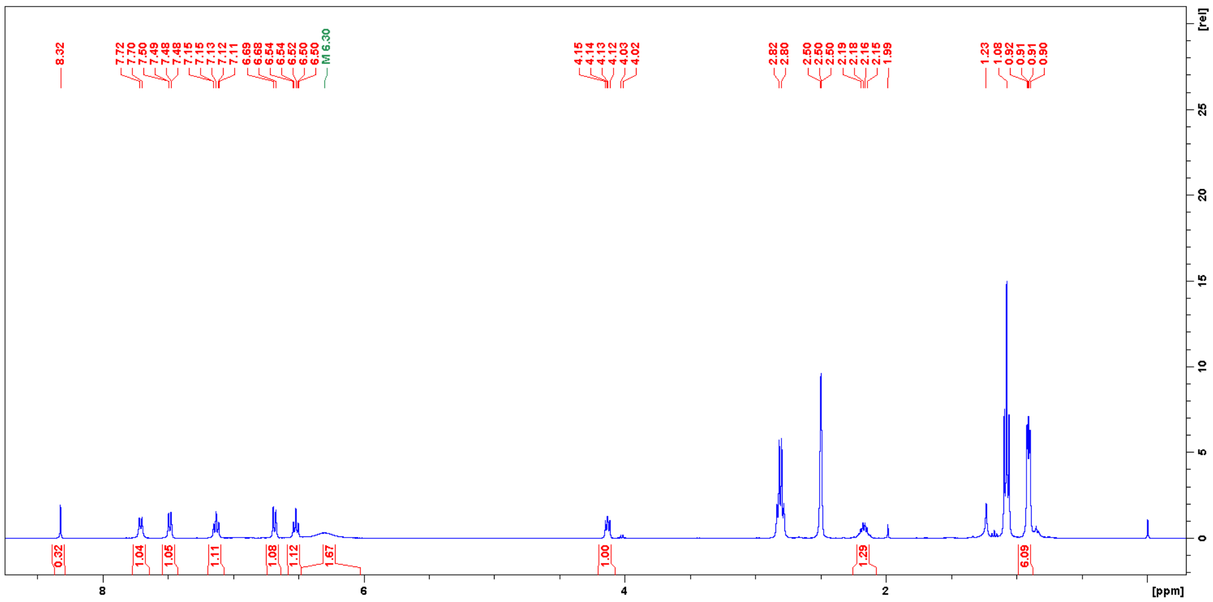

^{13}C-NMR spectrum of **(2*S*)-2-[(2-aminobenzoyl)amino]-3-methylbutanoic acid (2)**

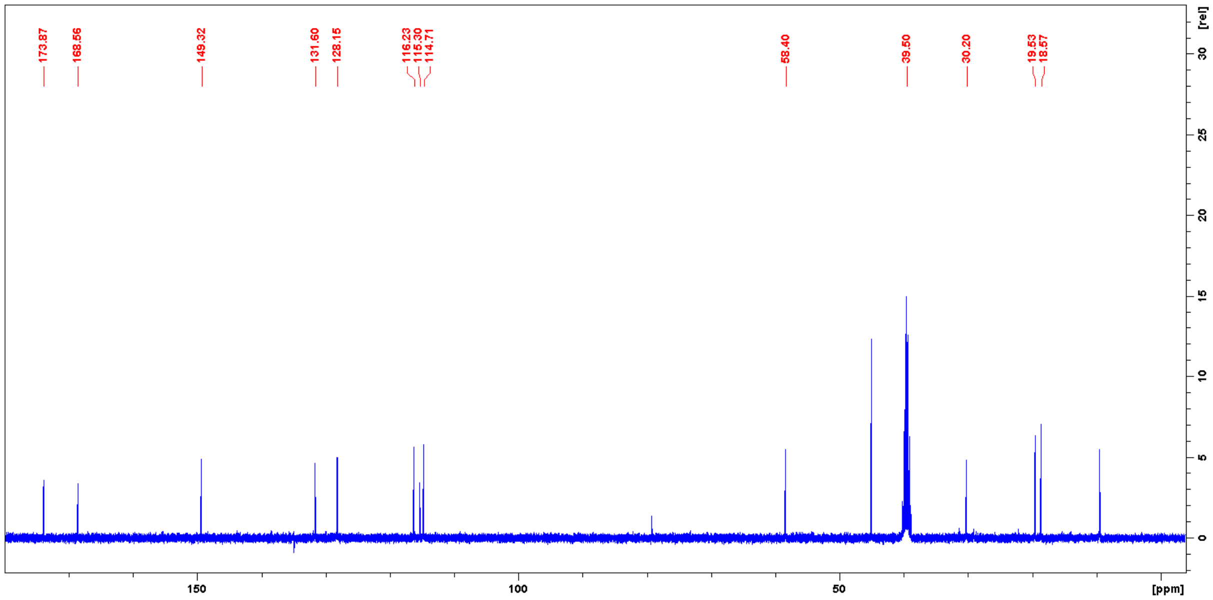

^{1}H-NMR spectrum of **(2*S*)-2-[(2-aminobenzoyl)amino]-4-methylpentanoic acid (3)**

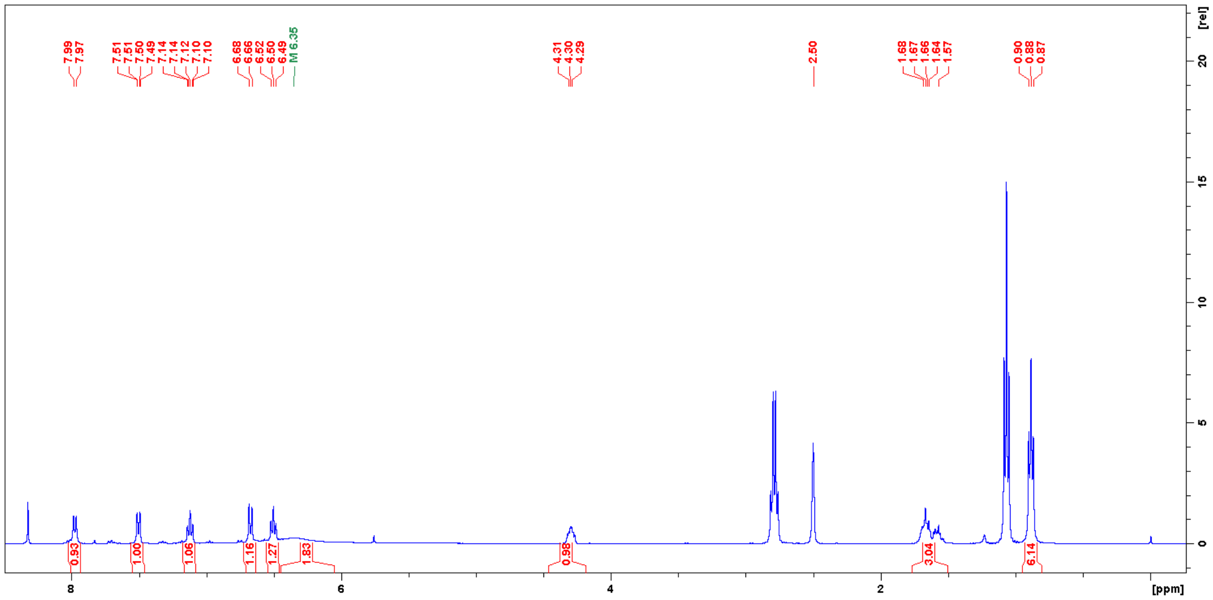

^{13}C-NMR spectrum of **(2*S*)-2-[(2-aminobenzoyl)amino]-4-methylpentanoic acid (3)**

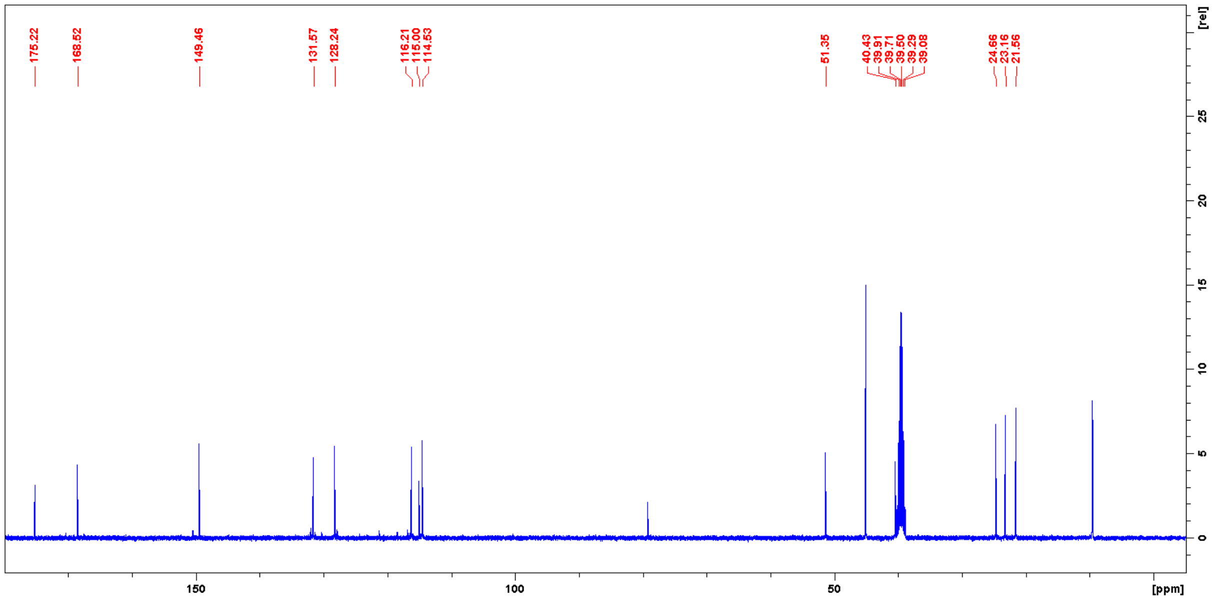

^{1}H-NMR spectrum of **(2*S*)-2-[(2-aminobenzoyl)amino]-3-methylpentanoic acid (4)**

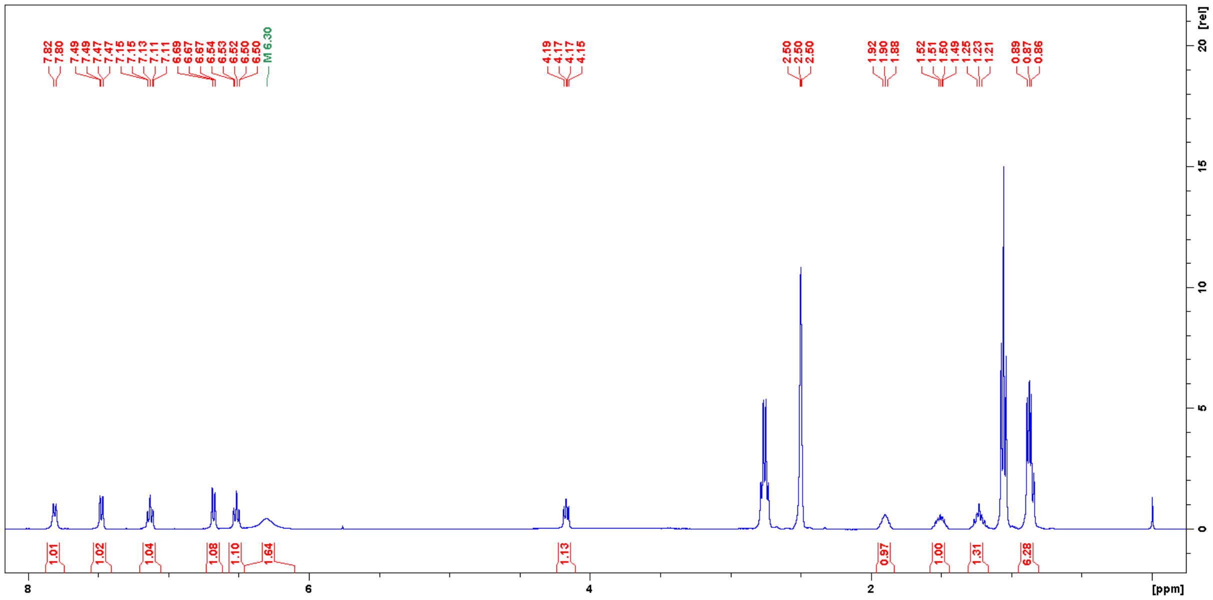

^{13}C-NMR spectrum of **(2*S*)-2-[(2-aminobenzoyl)amino]-3-methylpentanoic acid (4)**

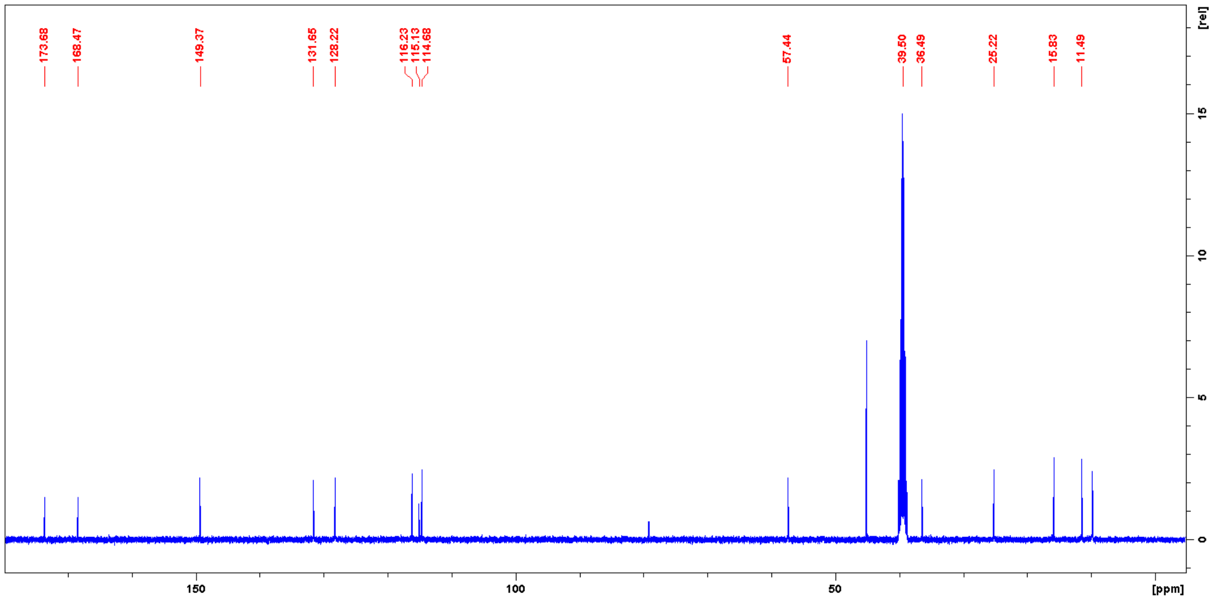

^{1}H-NMR spectrum of **2,3-Dihydro-1*H*-pyrrolo[2,1-*c*][1,4]benzodiazepine-5,11(10*H*,11a*H*)-dione (5)**

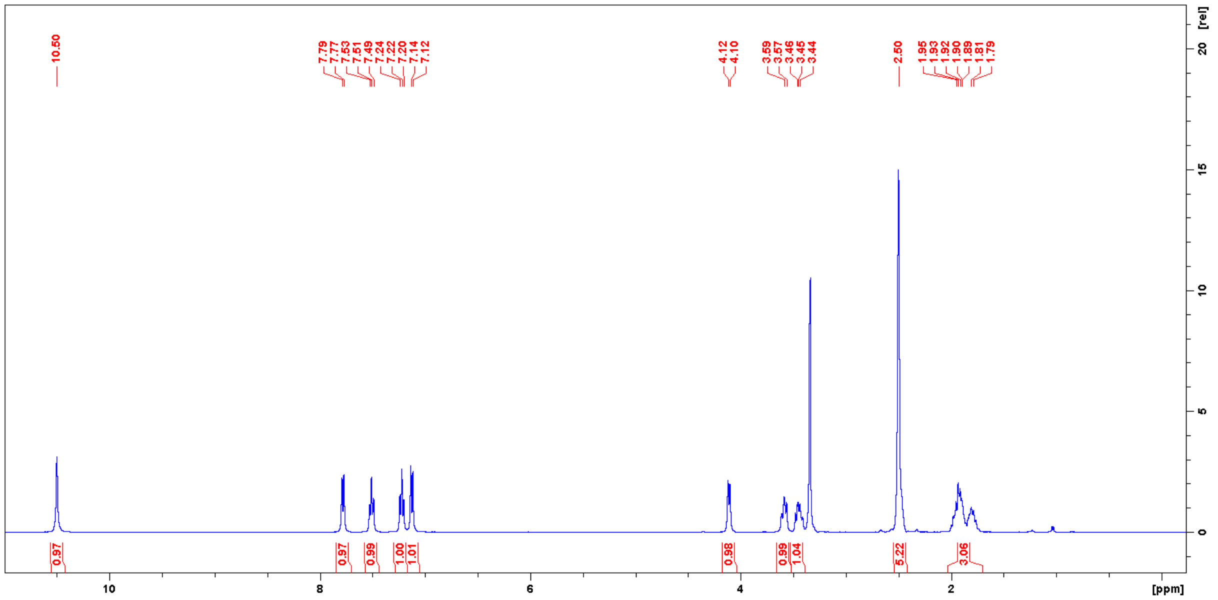

^{13}C-NMR spectrum of **2,3-Dihydro-1*H*-pyrrolo[2,1-*c*][1,4]benzodiazepine-5,11(10*H*,11a*H*)-dione (5)**

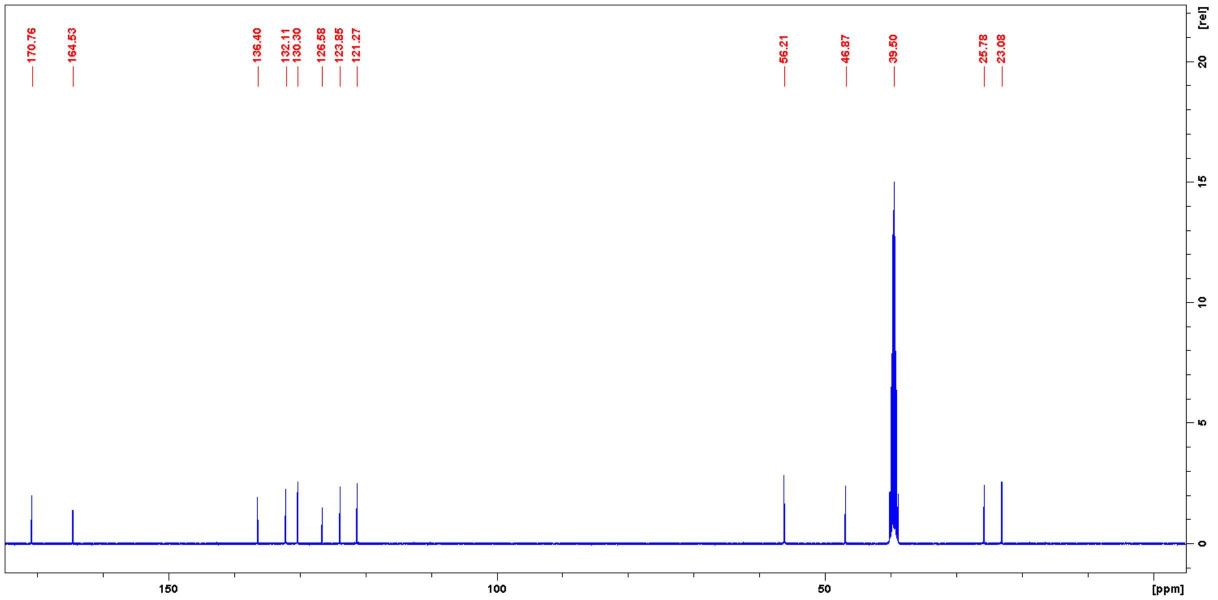

^{1}H-NMR spectrum of **2,3-Dihydro-3-phenyl-2-thioxo-4(1*H*)-quinazolinone (6)**

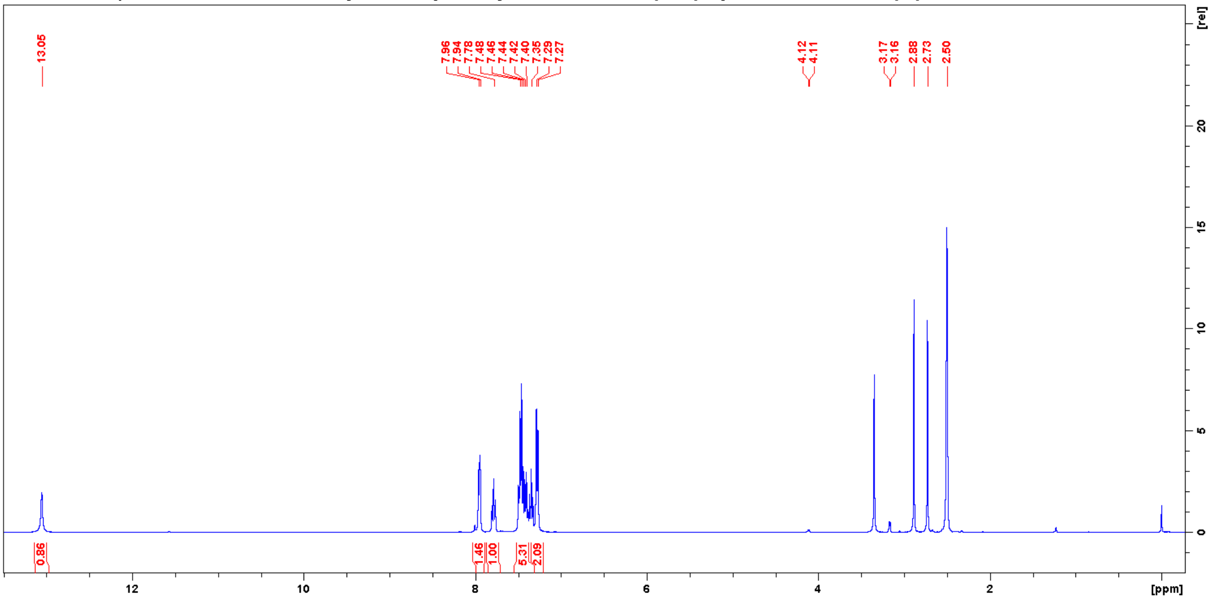

^{13}C-NMR spectrum of **2,3-Dihydro-3-phenyl-2-thioxo-4(1*H*)-quinazolinone (6)**

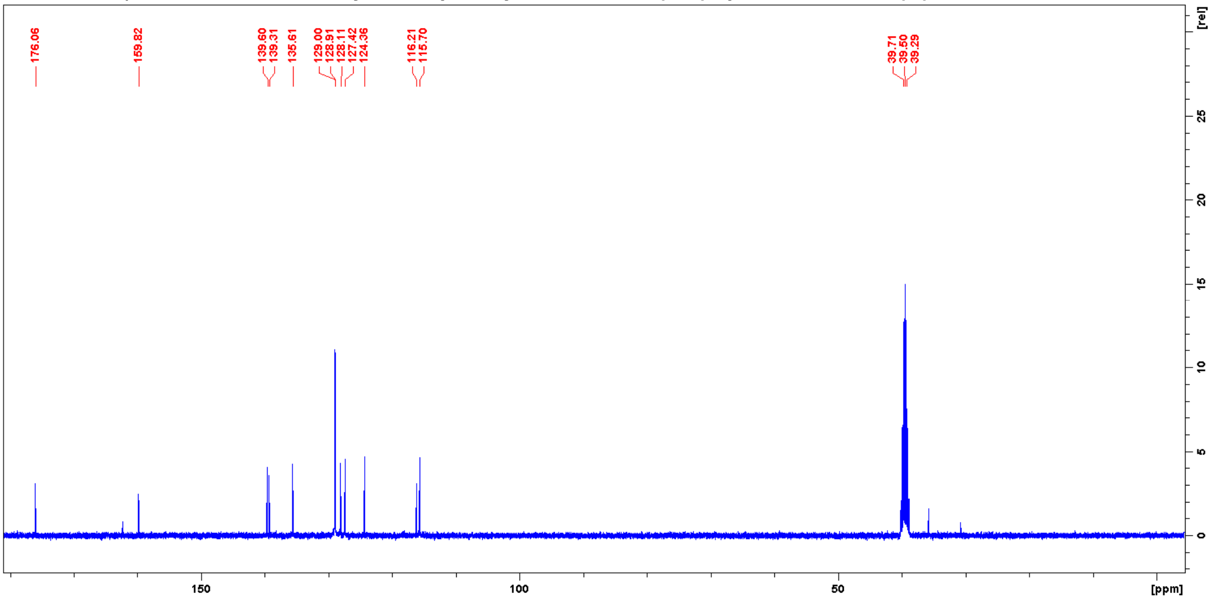

^{1}H-NMR spectrum of **2,3-Dihydro-3(4-nitrophenyl)-2-thioxo-4(1*H*)-quinazolinone (7)**

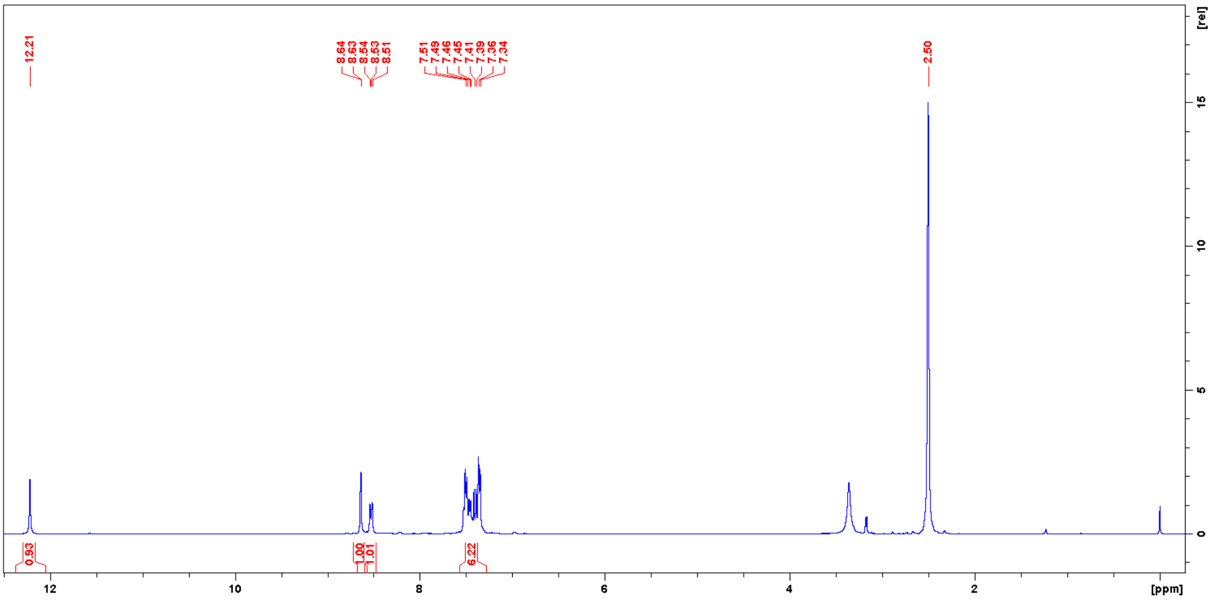

^{13}C-NMR spectrum of **2,3-Dihydro-3(4-nitrophenyl)-2-thioxo-4(1*H*)-quinazolinone (7)**

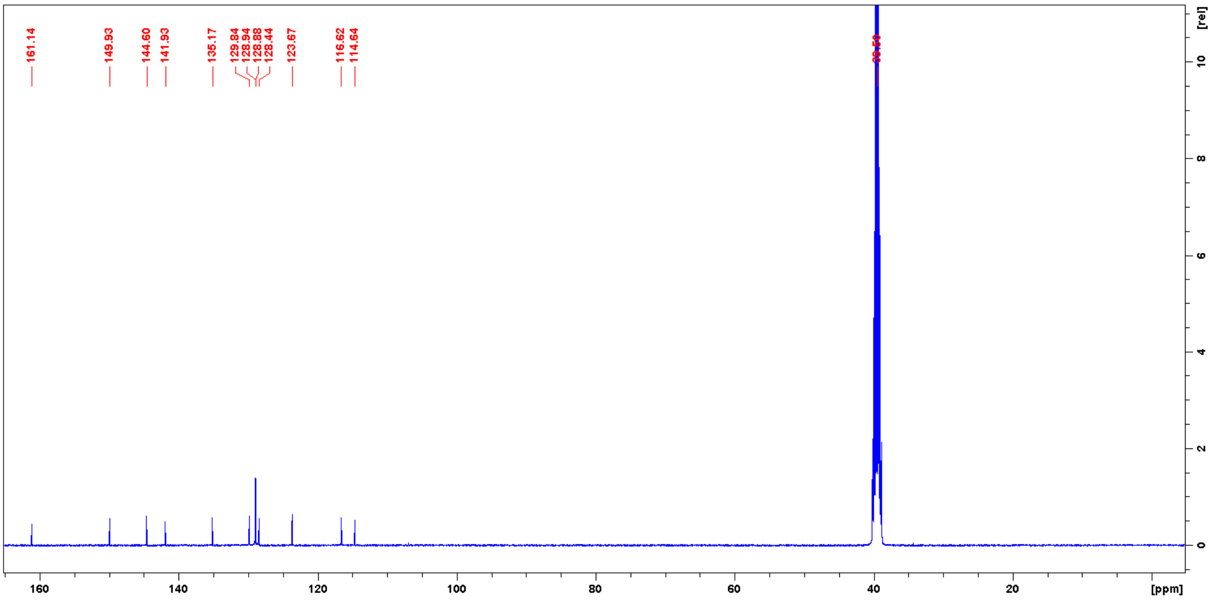

^{1}H-NMR spectrum of**2,3-Dihydro-3-[3,5-bis(trifluoromethyl)phenyl]-2-thioxo-4(1*H*)-quinazolinone (8)**

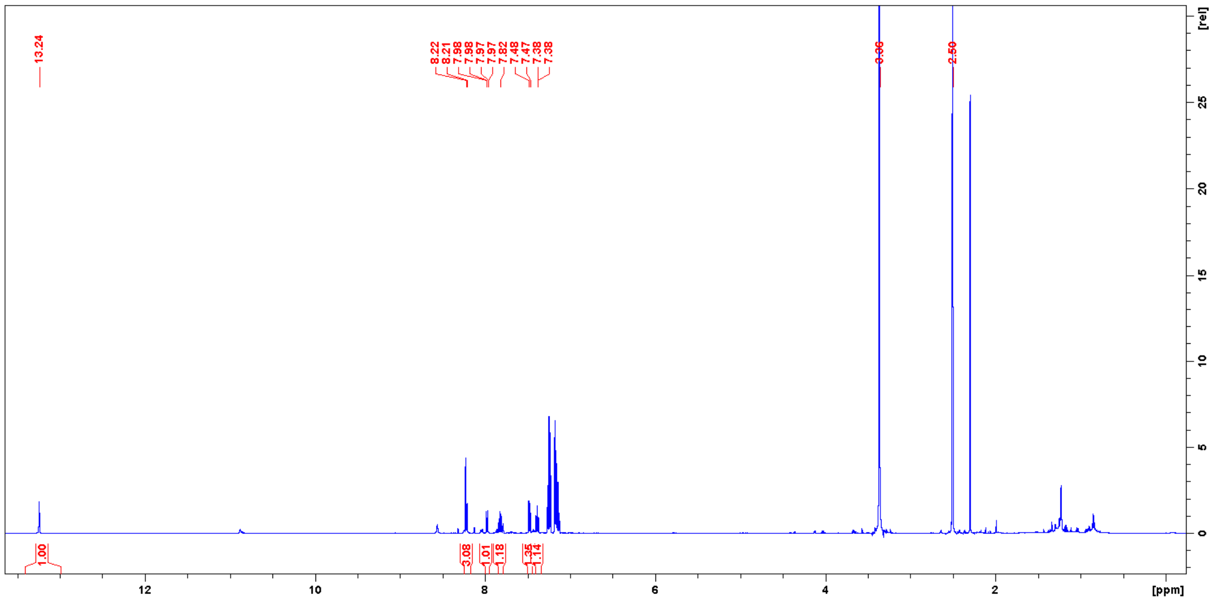

^{13}C-NMR spectrum of **2,3-Dihydro-3-[3,5-bis(trifluoromethyl)phenyl]-2-thioxo-4(1*H*)-quinazolinone (8)**

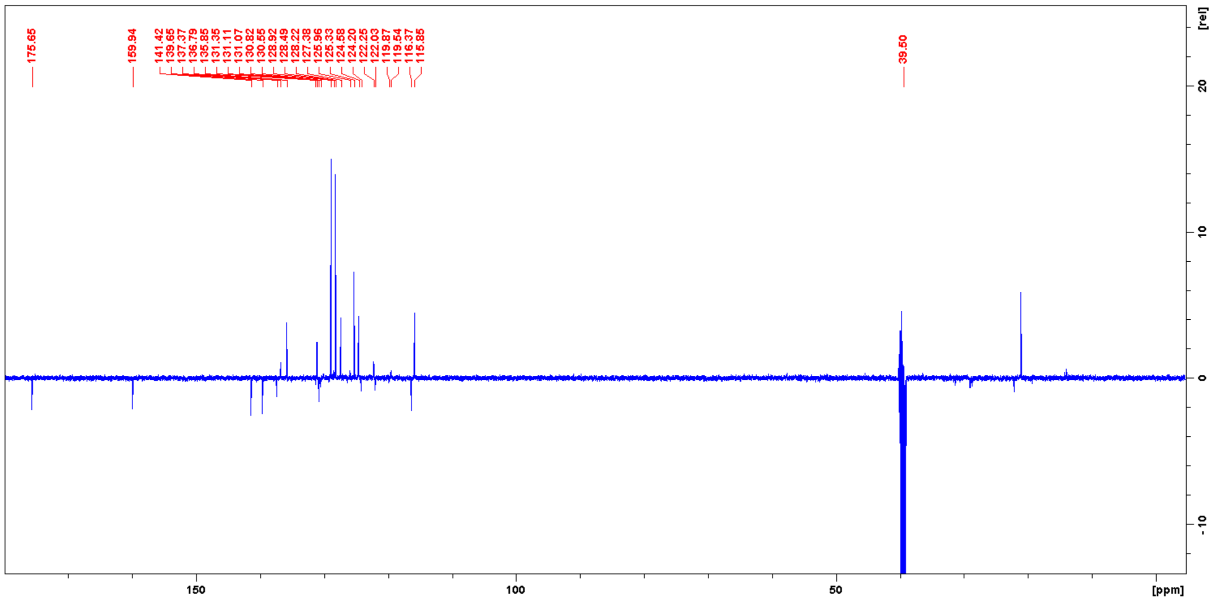

^{1}H-NMR spectrum of **methyl (2*S*)-2-[(2-aminobenzoyl)amino]-3-methylbutanoate (9)**

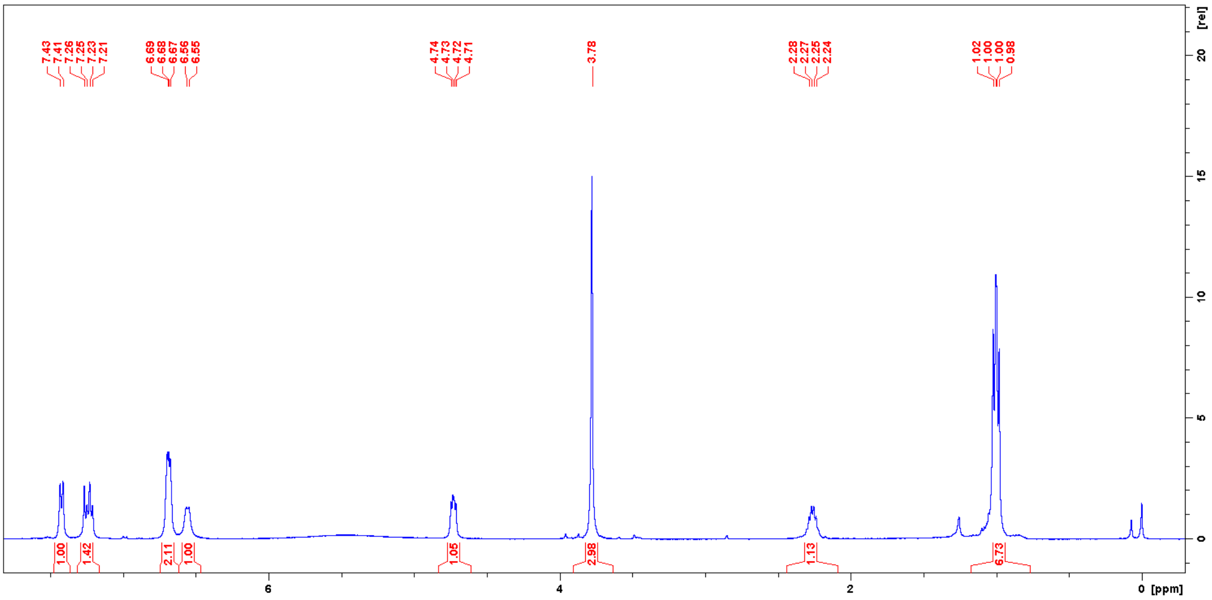

^{13}C-NMR spectrum of **methyl (2*S*)-2-[(2-aminobenzoyl)amino]-3-methylbutanoate (9)**

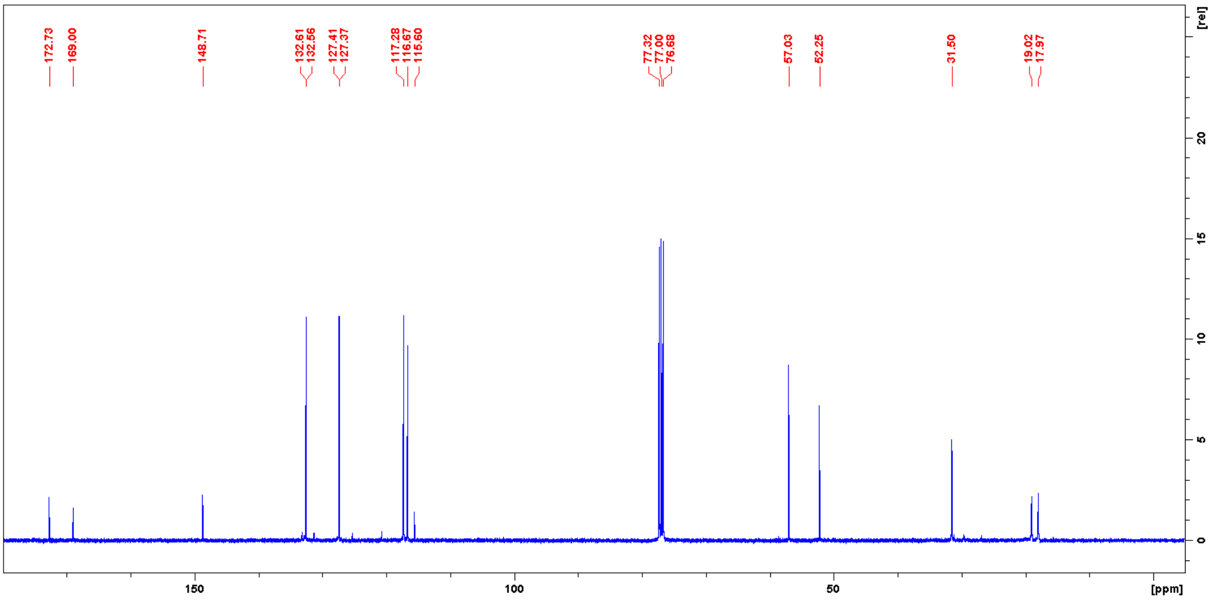

^{1}H-NMR spectrum of **methyl (2*S*)-2-[(2-aminobenzoyl)amino]-4-methylpentanoate (10)**

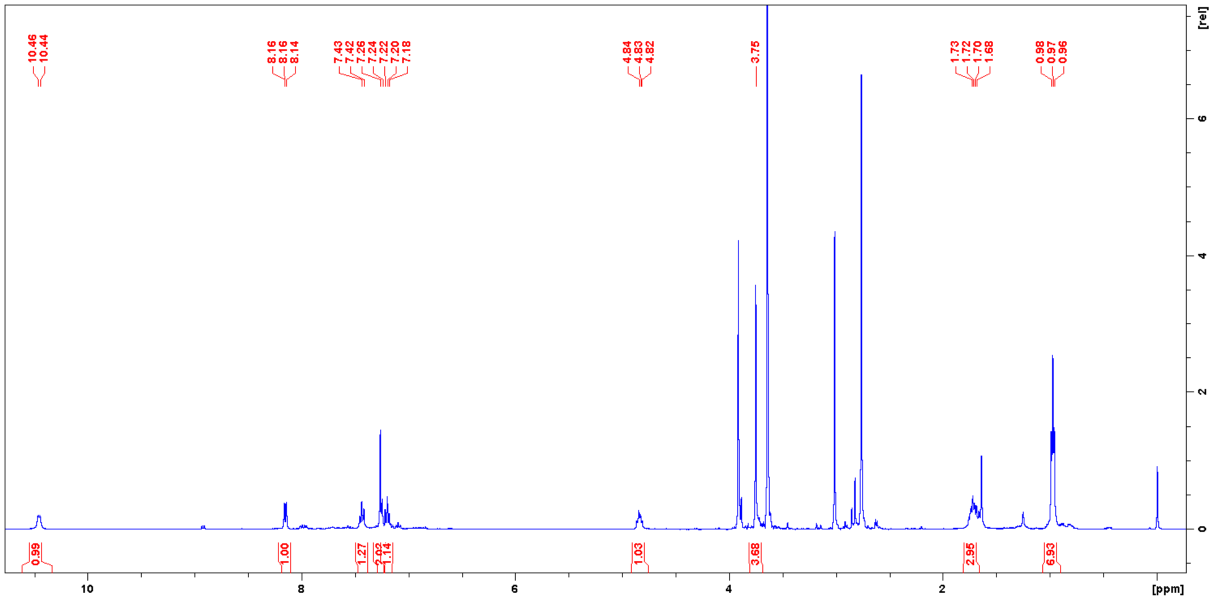

^{13}C-NMR spectrum of **methyl (2*S*)-2-[(2-aminobenzoyl)amino]-4-methylpentanoate (10)**

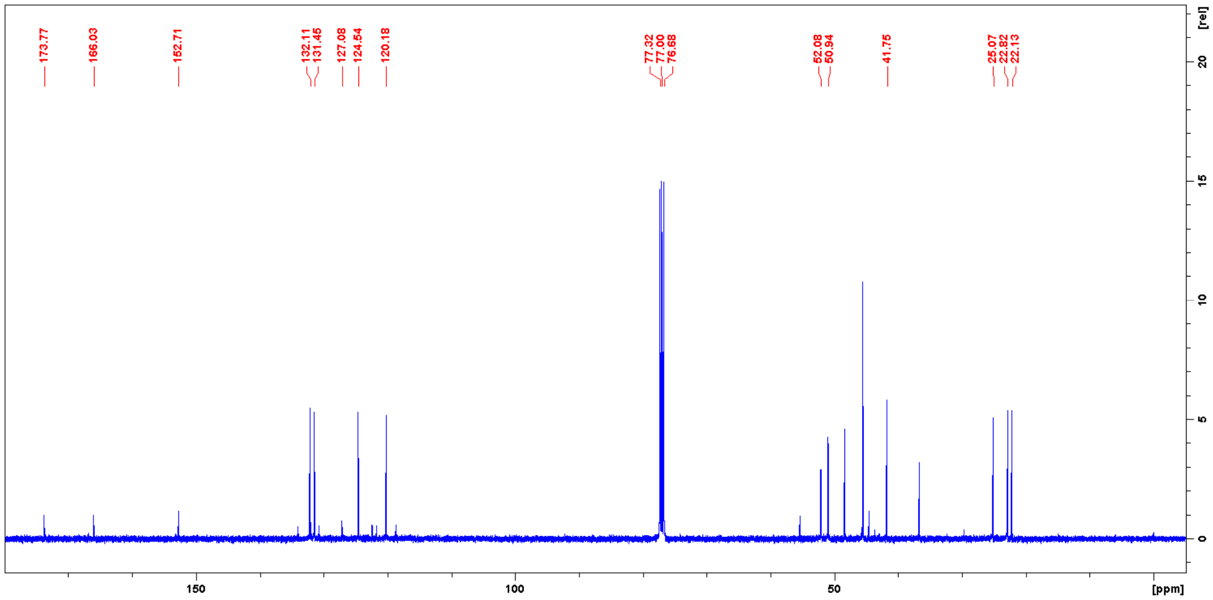

^{1}H-NMR spectrum of **methyl (2*S*)-2-[(2-aminobenzoyl)amino]-3-methylpentanoate (11)**

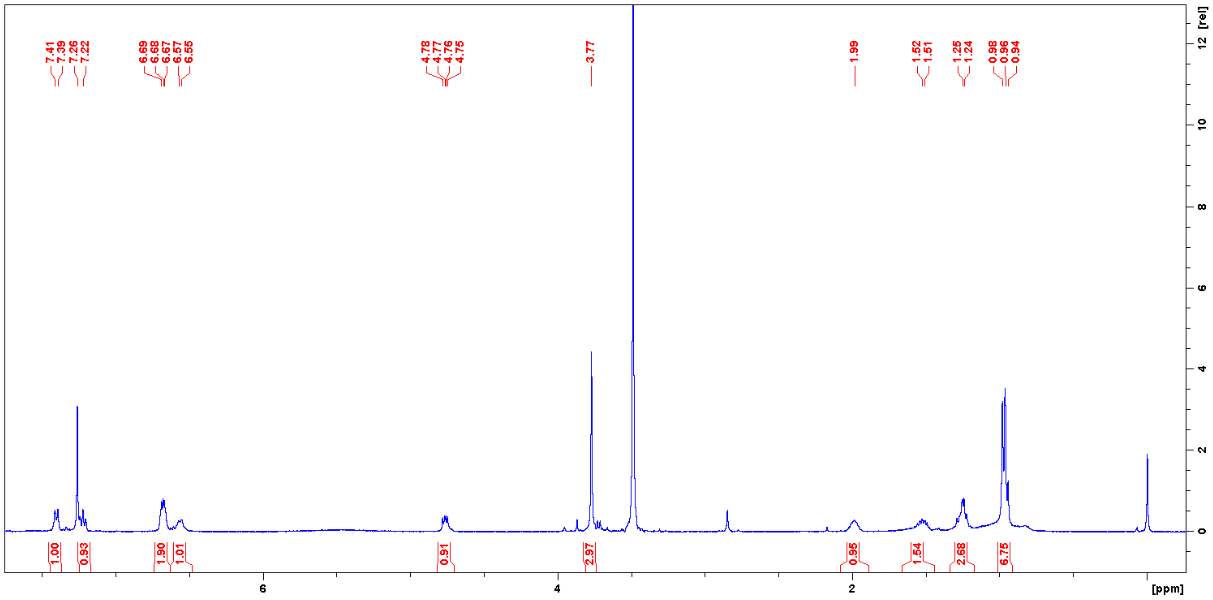

^{13}C-NMR spectrum of **methyl (2*S*)-2-[(2-aminobenzoyl)amino]-3-methylpentanoate (11)**

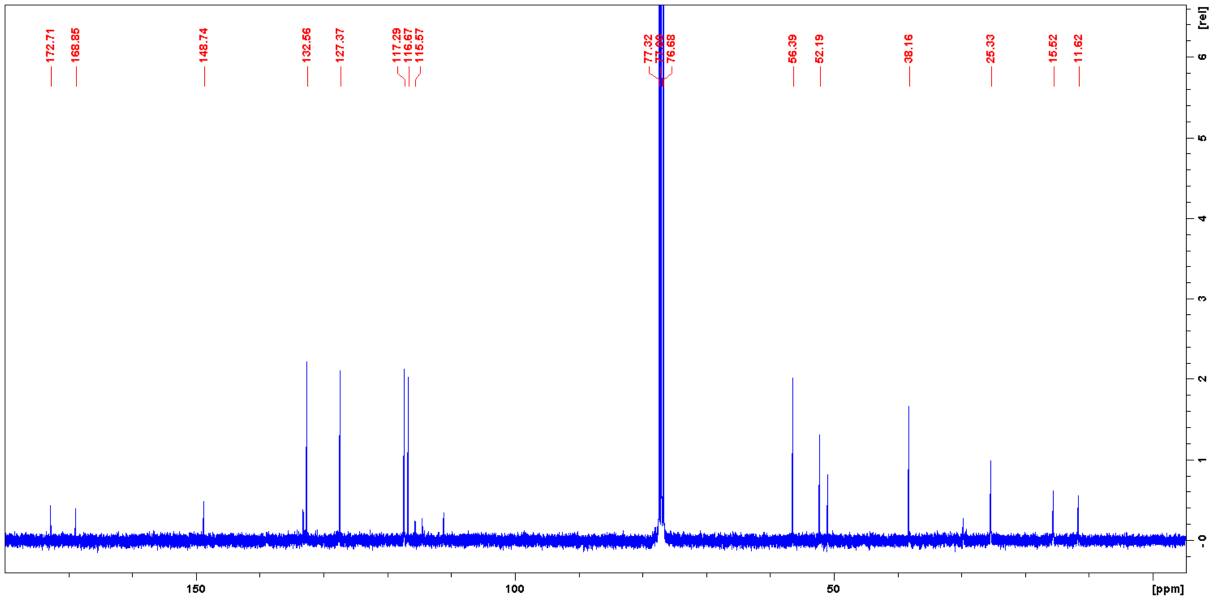

^{1}H-NMR spectrum of **methyl (2*S*)-3-methyl-2-({2-[(phenylcarbamothioyl)amino]benzoyl}amino)butanoate (9a)**

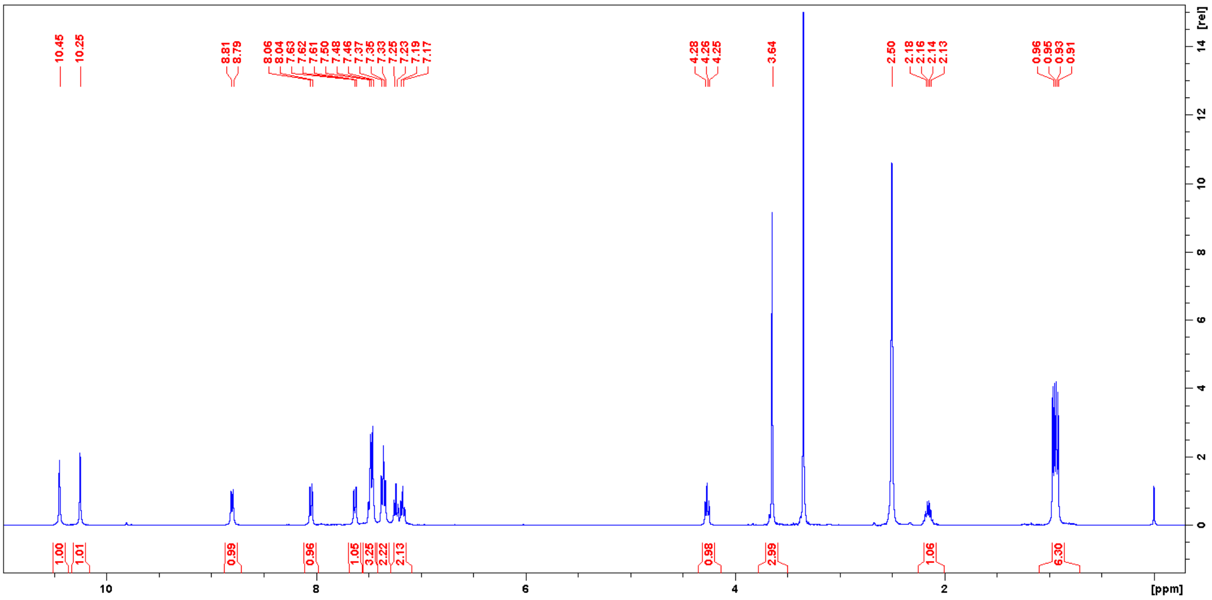

^{13}C-NMR spectrum of **methyl (2*S*)-3-methyl-2-({2-[(phenylcarbamothioyl)amino]benzoyl}amino)butanoate (9a)**

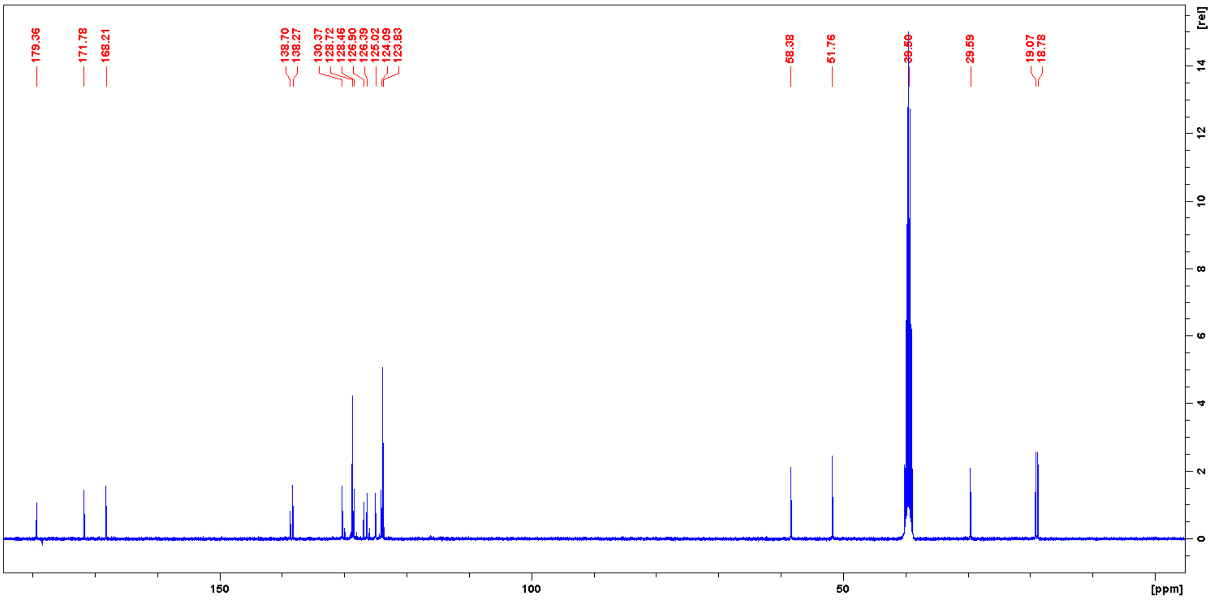

^{1}H-NMR spectrum of **methyl (2*S*)-3-methyl 2-[(2-{[(4-nitrophenyl)carbamothioyl]amino}benzoyl)amino]butanoate (9b)**

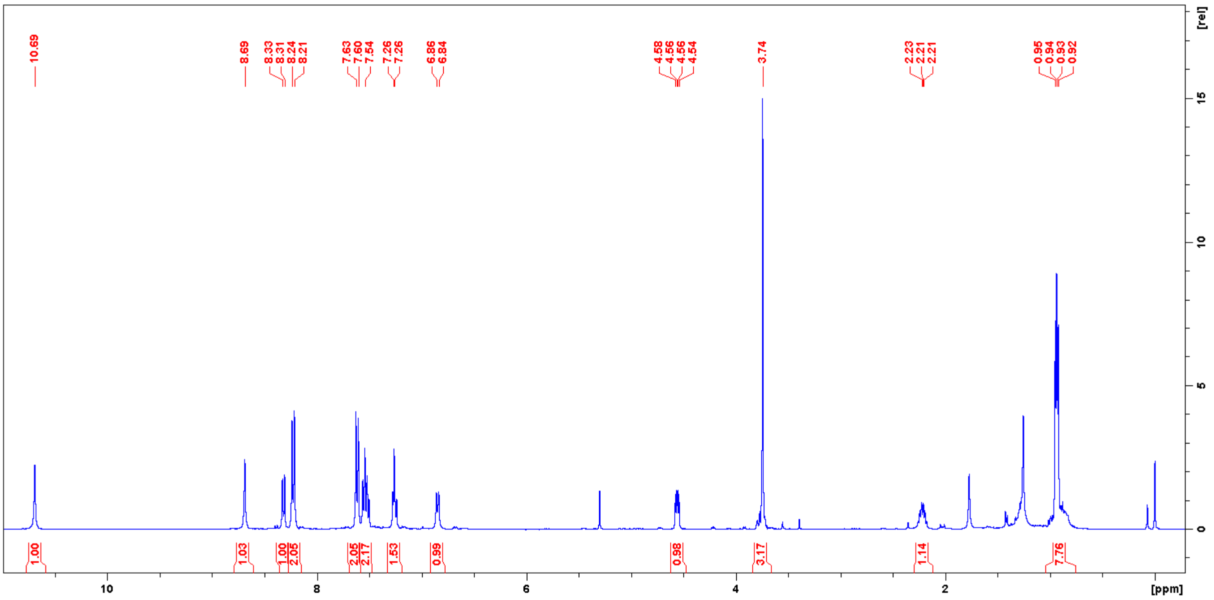

^{13}C-NMR spectrum of **methyl (2*S*)-3-methyl 2-[(2-{[(4-nitrophenyl)carbamothioyl]amino}benzoyl)amino]butanoate (9b)**

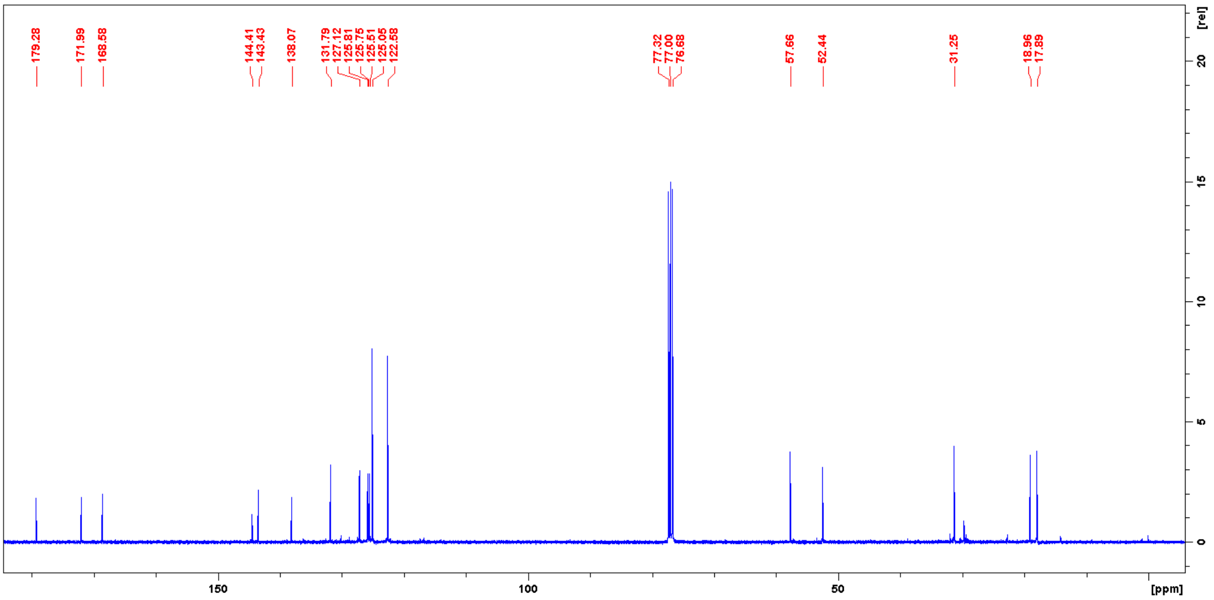

^{1}H-NMR spectrum of **methyl (2*S*)-3-methyl 2-[(2-{[(3,5-bistrifluoromethylphenyl)carbamothioyl]amino} benzoyl)amino]butanoate (9c)**

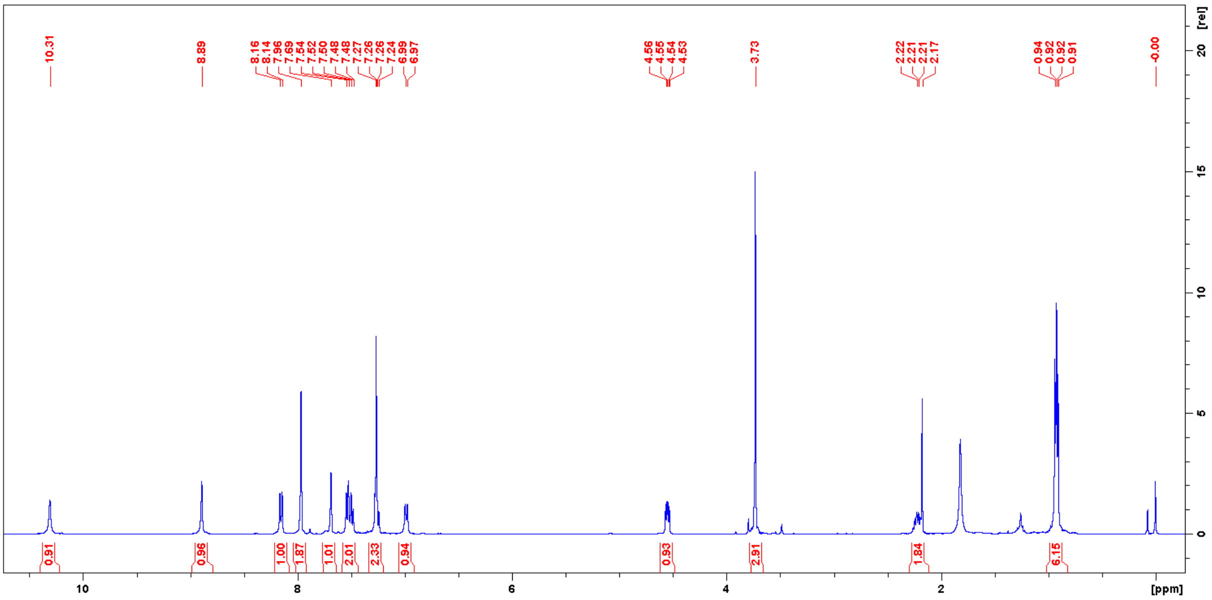

^{13}C-NMR spectrum of **methyl (2*S*)-3-methyl 2-[(2-{[(3,5-bistrifluoromethylphenyl)carbamothioyl]amino} benzoyl)amino]butanoate (9c)**

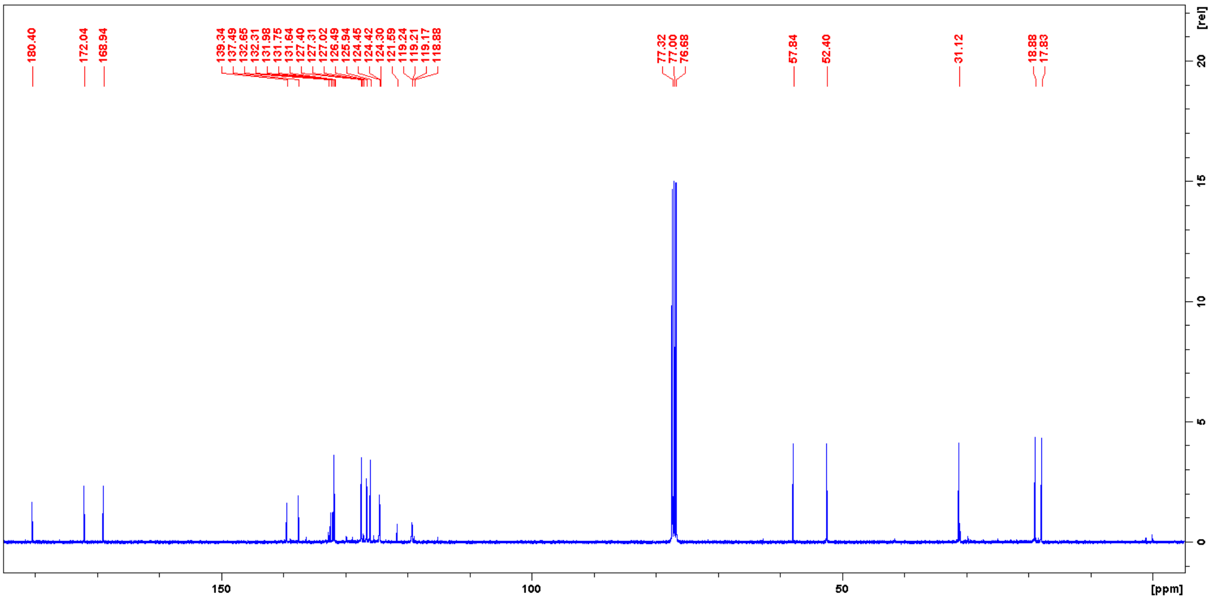

^{1}H-NMR spectrum of **methyl (2*S*)-3-methyl 2-[(2-{[(4-methoxyphenyl)carbamothioyl]amino}benzoyl)amino]butanoate (9d)**

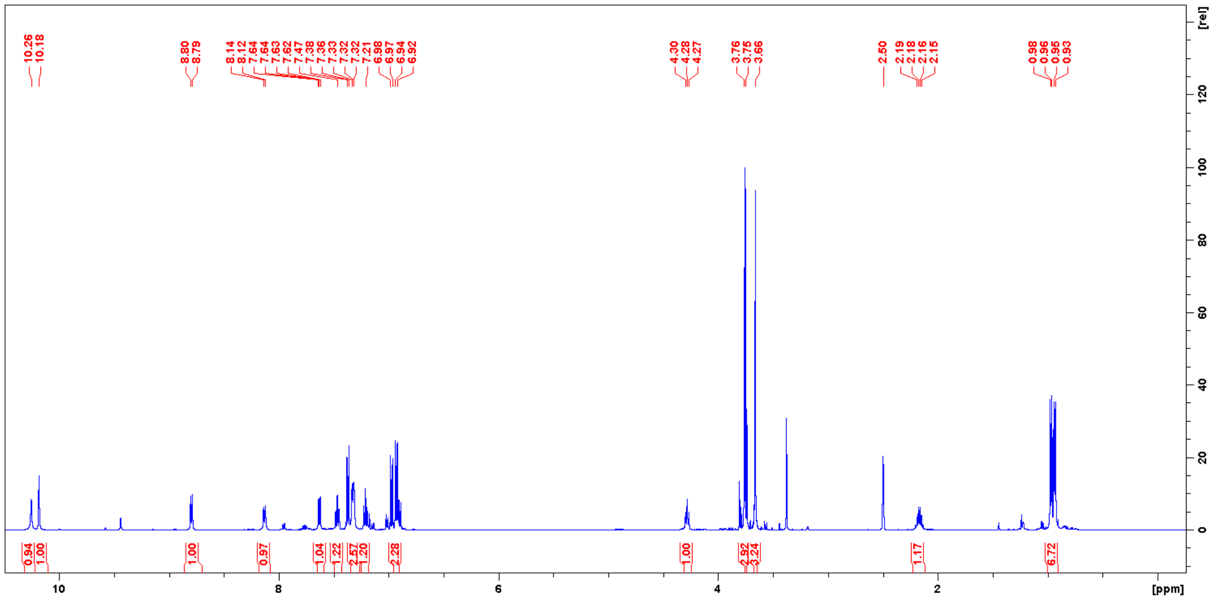

^{13}C-NMR spectrum of **methyl (2*S*)-3-methyl 2-[(2-{[(4-methoxyphenyl)carbamothioyl]amino}benzoyl)amino]butanoate (9d)**

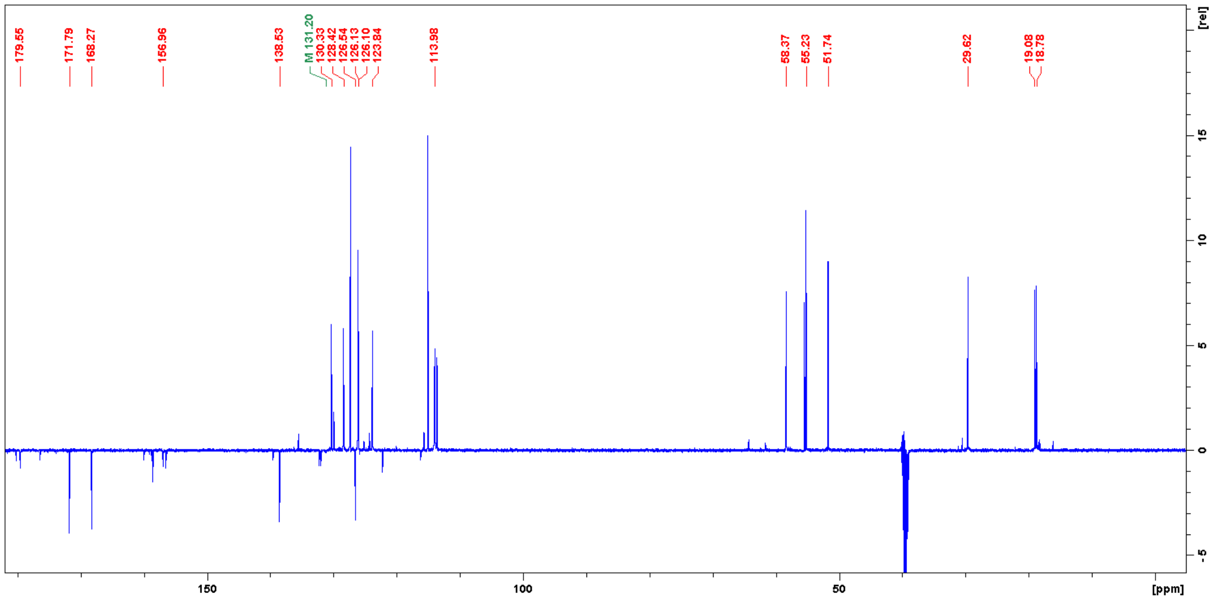

^{1}H-NMR spectrum of **Methyl (2*S*)-3-methyl 2-[(2-{[(4-fluorophenyl)carbamothioyl]amino}benzoyl)amino]butanoate (9e)**

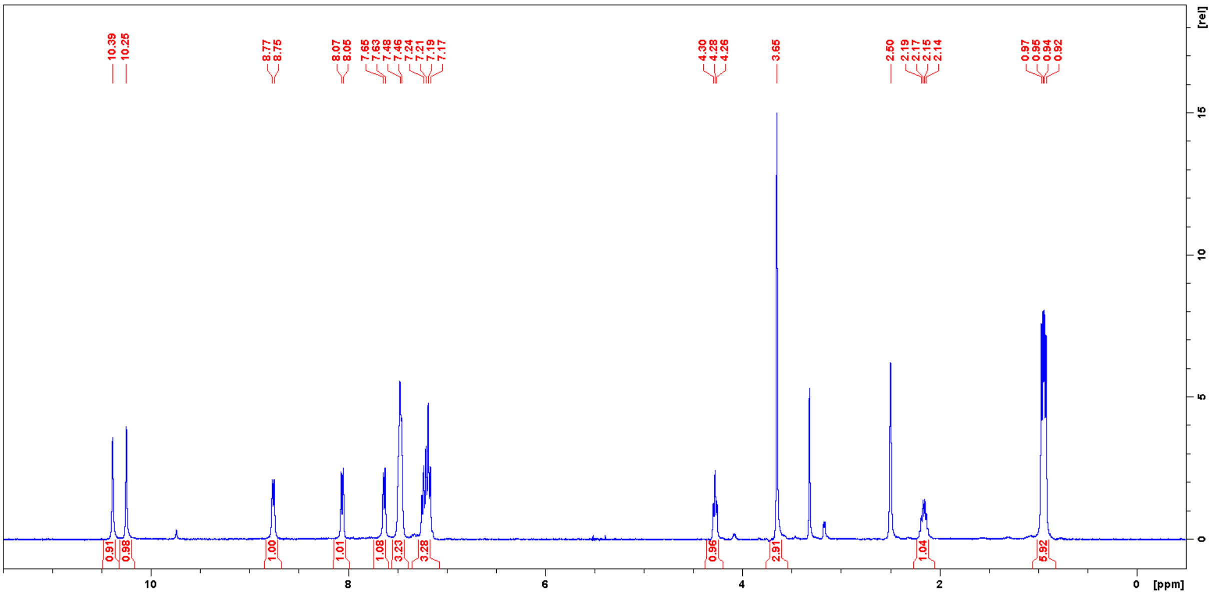

^{13}C-NMR spectrum of **Methyl (2*S*)-3-methyl 2-[(2-{[(4-fluorophenyl)carbamothioyl]amino}benzoyl)amino]butanoate (9e)**

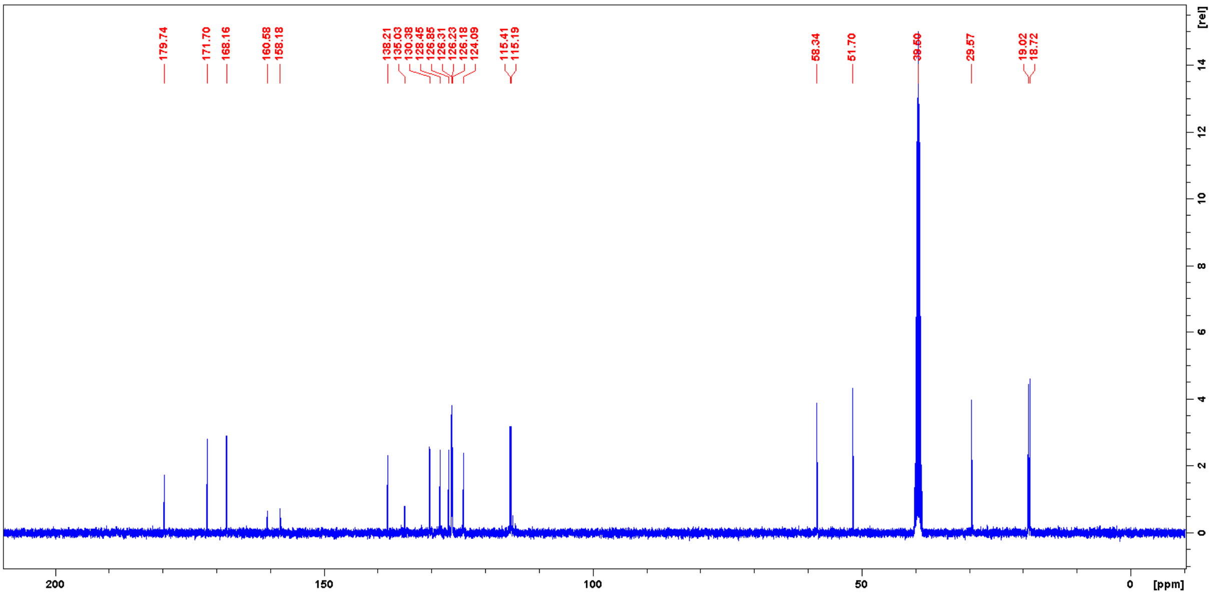

^{1}H-NMR spectrum of **Methyl (2*S*)-3-methyl 2-[(2-{[(3,5-difluorophenyl)carbamothioyl]amino}benzoyl)amino]butanoate (9f)**

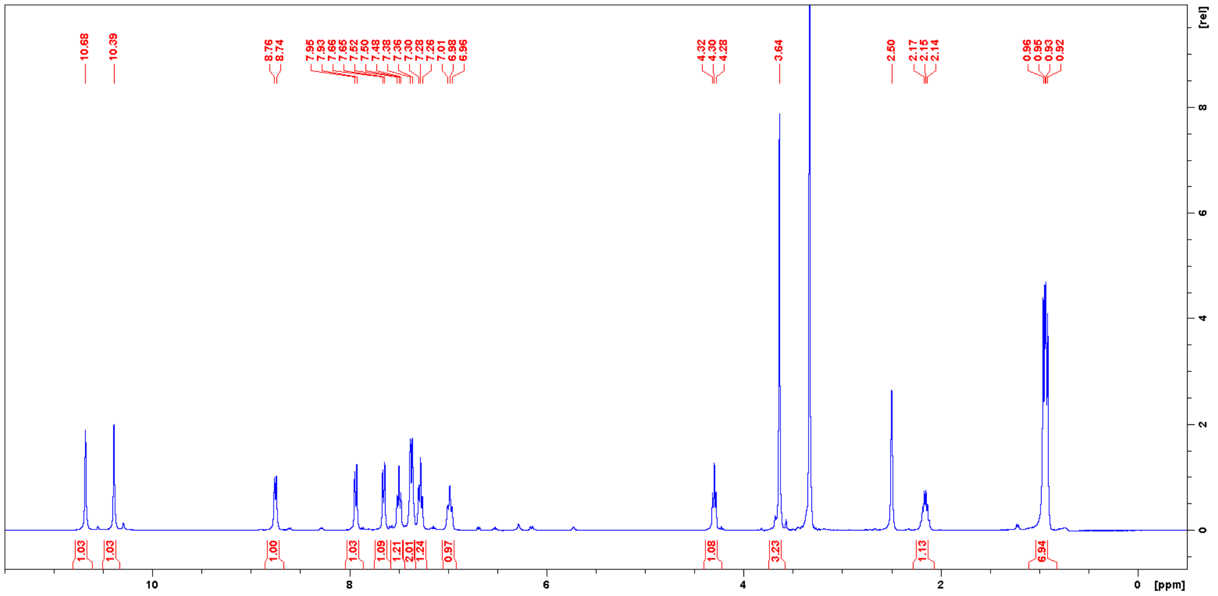

^{13}C-NMR spectrum of **Methyl (2*S*)-3-methyl 2-[(2-{[(3,5-difluorophenyl)carbamothioyl]amino}benzoyl)amino]butanoate (9f)**

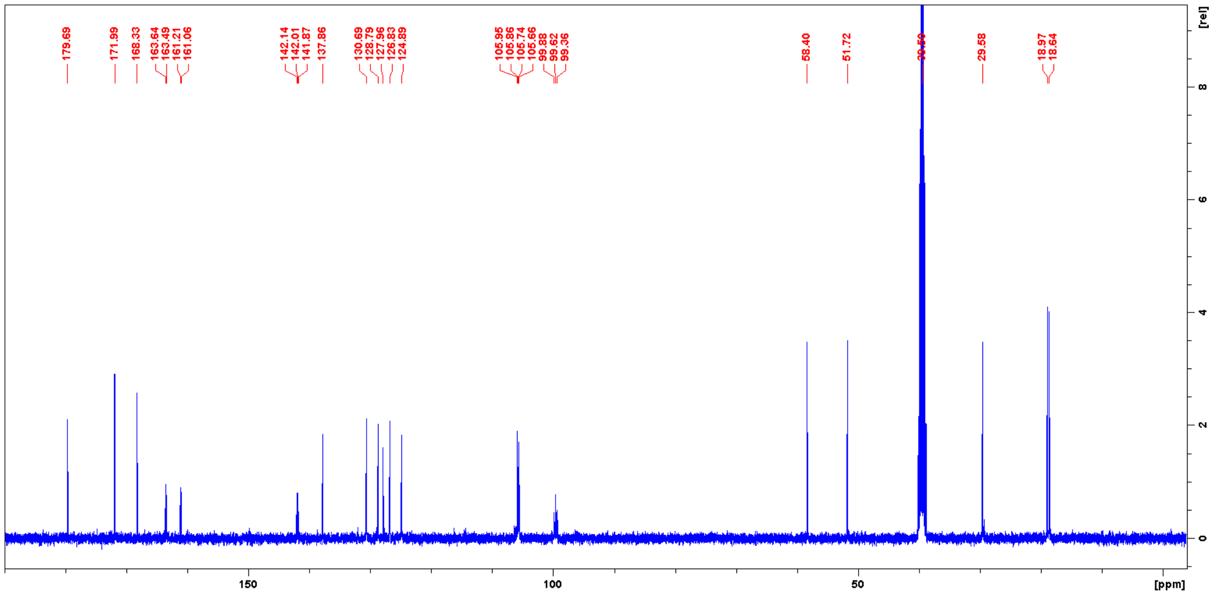

^{1}H-NMR spectrum of **Methyl (2*S*)-3-methyl 2-[(2-{[(2,6-difluorophenyl)carbamothioyl]amino}benzoyl)amino]butanoate (9g)**

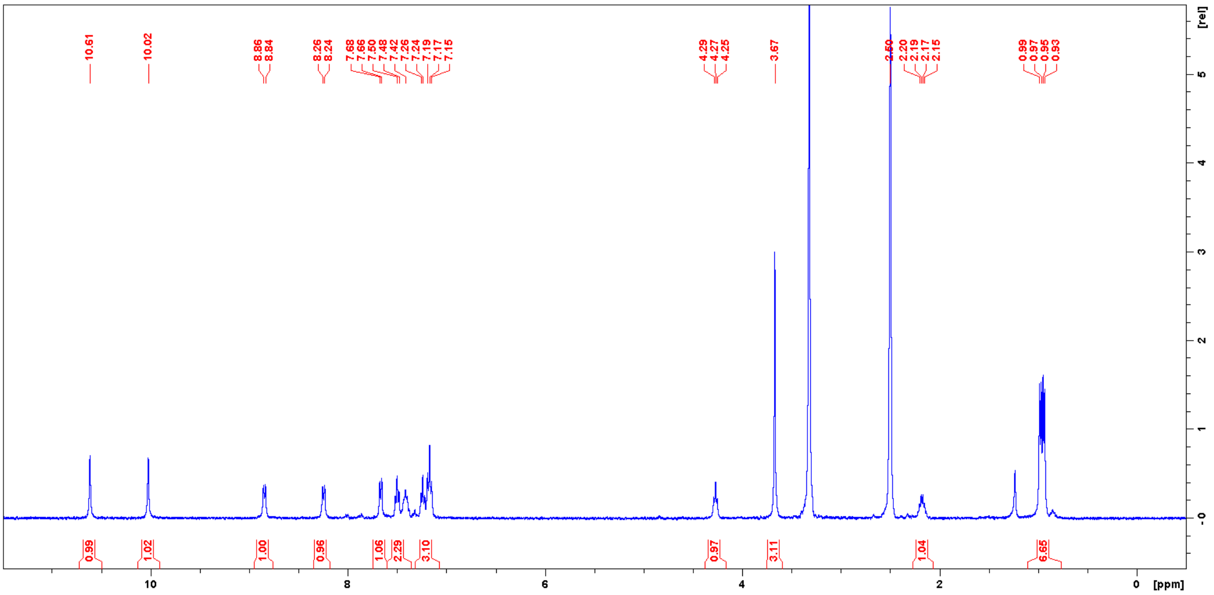

^{13}C-NMR spectrum of **Methyl (2*S*)-3-methyl 2-[(2-{[(2,6-difluorophenyl)carbamothioyl]amino}benzoyl)amino]butanoate (9g)**

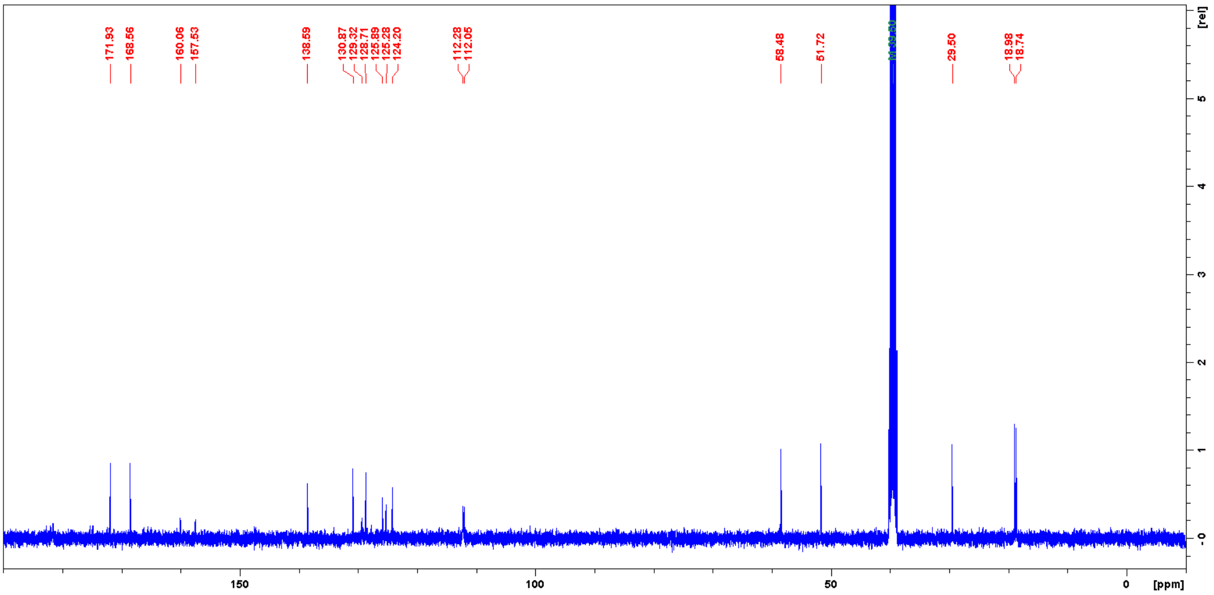

^{1}H-NMR spectrum of **methyl (2*S*)-4-methyl 2-({2-[(phenylcarbamothioyl)amino]benzoyl}amino)pentanoate (10a)**

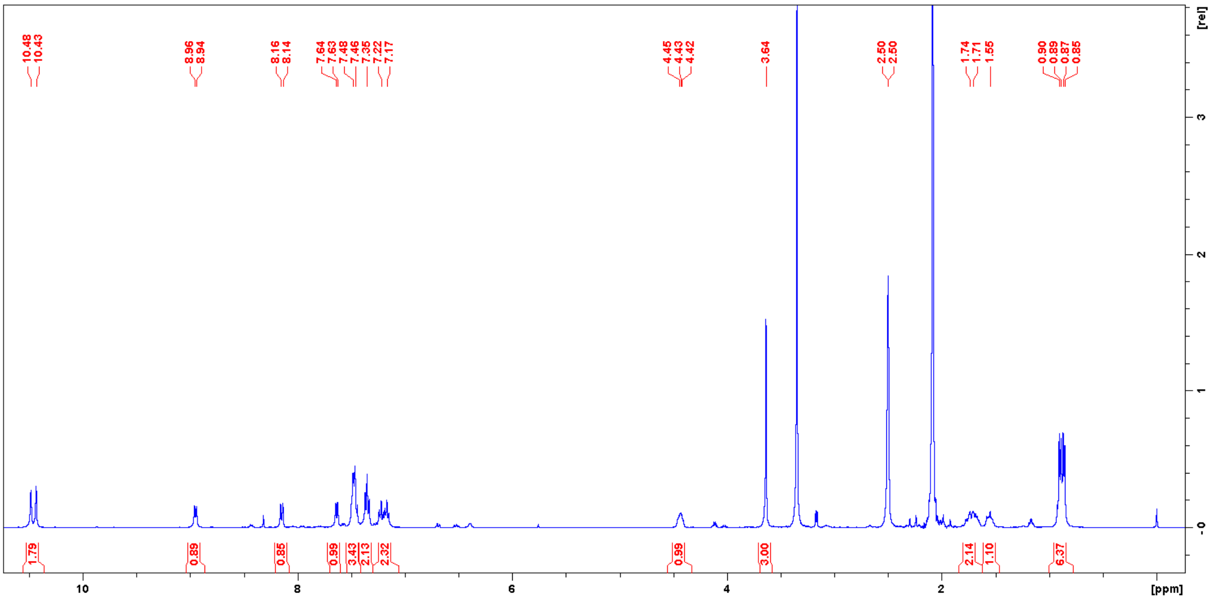

^{13}C-NMR spectrum of **methyl (2*S*)-4-methyl 2-({2-[(phenylcarbamothioyl)amino]benzoyl}amino)pentanoate (10a)**

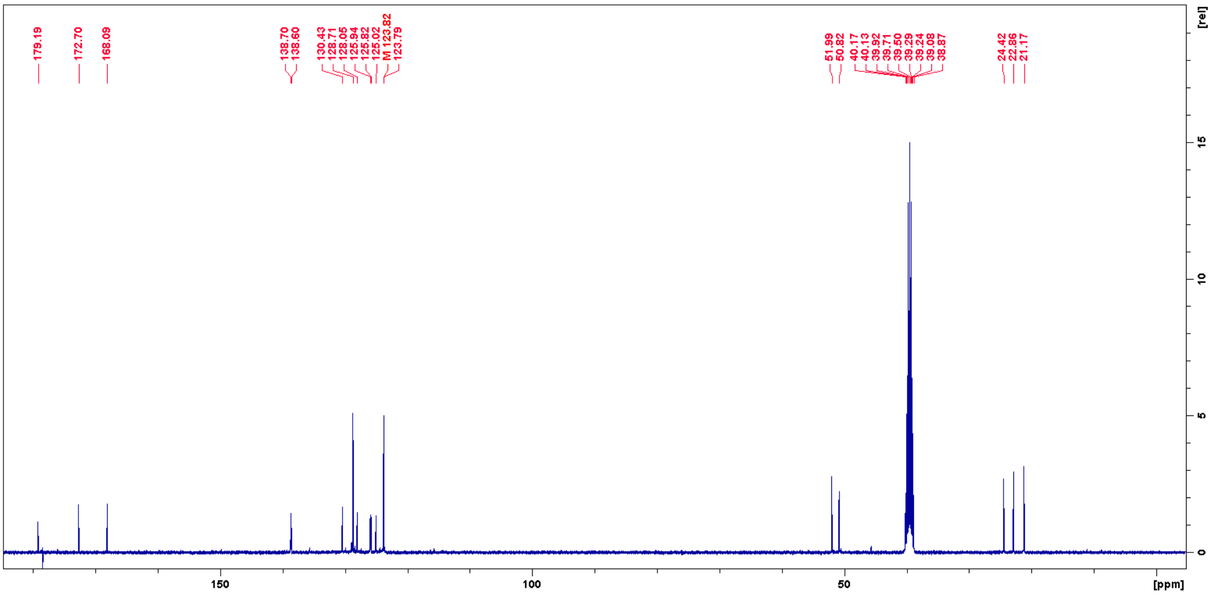

^{1}H-NMR spectrum of **methyl (2*S*)-4-methyl 2-[(2-{[(4-nitrophenyl)carbamothioyl]amino}benzoyl)amino]pentanoate (10b)**

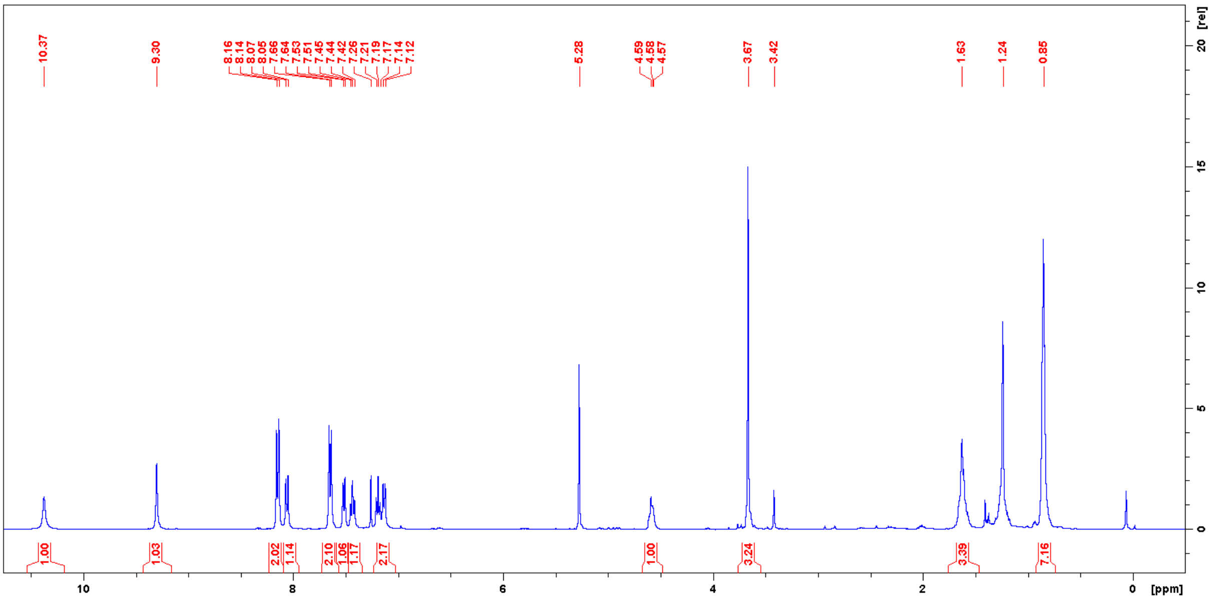

^{13}C-NMR spectrum of **methyl (2*S*)-4-methyl 2-[(2-{[(4-nitrophenyl)carbamothioyl]amino}benzoyl)amino]pentanoate (10b)**

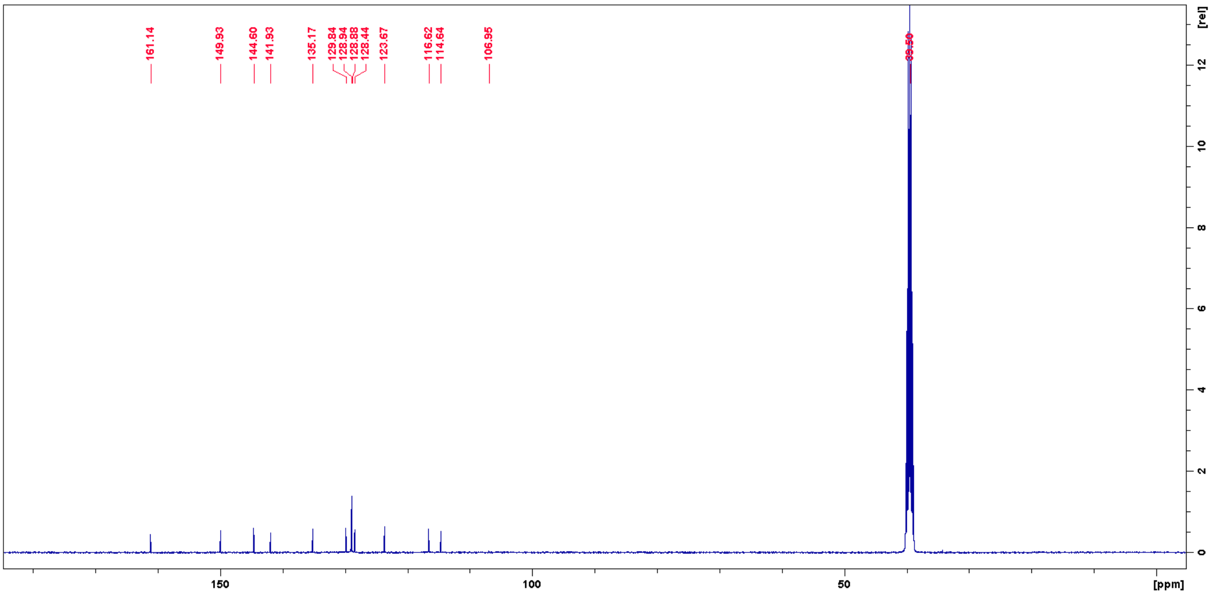

^{1}H-NMR spectrum of **methyl (2*S*)-4-methyl 2-[(2-{[(3,5-bistrifluoromethylphenyl)carbamothioyl]amino}-benzoyl)amino]pentanoate (10c)**

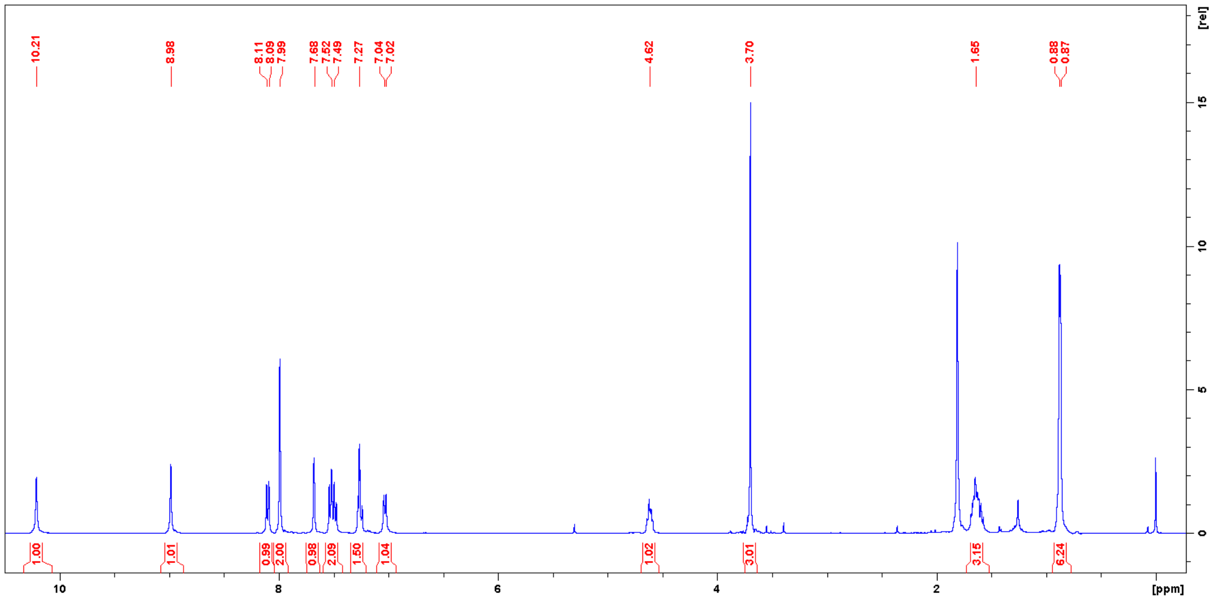

^{13}C-NMR spectrum of **methyl (2*S*)-4-methyl 2-[(2-{[(3,5-bistrifluoromethylphenyl)carbamothioyl]amino}-benzoyl)amino]pentanoate (10c)**

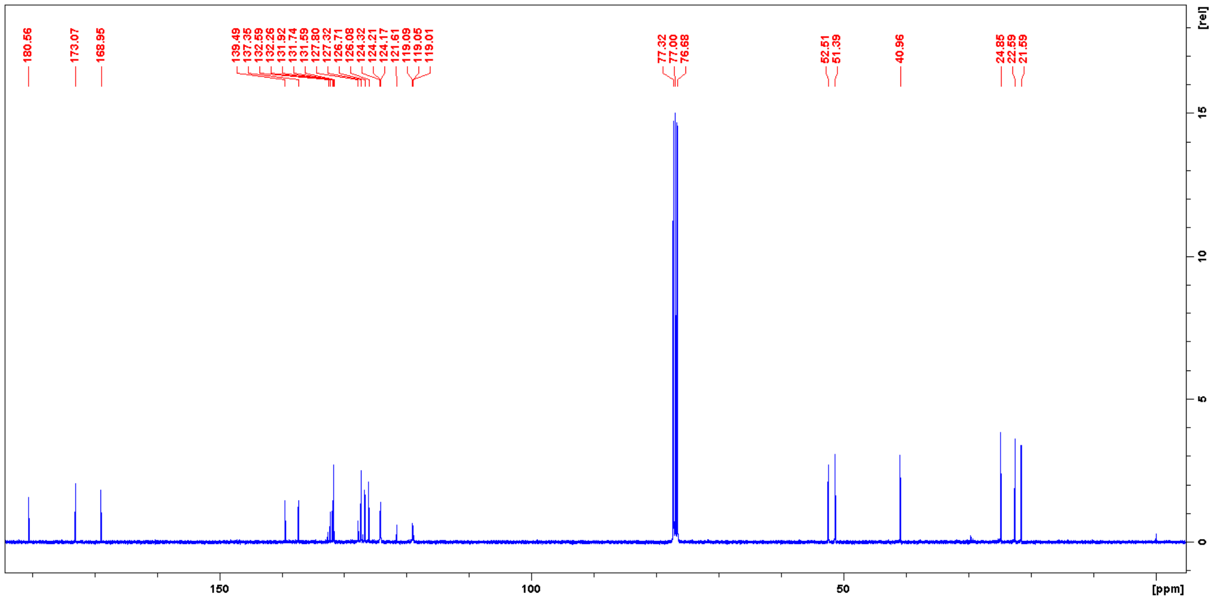

^{1}H-NMR spectrum of **methyl (2*S*)-4-methyl 2-[(2-{[(4-methoxyphenyl)carbamothioyl]amino}benzoyl)amino]pentanoate (10d)**

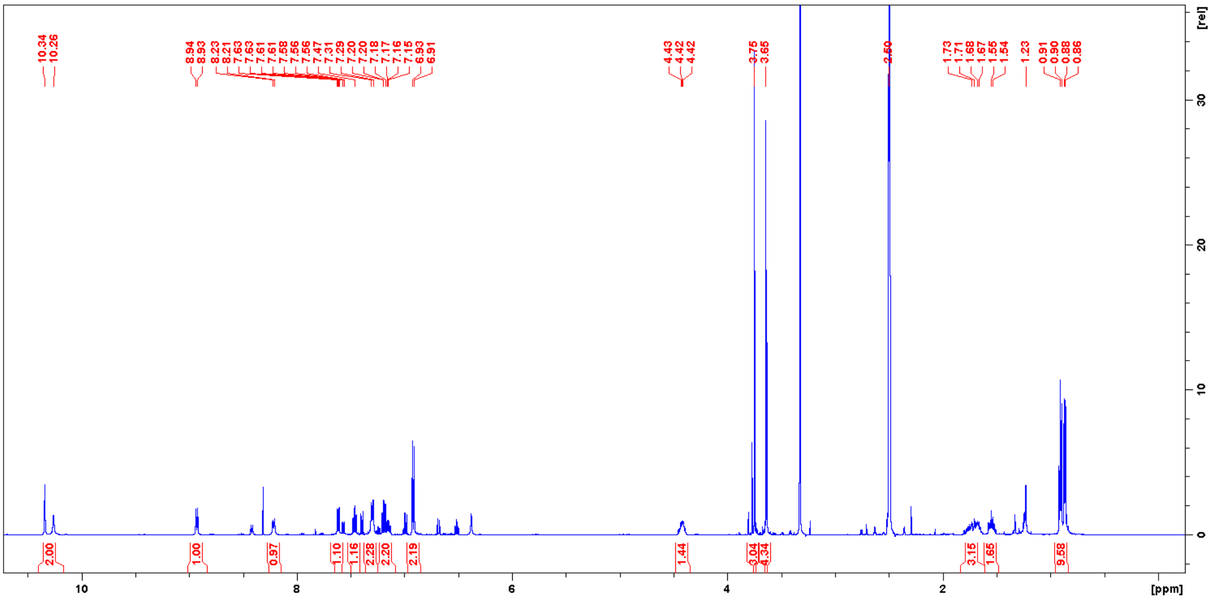

^{13}C-NMR spectrum of **methyl (2*S*)-4-methyl 2-[(2-{[(4-methoxyphenyl)carbamothioyl]amino}benzoyl)amino]pentanoate (10d)**

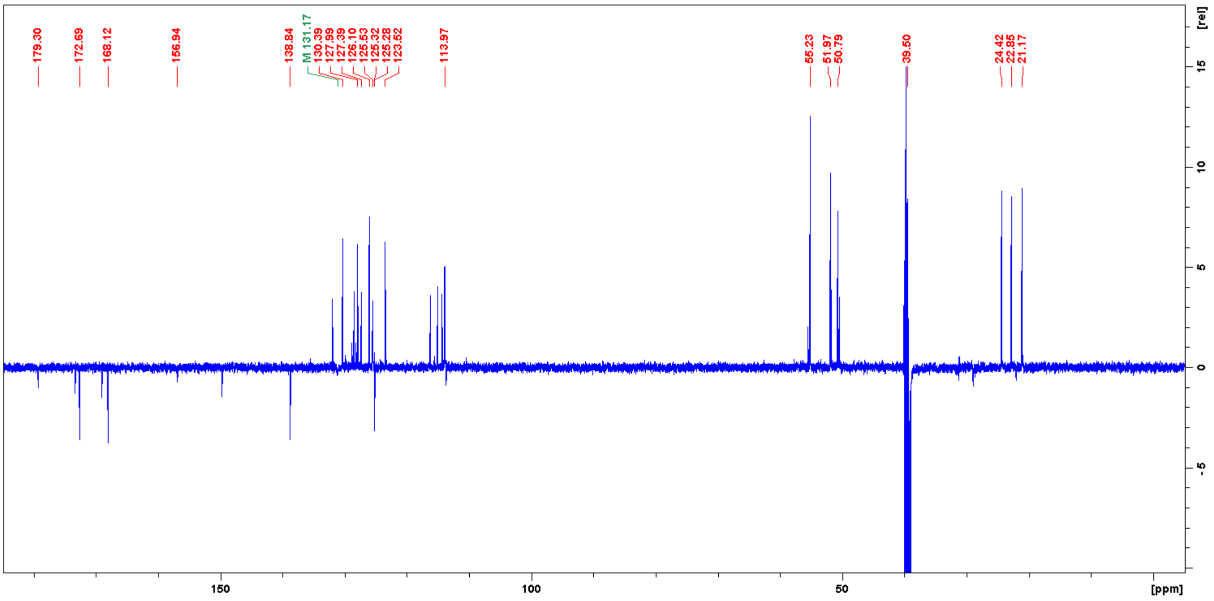

^{1}H-NMR spectrum of **Methyl (2*S*)-4-methyl 2-[(2-{[(4-fluorophenyl)carbamothioyl]amino}benzoyl)amino]pentanoate (10e)**

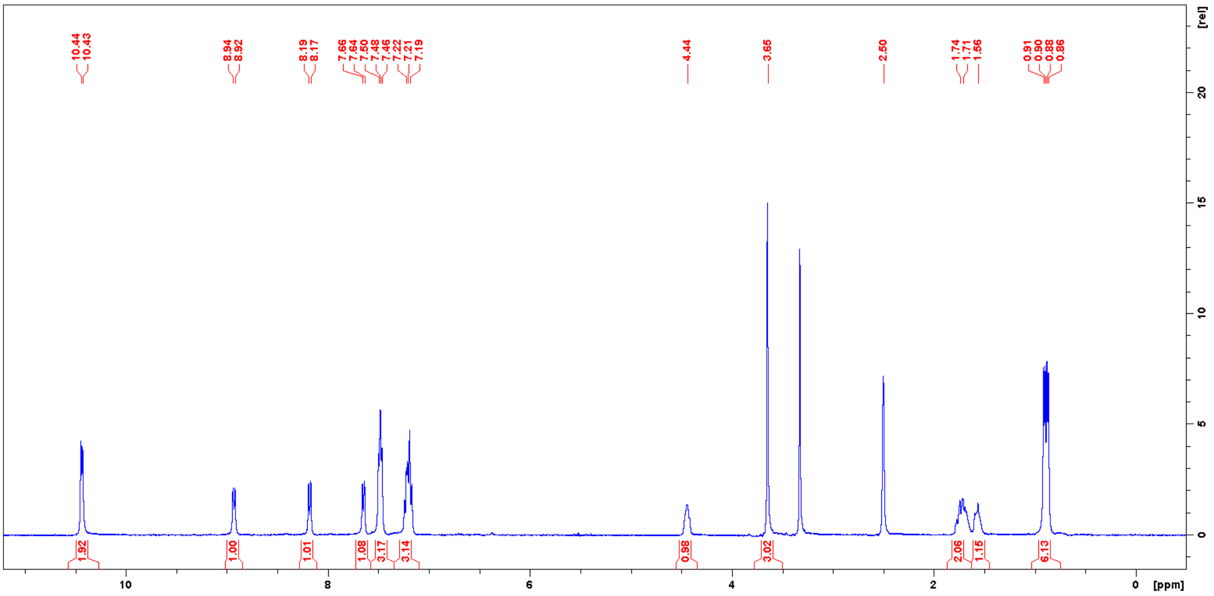

^{13}C-NMR spectrum of **Methyl (2*S*)-4-methyl 2-[(2-{[(4-fluorophenyl)carbamothioyl]amino}benzoyl)amino]pentanoate (10e)**

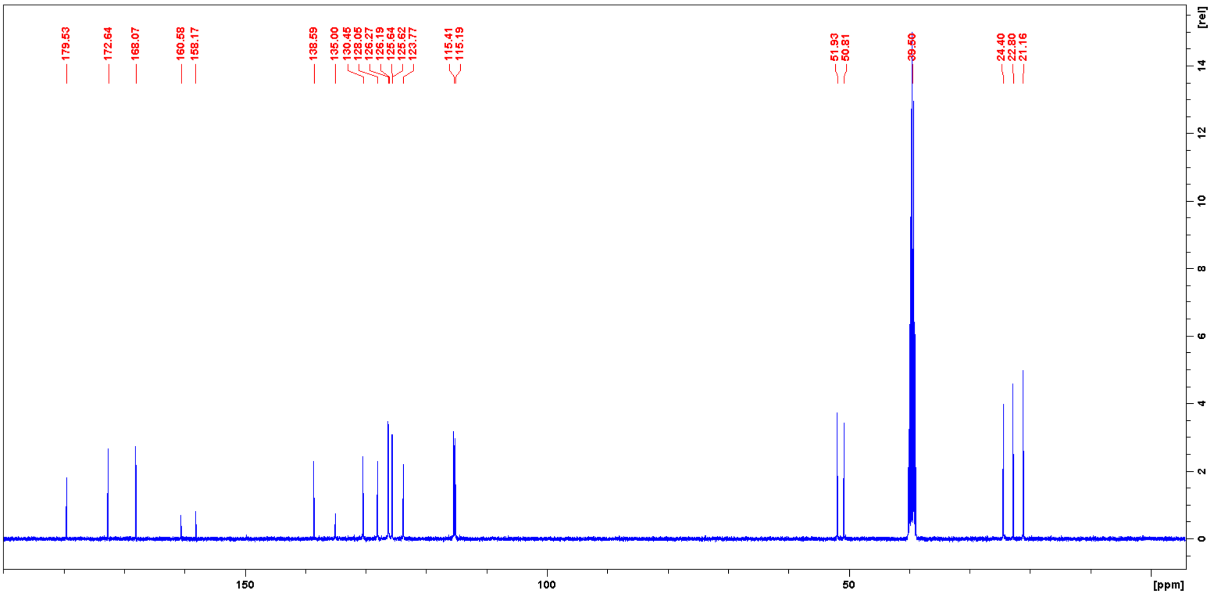

^{1}H-NMR spectrum of **Methyl (2*S*)-4-methyl 2-[(2-{[(3,5-difluorophenyl)carbamothioyl]amino}benzoyl)amino]pentanoate (10f)**

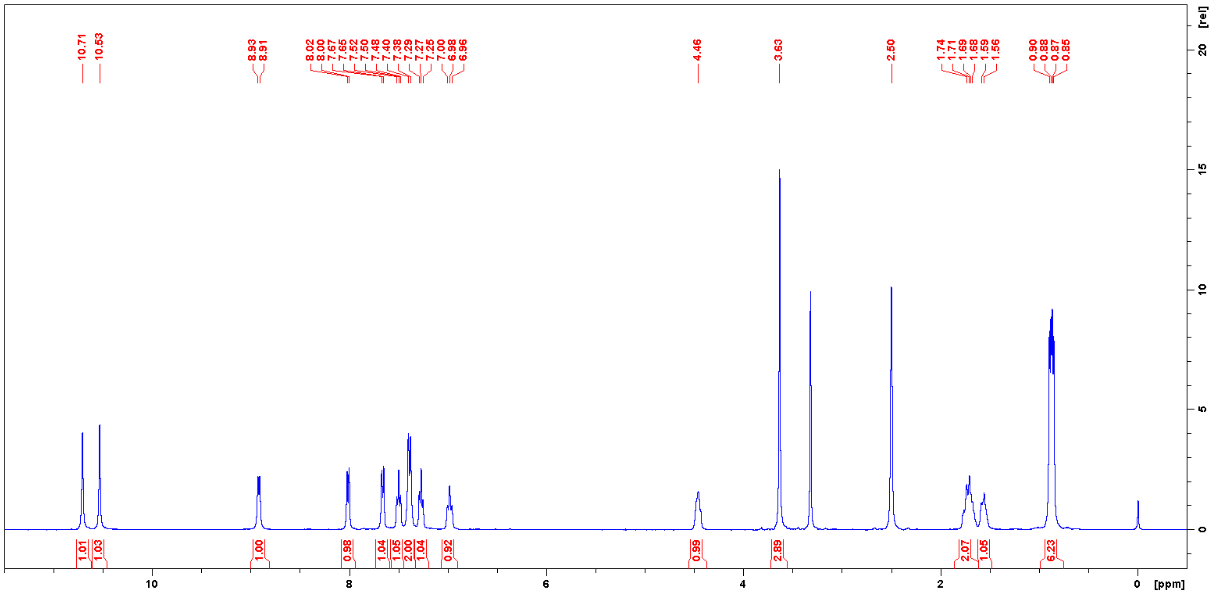

^{13}C-NMR spectrum of **Methyl (2*S*)-4-methyl 2-[(2-{[(3,5-difluorophenyl)carbamothioyl]amino}benzoyl)amino]pentanoate (10f)**

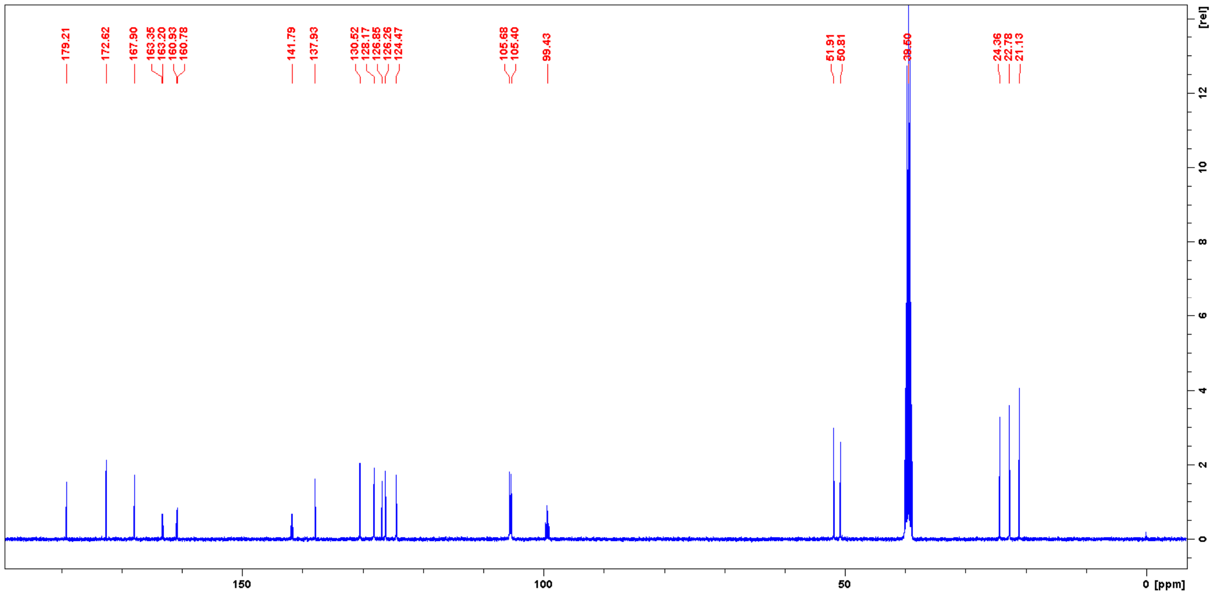

^{1}H-NMR spectrum of **Methyl (2*S*)-4-methyl 2-[(2-{[(2,6-difluorophenyl)carbamothioyl]amino}benzoyl)amino]pentanoate (10g)**

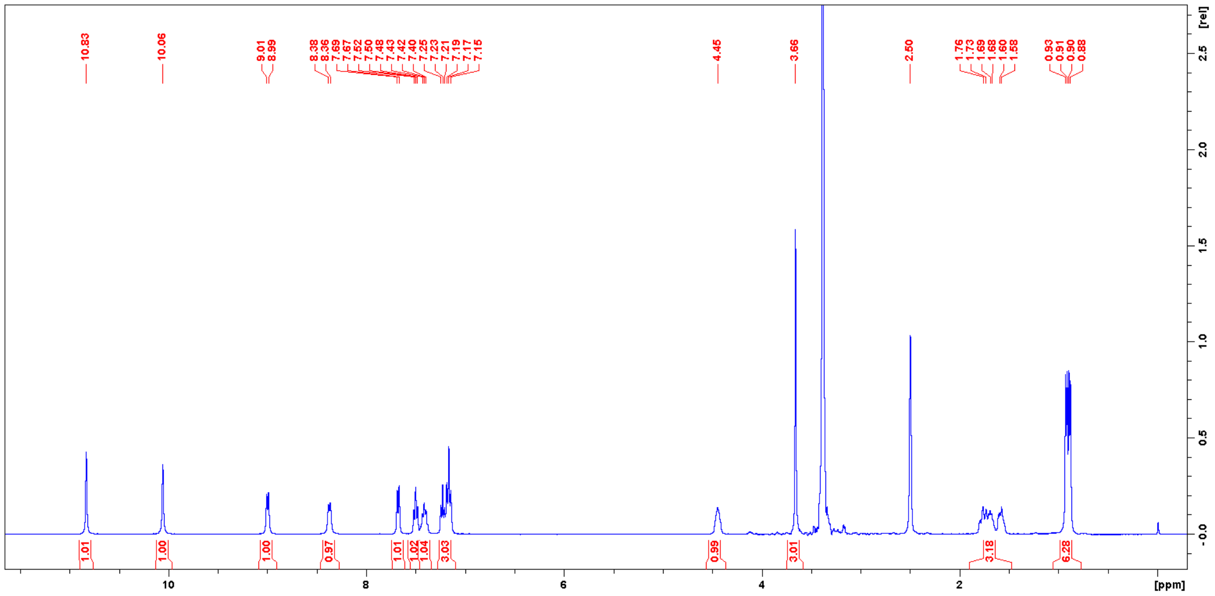

^{13}C-NMR spectrum of **Methyl (2*S*)-4-methyl 2-[(2-{[(2,6-difluorophenyl)carbamothioyl]amino}benzoyl)amino]pentanoate (10g)**

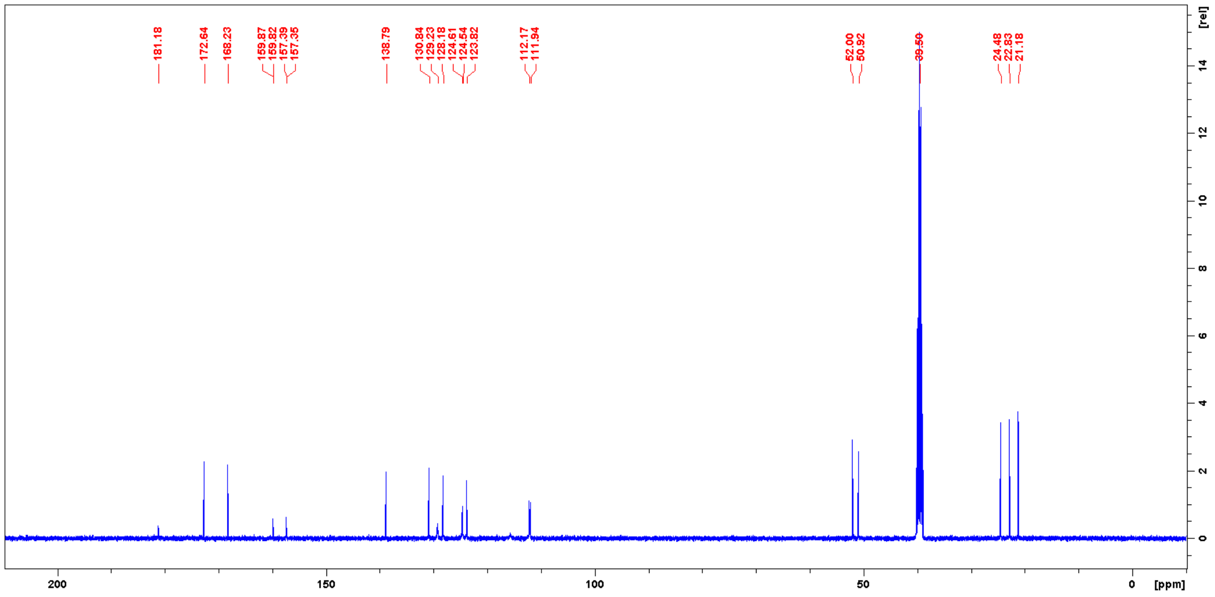

^{1}H-NMR spectrum of **methyl (2*S*)-3-methyl 2-({2-[(phenylcarbamothioyl)amino]benzoyl}amino)pentanoate (11a)**

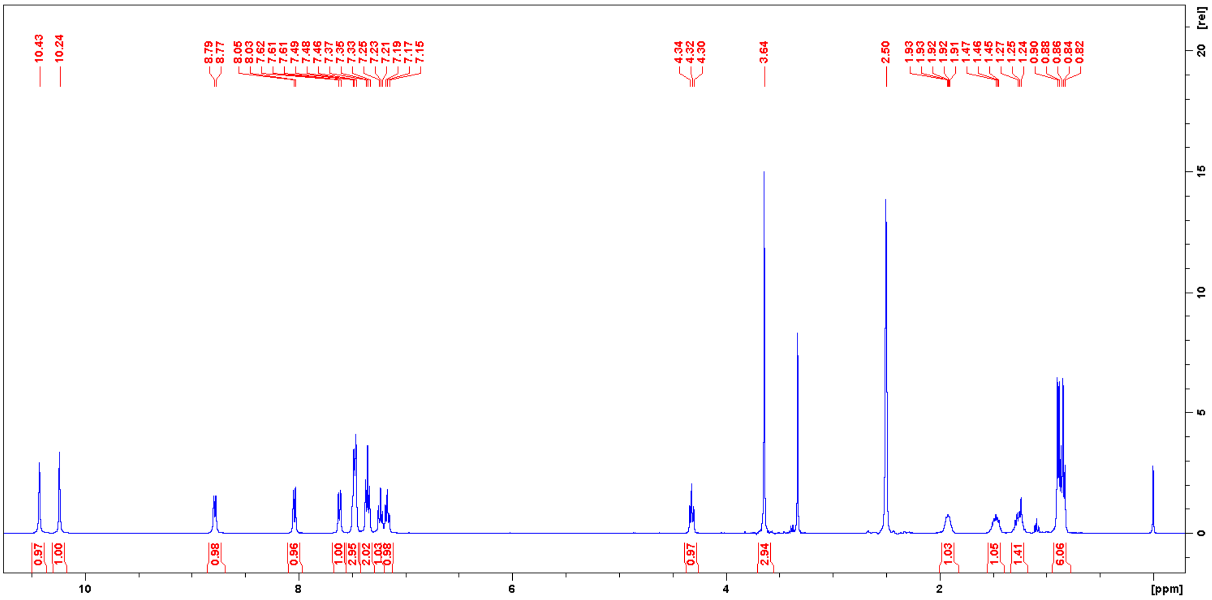

^{13}C-NMR spectrum of **methyl (2*S*)-3-methyl 2-({2-[(phenylcarbamothioyl)amino]benzoyl}amino)pentanoate (11a)**

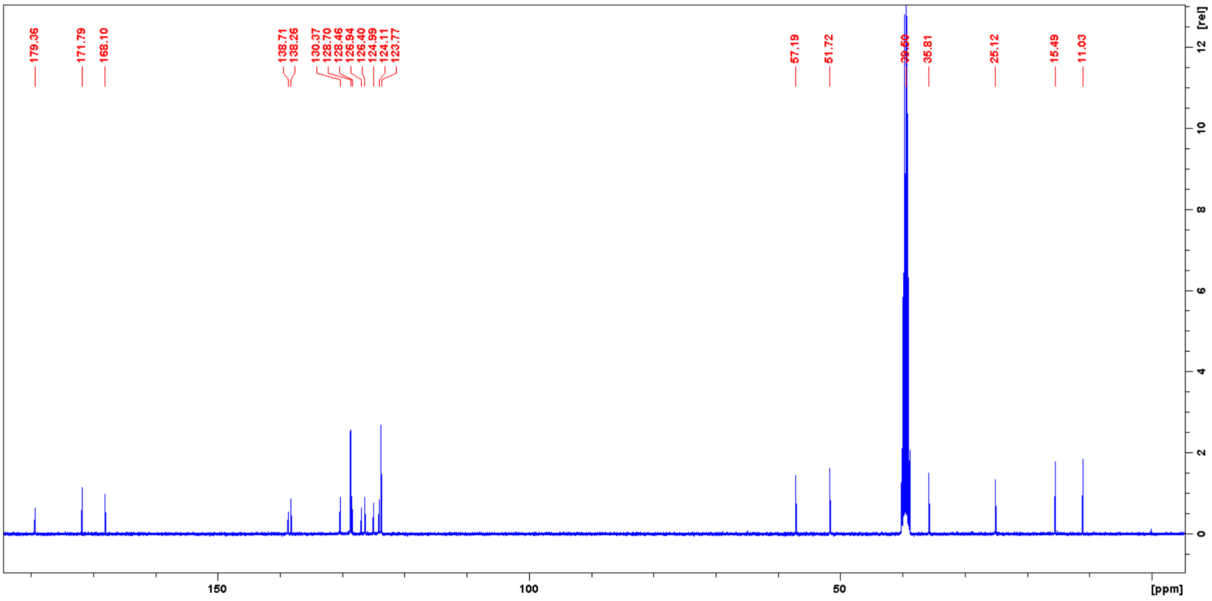

^{1}H-NMR spectrum of **methyl (2*S*)-3-methyl 2-[(2-{[(4-nitrophenyl)carbamothioyl]amino}benzoyl)amino]pentanoate (11b)**

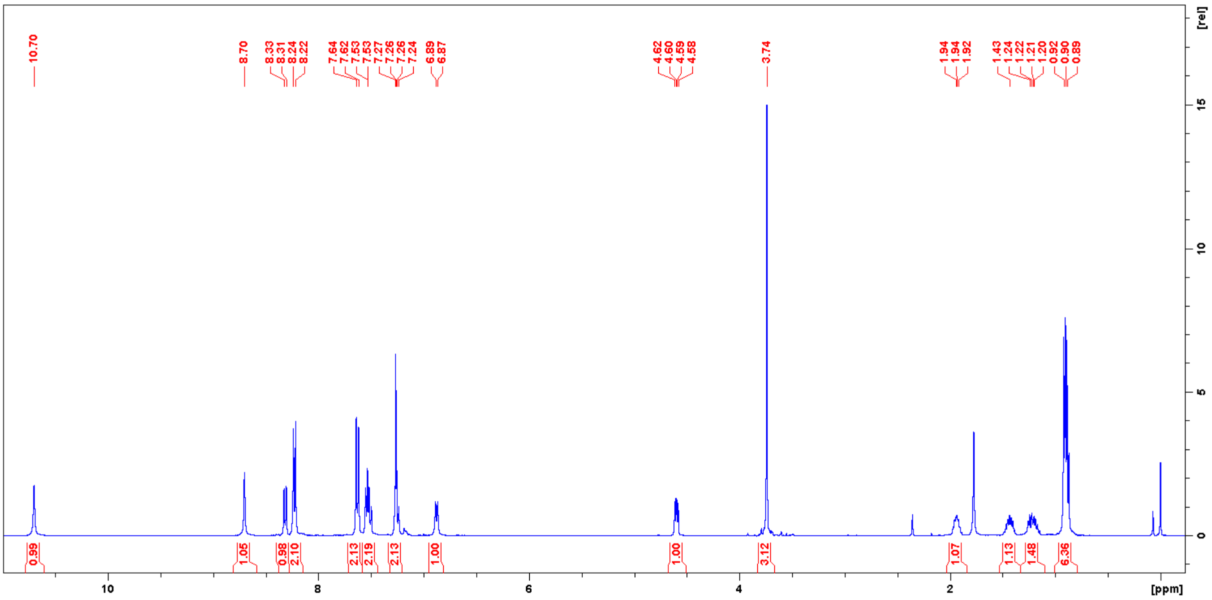

^{13}C-NMR spectrum of **methyl (2*S*)-3-methyl 2-[(2-{[(4-nitrophenyl)carbamothioyl]amino}benzoyl)amino]pentanoate (11b)**

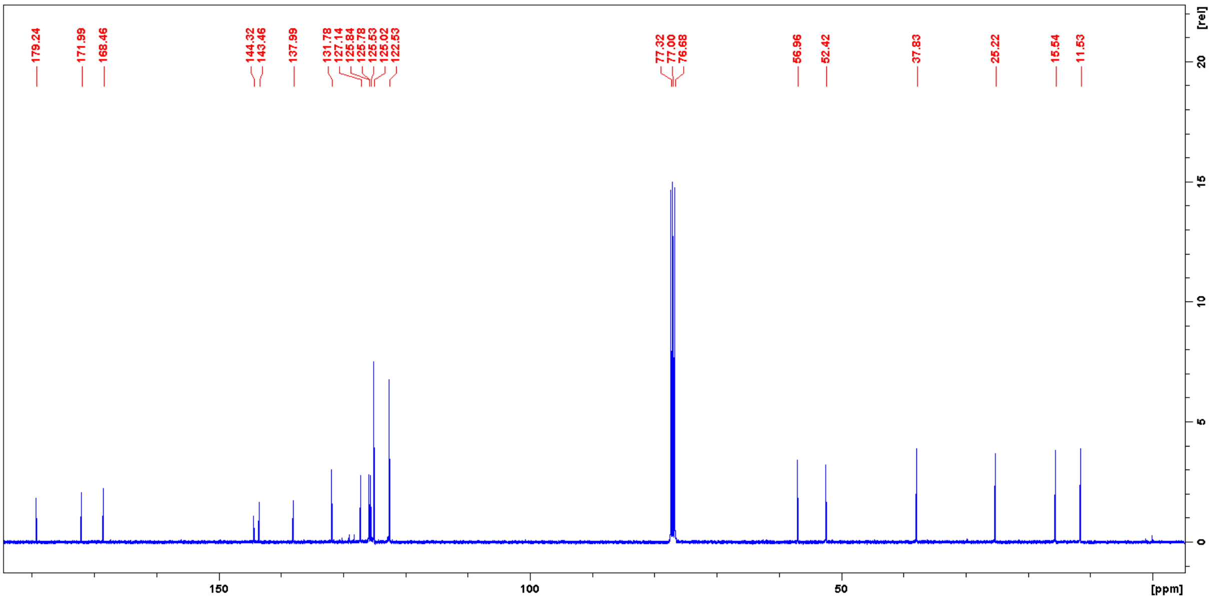

^{1}H-NMR spectrum of **methyl (2*S*)-3-methyl 2-[(2-{[(3,5-bistetrifluoromethylphenyl)carbamothioyl]amino} benzoyl)amino]pentanoate (11c)**

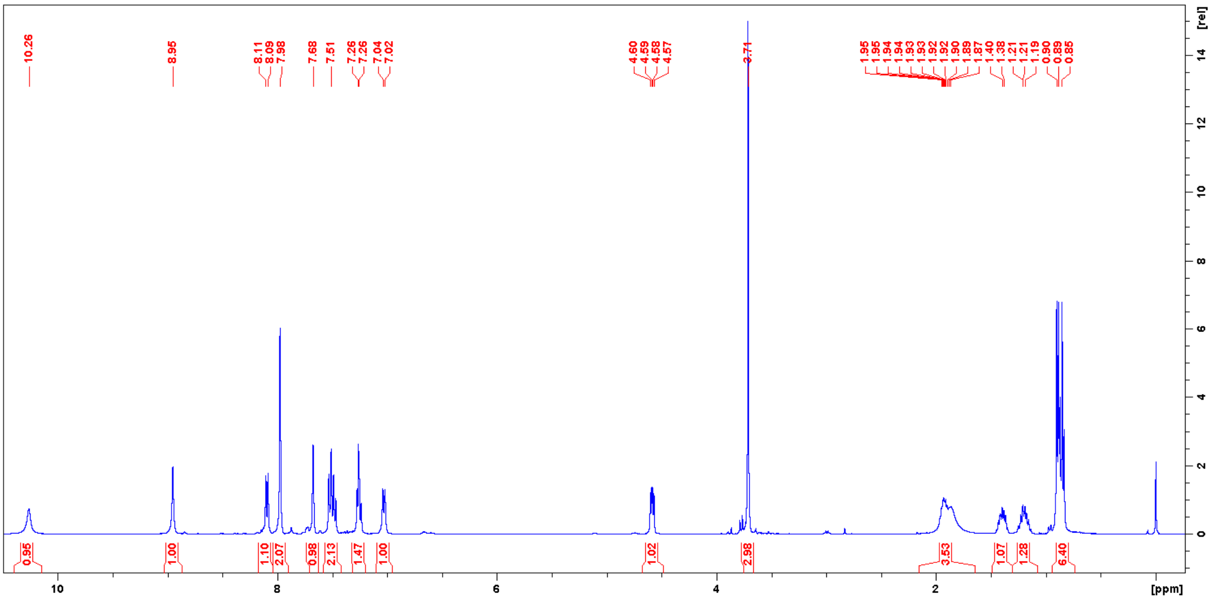

^{13}C-NMR spectrum of **methyl (2*S*)-3-methyl 2-[(2-{[(3,5-bistetrifluoromethylphenyl)carbamothioyl]amino} benzoyl)amino]pentanoate (11c)**

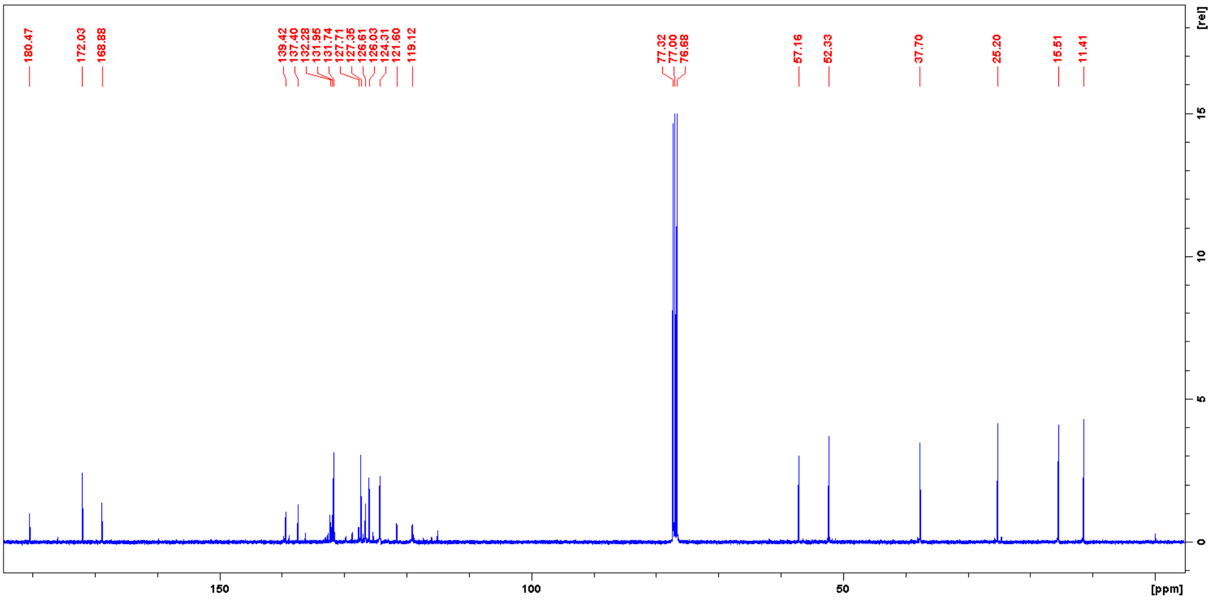

^{1}H-NMR spectrum of **methyl (2*S*)-3-methyl 2-[(2-{[(4-methoxyphenyl)carbamothioyl]amino}benzoyl)amino]pentanoate (11d)**

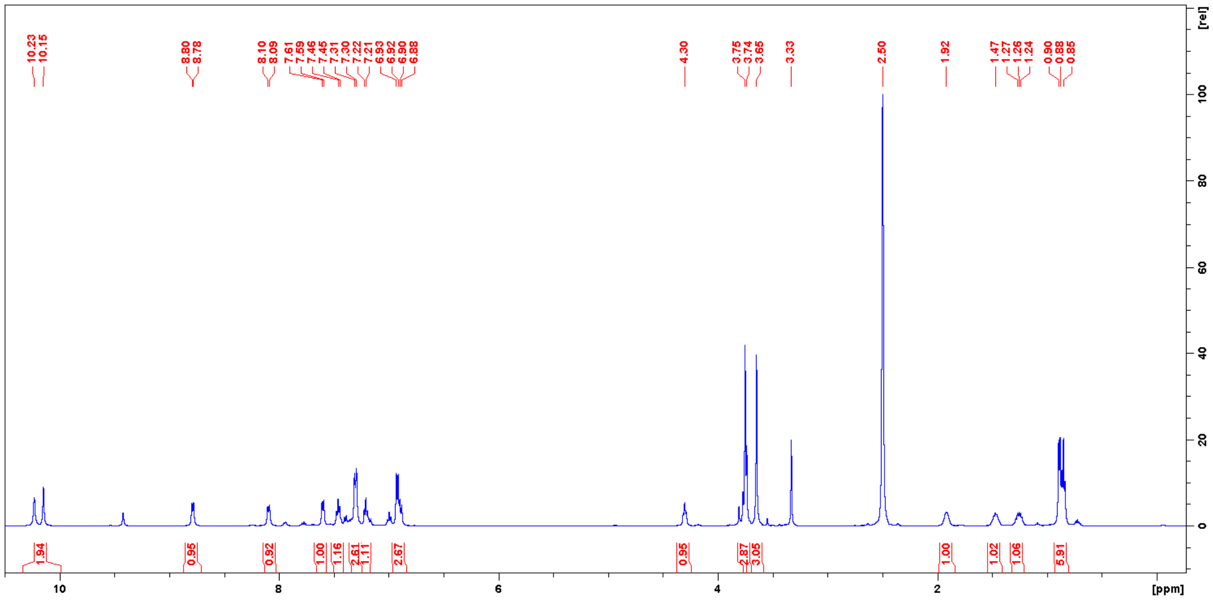

^{13}C-NMR spectrum of **methyl (2*S*)-3-methyl 2-[(2-{[(4-methoxyphenyl)carbamothioyl]amino}benzoyl)amino]pentanoate (11d)**

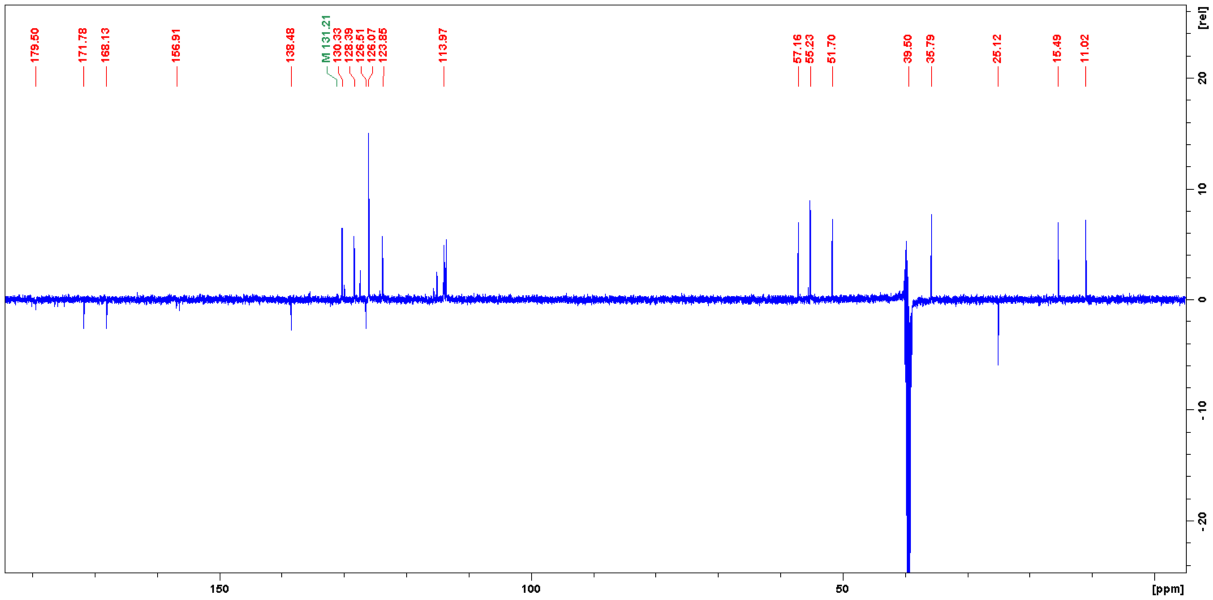

^{1}H-NMR spectrum of **Methyl (2*S*)-3-methyl 2-[(2-{[(4-fluorophenyl)carbamothioyl]amino}benzoyl)amino]pentanoate (11e)**

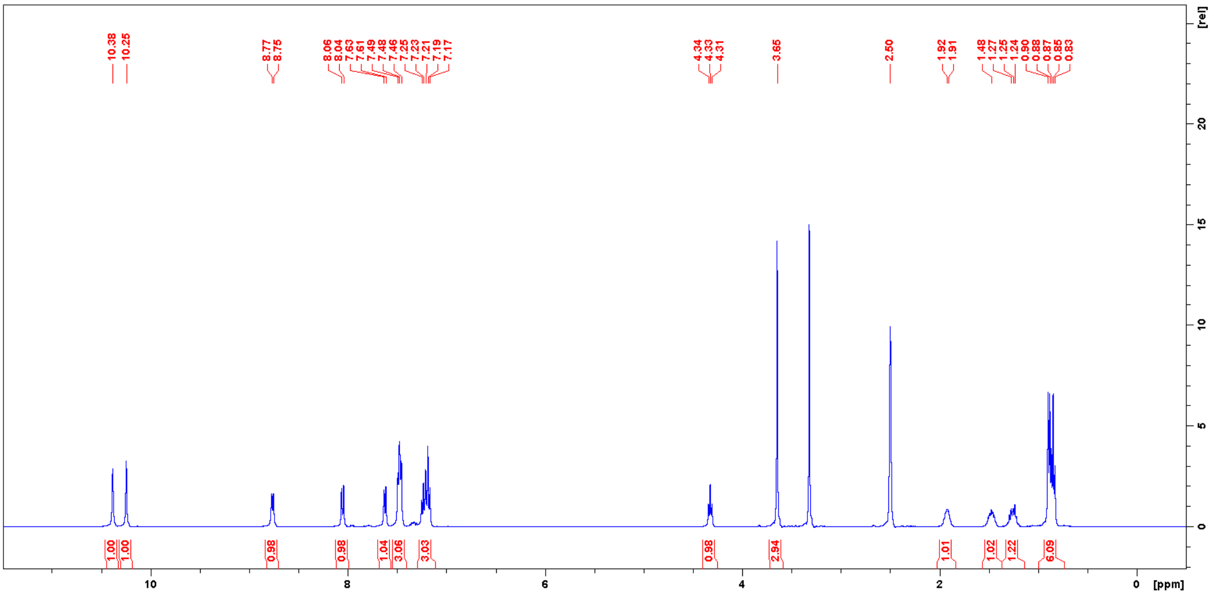

^{13}C-NMR spectrum of **Methyl (2*S*)-3-methyl 2-[(2-{[(4-fluorophenyl)carbamothioyl]amino}benzoyl)amino]pentanoate (11e)**

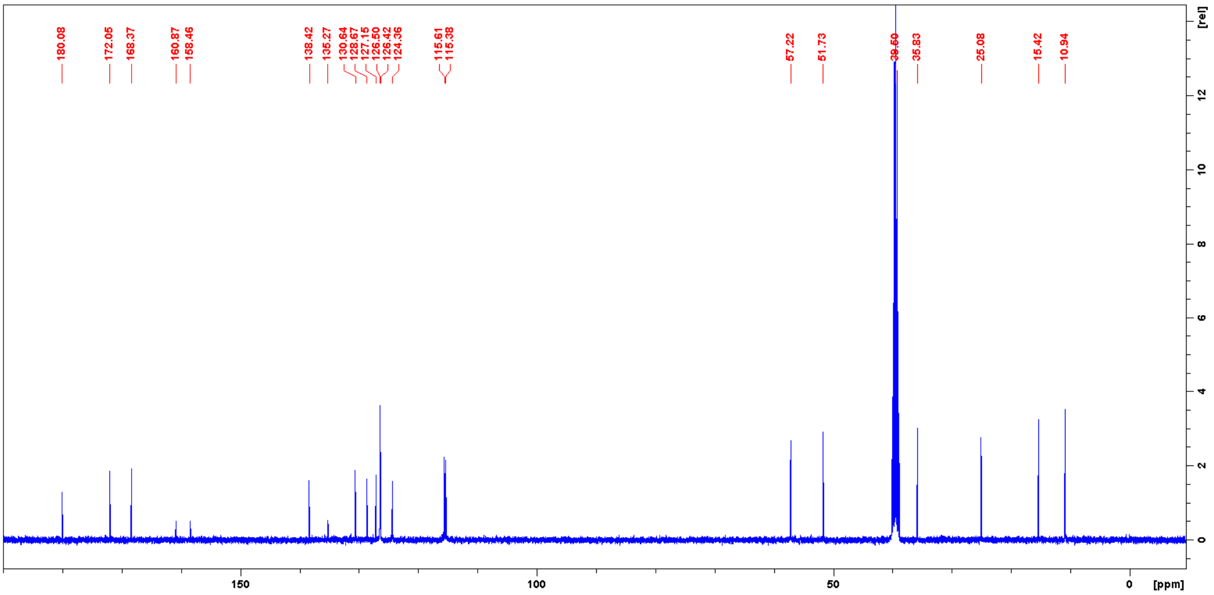

^{1}H-NMR spectrum of **Methyl (2*S*)-3-methyl 2-[(2-{[(3,5-difluorophenyl)carbamothioyl]amino}benzoyl)amino]pentanoate (11f)**

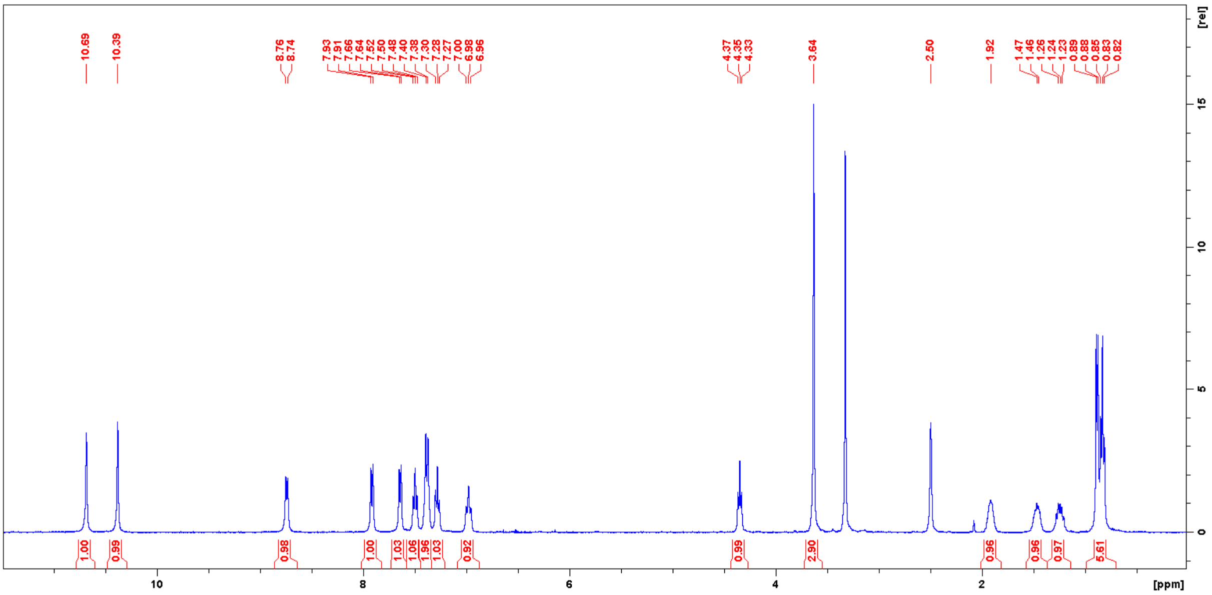

^{13}C-NMR spectrum of **Methyl (2*S*)-3-methyl 2-[(2-{[(3,5-difluorophenyl)carbamothioyl]amino}benzoyl)amino]pentanoate (11f)**

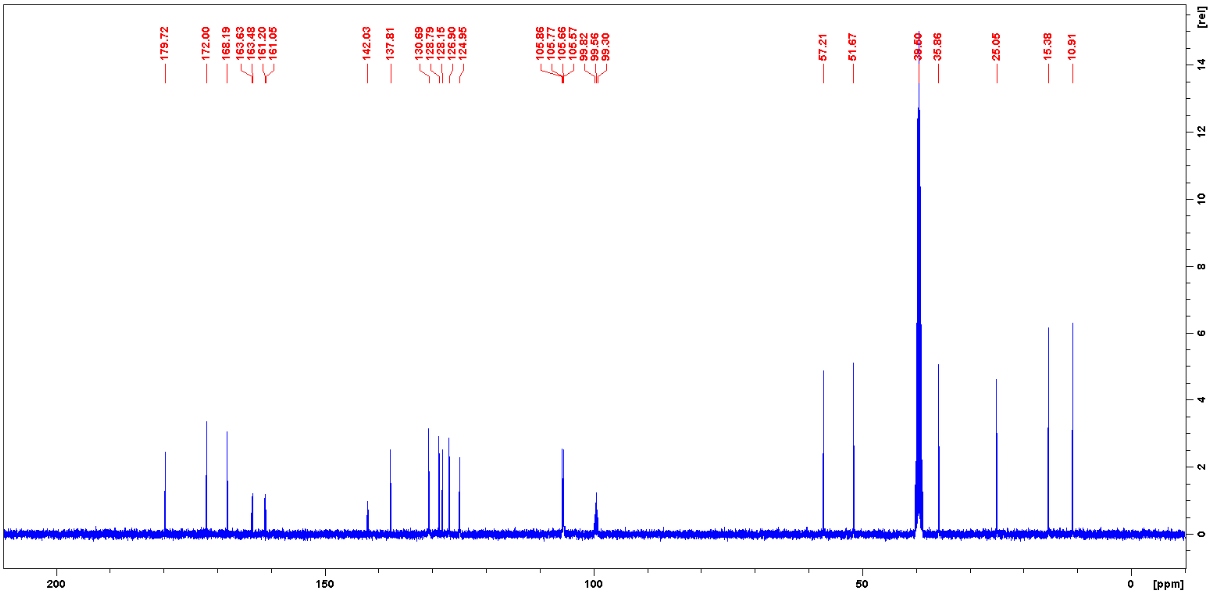

^{1}H-NMR spectrum of **Methyl (2*S*)-3-methyl 2-[(2-{[(2,6-difluorophenyl)carbamothioyl]amino}benzoyl)amino]pentanoate (11g)**

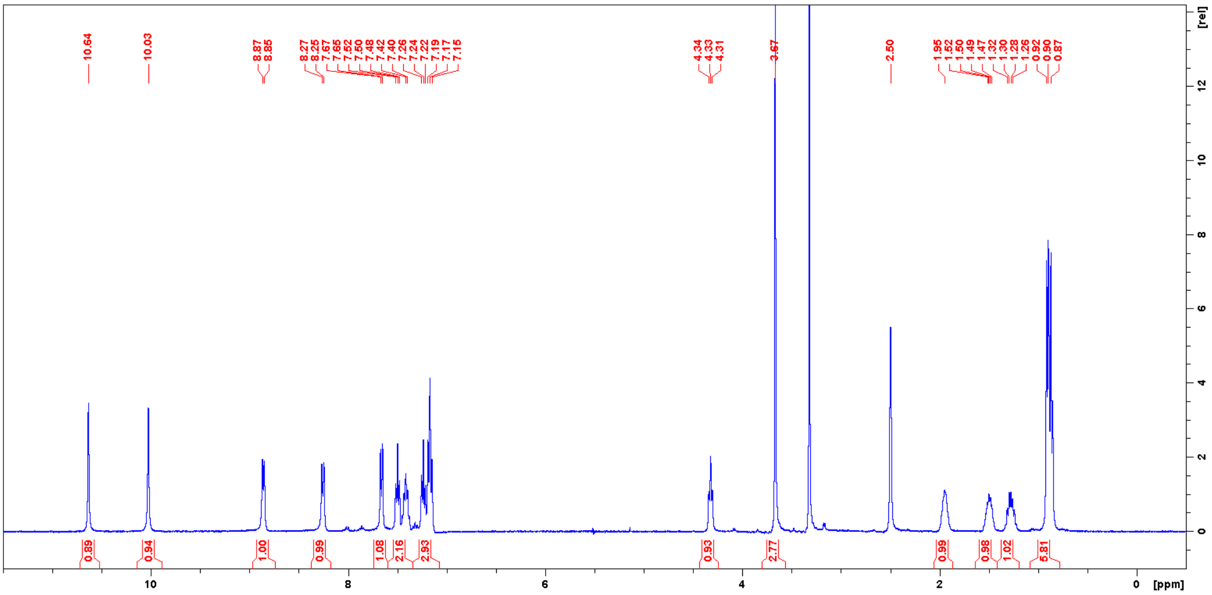

^{13}C-NMR spectrum of **Methyl (2*S*)-3-methyl 2-[(2-{[(2,6-difluorophenyl)carbamothioyl]amino}benzoyl)amino]pentanoate (11g)**

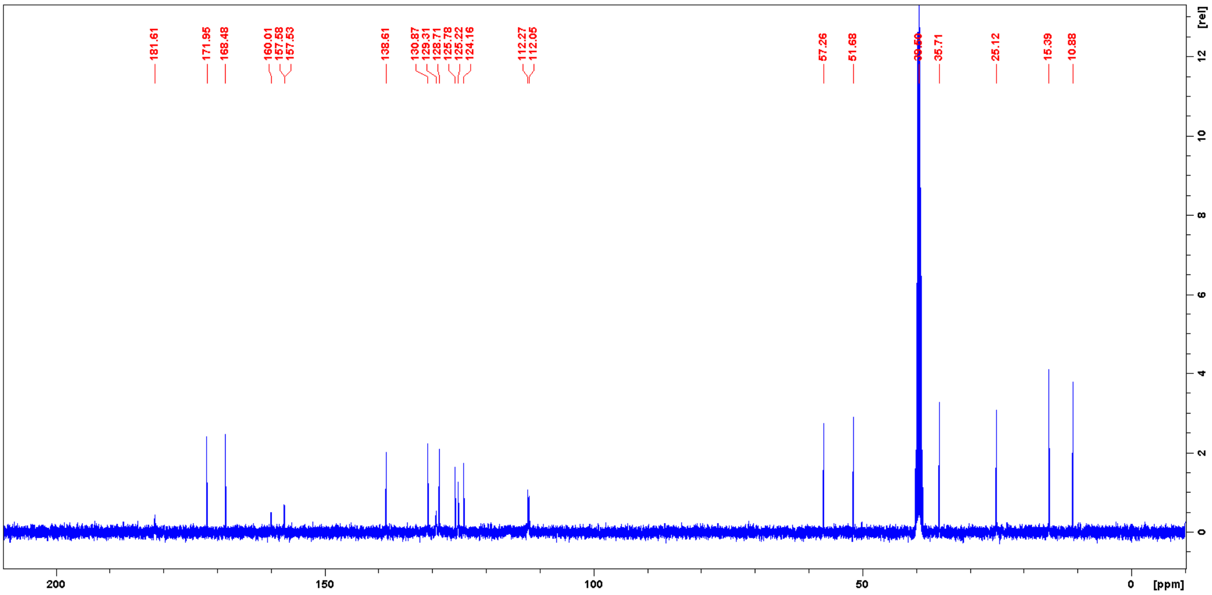

^{1}H-NMR spectrum of ***N,N'*-diphenylthiourea (12a)**

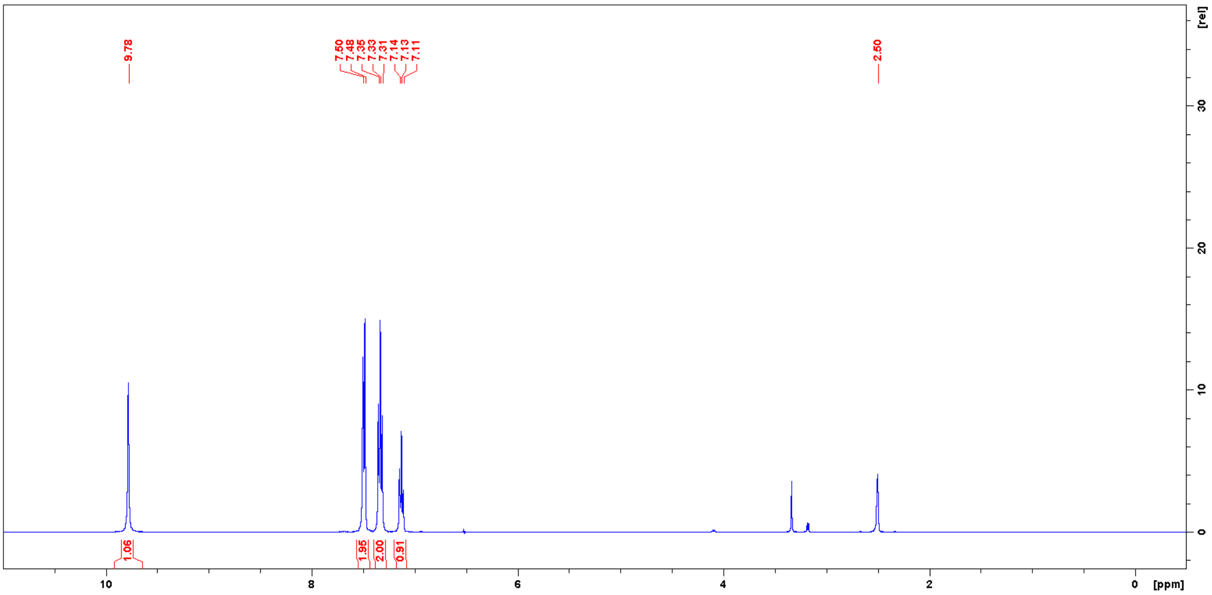

^{13}C-NMR spectrum of ***N,N'*-diphenylthiourea (12a)**

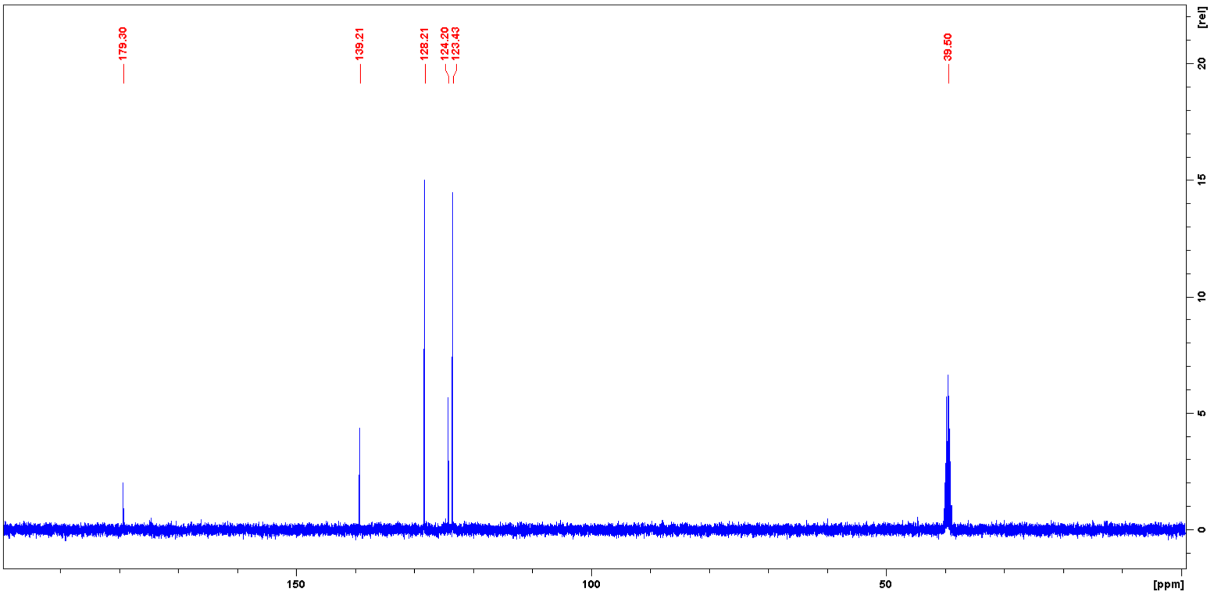

^{1}H-NMR spectrum of ***N*-(4-nitrophenyl)-*N*'-phenylthiourea (12b)**

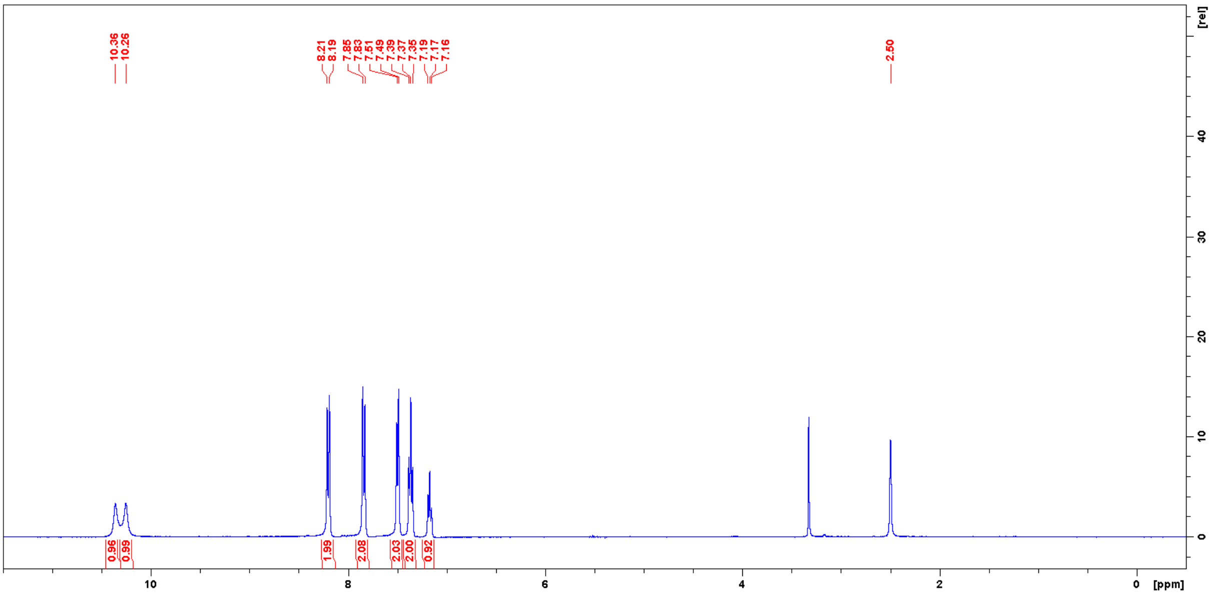

^{13}C-NMR spectrum of ***N*-(4-nitrophenyl)-*N*'-phenylthiourea (12b)**

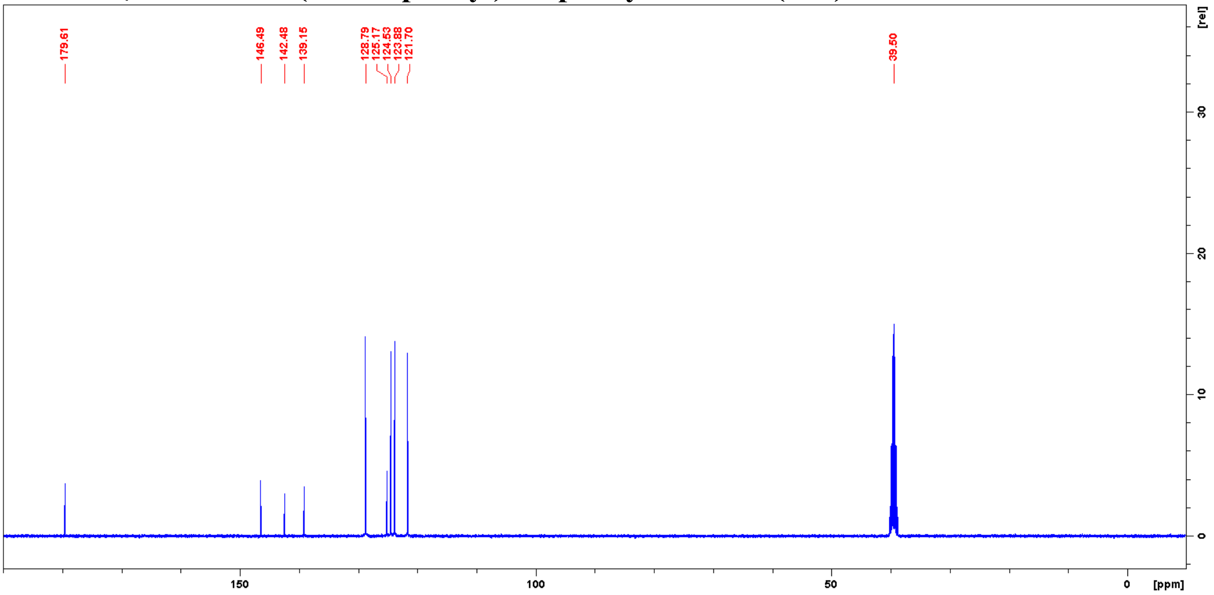

^{1}H-NMR spectrum of ***N*-[3,5-bis(trifluoromethyl)phenyl]-*N*'-phenylthiourea (12c)**

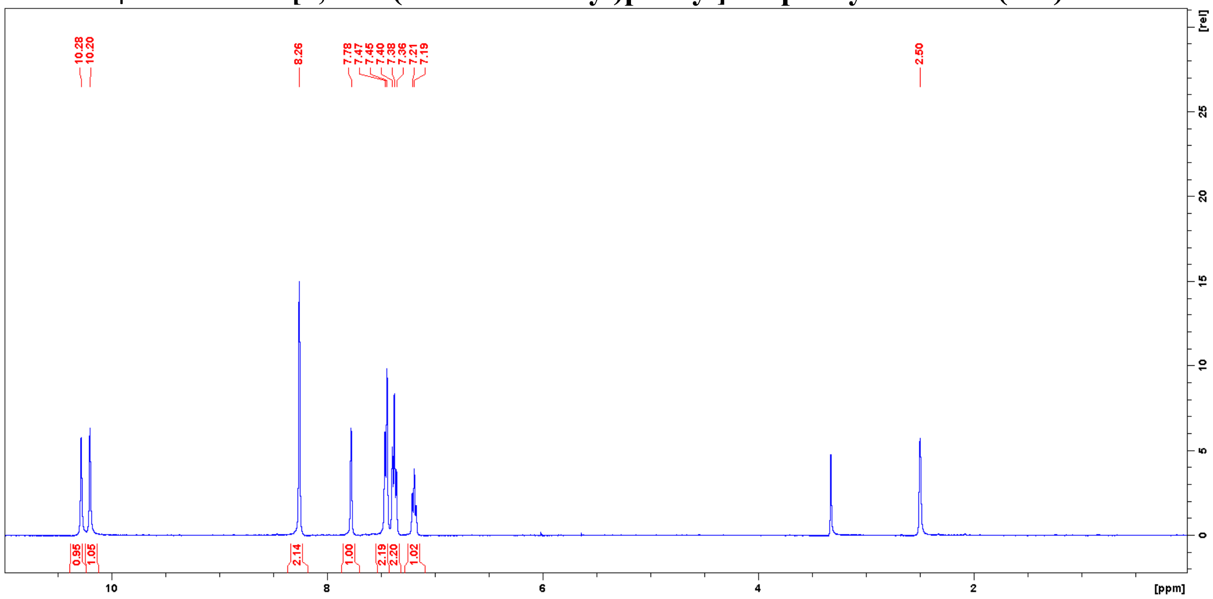

^{13}C-NMR spectrum of ***N*-[3,5-bis(trifluoromethyl)phenyl]-*N*'-phenylthiourea (12c)**

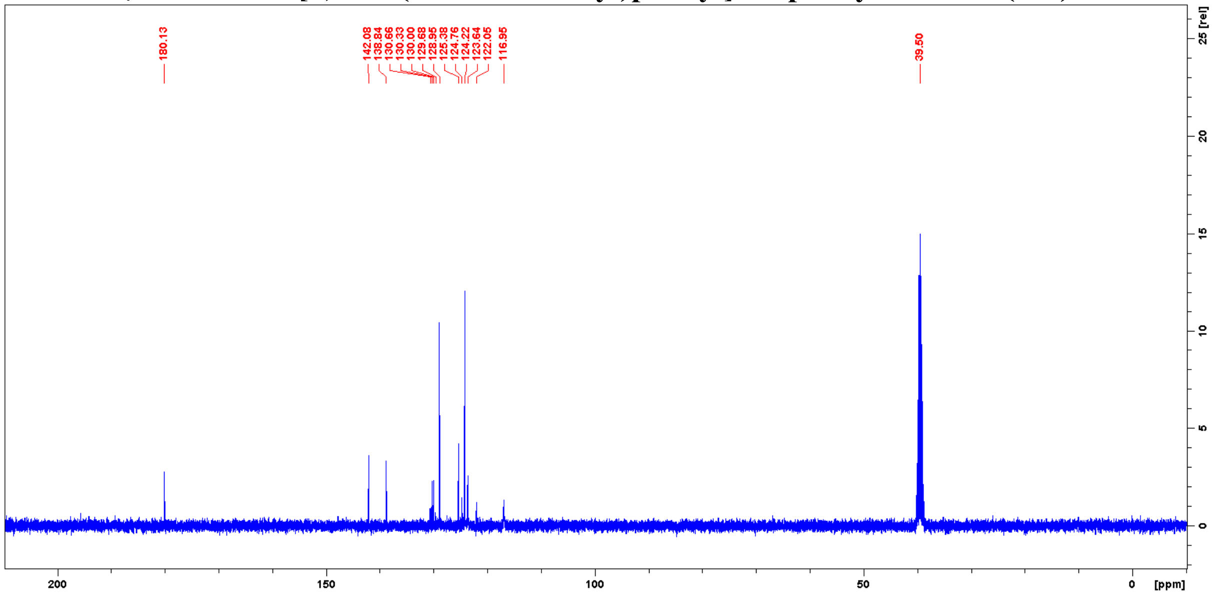

^{1}H-NMR spectrum of ***N*-(4-methoxyphenyl)-*N'*-phenylthiourea (12d)**

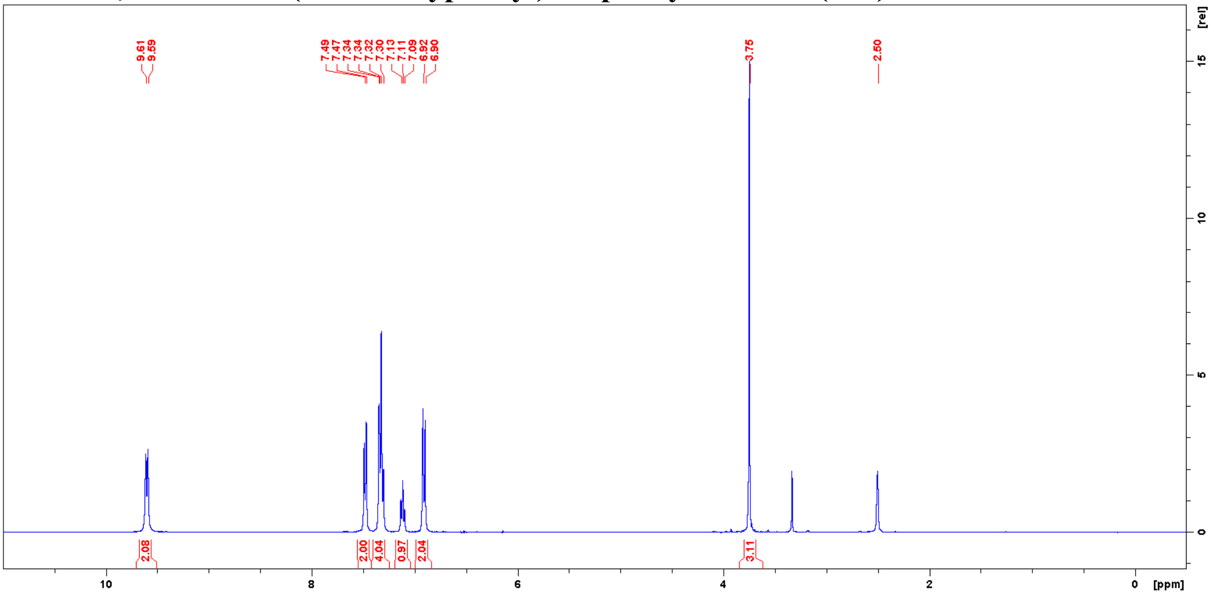

^{13}C-NMR spectrum of ***N*-(4-methoxyphenyl)-*N'*-phenylthiourea (12d)**

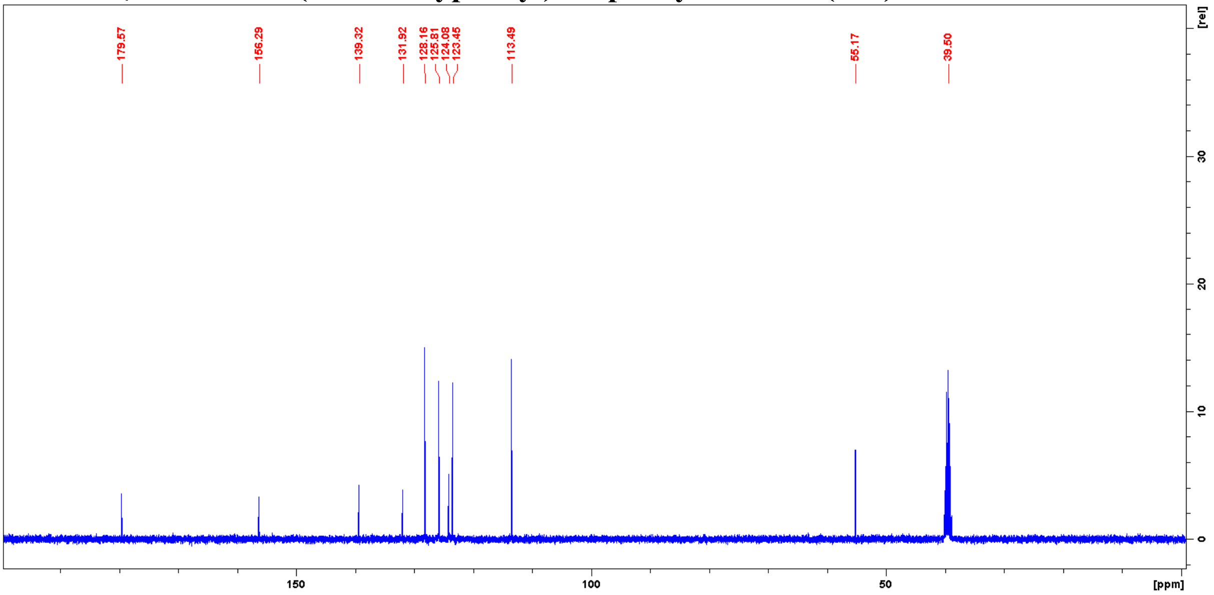

^{1}H-NMR spectrum of ***N*-(4-fluorophenyl)-*N*'-phenylthiourea (12e)**

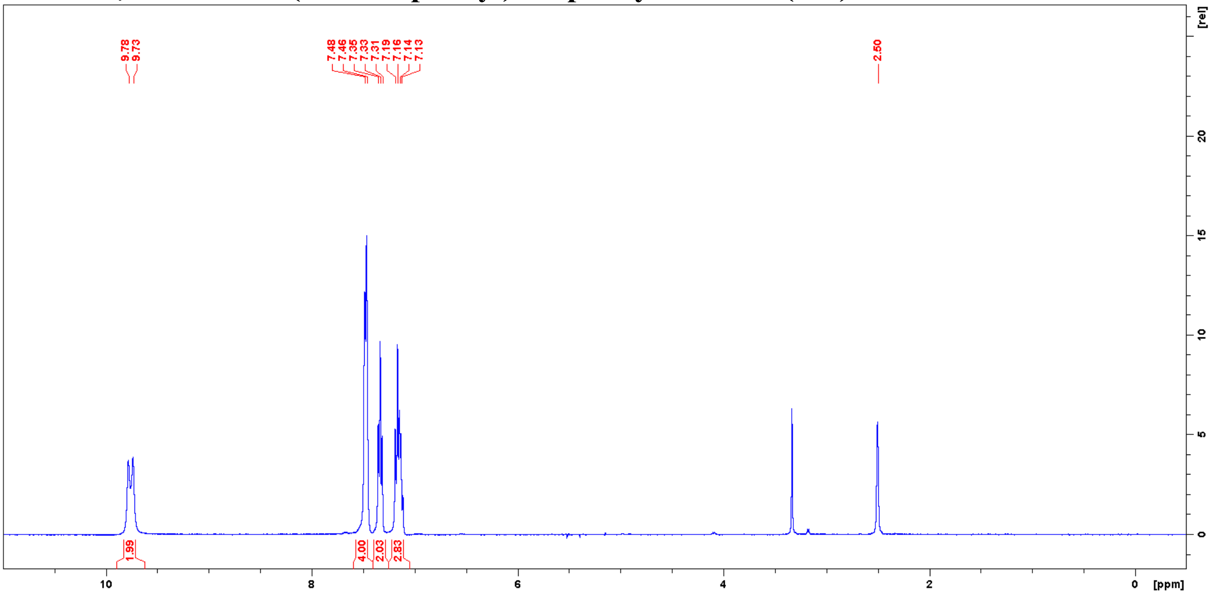

^{13}C-NMR spectrum of ***N*-(4-fluorophenyl)-*N*'-phenylthiourea (12e)**

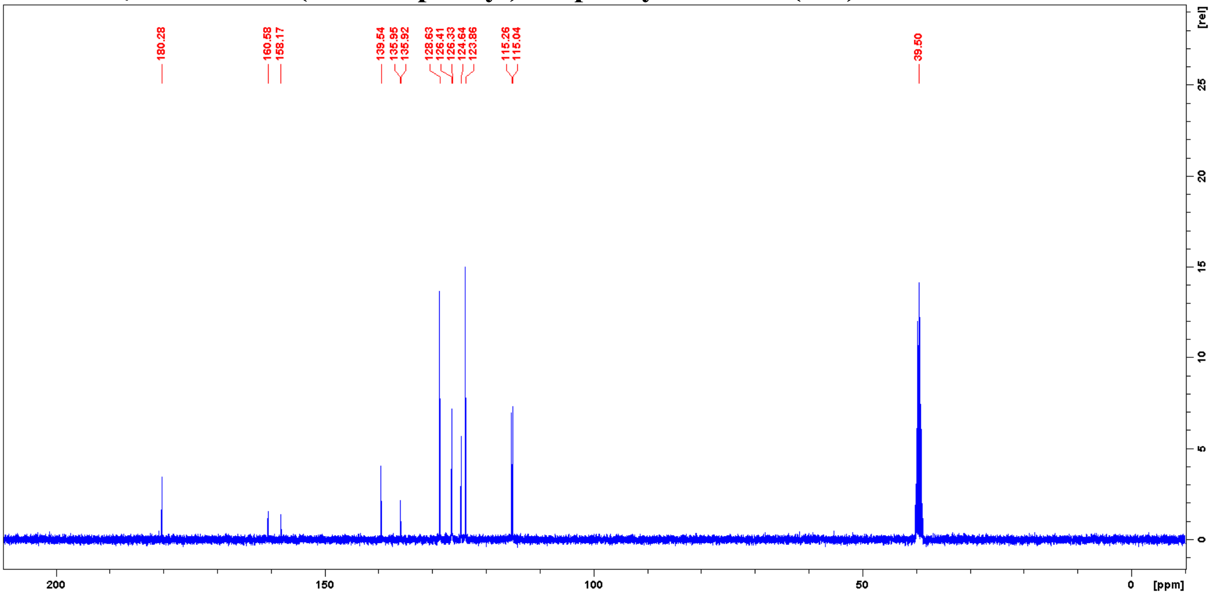

^{1}H-NMR spectrum of ***N*-(3,5-difluorophenyl)-*N'*-phenylthiourea (12f)**

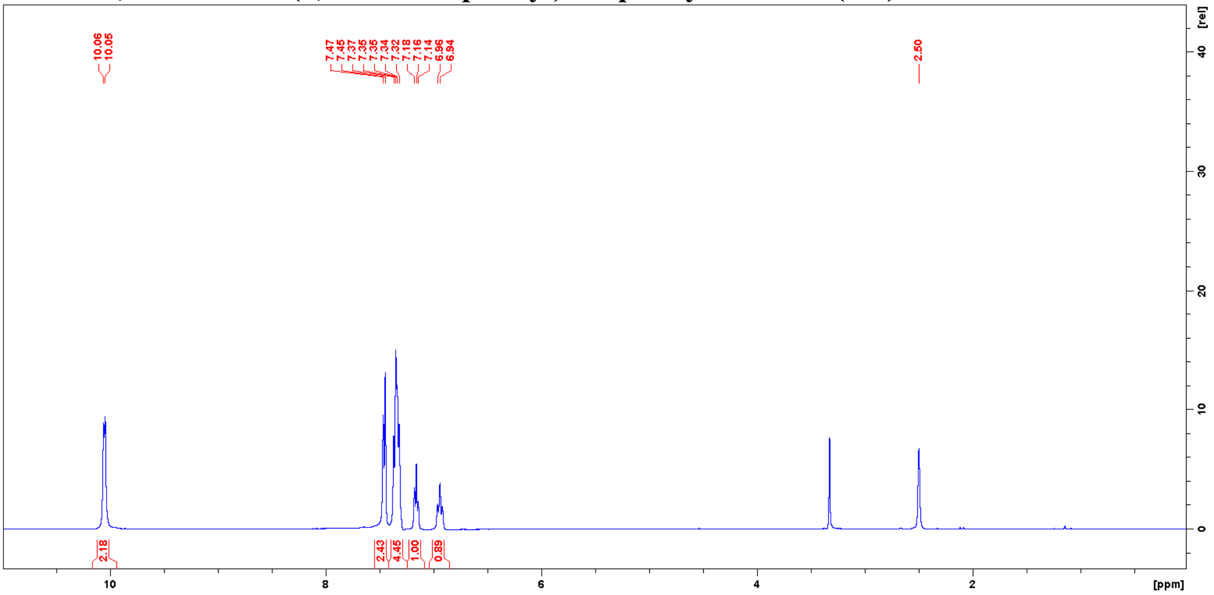

^{13}C-NMR spectrum of ***N*-(3,5-difluorophenyl)-*N'*-phenylthiourea (12f)**

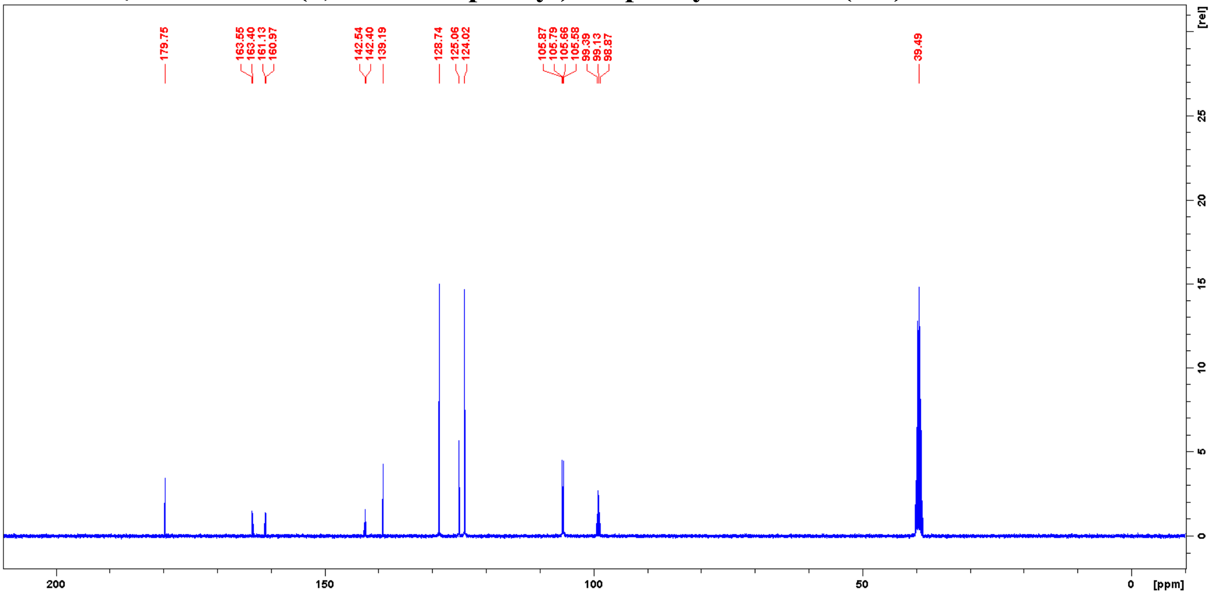

^{1}H-NMR spectrum of ***N*-(2,6-difluorophenyl)-*N'*-phenylthiourea (12g)**

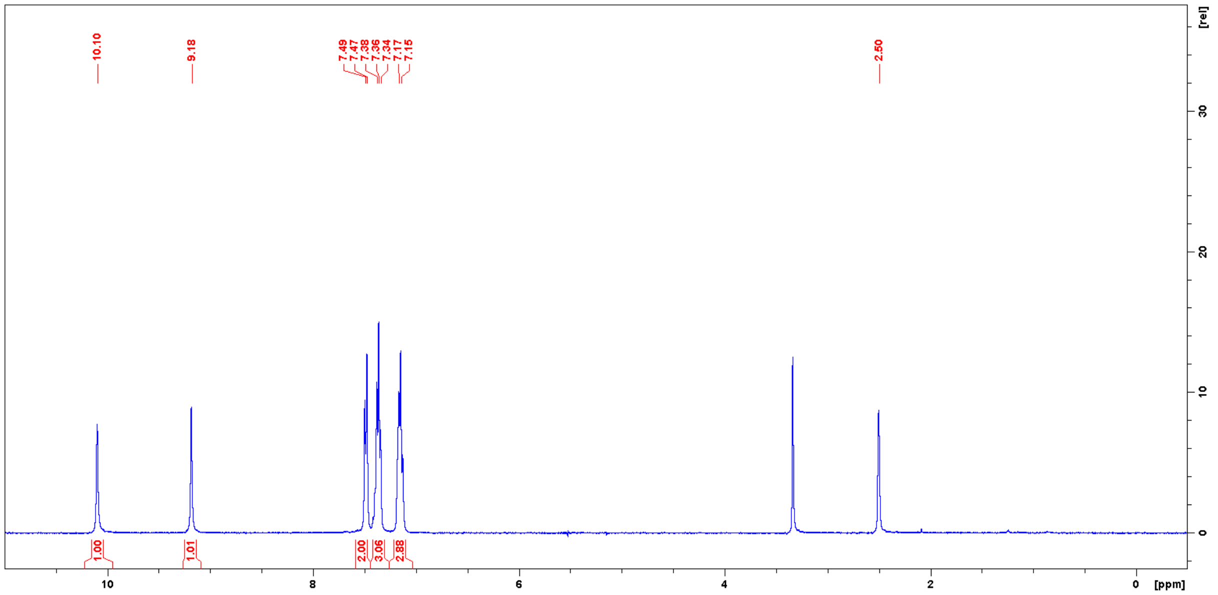

^{13}C-NMR spectrum of ***N*-(2,6-difluorophenyl)-*N'*-phenylthiourea (12g)**

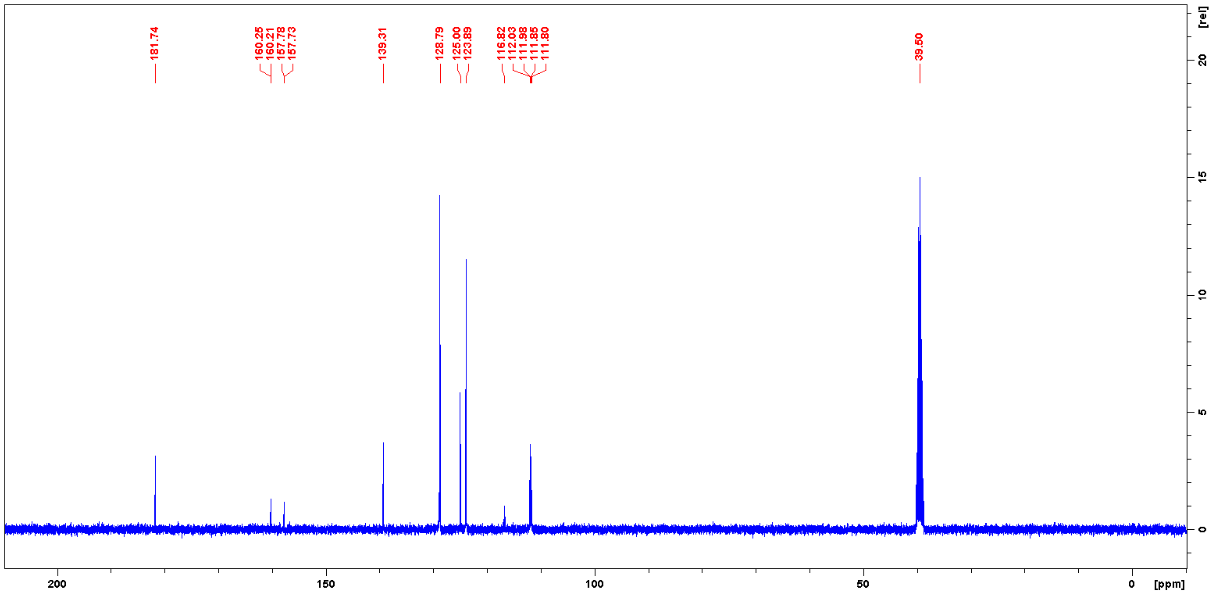

Methyl (2*S*)-3-methyl-2-({2-[(phenylcarbamothioyl)amino]benzoyl}amino)butanoate (9a)

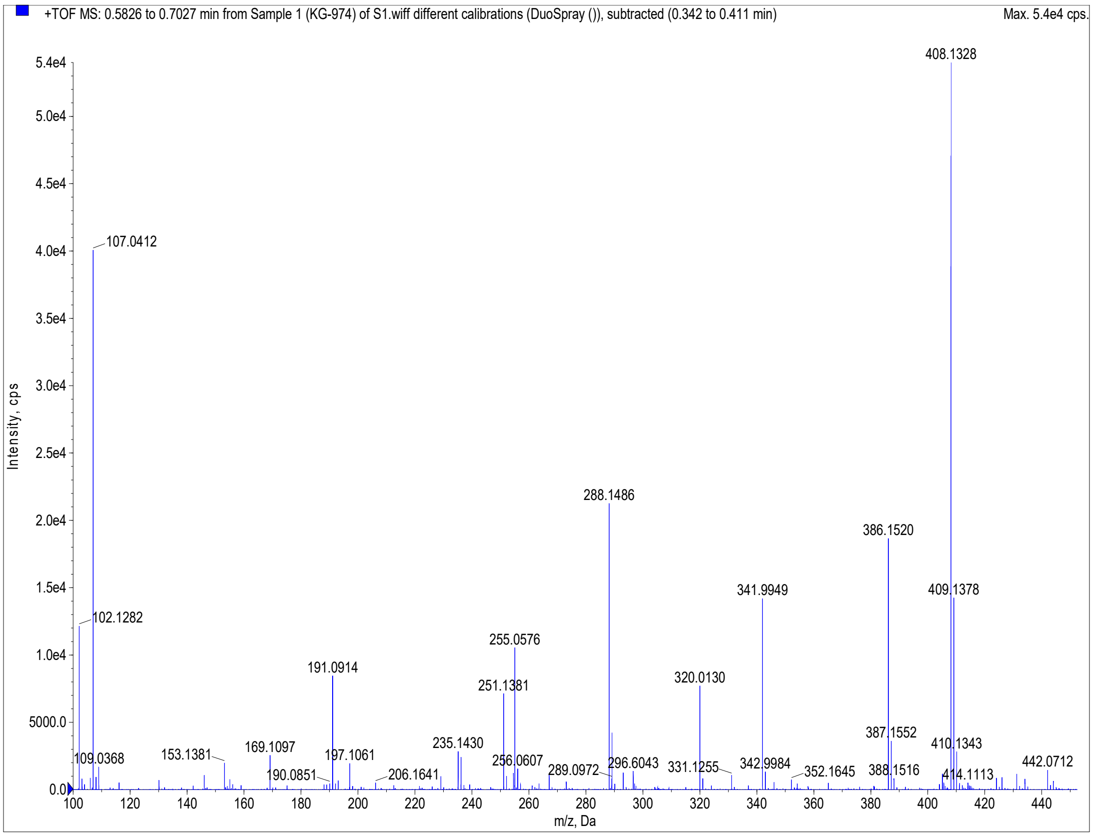

Formula	Calculated mass	Error / mDa	Error / ppm	DBE
C20 H24 N3 O3 S	386.1538	-1.8387	-4.7616	10.5
C20 H23 N3 O3 Na S	408.1357	-2.9833	-7.3097	10.5

Methyl (2*S*)-3-methyl 2-[(2-{[(4-nitrophenyl)carbamothioyl]amino}benzoyl)amino]butanoate (9b)

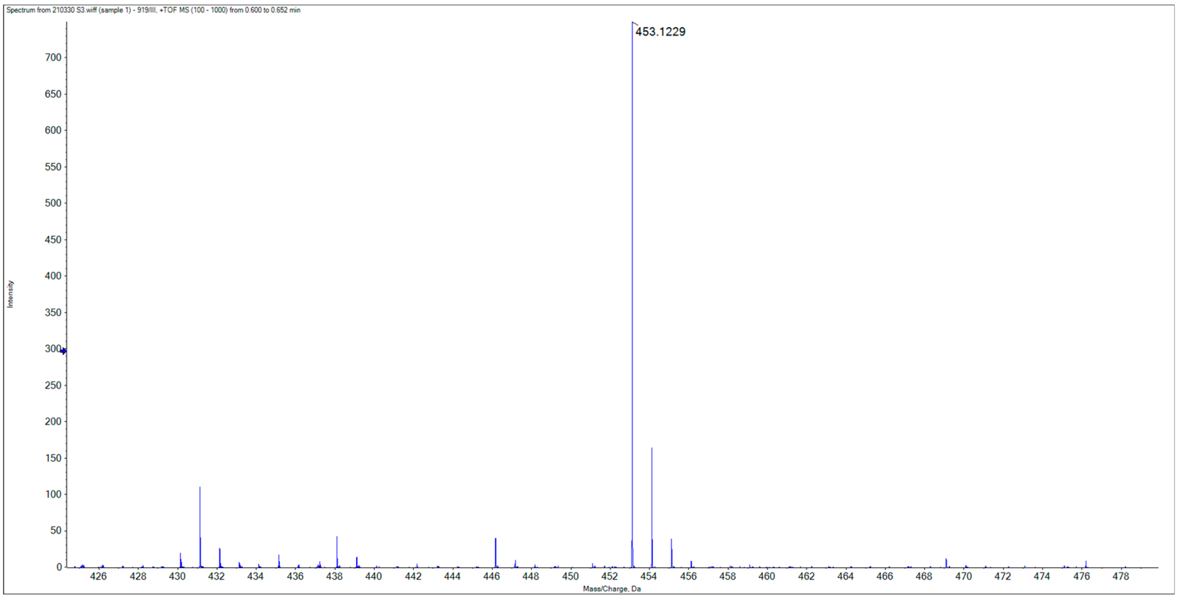

Formula	Calculated mass	Error / ppm	Error / mDa	DBE
C20H22N4NaO5S	453.12031	5.7	2.57	12.0

Methyl (2*S*)-3-methyl 2-[(2-{[(3,5-bistrifluoromethylphenyl)carbamothioyl]amino} benzoyl)amino]butanoate (9c)

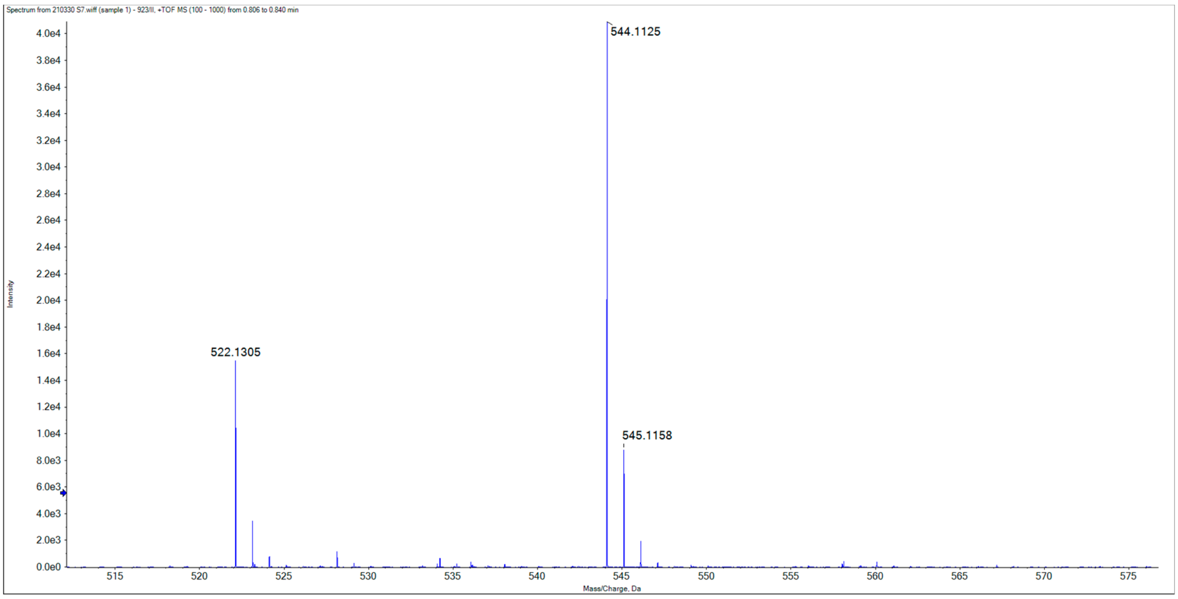

Formula	Calculated mass	Error / ppm	Error / mDa	DBE
C22H22F6N3O3S	522.12806	4.6	2.39	11.0
C22H21F6N3NaO3S	544.11000	4.6	2.51	11.0

Methyl (2*S*)-3-methyl 2-[(2-{[(4-methoxyphenyl)carbamothioyl]amino}benzoyl)amino]butanoate (9d)

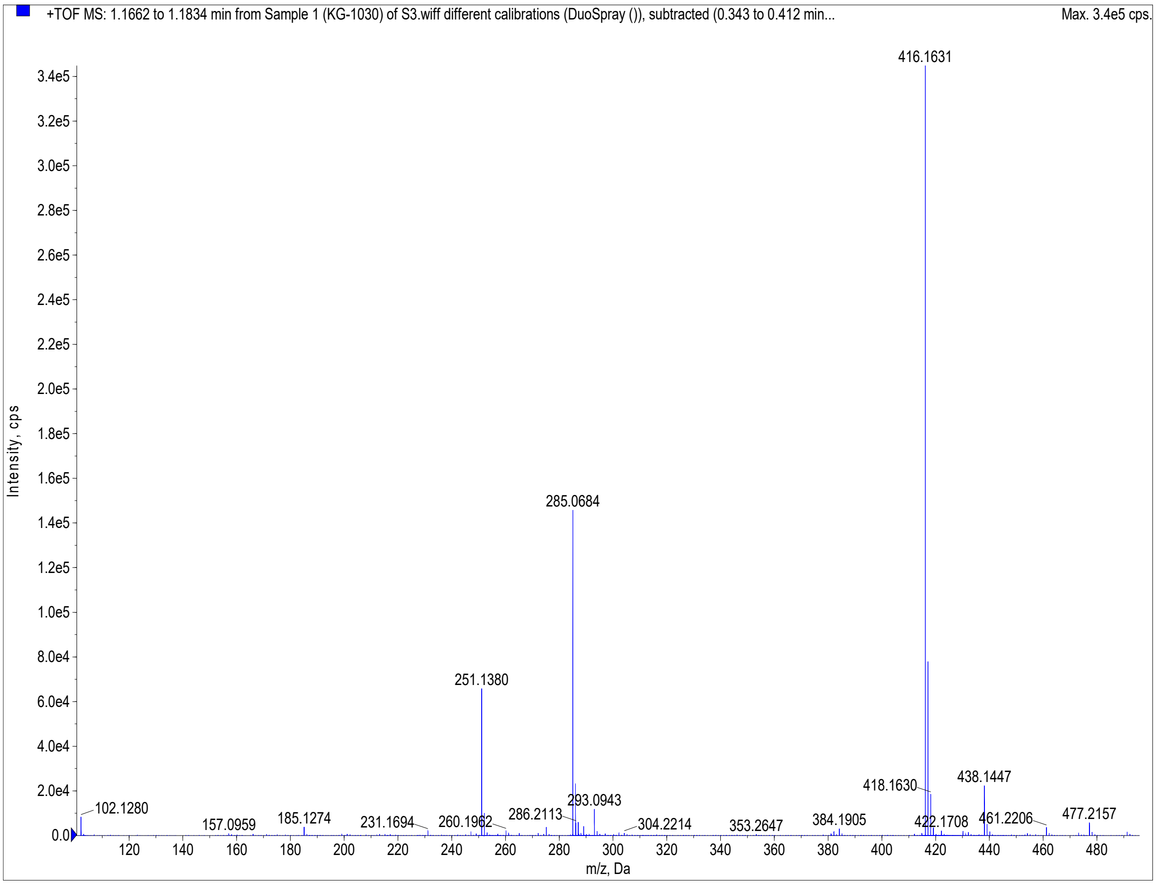

Formula	Calculated mass	Error / mDa	Error / ppm	DBE
C21 H26 N3 O4 S	416.1644	-1.3034	-3.132	10.5

Methyl (2*S*)-3-methyl 2-[(2-{[(4-fluorophenyl)carbamothioyl]amino}benzoyl)amino]butanoate (9e)

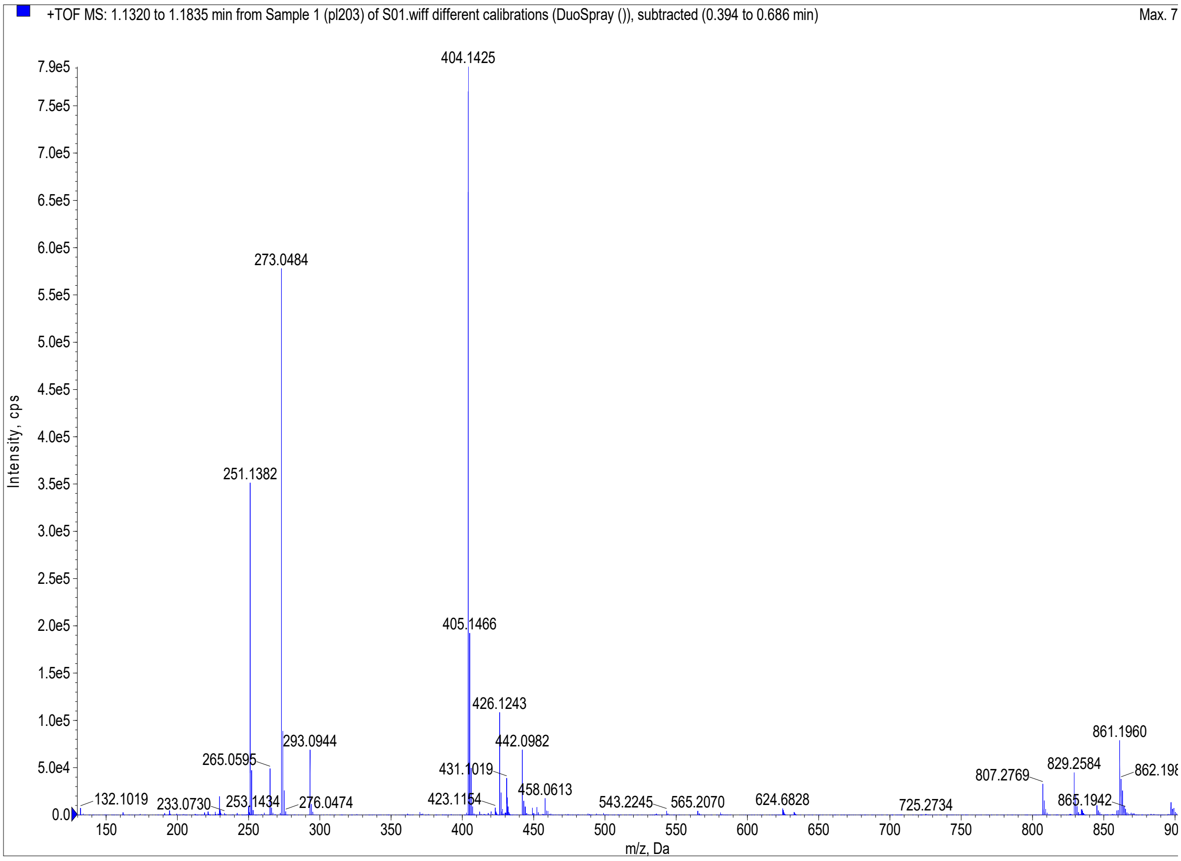

Formula	Calculated mass	Error / mDa	Error / ppm	DBE
C20 H23 N3 O3 F S	404.1438	-1.9169	-4.7431	10.5

Methyl (2*S*)-3-methyl 2-[(2-{[(3,5-difluorophenyl)carbamothioyl]amino}benzoyl)amino]butanoate (9f)

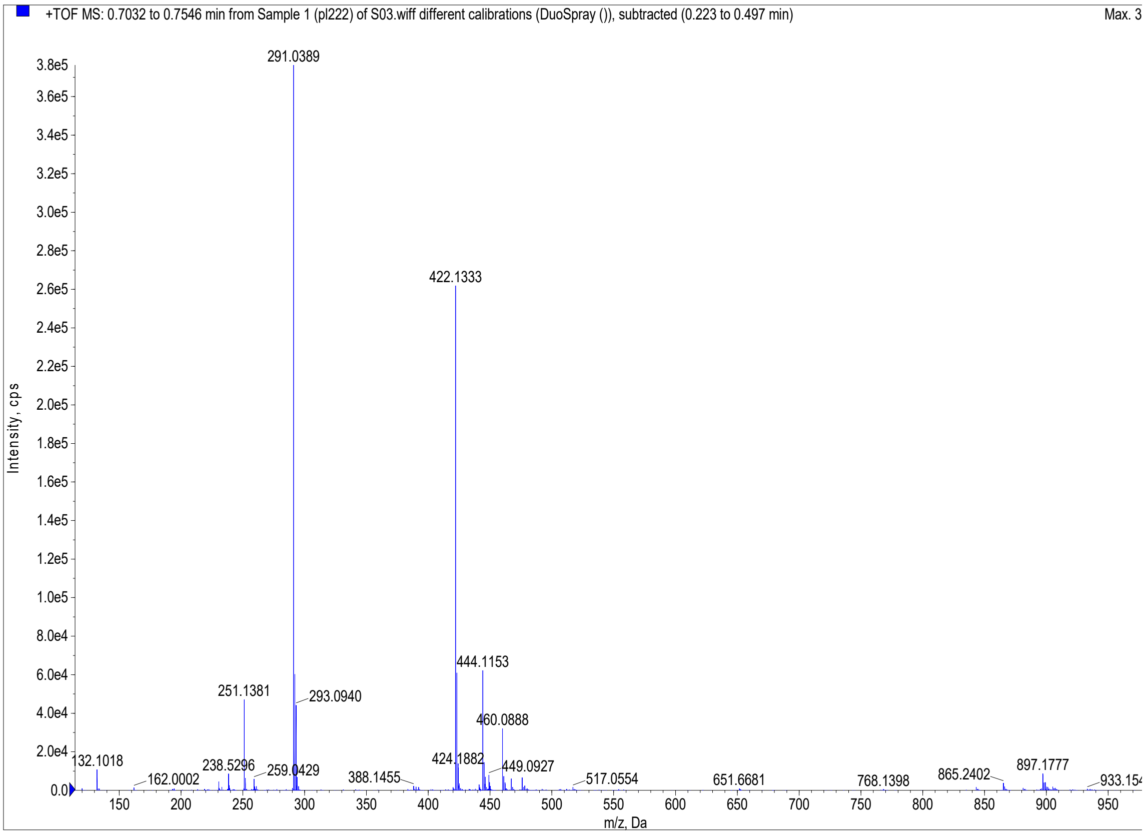

Formula	Calculated mass	Error / mDa	Error / ppm	DBE
C20 H22 N3 O3 F2 S	422.1344	-1.6951	-4.0156	10.5

Methyl (2*S*)-3-methyl 2-[(2-{[(2,6-difluorophenyl)carbamothioyl]amino}benzoyl)amino]butanoate (9g)

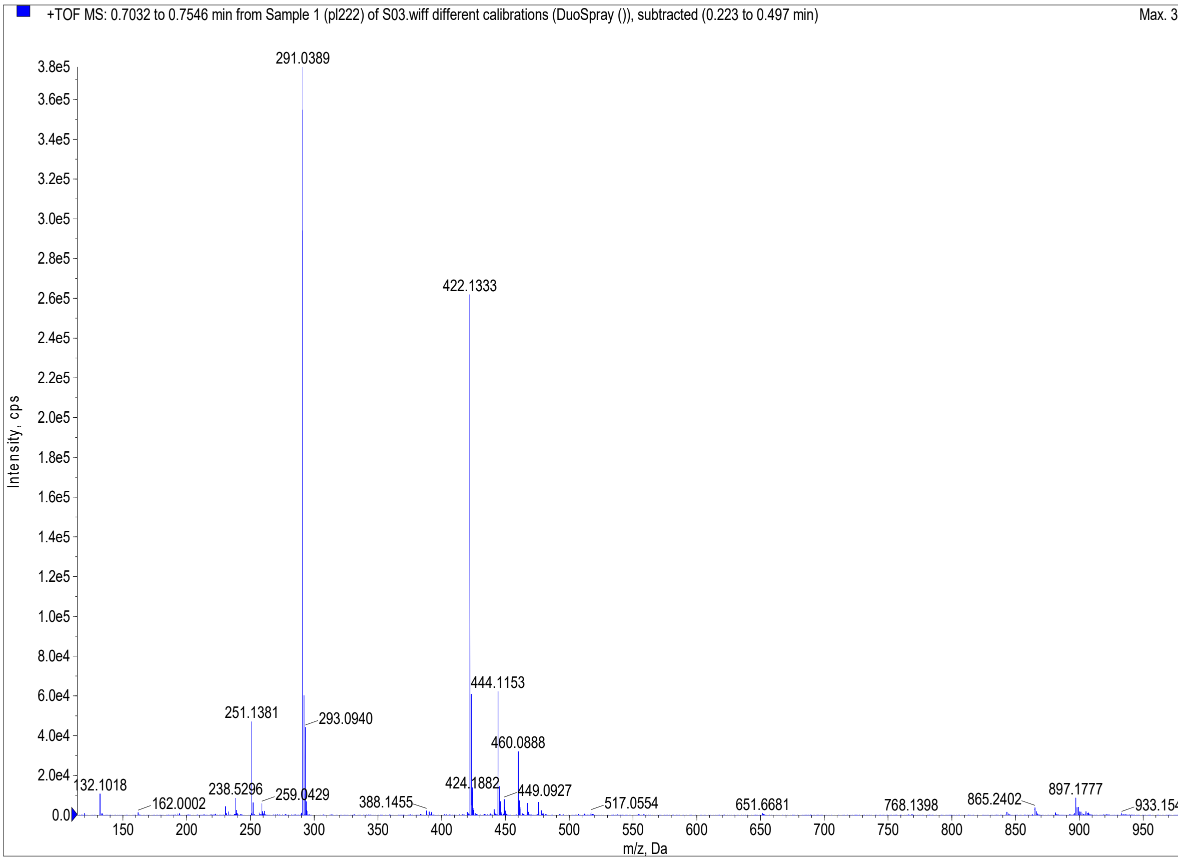

Formula	Calculated mass	Error / mDa	Error / ppm	DBE
C20 H22 N3 O3 F2 S	422.1344	-1.6951	-4.0156	10.5

Methyl (2*S*)-4-methyl 2-({2-[(phenylcarbamothioyl)amino]benzoyl}amino)pentanoate (10a)

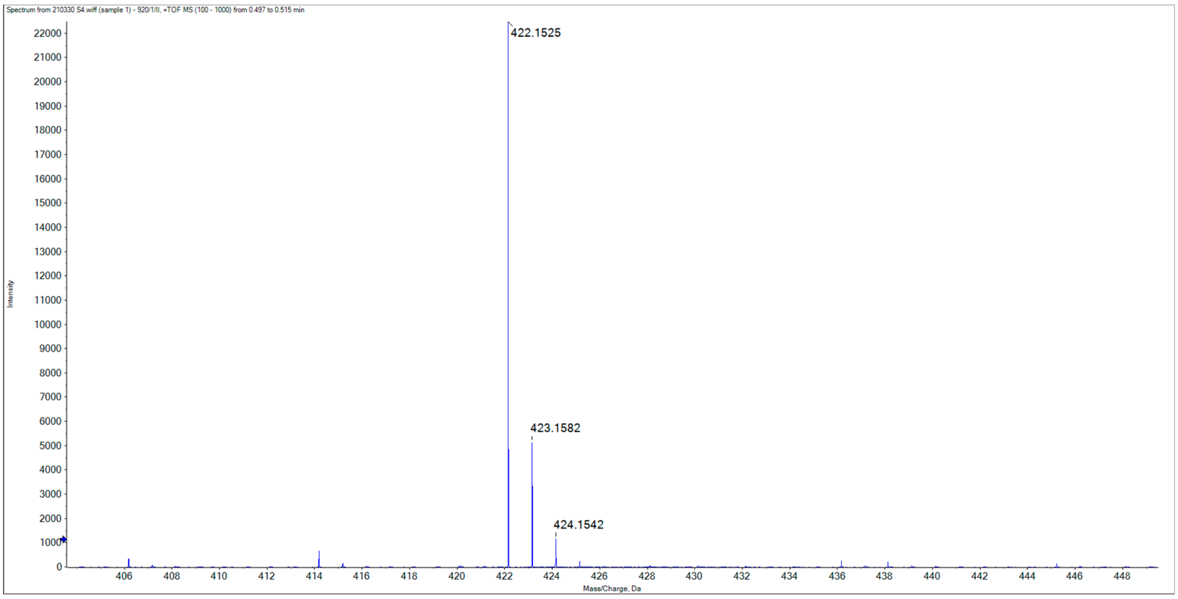

Formula	Calculated mass	Error / ppm	Error / mDa	DBE
C21H25N3NaO3S	422.15088	3.9	1.66	11.0

Methyl (2*S*)-4-methyl 2-[(2-{[(4-nitrophenyl)carbamothioyl]amino}benzoyl)amino]pentanoate (10b)

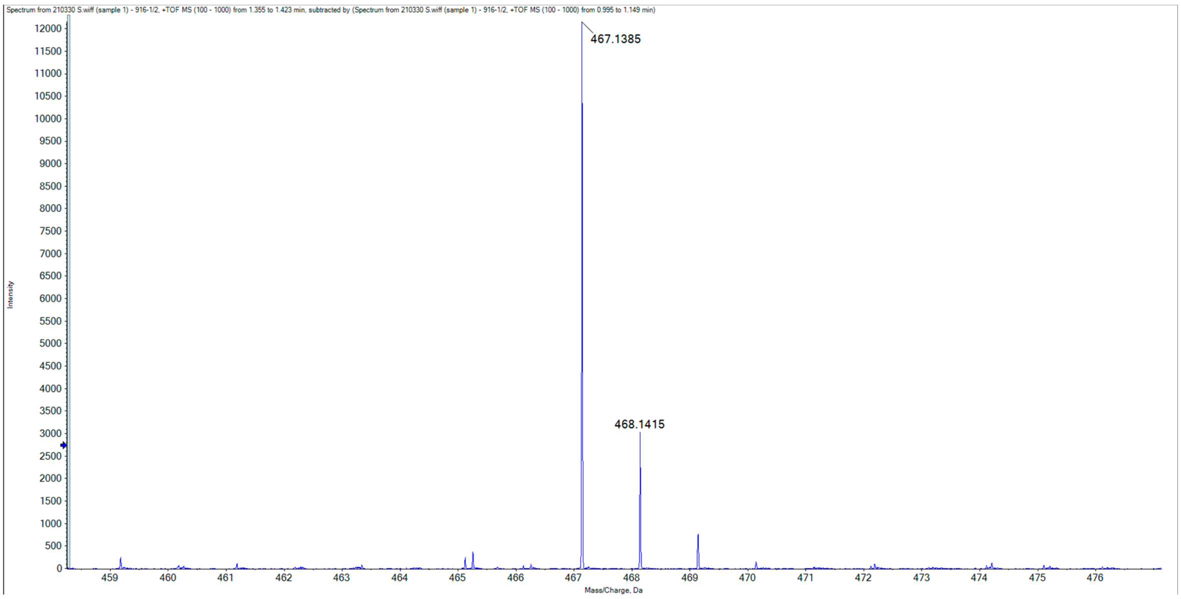

Formula	Calculated mass	Error / ppm	Error / mDa	DBE
C21H24N4NaO5S	467.13596	5.4	2.53	12.0

Methyl (2*S*)-4-methyl 2-[(2-{[(3,5-bistrifluoromethylphenyl)carbamothioyl]amino}-benzoyl)amino]pentanoate (10c)

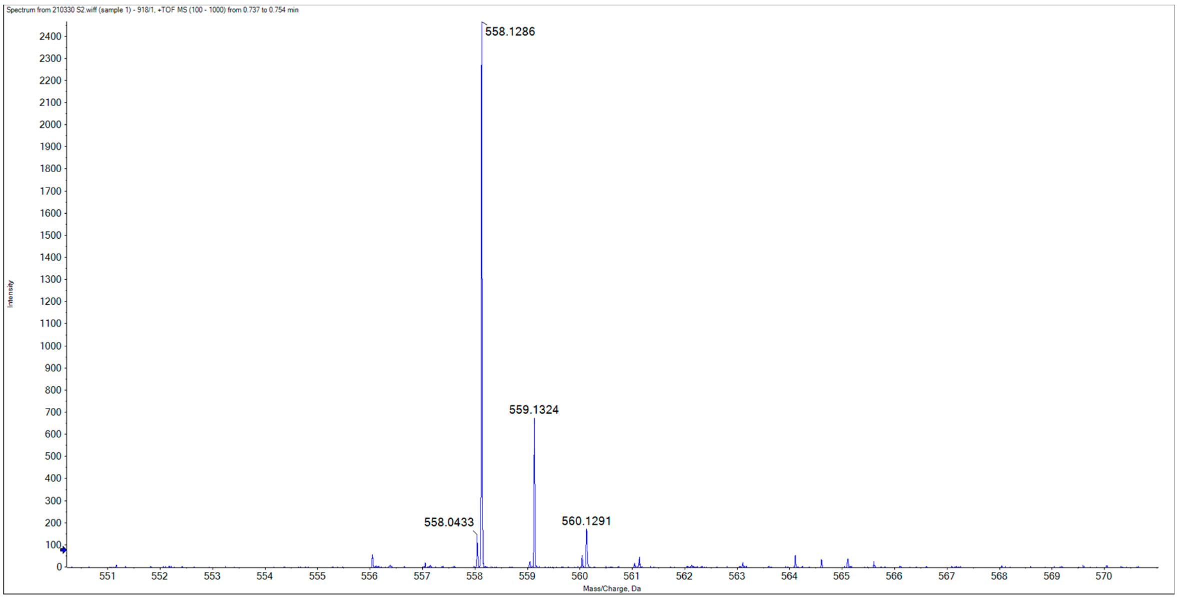

Formula	Calculated mass	Error / ppm	Error / mDa	DBE
C23H23F6N3NaO3S	558.12565	5.3	2.99	11.0

Methyl (2*S*)-4-methyl 2-[(2-{[(4-methoxyphenyl)carbamothioyl]amino}benzoyl)amino]pentanoate (10d)

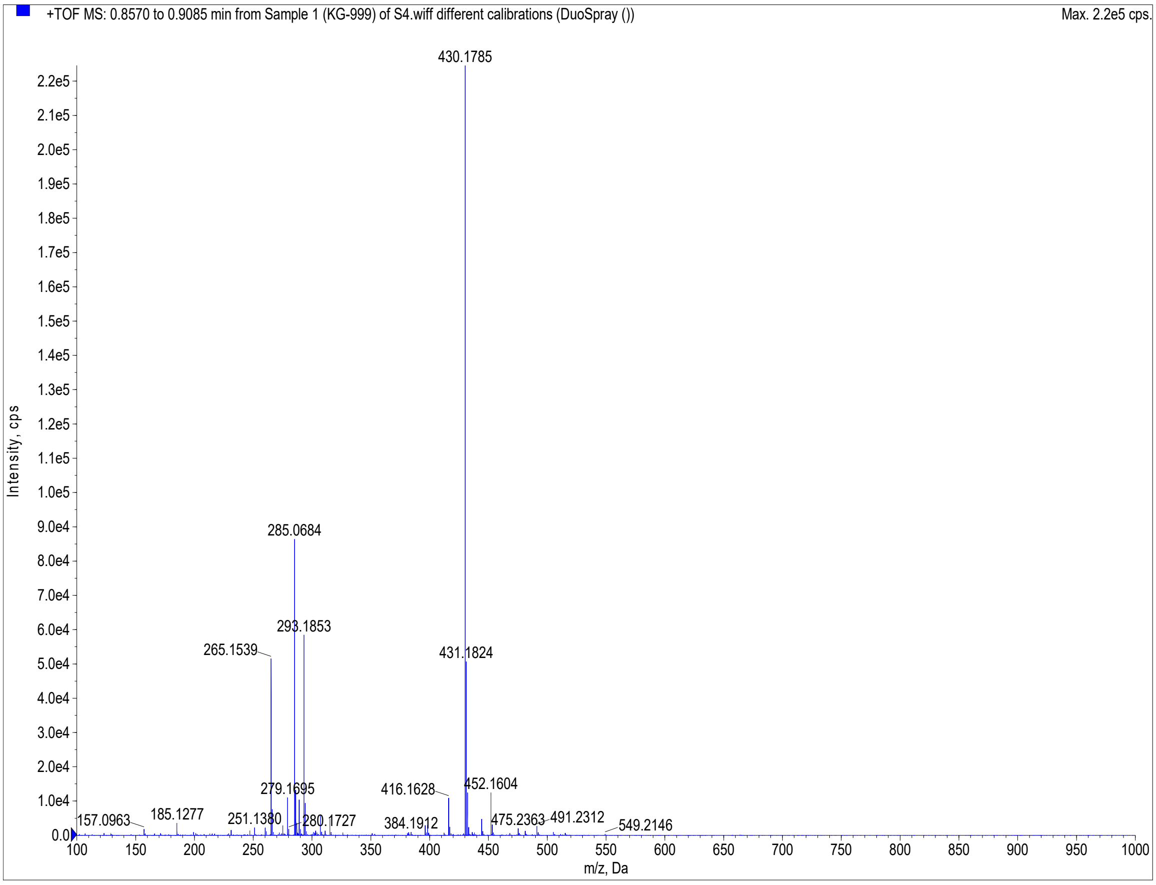

Formula	Calculated mass	Error / mDa	Error / ppm	DBE
C22 H28 N3 O4 S	430.18	-1.5535	-3.6113	10.5

Methyl (2*S*)-4-methyl 2-[(2-{[(4-fluorophenyl)carbamothioyl]amino}benzoyl)amino]pentanoate (10e)

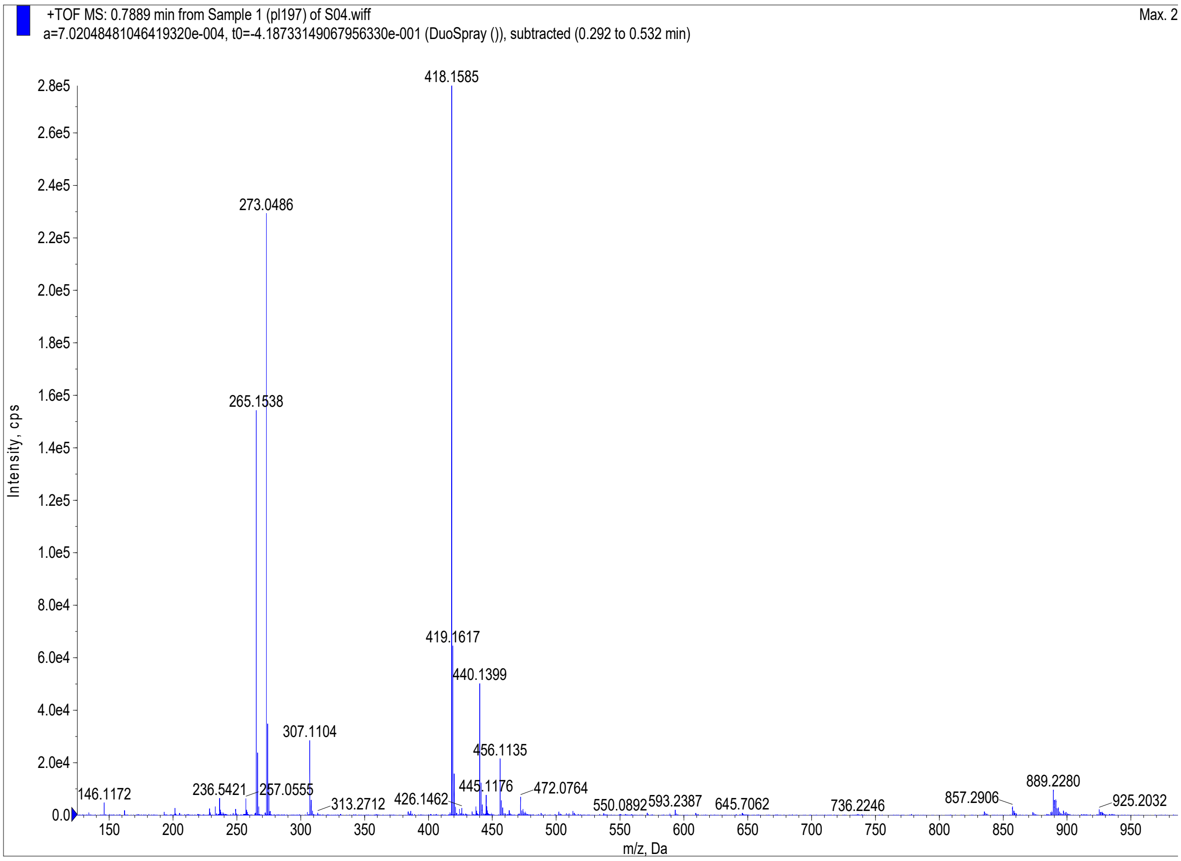

Formula	Calculated mass	Error / mDa	Error / ppm	DBE
C21 H25 N3 O3 F S	418.1595	-1.5669	-3.7473	10.5

Methyl (2*S*)-4-methyl 2-[(2-{[(3,5-difluorophenyl)carbamothioyl]amino}benzoyl)amino]pentanoate (10f)

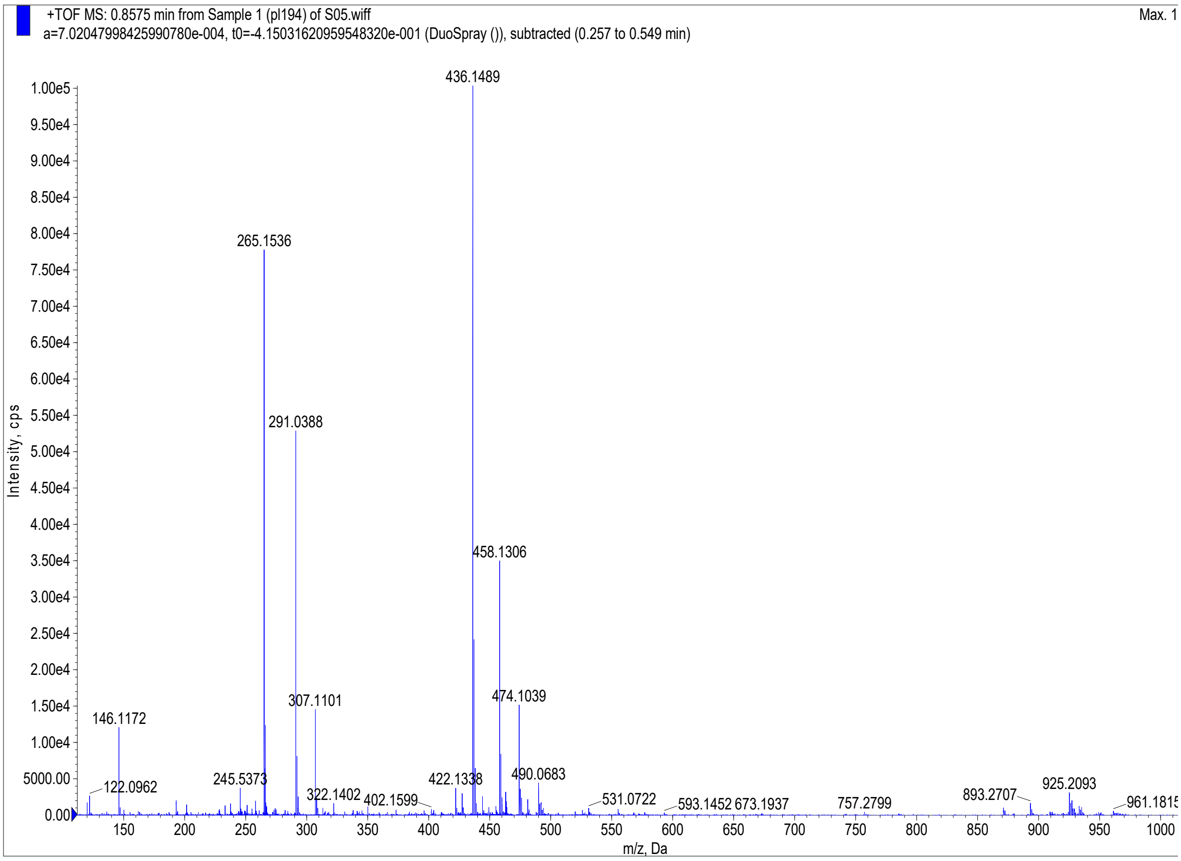

Formula	Calculated mass	Error / mDa	Error / ppm	DBE
C21 H24 N3 O3 F2 S	436.15	-1.7452	-4.0013	10.5

Methyl (2*S*)-4-methyl 2-[(2-{[(2,6-difluorophenyl)carbamothioyl]amino}benzoyl)amino]pentanoate (10g)

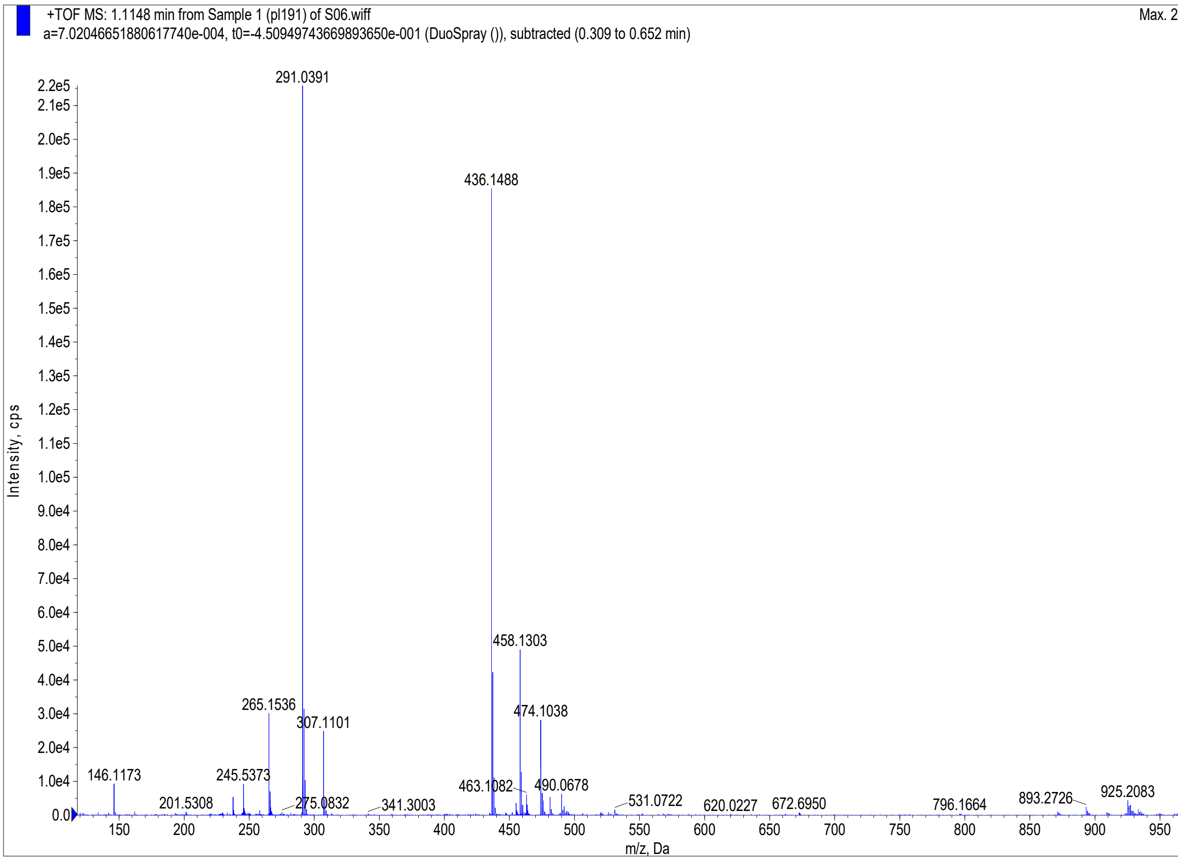

Formula	Calculated mass	Error / mDa	Error / ppm	DBE
C21 H24 N3 O3 F2 S	436.15	-1.8452	-4.2306	10.5

Methyl (2*S*)-3-methyl 2-({2-[(phenylcarbamothioyl)amino]benzoyl}amino)pentanoate (11a)

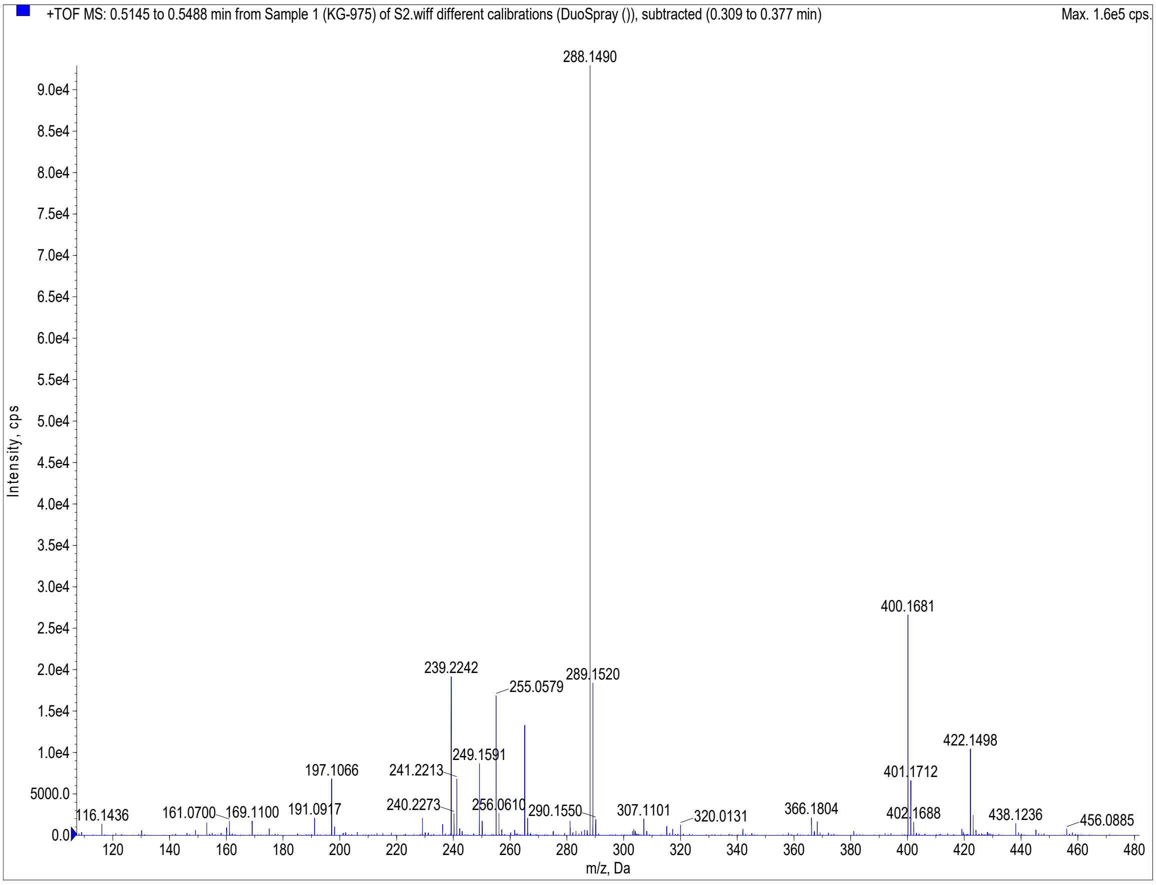

Formula	Calculated mass	Error / mDa	Error / ppm	DBE
C21 H26 N3 O3 S	400.1694	-1.3887	-3.4705	10.5
C21 H25 N3 O3 Na S	422.1514	-1.6334	-3.8693	10.5

Methyl (2*S*)-3-methyl 2-[(2-{[(4-nitrophenyl)carbamothioyl]amino}benzoyl)amino]pentanoate (11b)

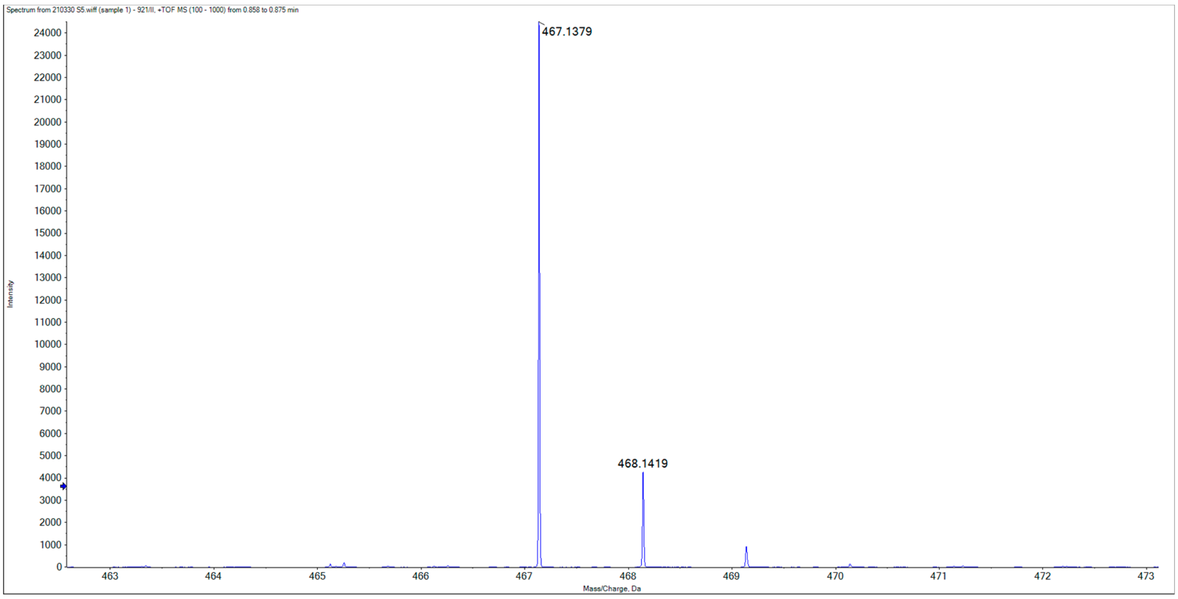

Formula	Calculated mass	Error / ppm	Error / mDa	DBE
C21H24N4NaO5S	467.13596	4.1	1.94	12.0

Methyl (2*S*)-3-methyl 2-[(2-{[(3,5-bistetrifluoromethylphenyl)carbamothioyl]amino} benzoyl)amino]pentanoate (11c)

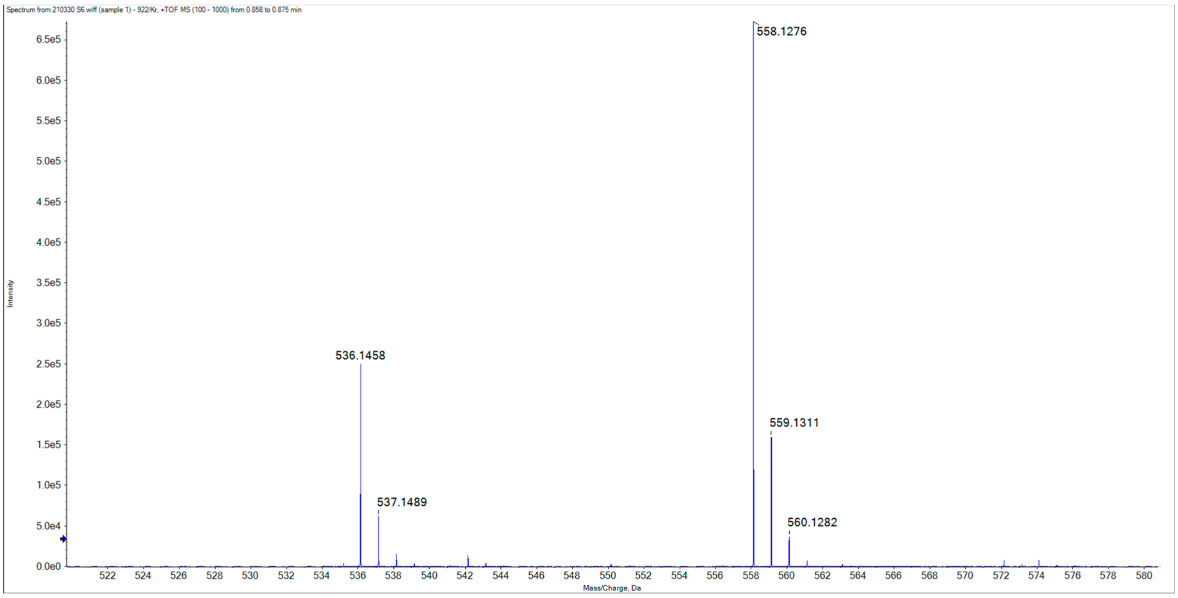

Formula	Calculated mass	Error / ppm	Error / mDa	DBE
C23H23F6N3NaO3S	558.12565	3.4	1.91	11.0
C23H24F6N3O3S	536.14371	4.0	2.13	11.0
